# Cascade Synthesis of Structurally Diverse B–X-Doped Polycyclic Aromatic Hydrocarbons

**DOI:** 10.1021/acs.orglett.6c03304

**Published:** 2026-09-02

**Authors:** Marcos Humanes, Jaime Mateos-Gil, Francisco Mendicuti, Manuel A. Fernández-Rodríguez, Patricia García-García

**Affiliations:** † Universidad de Alcalá (IRYCIS), Departamento de Química Orgánica y Química Inorgánica, Instituto de Investigación Química “Andrés M. del Río” (IQAR), 28805 Alcalá de Henares, Madrid, Spain; ‡ Universidad de Alcalá, Departamento de Química Analítica, Química Física e Ingeniería Química, Instituto de Investigación Química “Andrés M. del Río” (IQAR), 28805 Alcalá de Henares, Madrid, Spain

## Abstract

A modular, metal-free, cascade cyclization of functionalized enynes provides rapid access to structurally diverse boron-doped polycyclic aromatic hydrocarbons. The method simultaneously constructs the polycyclic framework and B–O, B–N, or C–B bonds while allowing straightforward boron functionalization. The alkene substitution pattern dictates the cyclization pathway, enabling selective formation of five- or six-membered rings. Photophysical studies reveal how structural variations govern the optical and fluorescence properties of these π-conjugated materials.

## Graphical abstract

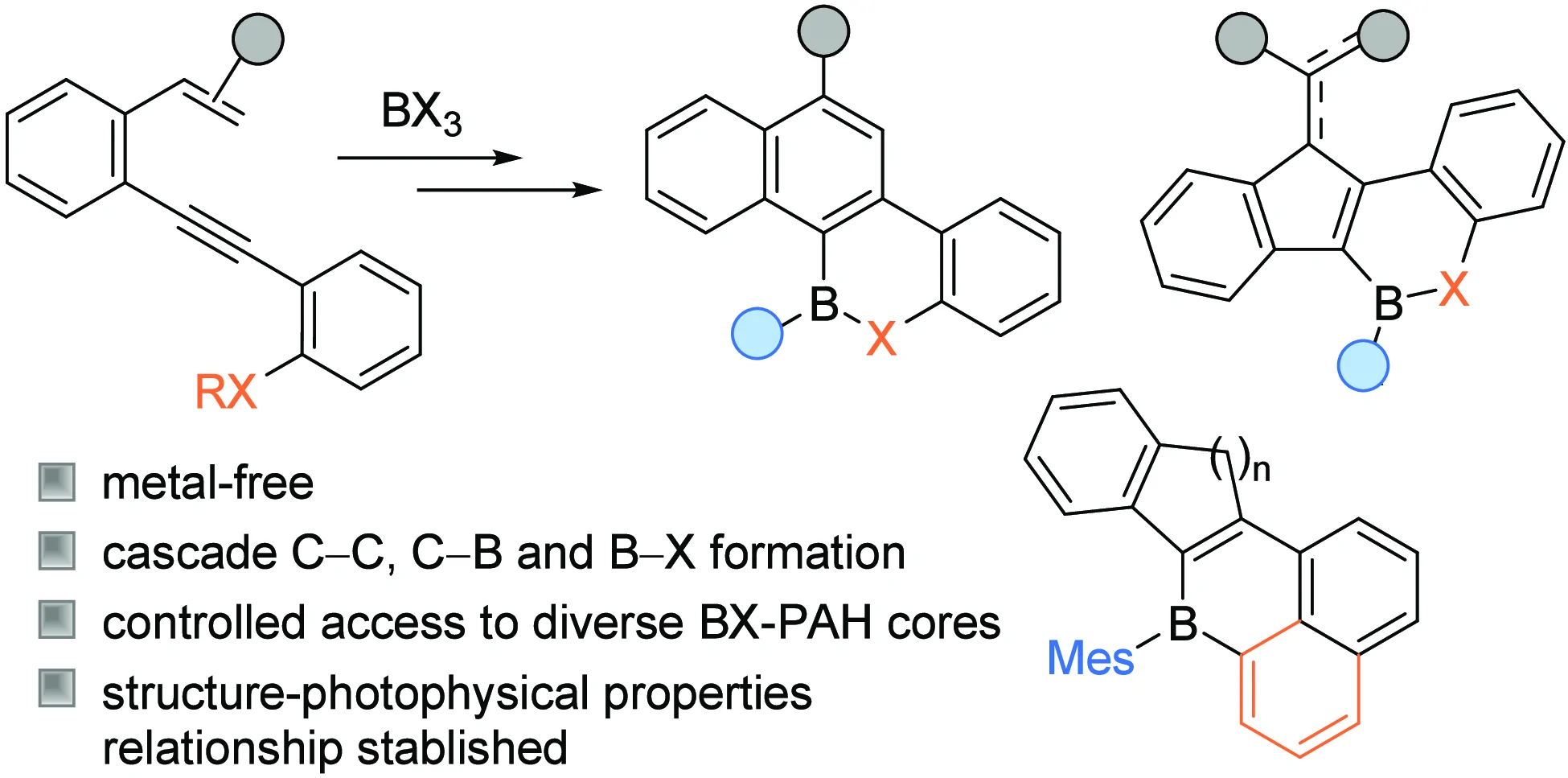

The controlled incorporation of boron into organic molecules has attracted growing attention across a wide range of disciplines.^1^ In particular, the introduction of B–O and B–N bonds within cyclic frameworks has emerged as an effective strategy for the design of novel molecules with valuable applications in medicinal chemistry and materials science (Figure 1). Among these motifs, cyclic boronates stand out as promising scaffolds for drug development,^2^ with several candidates in advanced clinical trials as β-lactamase inhibitors, such as xeruborbactam and ledaborbactam.^3^ Inhibition of β-amyloid aggregation in Alzheimer’s disease has also been reported for compounds containing this substructure.^4^ Although related systems featuring B–N bonds have been explored to a lesser extent, they have likewise demonstrated considerable promise in medicinal chemistry.^5^ Beyond their pharmaceutical significance, compounds containing B–X bonds play a central role in the development of functional organic materials. The formal replacement of a C=C bond in polycyclic aromatic hydrocarbons (PAHs) by an isoelectronic yet polarized B–N unit has become a well-established approach for fine-tuning the electronic and photophysical properties of π-conjugated systems.^6^ As a result, polycyclic azaborines have found widespread application in optoelectronic devices, including organic light-emitting diodes (OLEDs), organic field-effect transistors (OFETs), and organic photovoltaic cells.^7^ In contrast, the incorporation of B–O bonds into PAHs has been comparatively less developed and, only in recent years, has begun to attract increasing interest as a complementary strategy for the design of advanced materials.^8^

**Figure 1:**
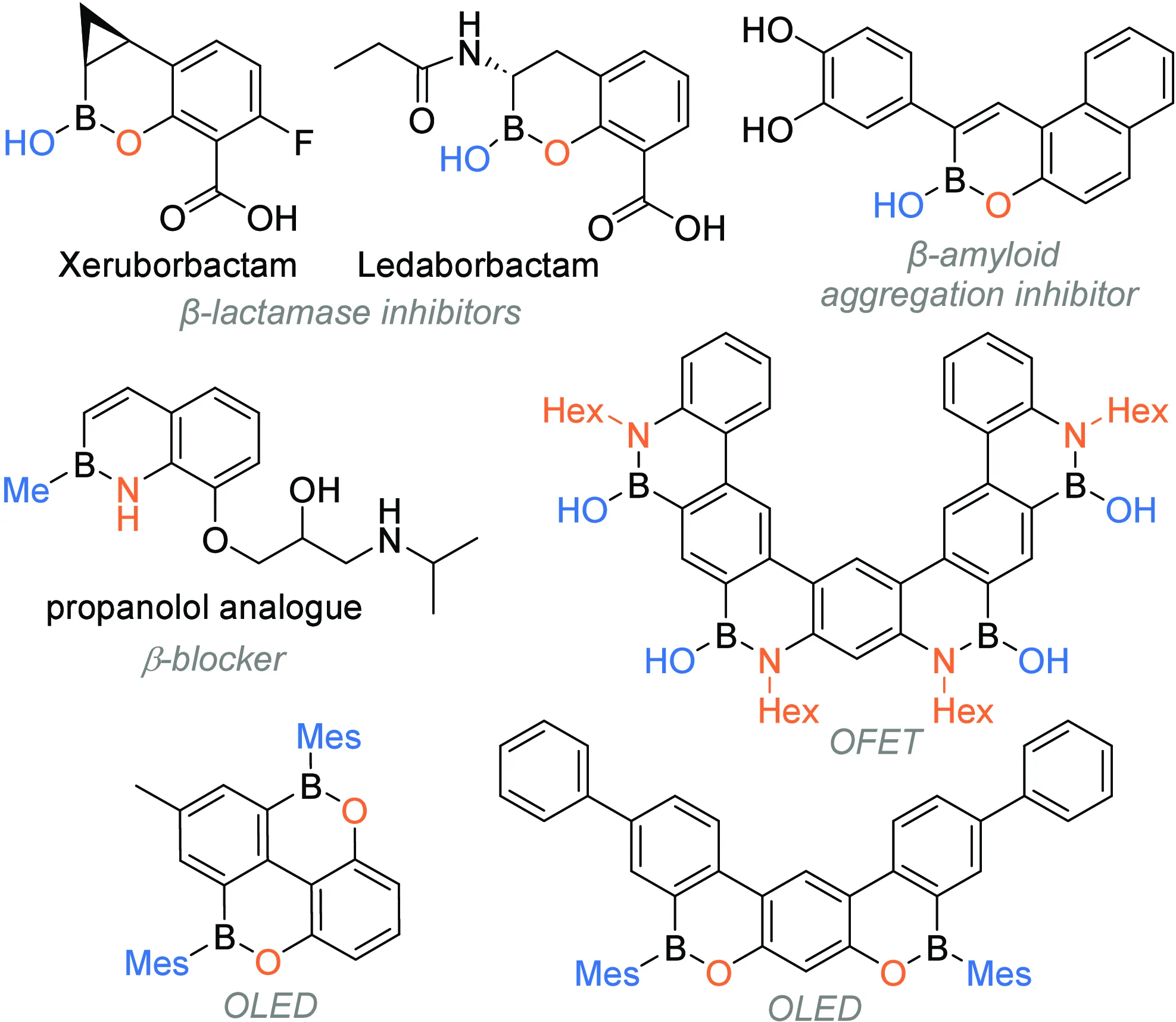
Selected B–X-containing molecules with applications in medicine and materials science.

The growing interest in cyclic compounds containing B–X bonds has made the development of efficient synthetic methods a major goal. Several strategies for the synthesis of bicyclic boronates have been reported recently.^9^ Although these methods have enabled the rapid synthesis of scaffolds of considerable relevance to medicinal chemistry, their applicability to materials science remains limited, as more extensively π-conjugated frameworks are usually required in this field. In this regard, the synthesis of B–O-doped polycyclic aromatic hydrocarbons (BO-PAHs) has traditionally relied on electrophilic borylation of arenes using highly reactive BBr_3_, typically followed by thermal cyclization under forcing conditions, often in the presence of AlCl_3_ and/or at elevated temperatures.^8,10^ Milder alternatives based on gold-catalyzed,^11^ nickel-catalyzed,^12^ or palladium-catalyzed^13^ processes have been described as well. Notably, although the nature of the boron substituent can strongly influence the optoelectronic properties of the resulting materials, variation at this position has not been systematically investigated in most of the reported methodologies. Therefore, the development of general, modular, and mild methodologies for the synthesis of cyclic B–X-containing compounds and especially B–X-doped PAHs remains of high interest.

Herein, we propose a modular and metal-free strategy for the synthesis of B-doped PAHs based on a cascade cyclization of appropriately functionalized enynes (Scheme 1A). This transformation would enable the simultaneous construction of the fused polycyclic framework and the B–X bond in a single operation using BCl_3_ as a promoter.^14^ Building on our previous experience on the electrophilic cyclization of enynes,^15^ we envisioned that the substitution pattern of the alkene moiety could control the selectivity of the cyclization, providing scaffolds containing five- or six-membered rings on demand. In addition, both the heteroatom incorporated into the boron-containing ring and the substituent bound to boron could be readily tuned through an appropriate choice of the enyne precursor and the reagent employed in the final quenching step.^16^

**Scheme 1:**
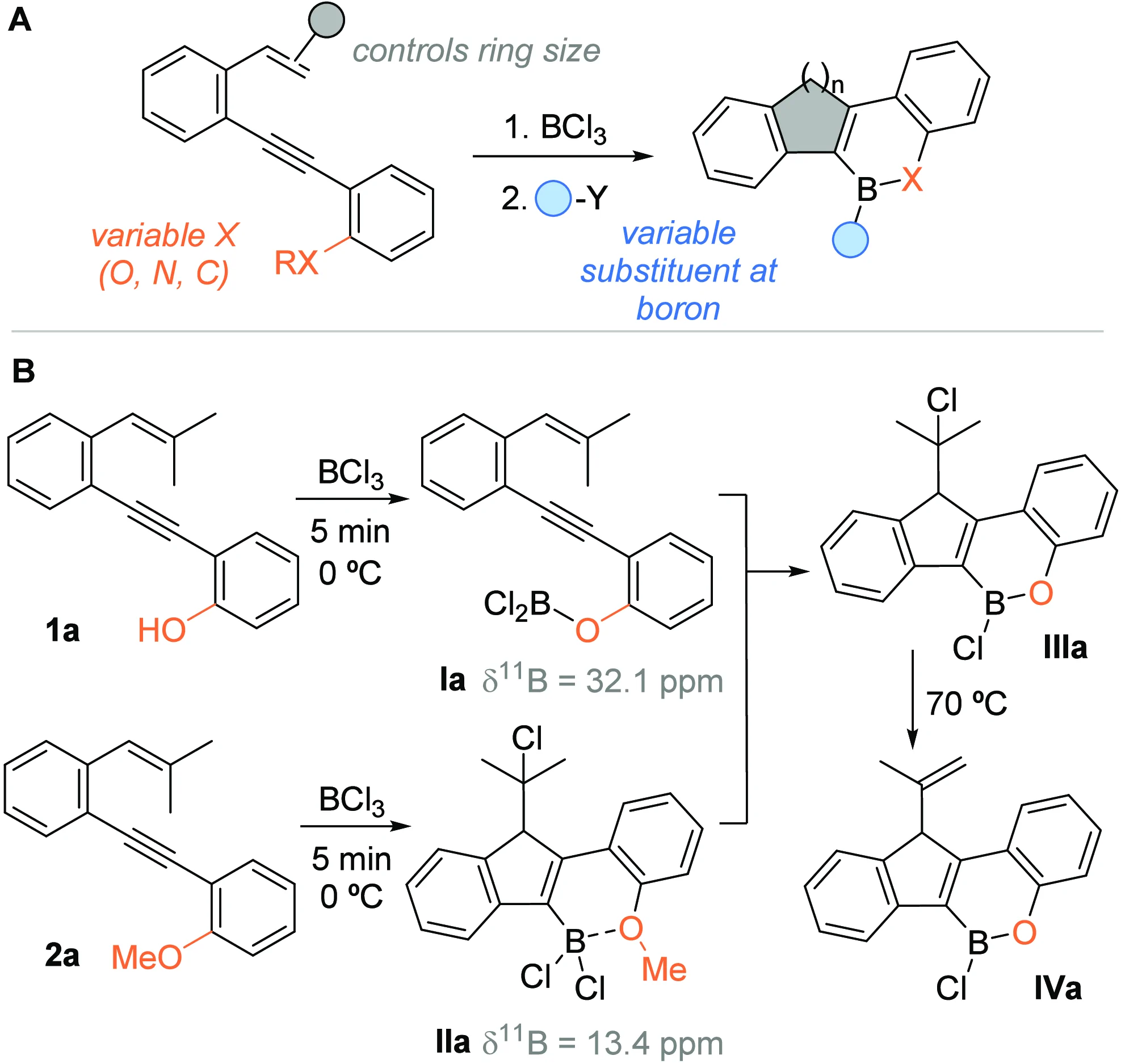
Proposed Modular Synthesis of B–X-Doped PAHs and Preliminary Results

To evaluate the feasibility of the proposed cascade process, we conducted preliminary experiments using enynes **1a** and **2a** as model substrates (Scheme 1B).^17^ Treatment of **1a**, bearing a hydroxyl group, with 3 equiv of BCl_3_ at 0 °C led to the immediate formation of intermediate **Ia**, as evidenced by ^1^H and ^11^B NMR.^18^ These data indicate coordination of boron to the oxygen atom, while the enyne framework remains unchanged. Upon continued stirring at this temperature, **Ia** was gradually converted into polycycle **IIIa**, thereby validating our approach. In contrast, treatment of substrate **2a**, which contains a methoxy group, with BCl_3_ triggered enyne cyclization as the initial step, affording intermediate **IIa**.^19^ Upon prolonged reaction times, **IIa** releases methyl chloride to furnish the same B–O-doped tetracyclic scaffold **IIIa**. In both cases, minor amounts of alkenyl-substituted B–O-doped benzo­[*a*]­fluorene derivative **IVa** were detected in the crude reaction mixtures. This compound arises from elimination of HCl from intermediate **IIIa**, a process that proved difficult to suppress completely. However, heating at 70 °C effectively promotes this transformation, enabling the selective formation of **IVa** either from **1a** or **2a**, although the reaction of methoxy-substituted enyne **2a** proceeded more cleanly. Based on these results, the substrate scope of the cascade cyclization was next evaluated using methoxy-substituted enynes **2** at 70 °C (Scheme 2).

**Scheme 2:**
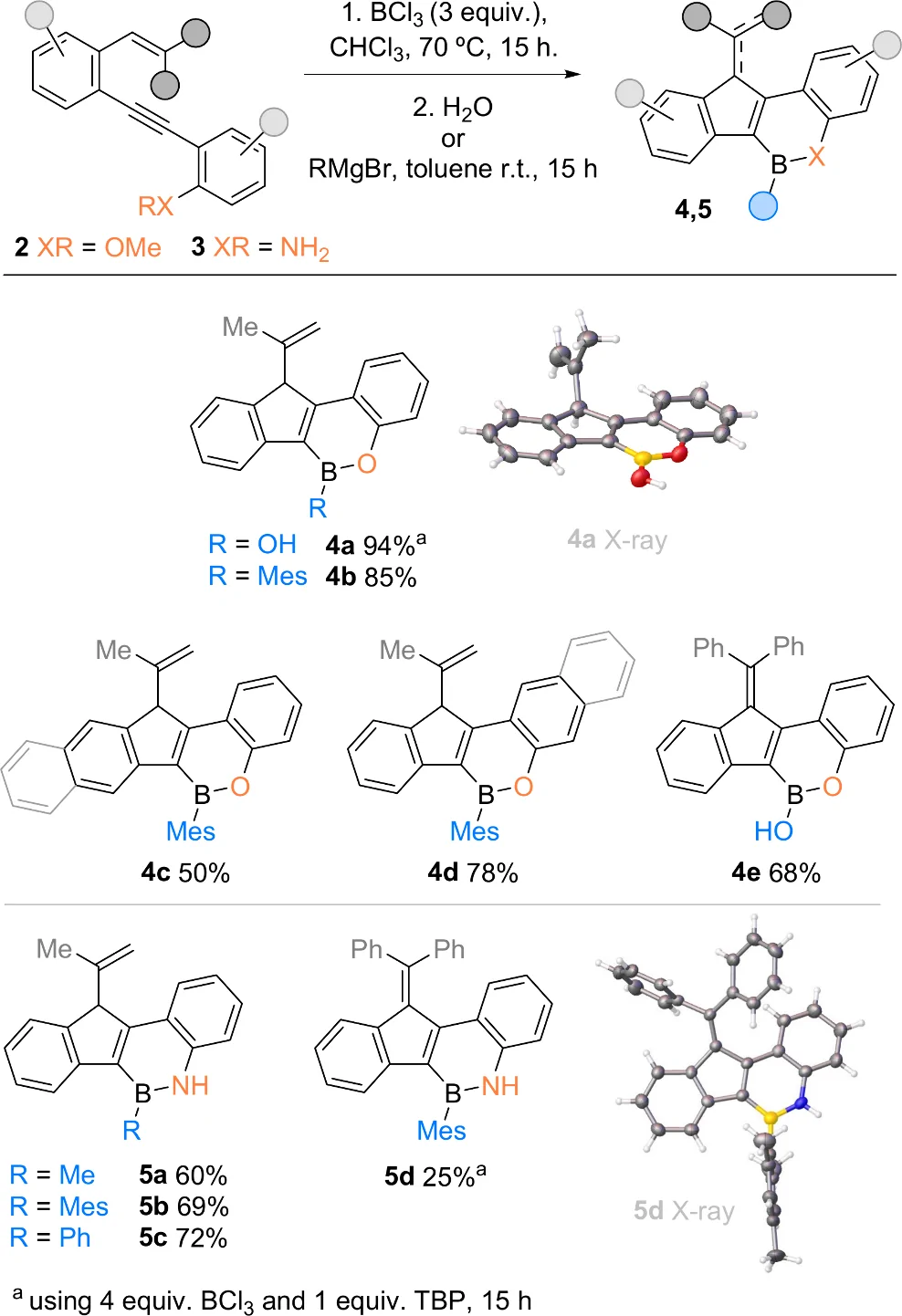
Scope of the Synthesis of B–X-Doped Benzo­[*a*]­fluorene Derivatives **4** and **5**

Polycycle **IVa**, obtained upon treatment of enyne **2a** with BCl_3_, contains a highly reactive B–Cl bond. This intermediate could be quenched either with water or with mesityl magnesium bromide, to provide the corresponding stable B–O-doped PAHs **4a** and **4b**, which were isolated in high yields.^20^ The methodology was also applicable to substrates leading to more extensively conjugated systems, as illustrated by the synthesis of derivatives **4c** and **4d**. Interestingly, enyne **2e**, bearing a β,β-diphenyl-substituted alkene, underwent an analogous cascade cyclization to furnish B–O-doped fulvene derivative **4e** in a good yield. In this case, intermediate **IIIe** undergoes HCl elimination with a different regiochemical outcome, as the elimination required to generate **IVe** is not possible due to the absence of a proton at the requisite position.

The strategy has been successfully extended to the preparation of B–N-doped PAHs. Thus, cyclization of amino-substituted enyne **3a** under the optimal conditions for the synthesis of B–O-doped PAHs efficiently provided target compounds **5a**–**5c** upon quenching with either alkyl or aryl Grignard reagents. In addition, B–N-doped fulvene derivative **5d** could be obtained from enyne **3d** bearing a β,β-diphenyl-substituted alkene, albeit in a modest yield. In this case, the addition of 2,4,6-tri-*tert*-butylpyridine (TBP) was required to minimize decomposition pathways.

Once the synthesis of B–X-doped benzo­[*a*]­fluorenes **4** and **5** had been successfully achieved, we turned our efforts to determine whether modifying the substitution pattern of the alkene moiety in the enyne precursor could change the selectivity of the cyclization and therefore provide access to a distinct family of B–X-doped PAHs containing a different scaffold (Scheme 3). To this end, we tested the cyclization of methoxy-substituted enyne **6a**, having an α-methyl-substituted alkene, in the presence of BCl_3_ at room temperature. As anticipated, the reaction proceeded selectively to furnish B–O-doped chrysene derivatives **8a**–**8c** after quenching with water, methanol, or mesitylmagnesium bromide. An enyne containing an α-phenyl-substituted alkene also proved to be an excellent substrate for this cascade transformation, affording the corresponding B–O-doped PAHs **8d** and **8e** in high yields. In contrast, synthesis of B–N-doped chrysenes proved more challenging, as significant decomposition was observed under the reaction conditions. Nevertheless, derivative **9a** could be isolated when the cyclization was carried out in the presence of TBP, although in a modest yield. The methodology proved also suitable for the synthesis of more complex B–O-doped PAHs, such as compounds **11** and **13**, which were readily obtained through double cascade cyclization of bisenynes **10** and **12**. The yields for these transformations are outstanding considering that each process involves the simultaneous formation of four new rings and six new covalent bonds, namely, two C–B, two C–C, and two B–O bonds.

**Scheme 3:**
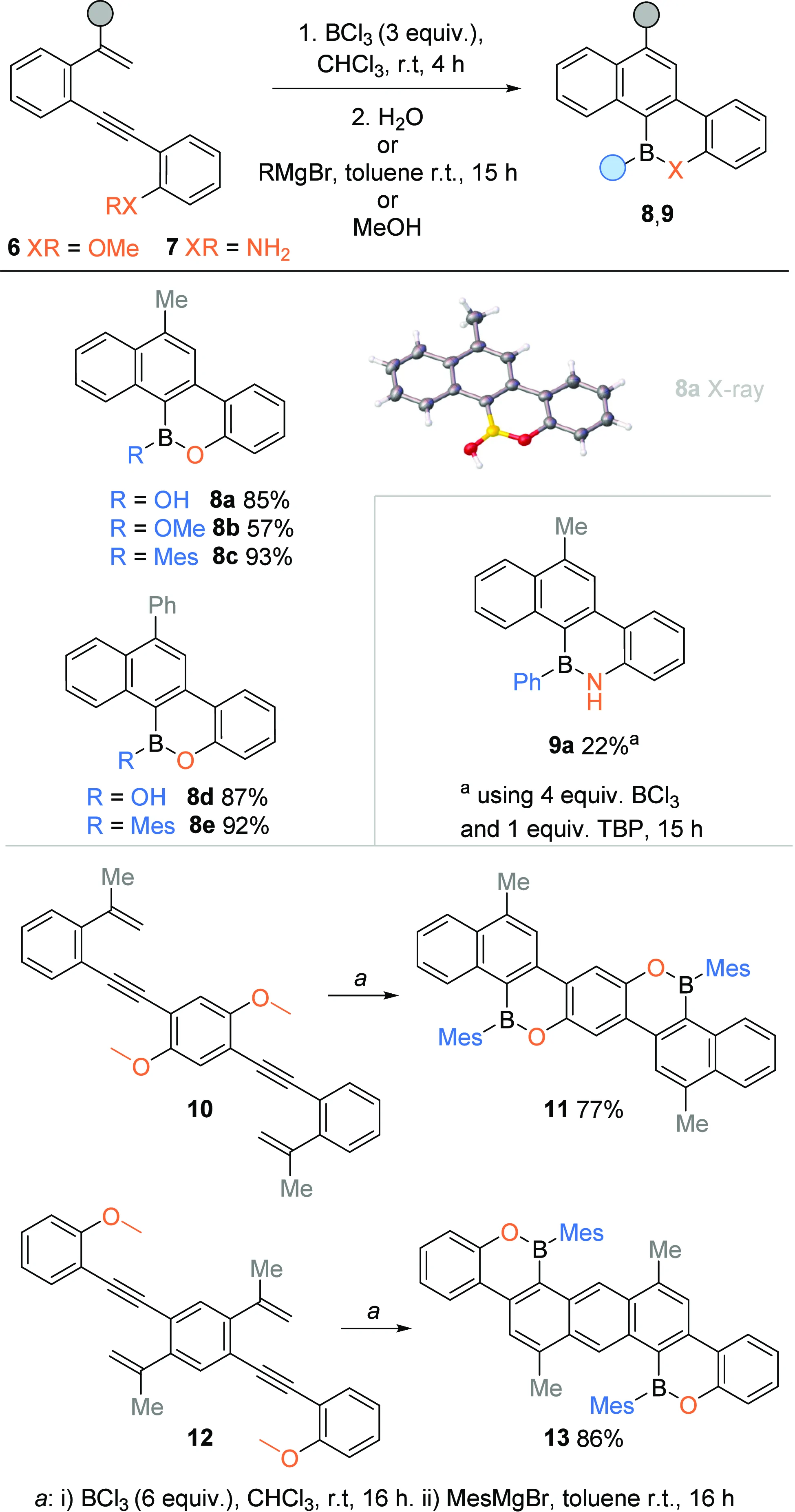
Scope of the Synthesis of B–X-Doped Chrysene Derivatives **8** and **9**

Remarkably, the scope of the methodology is not restricted to the synthesis of B–X-doped PAHs. Polycyclic compounds **15** and **17**, incorporating boron as the sole heteroatom, were also successfully prepared following the same cyclization cascade strategy, although more active BBr_3_ was necessary to promote the reaction (Scheme 4).^21^ In these substrates, the nucleophilic heteroatomic groups present in previous examples (e.g., OMe or NH_2_) were replaced by a naphthyl moiety capable of intramolecular attack onto the electrophilic boron center, thereby forming a new C–B bond and giving rise to the corresponding boron-doped polycyclic frameworks.^22^

**Scheme 4:**
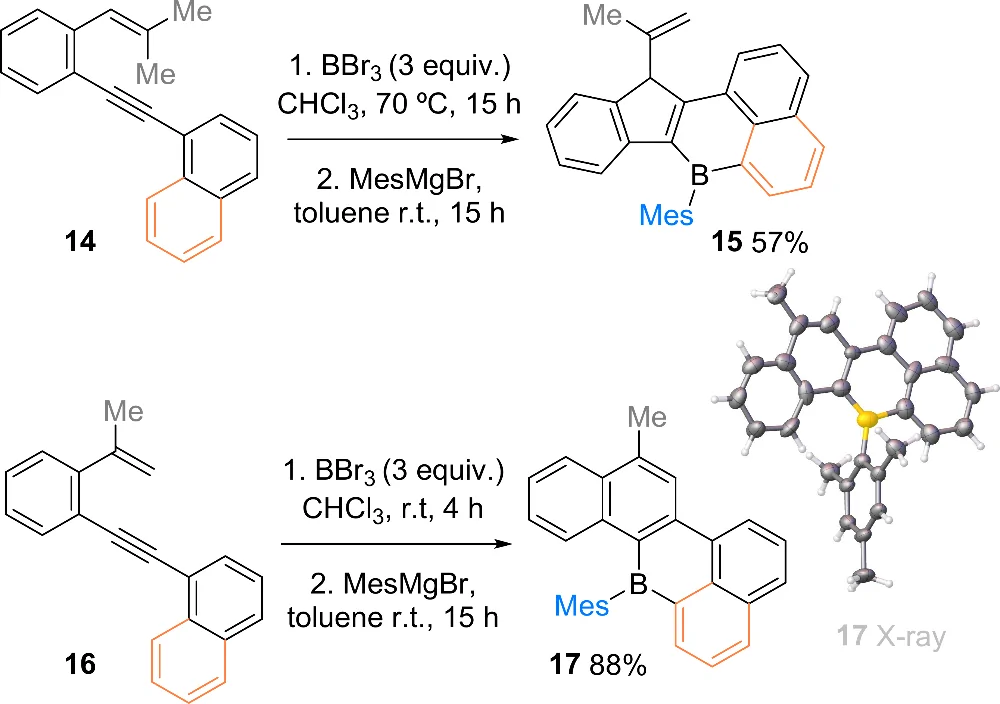
Synthesis of B-Doped PAHs **15** and **17**

The photophysical properties of the synthesized B-doped PAHs were evaluated to establish structure–property relationships. The corresponding absorption, excitation, and emission spectra are provided in Figures S6–S9 of the Supporting Information. Essential photophysical parameters, including absorption maxima, molar extinction coefficients, fluorescence quantum yields (ϕ_f_), lifetimes (τ), and radiative decay rates (*k*_r_), are detailed in Tables S1 and S2 (along with non-radiative constants, *k*_nr_, and radiative lifetimes, τ°), with a representative selection summarized in Table 1.

**Table 1:** Representative Photophysical and Radiative Decay Kinetic Data for Selected B-Doped PAHs in Dilute Solutions of Dichloromethane (DCM) at 25 °C

	λ_abs,max_ (nm)^a^	ε (M^–1^ cm^–1^)^b^	λ_em_ (nm)^c^	ϕ_f_ ^d^	τ (ns)^f^	*k*_r_ (10^7^, s^–1^)^h^
**4b**	333	19444 ± 110	385	0.91	3.0	30.3
**5b**	337	23520 ± 990	375	0.56	2.4	23.3
**5d**	383	9650 ± 30	462	0.01^e^	6.9^g^	0.1
**8c**	305	12075 ± 190	370	0.20	4.7	4.3
**11**	380	59930 ± 155	385	0.91	1.3	70.0
**13**	397	11290 ± 120	425	0.41^e^	3.4^g^	12.1
**17**	399	18780 ± 350	480	0.17^e^	11.4	1.5

a Lowest energy experimental absorption maximum λ_abs,max_.

b Molar extinction coefficients (ε) determined at λ_abs,max_.

c Emission maxima λ_em_.

d Fluorescence quantum yield ϕ_f_, determined using 9,10-diphenylanthracene (perylene).

e Singlet excited-state lifetimes (τ) recorded by time-correlated single-photon counting (TCSPC). Decay profiles were obtained by using a NanoLED (Horiba) emitting at 295 nm (370 nm).

f Radiative *k*_r_ (=ϕ_f_/τ) decay rate constant.

g In cyclohexane as a standard.^23^

h As an excitation source.

In the ground state, these organoborane heterocycles display intense, highly structured bands (300–360 nm), corresponding to symmetry-allowed π → π* transitions within a rigid polycyclic framework. In structurally extended derivatives, broader low-energy features (360–500 nm) emerge; these correspond to π → π* transitions acquiring significant intramolecular charge transfer (ICT) character due to effective resonance between the heteroatom lone pairs and the empty p_*z*_ orbital on boron.

Quantitatively, the molar extinction coefficients (ε) depend directly on the size and symmetry of the chromophore, while the five- and six-membered derivatives containing a single B–X exhibit values typically ranging between 0.5 × 10^4^ and 2.5 × 10^4^ M^–1^ cm^–1^; the dimeric architectures reach higher values (e.g., 6.0 × 10^4^ M^–1^ cm^–1^ for **11**) (Table 1 and Tables S1 and S2 of the Supporting Information). Beyond the simple heterocycles, structural expansion changes the optical properties in different ways. For single B–X analogues, incorporating fused aromatic rings (e.g., **4d**, **15**, and **17**) or appending an exocyclic diphenylmethylene group (**4e** and **5d**) causes a general bathochromic shift by extending covalent π conjugation. However, for **4e** and **5d**, this red shift comes with severe fluorescence quenching. In contrast, for compounds containing two B–O units (**11** and **13**), the optical properties change through space depending on the molecular shape. Coplanar dimer **11** yields a strong, red-shifted absorption band (380 nm). Conversely, the twisted structure of **13** distorts these spatial interactions, shifting its most intense absorption band to the UV region (325 nm) while rendering its lowest energy transition weak at ∼397 nm.

Upon photoexcitation, ϕ_f_ values are dictated by structural rigidity, which prevents non-radiative losses (Table 1). Rigid five-membered oxaborine **4b** and coplanar dimer **11** exhibit maximum efficiency (ϕ_f_ = 0.91) because their rigid polycyclic frameworks effectively diminish non-radiative decay while maintaining remarkably fast radiative rates. Conversely, introducing vibrational or conformational freedom systematically penalizes emission. For instance, core-localized N–H vibrations in azaborine **5b** introduce efficient non-radiative deactivation channels, lowering ϕ_f_ to 0.56 relative to its oxaborine analogue (**4b**). More severely, expanding the central ring from five- to six-membered cores (such as **8c** and **17**) generally increases non-radiative pathways across the series, collapsing the emission efficiency to or below 0.20. Finally, appending a flexible diphenylmethylene moiety (**4e** and **5d**) activates molecular rotors, completely draining the singlet excited-state energy through internal conversion and triggering a total collapse of fluorescence.

Mechanistically, all emissive derivatives displayed monoexponential fluorescence decays, confirming deactivation from a single homogeneous excited-state population. Furthermore, excitation spectra (Figures S7 and S9) superimpose with their ground-state absorption profiles (Figures S6 and S8), demonstrating that fluorescence originates strictly from the main species and ruling out interference from fluorescent impurities, emissive aggregates in solution, or newly formed excited-state species.

Given the library’s extensive scope, the mechanical impacts and photokinetic profiles governing each structural trend, covering core heteroatoms, fusion topologies, boron substituents, exocyclic rotors, ring expansion, and positional regioisomerism of coupled oxaborines, are comprehensively detailed on pages SI 19–SI 28 of the Supporting Information.

In conclusion, we have developed a modular, metal-free cascade strategy for the synthesis of structurally diverse boron-doped polycyclic aromatic hydrocarbons from readily accessible enynes using BCl_3_ (or BBr_3_) as the sole promoter. The methodology simultaneously constructs the polycyclic framework and B–O, B–N, or C–B bonds in a single operation, while the boron substituent can be readily diversified through the quenching step. The substitution pattern of the alkene dictates the cyclization pathway, providing selective access to either five- or six-membered rings and, consequently, to distinct PAH scaffolds. Finally, photophysical studies established clear structure–property relationships, demonstrating how the ring size, heteroatom identity, π conjugation, and molecular rigidity govern the optical and emission properties of these boron-containing materials. This strategy provides a versatile platform for the rapid synthesis and systematic tuning of boron-doped π-conjugated systems with potential applications in functional materials.

## Supplementary Material



## Data Availability

The data underlying this study are available in the published article and its Supporting Information.

## Notes

Experimental procedures, characterization data, comprehensive structural and photokinetic analyses, X-ray crystallographic data for **4a**, **5d**, **8a**, and **17**, and NMR spectra for new compounds (PDF)
