# Supplementary material for: Cascade Synthesis of Structurally Diverse B–X-Doped Polycyclic Aromatic Hydrocarbons
Source: Org Lett. 2026 Sep 2;28(37):11631–6. doi: 10.1021/acs.orglett.6c03304 (PMC13595909; doi:10.1021/acs.orglett.6c03304)
Supplement: Supplementary file 1 [file ol6c03304_si_001.pdf]

Supporting Information

Cascade synthesis of structurally diverse B–X-doped polycyclic aromatic hydrocarbons

Marcos Humanes,[a] Jaime Mateos-Gil,[a] Francisco Mendicuti,[b] Manuel A. Fernández-Rodríguez,[a]* Patricia García-García[a]*

a Universidad de Alcalá (IRYCIS). Departamento de Química Orgánica y Química Inorgánica, Instituto de Investigación Química "Andrés M. del Río" (IQAR), 28805 Alcalá de Henares, Madrid, Spain.

b Universidad de Alcalá, Departamento de Química Analítica, Química Física e Ingeniería Química, Instituto de Investigación Química "Andrés M. del Río" (IQAR). 28805 Alcalá de Henares, Madrid, Spain.

Table of Contents

General Experimental Details --SI 2

Experimental Procedures and Characterization Data--------------------------------------SI 3

Initial NMR-studies of the cyclization of **1a** and **2a** in the presence of BCl_3 ---------SI 3

Synthesis and characterization data of starting enynes and their corresponding precursors---SI 5

General procedures for the synthesis of B-doped PAHs ------------------------------SI 11

Characterization data of B-doped PAHs --SI 12

Comprehensive Structural and Photokinetic Analysis-----------------------------------SI 19

1. Influence of the Core Heteroatom (N vs. O) ---SI 19

2. Position-dependent π-extension---SI 19

3. Influence of the Boron Substituent ---SI 20

4. Influence of the Exocyclic Diphenylmethylene Molecular Rotor------------------SI 21

5. Expansion of the Core Heterocycle (5- vs. 6-Membered Rings) -------------------SI 21

6. Interplay of Core Size (5- vs. 6-Membered Rings), Boron Substitution, and Peripheral Conjugation in Oxaborines ---SI 22

7. From Single B–O to Double B–O-containing Systems: Positional Isomerism in Coupled Oxaborines ---SI 23

8. Photophysical Behavior of Oxygen-Free Expanded Analogues (15 and 17) -----SI 24

X-ray diffraction data for **4a**, **5d**, **8a** and **17** ---SI 29

Copies of ^1H, ^{13}C, ^{19}F and ^{11}B NMR spectra for novel compounds -------------------SI 36

General Experimental Details

All reactions involving air or moisture sensitive compounds were carried out under inert atmosphere (Ar). Glassware was dried in a hot oven overnight. All reactions that require heating were performed using a sand bath. Anhydrous triethylamine and deuterated chloroform were dried over calcium hydride, distilled under vacuum and stored over molecular sieves (3 Å) under inert atmosphere. Temperatures are reported as sand-bath temperatures. Dry solvents, where necessary, were purchased directly from Fisher Scientific and stored over molecular sieves (3 Å) under inert atmosphere. Starting materials sourced from commercial suppliers were used as received unless otherwise stated. Reactions were monitored using analytical TLC plates (Merck; silica gel 60 F254, 0.25 mm), and compounds were visualized with UV radiation. Silica gel grade 60 (70-230 mesh, Silicycle) was used for column chromatography. All melting points were determined in open capillary tubes on a Stuart Scientific SMP3 melting point apparatus (uncorrected). ^{1}H, ^{13}C and ^{11}B NMR spectra were recorded on either Varian Mercury VX-300, Varian Unity 300, Bruker Avance Neo 400 or Varian Unity 500 MHz spectrometer at room temperature. Low temperature NMR experiments were carried out on a Varian Unity 500 MHz spectrometer at the indicated temperature. Chemical shifts are given in ppm (δ) downfield from tetramethylsilane, with calibration on the residual protio-solvent used (δ_H = 7.26 ppm and δ_C = 77.2 ppm for $CDCl_3$). Coupling constants (*J*) are in Hertz (Hz) and signals are described as follows: s, singlet; bs, broad singlet; d, doublet; t, triplet; p, pentuplet; dd, double doublet; dt, doublet of triplets; dq, doublet of quartets; td, triplet of doublets; ddd, double doublet of doublets; and m, multiplet. Resonances for the carbon atoms directly bonded to boron are not observed in the $^{13}C\{^{1}H\}$ NMR spectra due to quadrupolar relaxation effects. High-resolution mass spectroscopy analyses (HRMS) were performed on an Agilent 6210 time of-flight LC/MS.

Experimental Procedures and Characterization Data

Initial NMR-studies of the cyclization of 1a and 2a in the presence of BCl_3:

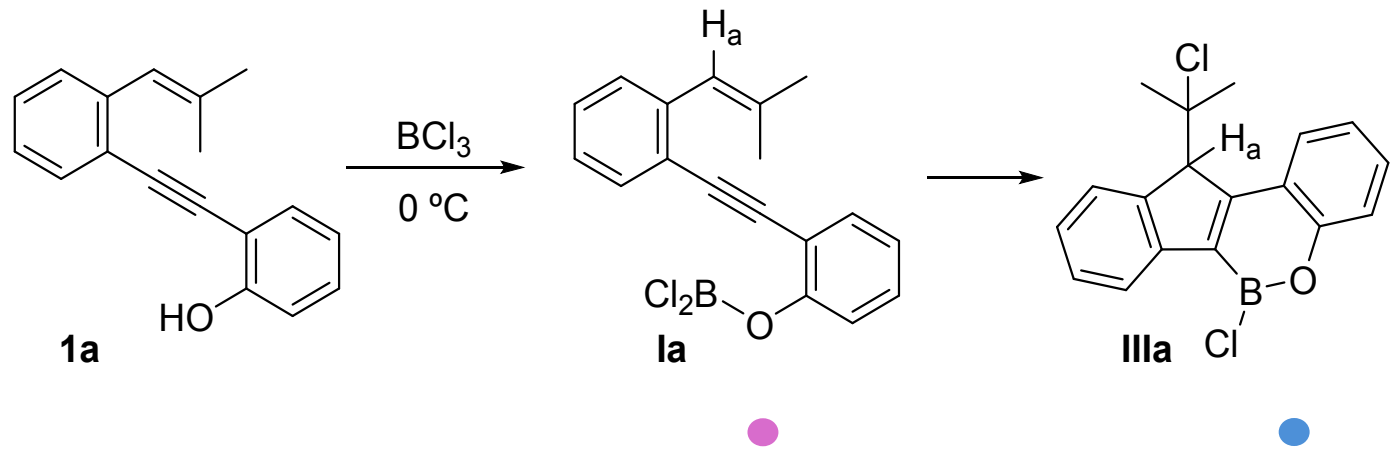

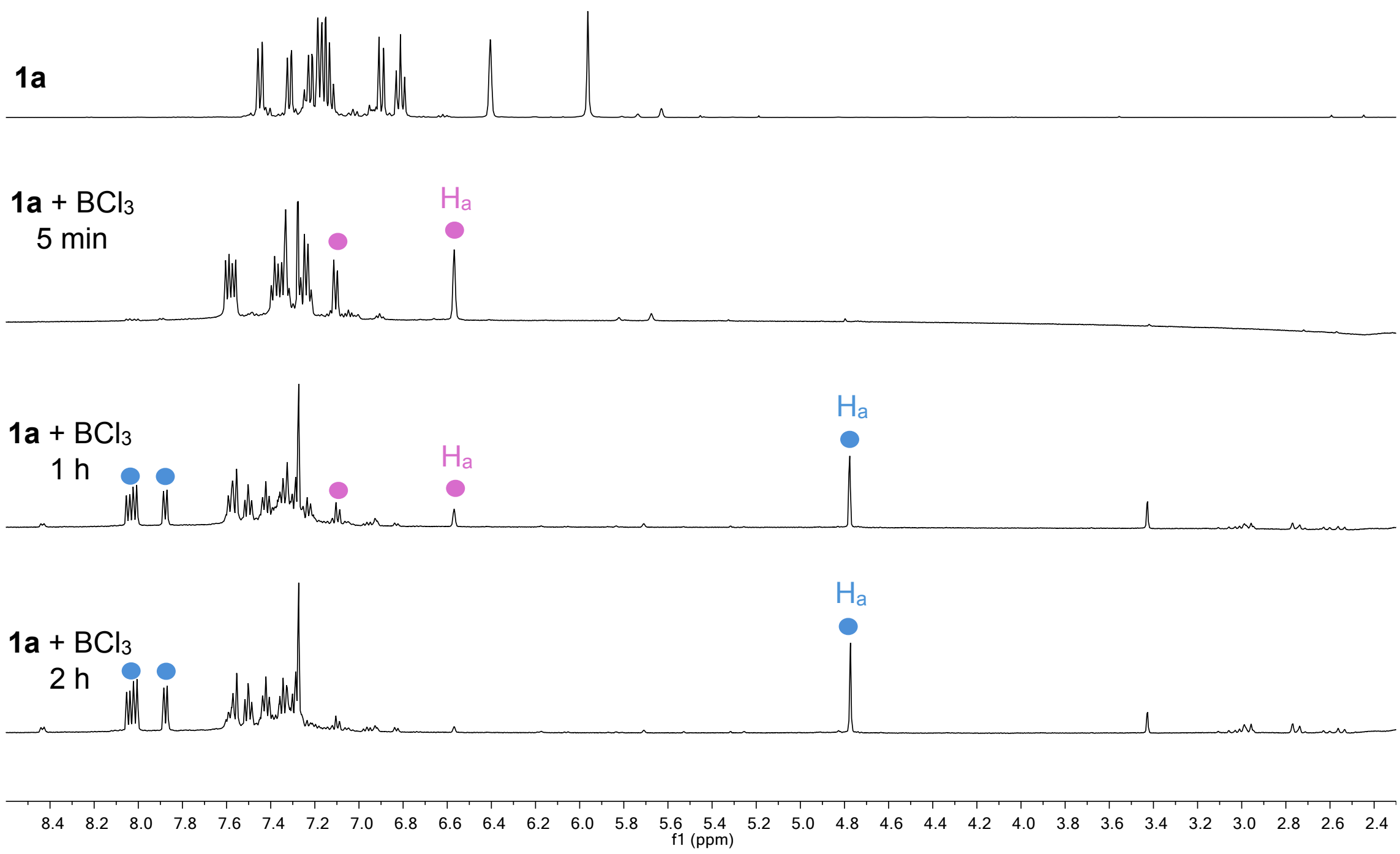

Figure S1. ^{1}H-NMR spectra of the reaction of **1a** with 3 equivalents of BCl_3 in $CDCl_3$ at 0 ºC.

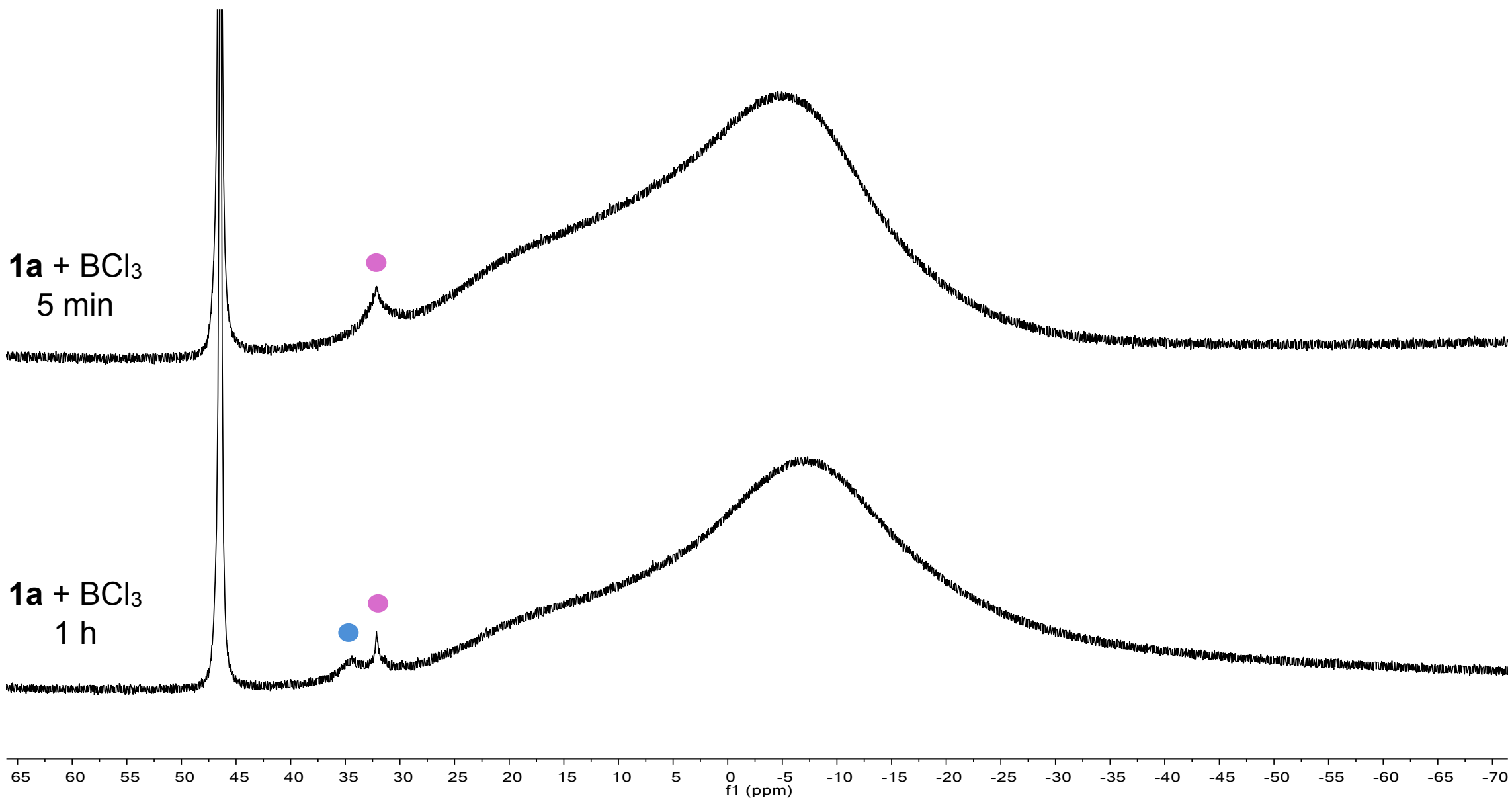

Figure S2. ^{11}B-NMR spectra of the reaction of **1a** with 3 equivalents of BCl_3 in $CDCl_3$ at 0 ºC.

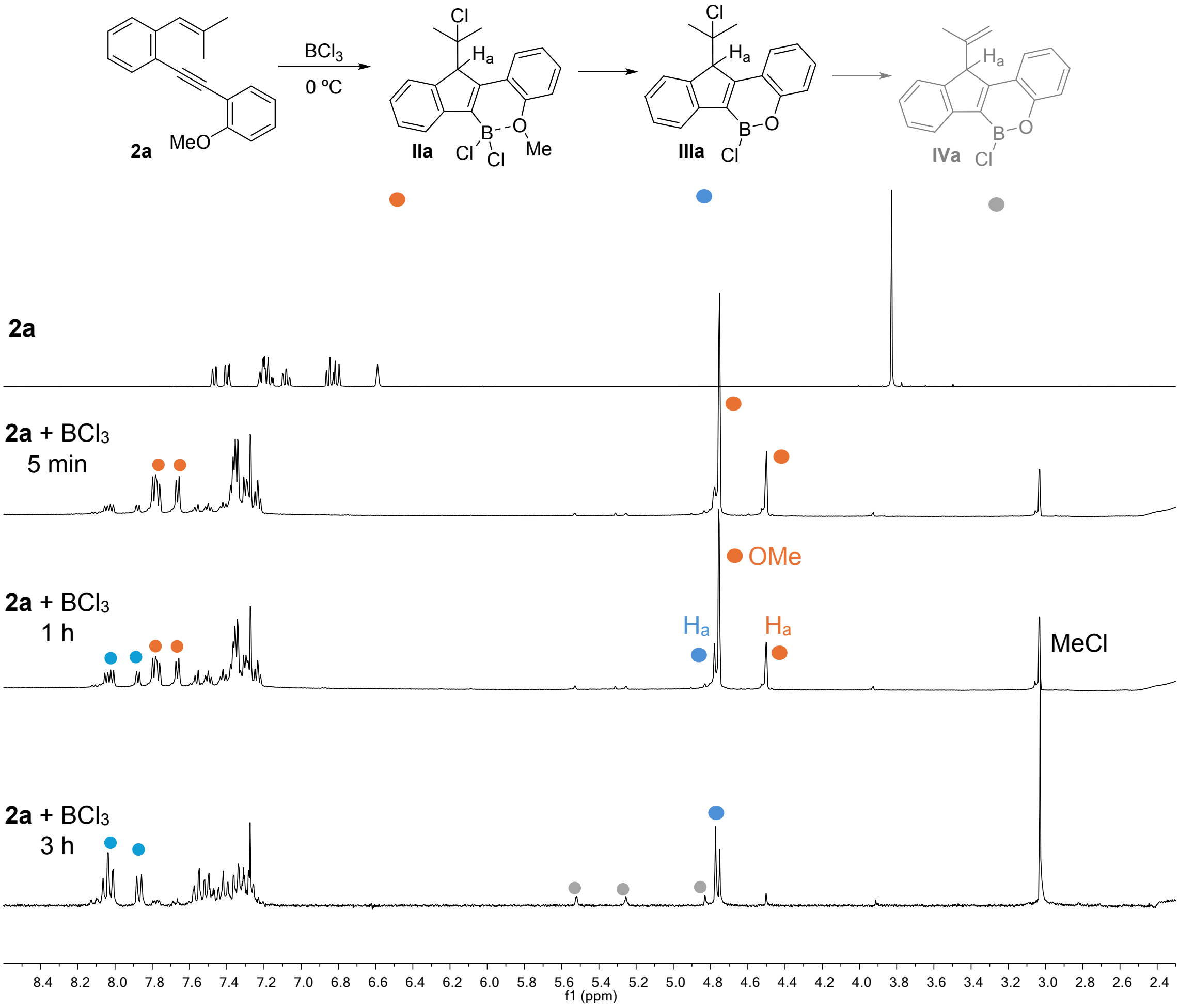

Figure S3. ^{1}H-NMR spectra of the reaction of **2a** with 3 equivalents of BCl_3 in $CDCl_3$ at 0 ºC.

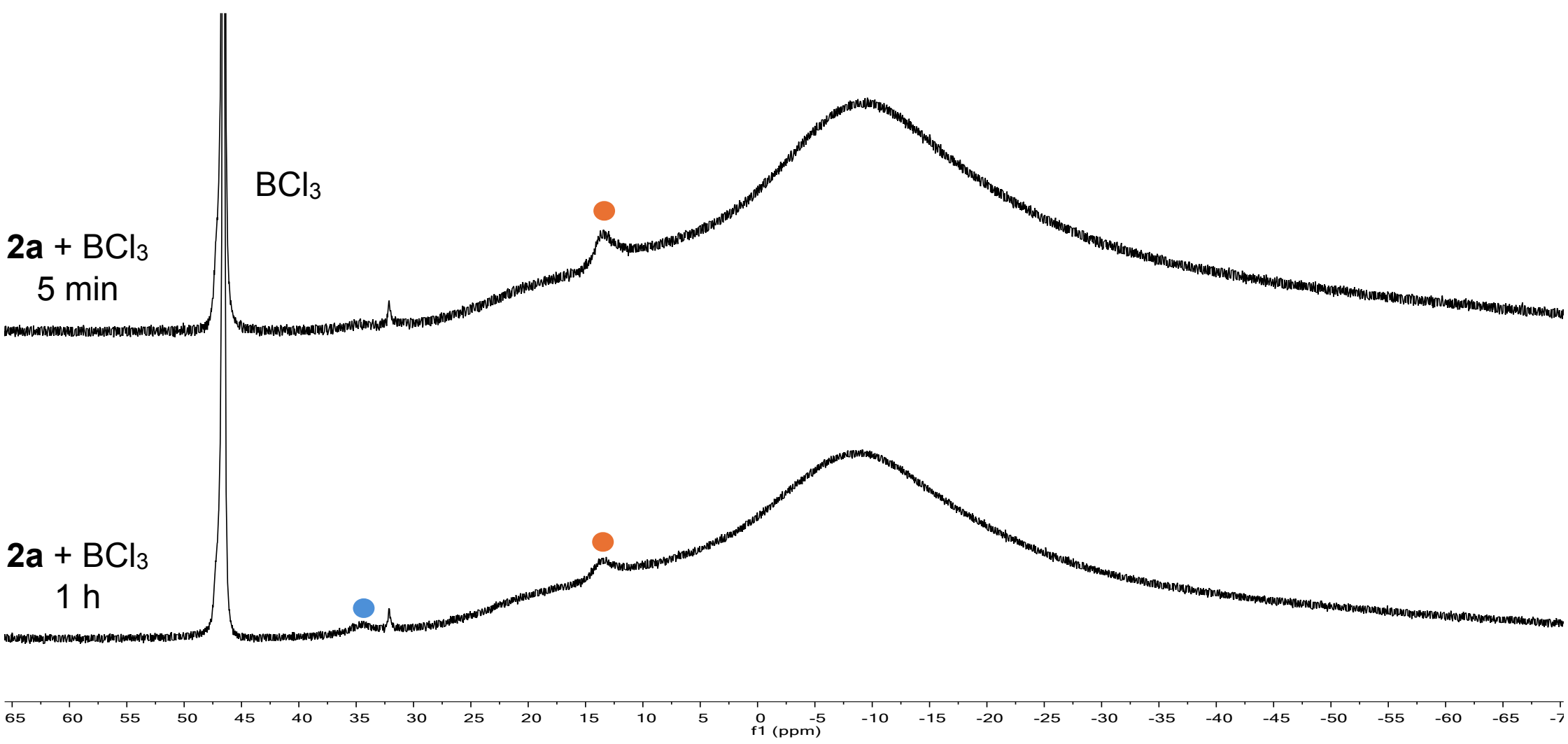

Figure S4. ^{1}H-NMR spectra of the reaction of **2a** with 3 equivalents of BCl_3 in $CDCl_3$ at 0 ºC.

Synthesis and characterization data of starting enynes and their corresponding precursors:

1a,[1] **2a**,[2] **7a**,[3] and **16**[4] had been previously described and their spectroscopic data match those previously reported. The rest of the starting materials were prepared as follows:

Method A:

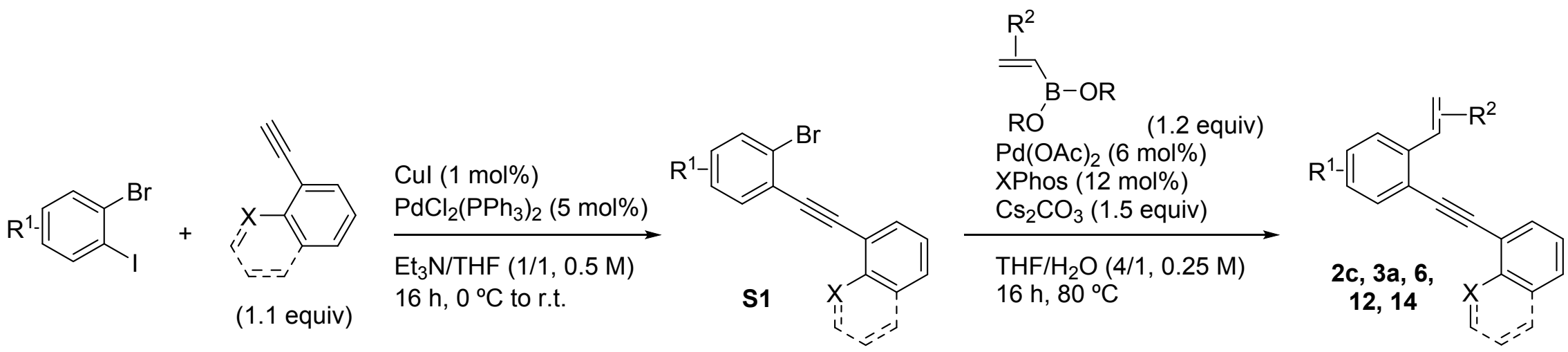

Step 1: In a sealable Schlenk flask, CuI (1 mol%), $PdCl_2(PPh_3)_2$ (5 mol%) and the corresponding *o*-substituted iodoarene (if solid) were added and an Ar atmosphere was established. The reagents were suspended in a mixture of anhydrous Et_3N and THF (1:1, 0.5 M) and cooled in an ice-water bath. Then, the appropriate *o*-substituted iodoarene derivative (if liquid) (1 equiv) and the corresponding acetylene (1.1 equiv) were sequentially added, and the Schlenk flask was sealed. After 30 minutes, the flask was allowed to warm to room temperature. The mixture was stirred until completion of the reaction as monitored by TLC (16-24 h). The crude was extracted with DCM and H_2O and the combined organic layers were dried over anhydrous Na_2SO_4, filtered and evaporated. The residue was purified by flash chromatography on silica gel using mixtures of hexane and EtOAc as eluents to obtain the desired *o*-substituted alkynes **S1**. **S1a**,[5] **S1b**,[6] **S1c**,[7] and **S1e**[8] (precursors of **2c**, **3a**, **6a-b** and **14**) had been previously described and their spectroscopic data match those previously reported.

2,2'-((2,5-Dibromo-1,4-phenylene)bis(ethyne-2,1-diyl))bis(methoxybenzene) (S1d)

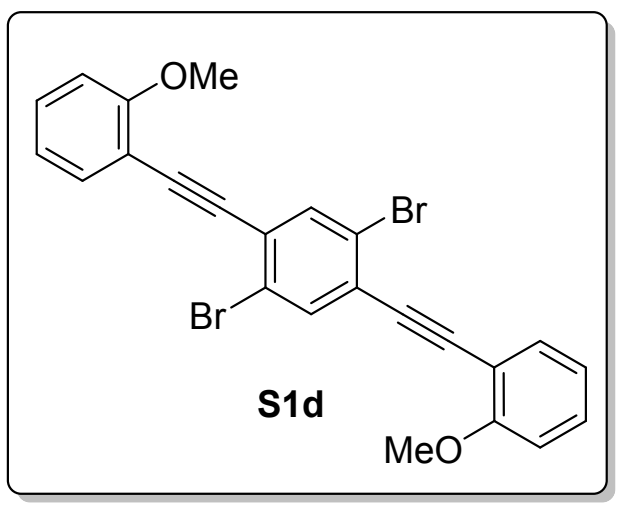

Obtained as yellow solid (367.0 mg, 0.739 mmol, 37%) from 1,4-dibromo-2,5-diiodobenzene (974 mg, 2.0 mmol) using doubled quantities respect to the general method for the rest of the reagents and solvents to account for the second coupling. R_f = 0.50 (Hexane/DCM 1/1). m.p. = 180–181 ºC.

^{1}H NMR (400 MHz, $CDCl_3$) δ 7.83 (s, 2H), 7.56 (dd, *J* = 7.6, 1.7 Hz, 2H), 7.38 (ddd, *J* = 8.4, 7.4, 1.8 Hz, 2H), 6.99 (td, *J* = 7.5, 1.0 Hz, 2H), 6.95 (d, *J* = 8.4 Hz, 2H), 3.96 (s, 6H).

---

1 A. M. Sanjuán, M. A. Rashid, P. García-García, A. Martínez-Cuezva, M. A. Fernández-Rodríguez, F. Rodríguez, R. Sanz *Chem. Eur. J.* **2015**, *21*, 3042–3052.
2 C. Grandclaudon, M. Ruamps, R. Duboc, V. Michelet, P. Y. Toullec *Synlett* **2018**, *29*, 1181–1186.
3 J. Fu, B. Li, X. Wang, Q. Liang, X. Peng, L. Yang, T. Wan, X. Wang, B. Lin, M. Cheng, Y. Liu *Chin. J. Chem.* **2022**, *40*, 46–52.
4 M. Humanes, M. A. Fernández-Rodríguez, P. García-García *Adv. Synth. Catal.* **2025**, *367*, e202500244.
5 T. Morishita, H. Yoshida, J. Ohshita *Chem. Commun.* **2010**, *46*, 640–642.
6 S. K. Banjare, L. Lezius, E. S. Horst, D. Leifert, C. G. Daniliuc, F. A. Alasmary, A. Studer *Angew. Chem. Int. Ed.* **2024**, *63*, e202404275.
7 M. Kuhn, F. C. Falk, J. Paradies *Org. Lett.* **2011**, *13*, 4100–4103.
8 K. Hirano, Y. Inaba, N. Takahashi, M. Shimano, S. Oishi, N. Fujii, H. Ohno *J. Org. Chem.* **2011**, *76*, 1212–1227.

^{13}C NMR (101 MHz, CDCl$_3$) δ 160.4 (2xC), 136.2 (2xCH), 133.9 (2xCH), 130.8 (2xCH), 126.7 (2xC), 123.7 (2xC), 120.7 (2xCH), 111.8 (2xC), 111.0 (2xCH), 93.4 (2xC), 91.0 (2xC), 56.1 (2xCH$_3$).

HRMS (EI+) m/z: $[M]^+$ Calcd for $[C_{24}H_{16}Br_2O_2]^+$ 493.9511. Found 493.9508.

Step 2: In a sealable Schlenk flask, $Pd(OAc)_2$ (6 mol%), XPhos (12 mol%) and Cs_2CO_3 (1.5 equiv) were added, and an Ar atmosphere was established. The catalysts were suspended in a degassed mixture of THF/H_2O (4/1, 0.25 M) and the appropriate *o*-(bromo)alkyne **S1** (1 equiv) and boronic pinacol ester (1.2 equiv) were sequentially added. The flask was sealed and the mixture was heated to 80 ºC and stirred until completion of the reaction as monitored by TLC (16-24 h). The crude was extracted with Et_2O and H_2O and the combined organic layers were dried over anhydrous Na_2SO_4, filtered and evaporated. The residue was purified by flash chromatography on silica gel using mixtures of hexane and EtOAc as eluents to obtain the desired enynes.

2-((2-Methoxyphenyl)ethynyl)-3-(2-methylprop-1-en-1-yl)naphthalene (2c)

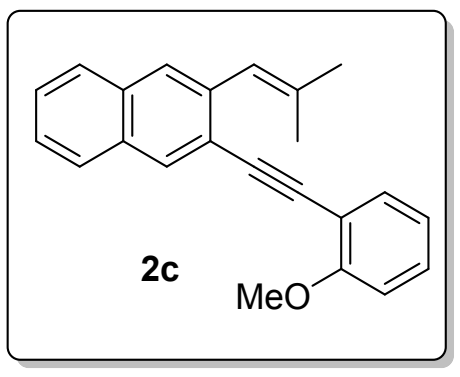

Obtained as yellow oil (134.0 mg, 0.428 mmol, 58%) from 2-bromo-3-((2-methoxyphenyl)ethynyl)naphthalene **S1a** (249 mg, 0,740 mmol). R_f = 0.40 (Hexane/AcOEt 95/5).

^{1}H NMR (400 MHz, CDCl$_3$) δ 8.09 (s, 1H), 7.81 – 7.74 (m, 2H), 7.70 (s, 1H), 7.54 (dd, *J* = 7.6, 1.8 Hz, 1H), 7.49 – 7.40 (m, 2H), 7.32 (ddd, *J* = 8.3, 7.4, 1.8 Hz, 1H), 6.97 (td, *J* = 7.5, 1.1 Hz, 1H), 6.93 (dd, *J* = 8.4, 1.0 Hz, 1H), 6.83 – 6.77 (m, 1H), 3.96 (s, 3H), 2.05 (d, *J* = 1.5 Hz, 3H), 1.95 (d, *J* = 1.4 Hz, 3H).

^{13}C NMR (101 MHz, CDCl$_3$) δ 160.0 (C), 136.9 (C), 136.6 (C), 133.4 (CH), 132.8 (C), 131.7 (CH), 131.5 (C), 129.7 (CH), 127.6 (CH), 127.5 (CH), 127.4 (CH), 126.5 (CH), 125.8 (CH), 124.1 (CH), 121.7 (C), 120.5 (CH), 112.9 (C), 110.7 (CH), 93.0 (C), 89.7 (C), 55.9 (CH$_3$), 26.9 (CH$_3$), 19.7 (CH$_3$)

HRMS (EI+) m/z: $[M]^+$ Calcd for $[C_{23}H_{20}O]^+$ 312.1509. Found 312.1509.

2-((2-(2-Methylprop-1-en-1-yl)phenyl)ethynyl)aniline (3a)

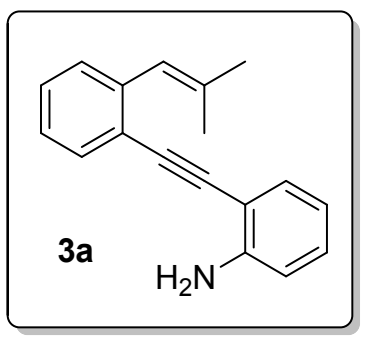

Obtained as yellow oil (399.5 mg, 1.62 mmol, 81%) from 2-((2-bromophenyl)ethynyl)aniline **S1b** (544 mg, 2.0 mmol). R_f = 0.60 (Hexane/AcOEt 9/1).

^{1}H NMR (400 MHz, CDCl$_3$) δ 7.43 (dt, *J* = 7.6, 1.1 Hz, 1H), 7.27 (dd, *J* = 7.9, 1.6 Hz, 1H), 7.20 – 7.15 (m, 2H), 7.14 – 7.07 (m, 1H), 7.03 (ddd, *J* = 8.2, 7.3, 1.6 Hz, 1H), 6.65 – 6.57 (m, 2H), 6.47 – 6.42 (m, 1H), 4.23 (s, 2H), 1.85 (d, *J* = 1.7 Hz, 3H), 1.72 (d, *J* = 1.5 Hz, 3H).

^{13}C NMR (101 MHz, CDCl$_3$) δ 148.0 (C), 140.4 (C), 137.0 (C), 131.9 (CH), 131.7 (CH), 129.7 (CH), 129.3 (CH), 127.8 (CH), 126.2 (CH), 124.4 (CH), 122.9 (C), 117.9 (CH), 114.3 (CH), 108.3 (C), 94.3 (C), 90.2 (C), 26.5 (CH$_3$), 19.6 (CH$_3$).

HRMS (ESI-TOF) m/z: $[M+H]^+$ Calcd for $[C_{18}H_{18}N]^+$ 248.1434. Found 248.1432.

2-((2-(Prop-1-en-2-yl)phenyl)ethynyl)anisole (6a)

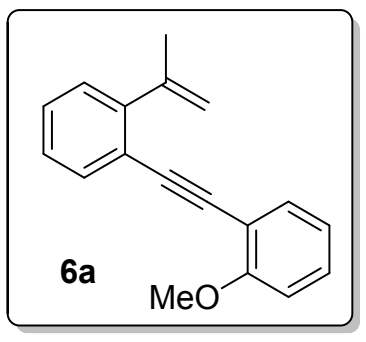

Obtained as yellow oil (355.0 mg, 1.43 mmol, 72%) from 1-bromo-2-((2-methoxyphenyl)ethynyl)benzene **S1c** (574 mg, 2.0 mmol). R_f = 0.50 (Hexane/AcOEt 97/3).

^{1}H NMR (400 MHz, CDCl$_3$) δ 7.60 – 7.54 (m, 1H), 7.46 (dd, *J* = 7.6, 1.7 Hz, 1H), 7.34 – 7.19 (m, 4H), 6.93 (td, *J* = 7.5, 1.1 Hz, 1H), 6.89 (dd, *J* = 8.3, 1.0 Hz, 1H), 5.26 (p, *J* = 1.6 Hz, 1H), 5.23 (dq, *J* = 1.7, 0.9 Hz, 1H), 3.90 (s, 3H), 2.27 (dd, *J* = 1.5, 0.9 Hz, 3H).

^{13}C NMR (101 MHz, CDCl$_3$) δ 160.1 (C), 145.9 (C), 145.1 (C), 133.3 (CH), 132.9 (CH), 129.6 (CH), 128.1 (CH), 127.9 (CH), 126.7 (CH), 121.1 (C), 120.5 (CH), 115.7 (CH), 112.9 (C), 110.8 (CH_2), 93.1 (C), 89.3 (C), 55.8 (CH_3), 23.3 (CH_3).

HRMS (ESI-TOF) m/z: $[M+H]^+$ Calcd for $[C_{18}H_{17}O]^+$ 249.1274. Found 249.1275.

2-((2-(1-Phenylvinyl)phenyl)ethynyl)anisole (6b)

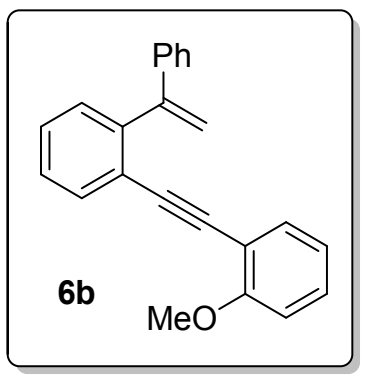

Obtained as pale yellow solid (598.0 mg, 1.92 mmol, 96%) from 1-bromo-2-((2-methoxyphenyl)ethynyl)benzene **S1c** (574 mg, 2.0 mmol). R_f = 0.37 (Hexane/AcOEt 97/3). m.p. = 88–89 ºC.

^{1}H NMR (400 MHz, CDCl$_3$) δ 7.73 – 7.63 (m, 1H), 7.44 – 7.30 (m, 8H), 7.29 – 7.23 (m, 1H), 7.04 (dt, *J* = 7.5, 2.0 Hz, 1H), 6.90 – 6.82 (m, 2H), 5.88 – 5.83 (m, 1H), 5.56 – 5.51 (m, 1H), 3.86 (s, 3H).

^{13}C NMR (101 MHz, CDCl$_3$) δ 159.9 (C), 148.8 (C), 144.2 (C), 141.2 (C), 133.7 (CH), 132.9 (CH), 130.1 (CH), 129.6 (CH), 128.3 (2xCH), 128.1 (CH), 127.5 (CH), 127.4 (CH), 127.3 (2xCH), 123.0 (C), 120.3 (CH), 116.5 (CH), 112.8 (C), 110.7 (CH_2), 92.9 (C), 89.9 (C), 55.8 (CH_3).

HRMS (ESI-TOF) m/z: $[M+H]^+$ Calcd for $[C_{23}H_{19}O]^+$ 311.1430. Found 311.1428.

2,2'-((2,5-Di(prop-1-en-2-yl)-1,4-phenylene)bis(ethyne-2,1-diyl))bis (methoxybenzene) (12)

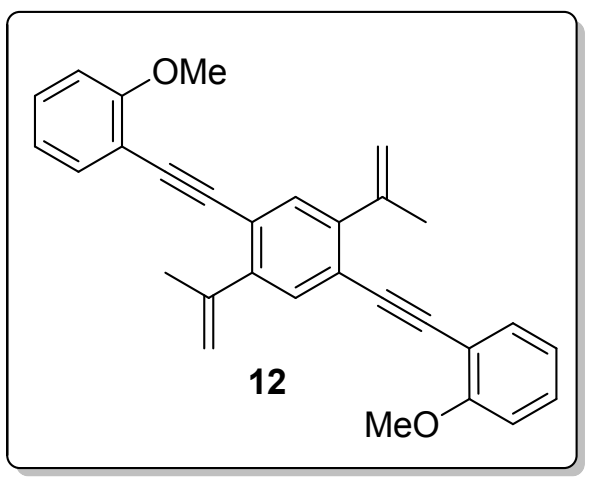

Obtained as yellow solid (570.0 mg, 0.619 mmol, 61%) from **S1d** (494 mg, 1.0 mmol) using doubled quantities respect to the general method for the rest of the reagents and solvents to account for the second coupling. R_f = 0.41 (Hexane/AcOEt 94/6). m.p. = 156–157 ºC.

^{1}H NMR (400 MHz, CDCl$_3$) δ 7.49 (s, 1H), 7.47 (dd, *J* = 7.6, 1.7 Hz, 1H), 7.31 (ddd, *J* = 8.3, 7.4, 1.7 Hz, 1H), 6.94 (td, *J* = 7.5, 1.1 Hz, 1H), 6.90 (dd, *J* = 8.4, 1.0 Hz, 1H), 5.31 (dq, *J* = 1.7, 0.9 Hz, 1H), 5.29 (p, *J* = 1.6 Hz, 1H), 3.91 (s, 3H), 2.29 (dd, *J* = 1.6, 0.9 Hz, 3H).

^{13}C NMR (101 MHz, CDCl$_3$) δ 160.2 (2xC), 144.17 (2xC), 144.16 (2xC), 133.5 (2xCH), 132.4 (2xCH), 129.94 (2xCH), 121.0 (2xC), 120.6 (2xCH), 116.2 (2xCH_2), 112.8 (2xC), 110.8 (2xCH), 93.2 (2xC), 90.8 (2xC), 55.9 (2xCH_3), 23.3 (2xCH_3).

HRMS (EI+) m/z: $[M]^+$ Calcd for $[C_{30}H_{26}O_2]^+$ 418.1927. Found 418.1925.

1-((2-(2-Methylprop-1-en-1-yl)phenyl)ethynyl)naphthalene (14)

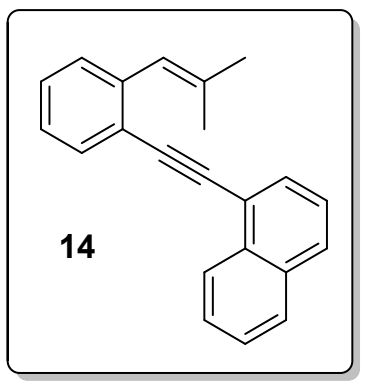

Obtained as yellow oil (508.3 mg, 1.80 mmol, 90%) from 1-((2-bromophenyl)ethynyl)naphthalene **S1e** (614 mg, 2.0 mmol). R_f = 0.33 (Hexane).

^{1}H NMR (400 MHz, CDCl$_3$) δ 8.40 (d, *J* = 8.2 Hz, 1H), 7.76 (d, *J* = 7.8 Hz, 1H), 7.72 (d, *J* = 8.3 Hz, 1H), 7.66 (dd, *J* = 7.2, 1.7 Hz, 1H), 7.55 (d, *J* = 7.5 Hz, 1H), 7.48 (ddd, *J* = 8.3, 6.8, 1.5 Hz, 1H), 7.42 (ddd, *J* = 8.2, 6.8, 1.4 Hz, 1H), 7.35 (dd, *J* = 8.3, 7.1 Hz, 1H), 7.26 – 7.18 (m, 2H), 7.17 – 7.10 (m, 1H), 6.68 – 6.60 (m, 1H), 1.95 (d, *J* = 1.6 Hz, 3H), 1.77 (d, *J* = 1.5 Hz, 3H).

^{13}C NMR (101 MHz, CDCl$_3$) δ 140.9 (C), 136.9 (C), 133.5 (C), 133.4 (C), 132.2 (CH), 130.3 (CH), 129.4 (CH), 128.7 (CH), 128.4 (CH), 128.1 (CH), 126.9 (CH), 126.6 (CH), 126.5 (CH), 126.2 (CH), 125.5 (CH), 124.5 (CH), 122.9 (C), 121.5 (C), 93.9 (C), 91.8 (C), 26.8 (CH$_3$), 19.7 (CH$_3$).

HRMS (EI+) m/z: $[M]^+$ Calcd for $[C_{22}H_{18}]^+$ 282.1403. Found 282.1401.

Method B:

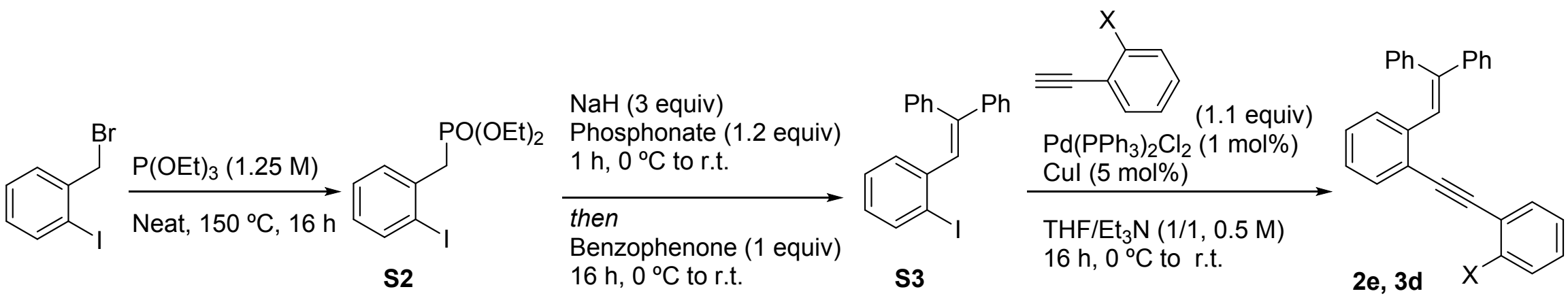

Step 1:[9] 2-(Bromomethyl)iodobenzene (1.90 g, 6.40 mmol) was added to a sealable Schlenk flask and an Ar atmosphere was established. The product was dissolved in $P(OEt)_3$ (5 mL, 1.25 M), and the flask was sealed and heated to 150 ºC with vigorous stirring for 16 h. Then, the reaction crude was subjected to short-path vacuum distillation to remove the excess $P(OEt)_3$ and subsequently purified though flash chromatography on silica gel using a gradient of Hex/AcOEt (1:1 to 0:1) as eluents to obtain **S2** (2.17 g, 6.13 mmol, 95% yield). Spectroscopic data match those reported in the literature.

Step 2:[10] Diethyl (2-iodophenyl)methyl phosphonate **S2** (2.17 g, 6.13 mmol, 1.2 equiv) was added to a Schlenk flask and Ar atmosphere was established. The product was dissolved in anhydrous THF (13 mL, 0.5 M) and cooled in an ice bath. NaH (60% dispersion in paraffin oil) (408.4 mg, 15.3 mmol, 3 equiv) was added in small portions, and after complete addition, the flask was sealed and allowed to warm to room temperature and react for 1 h. After that time, the flask is submerged again in the ice bath, and a previously prepared solution of benzophenone (930 mg, 5 mmol, 1 equiv.) in anhydrous THF (5 mL, 1 M) under Ar was added dropwise. The flask was sealed and allowed to warm to room temperature and react for 16 h. The crude was extracted with Et_2O and H_2O and the combined organic layers were dried over anhydrous Na_2SO_4, filtered and evaporated. The residue was purified by flash chromatography on silica gel using hexane as eluent to obtain iodostyrene **S3** as a white solid (1.13 g, 5.11 mmol, 58%).

---

[9] A. Sabourin, J. Dufour, J.-P. Vors, D. Bernier, J.-L. Montchamp *Eur. J. Org. Chem.* **2022**, *2022*, e202200141.
[10] R. Wu, Y. Chen, S. Zhu *ACS Catal.* **2023**, *13*, 132–140.

1-(2,2-Diphenylethenyl)-2-iodobenzene (S3)

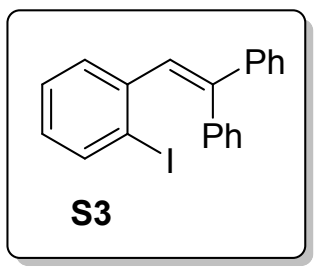

R_f = 0.20 (Hexane). m.p. = 90–91 ºC.

^{1}H NMR (400 MHz, $CDCl_3$) δ 7.75 (dd, *J* = 7.9, 1.3 Hz, 1H), 7.32 – 7.22 (m, 5H), 7.17 – 7.12 (m, 3H), 7.07 – 7.00 (m, 2H), 6.91 – 6.85 (m, 1H), 6.81 (s, 1H), 6.76 – 6.68 (m, 2H).

^{13}C NMR (101 MHz, $CDCl_3$) δ 144.2 (C), 142.8 (C), 141.9 (C), 139.6 (C), 138.9 (CH), 132.0 (CH), 131.0 (CH), 130.8 (2xCH), 128.4 (2xCH), 128.32 (2xCH), 128.29 (2xCH), 128.26 (CH), 128.0 (CH), 127.6 (CH), 127.5 (CH), 101.6 (C).

HRMS (ESI-TOF) m/z: $[M+H]^+$ Calcd for $[C_{20}H_{16}I]^+$ 383.0291. Found 383.0288.

Step 3: In a sealable Schlenk flask under Ar atmosphere, CuI (1 mol%, 3 mg, 0.01 mmol), $PdCl_2(PPh_3)_2$ (5 mol%, 35 mg, 0.05mmol) and 1-(2,2-diphenylethenyl)-2-iodobenzene **S3** (382 mg, 1 mmol) were suspended in a mixture of anhydrous Et_3N and THF (1:1, 0.5 M) and cooled in an ice-water bath. Then, the appropriate acetylene (1.1 equiv) was added, and the Schlenk flask was sealed. After 30 minutes, the mixture was allowed to warm to room temperature and was stirred until completion of the reaction as monitored by TLC (16-24 h). The crude was extracted with DCM and H_2O and the combined organic layers were dried over anhydrous Na_2SO_4, filtered and evaporated. The residue was purified by flash chromatography on silica gel using mixtures of hexane and EtOAc as eluents to obtain the desired enynes **2e** and **3d**.

2-((2-(2,2-Diphenylvinyl)phenyl)ethynyl)anisole (2e)

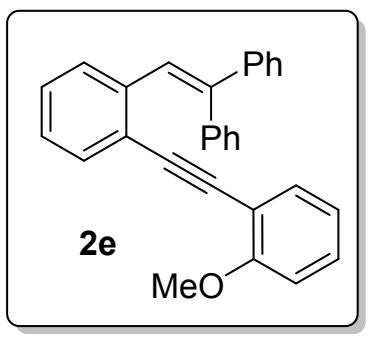

Obtained as white solid (327.4 mg, 0.847 mmol, 85%) using 1-ethynyl-2-methoxybenzene (145 mg, 1.10 mmol) as acetylene. R_f = 0.40 (Hexane/AcOEt 97/3). m.p. = 131–132 ºC

^{1}H NMR (400 MHz, $CDCl_3$) δ 7.57 (s, 1H), 7.54 (dd, *J* = 7.7, 0.9 Hz, 1H), 7.47 (dd, *J* = 7.6, 1.7 Hz, 1H), 7.44 – 7.40 (m, 2H), 7.36 – 7.22 (m, 9H), 7.09 (td, *J* = 7.5, 1.3 Hz, 1H), 6.95 – 6.88 (m, 2H), 6.85 – 6.80 (m, 2H), 3.52 (s, 3H).

^{13}C NMR (101 MHz, $CDCl_3$) δ 160.3 (C), 143.9 (C), 143.7 (C), 140.6 (C), 139.6 (C), 133.2 (CH), 132.0 (CH), 130.8 (2xCH), 129.8 (CH), 129.2 (CH), 128.6 (2xCH), 128.3 (2xCH), 128.2 (2xCH), 127.6 (CH), 127.5 (CH), 127.4 (CH), 127.2 (CH), 126.6 (CH), 124.4 (C), 120.5 (CH), 112.8. (C), 110.6 (CH), 92.8 (C), 91.2 (C), 55.5 (CH_3).

HRMS (ESI-TOF) m/z: $[M+H]^+$ Calcd for $[C_{29}H_{23}O]^+$ 387.1743. Found 387.1740.

2-((2-(2,2-Diphenylvinyl)phenyl)ethynyl)aniline (3d)

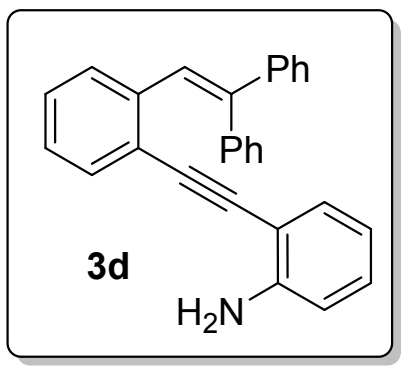

Obtained as white solid (300.7 mg, 0.809 mmol, 80%) using 2-ethynylaniline (130 mg, 1.10 mmol) as acetylene. m.p. = 108–109 ºC

^{1}H NMR (400 MHz, $CDCl_3$) δ 7.52 (dd, *J* = 7.7, 1.3 Hz, 1H), 7.42 – 7.31 (m, 7H), 7.30 – 7.25 (m, 3H), 7.22 – 7.16 (m, 2H), 7.15 – 7.07 (m, 2H), 6.97 (td, *J* = 7.7, 1.4 Hz, 1H), 6.85 (d, *J* = 7.9 Hz, 1H), 6.68 (td, *J* = 7.5, 1.1 Hz, 1H), 6.62 (dd, *J* = 8.1, 1.1 Hz, 1H), 4.01 (bs, 2H).

^{13}C NMR (101 MHz, $CDCl_3$) δ 148.1 (C), 144.4 (C), 143.1 (C), 139.9 (C), 139.5 (C), 132.0 (CH), 131.6 (CH), 130.7 (2xCH), 129.8 (CH), 129.3 (CH), 128.5 (2xCH), 128.4 (2xCH), 128.0 (2xCH), 127.9 (CH), 127.6 (CH), 127.5 (CH), 127.1 (CH), 126.8 (CH), 124.0 (C), 117.9 (CH), 114.3 (CH), 108.0 (C), 94.1 (C), 91.6 (C).

HRMS (ESI-TOF) m/z: $[M+H]^+$ Calcd for $[C_{28}H_{22}N]^+$ 372.1747. Found 372.1743.

Method C:

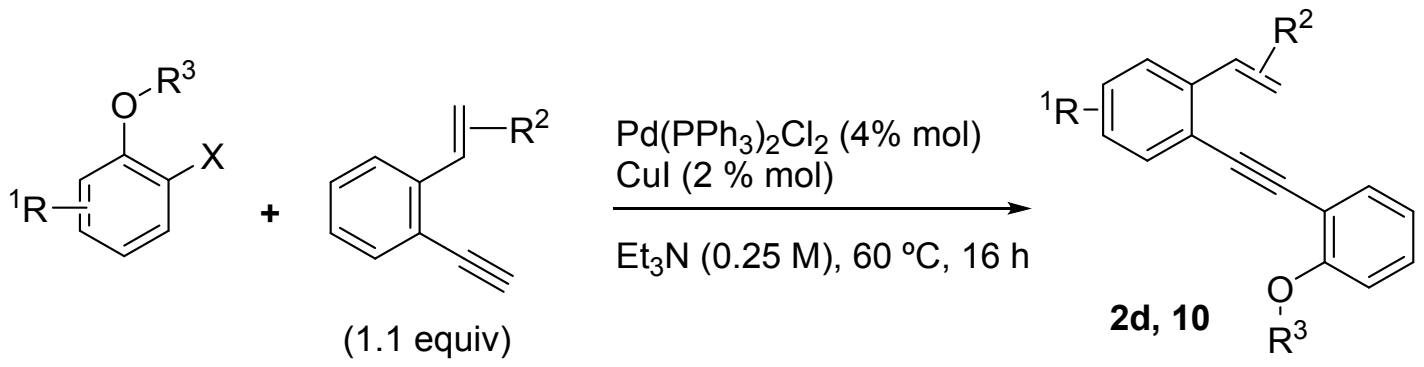

In a sealable Schlenk flask, CuI (2 mol%, 8 mg, 0.04 mmol), $PdCl_2(PPh_3)_2$ (4 mol%, 56 mg, 0.08 mmol) and the corresponding *o*-substituted halobenzene derivative (if solid) were added and an Ar atmosphere was established. The reagents were suspended in anhydrous Et_3N, and the corresponding *o*-substituted halobenzene derivative (if liquid) was added. Then, the appropriate terminal enyne (1.1 equiv) was added and the Schlenk flask was sealed. The mixture was heated to 60 ºC and stirred until completion of the reaction as monitored by TLC (16-24 h). The crude was extracted with DCM and H_2O and the combined organic layers were dried over anhydrous Na_2SO_4, filtered and evaporated. The residue was purified by flash chromatography on silica gel using mixtures of hexane and EtOAc as eluents to obtain the enynes **2d**, **10**.

1-Methoxy-2-((2-(2-methylprop-1-en-1-yl)phenyl)ethynyl)naphthalene (2d)

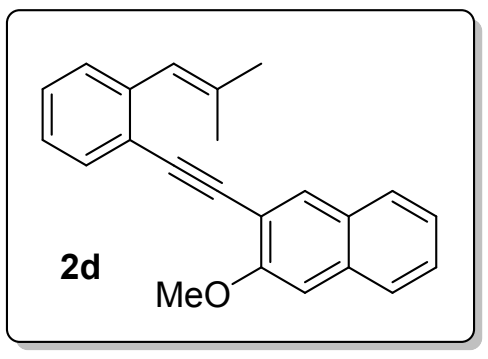

Obtained as yellow solid (435.4 mg, 1.39 mmol, 70%) using 2-bromo-3-methoxynaphthalene (474 mg, 2.0 mmol) and 1-ethynyl-2-(2-methylprop-1-en-1-yl)benzene (343 mg, 2.2 mmol). R_f = 0.53 (Hexane/AcOEt 95/5). m.p. = 94–95 ºC

^{1}H NMR (400 MHz, CDCl$_3$) δ 8.00 (s, 1H), 7.78 – 7.69 (m, 2H), 7.63 – 7.58 (m, 1H), 7.45 (ddd, J = 8.2, 6.9, 1.3 Hz, 1H), 7.38– 7.27 (m, 3H), 7.23 – 7.17 (m, 1H), 7.14 (s, 1H), 6.75 – 6.71 (m, 1H), 4.03 (s, 3H), 2.03 (d, J = 1.4 Hz, 3H), 1.89 (d, J = 1.4 Hz, 3H).

^{13}C NMR (101 MHz, CDCl$_3$) δ 156.9 (C), 140.7 (C), 136.6 (C), 134.2 (C), 133.4 (CH), 132.2 (CH), 129.0 (CH), 128.4 (C), 127.8 (CH), 127.5 (CH), 127.0 (CH), 126.6 (CH), 125.8 (CH), 124.2 (CH), 124.1 (CH), 122.8 (C), 114.6 (C), 105.3 (CH), 93.0 (C), 90.0 (C), 55.9 (CH$_3$), 26.9 (CH$_3$), 19.7 (CH$_3$)

HRMS (EI+) m/z: $[M]^+$ Calcd for $[C_{23}H_{20}O]^+$ 312.1509. Found 312.1510.

2,2'-((2,5-Dimethoxy-1,4-phenylene)bis(ethyne-2,1-diyl))bis(prop-1-en-2-ylbenzene) (10)

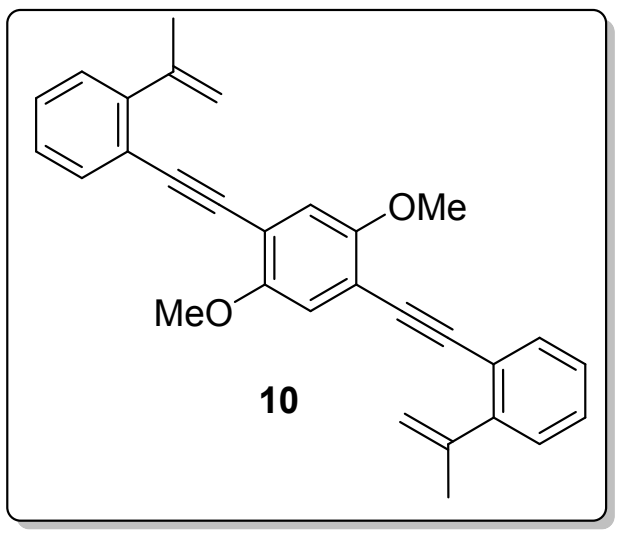

Obtained as yellow solid (619.0 mg, 1.48 mmol, 74%) using 1,4-diiodo-2,5-dimethoxybenzene (780 mg, 2.0 mmol), 1-ethynyl-2-(prop-1-en-2-yl)benzene (625 mg, 4.4 mmol) and doubled quantities respect to the general method for the rest of the reagents and solvents to account for the second coupling. R_f = 0.25 (Hexane/DCM 8/2). m.p. = 102–103 ºC.

^{1}H NMR (400 MHz, CDCl$_3$) δ 7.53 (ddd, J = 7.5, 1.5, 0.8 Hz, 2H), 7.29 – 7.16 (m, 6H), 6.93 (s, 2H), 5.23 (p, J = 1.5 Hz, 2H), 5.22 – 5.19 (m, 2H), 3.84 (s, 6H), 2.23 (dd, J = 1.5, 0.9 Hz, 6H).

^{13}C NMR (101 MHz, $CDCl_3$) δ 154.4 (2xC), 146.2 (2xC), 145.2 (2xC), 133.2 (2xCH), 128.7 (2xCH), 128.2 (2xCH), 127.0 (2xCH), 121.0 (2xC), 116.1 (2xCH), 115.8 ($2xCH_2$), 114.0 (2xC), 95.0 (2xC), 89.5 (2xC), 56.7 ($2xCH_3$), 23.6 ($2xCH_3$).

HRMS (EI+) m/z: $[M]^+$ Calcd for $[C_{30}H_{26}O_2]^+$ 418.1927. Found 418.1925.

General procedures for the synthesis of B-doped PAHs

Method D: General procedure for the synthesis of B-O-doped PAHs with B-OH

To a solution of the appropriate enyne **2a**, **2e** or **6a-b** (1 equiv) in anhydrous $CHCl_3$ (0.25 M) prepared under argon in a sealable Schlenk flask, cold BCl_3 (1.0 M in DCM, 3 equiv) was added dropwise, and the mixture was stirred for 4 h at room temperature (in the case of compounds **6a-b**) or 16 h at 70 ºC (in the case of compounds **2a**,**e**). The formed B-Cl intermediate was quenched with distilled water, and the mixture was vigorously stirred for 10 minutes at room temperature to ensure full transformation. The crude was then diluted with CH_2Cl_2, and the organic layer washed with aqueous HCl (1 M), distilled water, and brine, dried over anhydrous Na_2SO_4, filtered and concentrated under reduced pressure. The resulting residue was purified either by trituration with hexane until the washings become clear, or through column chromatography employing mixtures of CH_2Cl_2 and AcOEt as indicated, to obtain the desired B-OH functionalized products **4a**, **4e**, **8a** and **8d**. Specific examples required small modifications of the general procedure, as indicated below.

Method E: General procedure for the synthesis of B-X-doped PAHs with B-R (or B-OMe)

To a solution of the appropriate enyne **2**, **3**, **6**, **7a**, **10**, **12**, **14**, **16** (1 equiv) in anhydrous $CHCl_3$ (0.25 M) prepared under argon in a sealable Schlenk flask, cold BCl_3 (1.0 M in DCM, 3 equiv) was added dropwise, and the mixture was stirred for 4 h at room temperature (in the case of compounds **8**, **9**, **11**, **13** and **17**) or 16 h at 70 ºC (in the case of compounds **4**, **5**, **15**). The crude reaction mixture containing the formed B-Cl intermediate was evaporated in vacuo to remove the solvent and excess boron reagent, and then it was redissolved in anhydrous toluene (0.1 M). A commercial solution in THF of the corresponding Grignard reagent was added to the mixture (3 equiv) with strong stirring, and the reaction was allowed to react at room temperature for 16 h. The crude was then treated with a mixture of Tol/^{i}PrOH (4/1) to quench excess Grignard reagent, filtered over Celite, and concentrated under reduced pressure. The resulting residue was purified by column chromatography employing mixtures of hexane and AcOEt as indicated to obtain the desired B-R functionalized products. Specific examples required small modifications of the general procedure, as indicated below.

Alternatively, the evaporated B-Cl intermediate can be quenched with anhydrous MeOH and stirred to afford a suspension of crude **8b**. The solid was allowed to sediment, the liquid was decanted, and the solid was washed with further portions of anhydrous MeOH until the washings were colorless. The remaining solid was dried under vacuum to afford pure **8b** as a moisture sensitive white powder. NMR data was recorded in a J-Young sealed tube under Ar atmosphere to avoid hydrolysis of the methoxy group.

Characterization data of B-doped PAHs

11-(Prop-1-en-2-yl)benzo[*e*]indeno[1,2-*c*][1,2]oxaborinin-6(11*H*)-ol (4a)

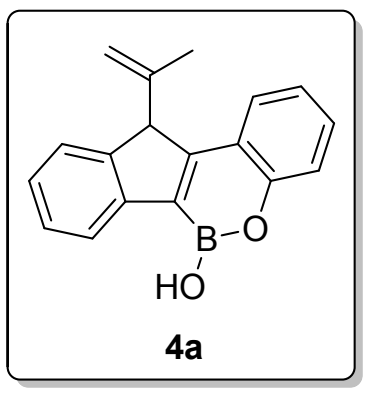

Obtained as white powder (201.7 mg, 0.751 mmol, 94%) following method D from **2a** (210 mg, 0.80 mmol). Purified by hexane trituration. R_f = 0.18 (CH_2Cl_2). m.p. = 194–195 ºC

^{1}H NMR (400 MHz, Acetone-*d6*) δ 8.20 (s, 1H), 7.95 (dd, *J* = 7.9, 1.7 Hz, 1H), 7.86 (d, *J* = 7.5 Hz, 1H), 7.46 – 7.32 (m, 3H), 7.29 (dd, *J* = 8.3, 1.2 Hz, 1H), 7.26 (td, *J* = 7.4, 1.2 Hz, 1H), 7.21 – 7.15 (m, 1H), 5.58 – 5.53 (m, 1H), 5.21 (p, *J* = 1.5 Hz, 1H), 4.87 (s, 1H), 1.13 (dd, *J* = 1.5, 0.9 Hz, 3H).

^{11}B NMR (128 MHz, Acetone-*d6*) δ 26.7.

^{13}C NMR (101 MHz, Acetone-*d6*) δ 161.1 (C), 153.4 (C), 147.2 (C), 145.81 (C), 145.77 (C), 129.8 (CH), 128.2 (CH), 126.6 (CH), 125.7 (CH), 123.9 (CH), 123.8 (C), 123.7 (CH), 123.1 (CH), 119.4 (CH), 115.8 (CH_2), 59.7 (CH), 17.5 (CH_3).

HRMS (APCI+) m/z: $[M+H]^+$ Calcd for $[C_{18}H_{16}BO_2]^+$ 275.1237. Found 275.1240.

6-Mesityl-11-(prop-1-en-2-yl)-6,11-dihydrobenzo[*e*]indeno[1,2-*c*][1,2]oxaborinine (4b)

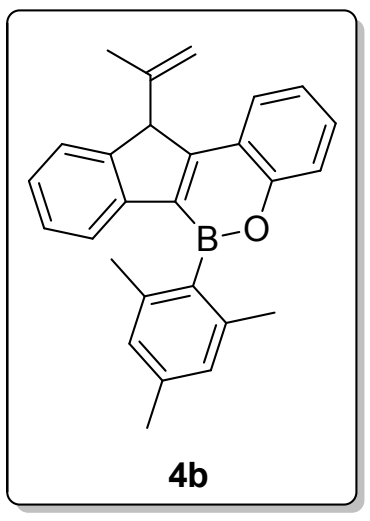

Obtained as white powder (64.1 mg, 0.170 mmol, 85%) from **2a** (52.4 mg, 0.20 mmol) following method E. R_f = 0.57 (Hexane/AcOEt 94/6). m.p. = 169–170 ºC

^{1}H NMR (400 MHz, $CDCl_3$) δ 8.17 (dd, *J* = 7.9, 1.6 Hz, 1H), 7.65 (dd, *J* = 8.2, 1.3 Hz, 1H), 7.56 – 7.47 (m, 2H), 7.34 (ddd, *J* = 8.3, 7.2, 1.3 Hz, 1H), 7.25 (td, *J* = 7.4, 1.4 Hz, 1H), 7.20 (td, *J* = 7.4, 1.4 Hz, 1H), 7.09 – 7.05 (m, 1H), 7.00 (bs, 2H), 5.58 – 5.53 (m, 1H), 5.29 – 5.25 (m, 1H), 4.91 (s, 1H), 2.42 (s, 3H), 2.28 (s, 3H), 2.26 (s, 3H), 1.22 (bs, 3H).

^{11}B NMR (128 MHz, $CDCl_3$) δ 32.1.

^{13}C NMR (101 MHz, $CDCl_3$) δ 160.0 (C), 153.4 (C), 146.3 (C), 145.1 (2xC), 140.0 (C), 139.9 (C), 138.5 (C), 129.3 (CH), 127.8 (CH), 127.6 (2xCH), 126.2 (CH), 125.2 (CH), 123.5 (C), 123.44 (CH), 123.36 (CH), 122.3 (CH), 120.1 (CH), 115.5 (CH_2), 59.1 (CH), 22.3 (CH_3), 22.1 (CH_3), 21.5 (CH_3), 17.4 (CH_3).

HRMS (APCI+) m/z: $[M]^+$ Calcd for $[C_{27}H_{25}BO]^+$ 376.1993. Found 376.1994.

6-Mesityl-13-(prop-1-en-2-yl)-6,13-dihydrobenzo[*e*]benzo[5,6]indeno[1,2-*c*][1,2]oxaborinine (4c)

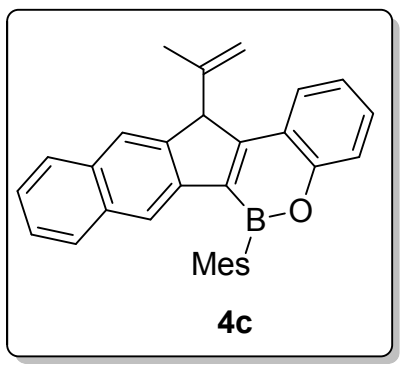

Obtained as orange solid (64.9 mg, 0.152 mmol, 50%) from **2c** (125 mg, 0.40 mmol) following method E. R_f = 0.55 (Hexane/AcOEt 97/3). m.p. = 204–205 ºC

^{1}H NMR (400 MHz, $CDCl_3$) δ 8.17 (dd, *J* = 7.9, 1.6 Hz, 1H), 7.85 (s, 1H), 7.84 – 7.79 (m, 1H), 7.68 – 7.64 (m, 1H), 7.63 (dd, *J* = 8.3, 1.2 Hz, 1H), 7.52 (ddd, *J* = 8.4, 7.1, 1.6 Hz, 1H), 7.43 – 7.30 (m, 4H), 7.01 (s, 1H), 6.99 (s, 1H), 5.59 (bs, 1H), 5.28 – 5.24 (m, 1H), 5.06 (s, 1H), 2.43 (s, 3H), 2.27 (s, 3H), 2.24 (s, 3H), 1.22 (s, 3H).

^{11}B NMR (128 MHz, $CDCl_3$) δ 42.2

^{13}C NMR (101 MHz, $CDCl_3$) δ 160.4 (C), 153.5 (C), 145.5 (C), 144.5 (C), 143.7 (C), 139.8 (2xC), 138.4 (C), 133.7 (C), 132.6 (C), 129.5 (CH), 128.4 (CH), 127.8 (CH), 127.6

(2xCH), 125.33 (CH), 125.29 (CH), 125.2 (CH), 123.4 (C), 123.3 (CH), 122.0 (CH), 120.2 (CH), 119.9 (CH), 115.1 (CH_2), 58.3 (CH), 22.1 (CH_3), 21.9 (CH_3), 21.4 (CH_3), 17.3 (CH_3).

HRMS (APCI+) m/z: $[M-H]^+$ Calcd for $[C_{31}H_{26}BO]^+$ 425.2071. Found 425.2072.

5-Mesityl-13-(prop-1-en-2-yl)-5,13-dihydroindeno[1,2-*c*]naphtho[2,3-*e*][1,2]oxaborinine (4d)

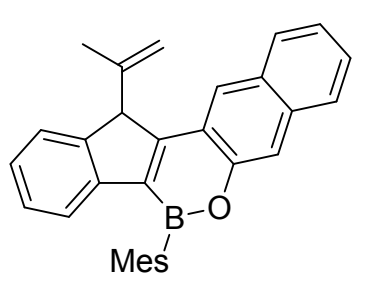

4d

Obtained as white solid (132.5. mg, 0.309 mmol, 78%) from **2d** (94 mg, 0.30 mmol) following method E. R_f = 0.42 (Hexane/AcOEt 97/3). m.p. = 256–257 ºC

^{1}H NMR (400 MHz, $CDCl_3$) δ 8.65 (s, 1H), 8.04 (s, 1H), 8.02 – 7.98 (m, 1H), 7.95 – 7.89 (m, 1H), 7.58 – 7.45 (m, 3H), 7.27 (td, *J* = 7.4, 1.3 Hz, 1H), 7.24 – 7.17 (m, 1H), 7.10 – 7.05 (m, 1H), 7.03 – 6.98 (m, 2H), 5.66 (bs, 1H), 5.35 – 5.31 (m, 1H), 5.01 (s, 1H), 2.43 (s, 3H), 2.32 (s, 3H), 2.29 (s, 3H), 1.26 – 1.21 (m, 3H).

^{11}B NMR (128 MHz, $CDCl_3$) δ 43.1.

^{13}C NMR (101 MHz, $CDCl_3$) δ 159.5 (C), 150.7 (C), 146.5 (C), 145.4 (C), 145.1 (C), 139.90 (C), 139.88 (C), 138.6 (C), 133.8 (C), 130.0 (C), 128.5 (CH), 127.8 (CH), 127.7 (2xCH), 127.4 (CH), 127.0 (CH), 126.3 (CH), 125.1 (CH), 124.6 (CH), 123.8 (C), 123.5 (CH), 122.3 (CH), 115.8 (CH), 115.6 (CH_2), 59.3 (CH), 22.3 (CH_3), 22.1 (CH_3), 21.6 (CH_3), 17.4 (CH_3).

HRMS (APCI+) m/z: $[M]^+$ Calcd for $[C_{31}H_{27}BO]^+$ 426.2149. Found 426.2153.

11-(Diphenylmethylene)benzo[*e*]indeno[1,2-*c*][1,2]oxaborinin-6(11*H*)-ol (4e)

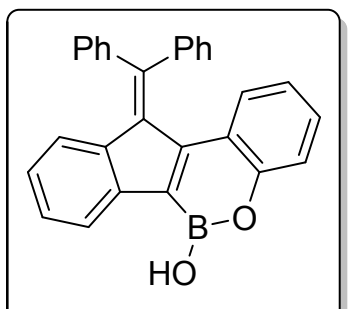

4e

Obtained as deep red solid (54.5 mg, 0.136 mmol, 68%) from **2e** (77 mg, 0.20 mmol) following method D. R_f = 0.40 (CH_2Cl_2). m.p. = 192–193 ºC

^{1}H NMR (400 MHz, Acetone-*d*$_6$) δ 8.18 (s, 1H), 7.88 – 7.83 (m, 1H), 7.67 – 7.54 (m, 3H), 7.48 – 7.42 (m, 2H), 7.26 – 7.08 (m, 7H), 7.05 – 6.97 (m, 2H), 6.82 – 6.75 (m, 1H), 6.47 – 6.40 (m, 1H), 6.25 (d, *J* = 7.9 Hz, 1H).

^{11}B NMR (128 MHz, Acetone-*d6*) δ 27.1.

^{13}C NMR (101 MHz, Acetone-*d6*) δ 153.4 (C), 152.7 (C), 152.0 (C), 144.1 (C), 144.0 (C), 143.6 (C), 141.0 (C), 138.7 (C), 134.1 (2xCH), 131.9 (2xCH), 130.5 (CH), 130.0 (CH), 129.8 (2xCH), 129.04 (CH), 129.02 (2xCH), 128.1 (CH), 127.7 (CH), 126.1 (CH), 123.7 (CH), 122.9 (CH), 121.9 (C), 121.3 (CH), 119.1 (CH).

HRMS (APCI+) m/z: $[M+H]^+$ Calcd for $[C_{28}H_{20}BO_2]^+$ 399.1550. Found 399.1552.

6-Methyl-11-(prop-1-en-2-yl)-6,11-dihydro-5*H*-benzo[*e*]indeno[1,2-*c*][1,2]azaborinine (5a)

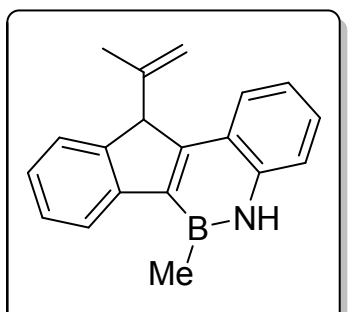

5a

Obtained as off-white powder (98.4 mg, 0.363 mmol, 60%) from **3a** (148 mg, 0.60 mmol) following method E. R_f = 0.50 (Hexane/AcOEt 95/5). m.p. = 185–186 ºC.

^{1}H NMR (400 MHz, $CDCl_3$) 8.14 (d, *J* = 8.0 Hz, 1H), 7.81 (d, *J* = 7.6 Hz, 1H), 7.75 (bs, 1H), 7.52 – 7.44 (m, 1H), 7.42 – 7.32 (m, 2H), 7.31 – 7.21 (m, 2H), 7.16 (ddd, *J* = 8.2, 7.0, 1.3 Hz, 1H), 5.48 (bs, 1H), 5.23 – 5.15 (m, 1H), 4.81 (s, 1H), 1.12 (s, 3H), 1.11 – 1.09 (m, 3H).

^{11}B NMR (128 MHz, $CDCl_3$) δ 36.7.

^{13}C NMR (101 MHz, $CDCl_3$) δ 155.9 (C), 147.4 (C), 146.7 (C), 146.4 (C), 140.0 (C), 127.7 (CH), 127.4 (CH), 125.42 (CH), 125.40 (CH), 123.5 (CH), 123.2 (C), 122.6 (CH), 121.1 (CH), 118.3 (CH), 114.7 (CH_2), 59.1 (CH), 17.5 (CH_3).

HRMS (APCI+) m/z: $[M+H]^+$ Calcd for $[C_{19}H_{19}BN]^+$ 272.1605. Found 272.1605.

6-Mesityl-11-(prop-1-en-2-yl)-6,11-dihydro-5*H*-benzo[*e*]indeno[1,2-*c*][1,2]azaborinine (5b)

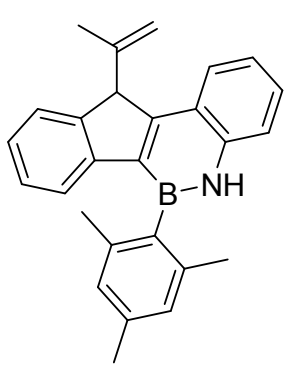

5b

Obtained as off-white powder (103.0 mg, 0.274 mmol, 69%) from **3a** (99 mg, 0.40 mmol) following method E. R_f = 0.25 (Hexane/AcOEt 97/3). m.p. = 140–141 ºC

^{1}H NMR (400 MHz, $CDCl_3$) δ 8.24 (d, *J* = 8.1 Hz, 1H), 7.92 (bs, 1H), 7.50 – 7.40 (m, 2H), 7.33 (d, *J* = 8.2 Hz, 1H), 7.28 – 7.23 (m, 1H), 7.22 – 7.16 (m, 1H), 7.13 (t, *J* = 7.5 Hz, 1H), 7.02 – 6.93 (m, 3H), 5.54 (bs, 1H), 5.26 – 5.20 (m, 1H), 4.93 (s, 1H), 2.42 (s, 3H), 2.20 (s, 3H), 2.18 (s, 3H), 1.16 (s, 3H).

^{11}B NMR (128 MHz, $CDCl_3$) δ 35.5.

^{13}C NMR (126 MHz, $CDCl_3$) δ 156.9 (C), 146.9 (C), 146.4 (C), 146.2 (C), 140.13 (C), 140.12 (C), 140.06 (C), 137.8 (C), 127.9 (CH), 127.6 (CH), 127.4 (2xCH), 125.6 (2xCH), 123.5 (C), 123.4 (CH), 122.1 (CH), 121.6 (CH), 118.8 (CH), 114.8 (CH_2), 59.1 (CH), 22.7 (CH_3), 22.6 (CH_3), 21.5 (CH_3), 17.3 (CH_3).

HRMS (APCI+): decomposes.

6-Phenyl-11-(prop-1-en-2-yl)-6,11-dihydro-5*H*-benzo[*e*]indeno[1,2-*c*][1,2]azaborinine (5c)

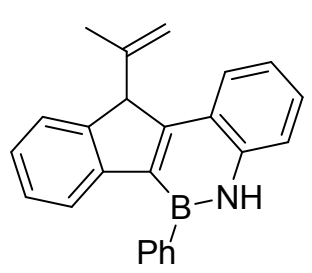

5c

Obtained as off-white powder (108.1 mg, 0.288 mmol, 72%) from **3a** (99 mg, 0.40 mmol) following method E. R_f = 0.38 (Hexane/AcOEt 97/3). m.p. = 129–130 ºC

^{1}H NMR (400 MHz, $CDCl_3$) δ 8.25 (d, *J* = 8.1 Hz, 1H), 7.97 (bs, 1H), 7.88 – 7.81 (m, 2H), 7.63 – 7.58 (m, 1H), 7.57 – 7.47 (m, 4H), 7.44 (ddd, *J* = 8.3, 7.0, 1.4 Hz, 1H), 7.35 (dd, *J* = 8.1, 1.3 Hz, 1H), 7.27 – 7.18 (m, 3H), 5.53 (bs, 1H), 5.26 – 5.20 (m, 1H), 4.90 (s, 1H), 1.17 (bs, 3H).

^{11}B NMR (128 MHz, $CDCl_3$) δ 34.7.

^{13}C NMR (101 MHz, $CDCl_3$) δ 157.9 (C), 146.6 (C), 146.5 (C), 146.3 (C), 139.8 (C), 132.8 (2xCH), 128.8 (CH), 128.19 (2xCH), 128.17 (CH), 127.2 (CH), 125.7 (CH), 125.6 (CH), 123.51 (CH), 123.47 (C), 123.1 (CH), 121.7 (CH), 118.8 (CH), 114.9 (CH_2), 59.1 (CH), 17.5 (CH_3).

HRMS (APCI+) m/z: $[M+H]^+$ Calcd for $[C_{24}H_{21}BN]^+$ 334.1761. Found 334.1762.

11-(Diphenylmethylene)-6-mesityl-6,11-dihydro-5*H*-benzo[*e*]indeno[1,2-*c*][1,2]azaborinine (5d)

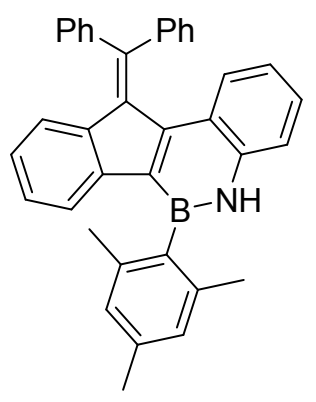

5d

Obtained as red powder (49.3 mg, 0.099 mmol, 25%) from **3d** (148 mg, 0.40 mmol) following method E with slight modifications (2,4,6-tri*tert*butylpyridine (1 equiv) and BCl_3 (4 equiv) are employed to limit decomposition). R_f = 0.38 (Hexane/AcOEt 97/3). m.p. > 275 ºC.

^{1}H NMR (400 MHz, $CDCl_3$) δ 7.75 (s, 1H), 7.60 – 7.40 (m, 6H), 7.20 – 7.02 (m, 7H), 6.97 (s, 2H), 6.96 – 6.88 (m, 2H), 6.72 (ddd, *J* = 8.4, 6.6, 2.1 Hz, 1H), 6.53 (ddd, *J* = 8.3, 6.8, 1.5 Hz, 1H), 6.26 (d, *J* = 7.9 Hz, 1H), 2.40 (s, 3H), 2.24 (s, 6H).

^{11}B NMR (128 MHz, $CDCl_3$) δ 35.7.

^{13}C NMR (101 MHz, $CDCl_3$) δ 150.8 (C), 148.5 (C), 145.0 (C), 143.7 (C), 143.3 (C), 140.8 (C), 140.32 (C), 140.25 (2xC), 138.9 (C), 137.7 (C), 133.5 (2xCH), 131.6 (2xCH), 129.5 (CH), 129.2 (CH), 128.9 (2xCH), 128.7 (CH), 128.2 (2xCH), 127.6 (CH), 127.4 (2xCH), 126.1 (CH), 125.2 (CH), 122.2 (CH), 122.0 (CH), 120.9 (C), 119.9 (CH), 118.3 (CH), 22.8 (2xCH_3), 21.5 (CH_3).

HRMS (APCI+) m/z: $[M+H]^+$ Calcd for $[C_{37}H_{31}BN]^+$ 500.2546. Found 500.2544.

12-Methyl-5*H*-benzo[*e*]naphtho[1,2-*c*][1,2]oxaborinin-5-ol (8a)

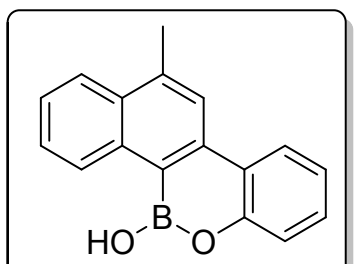

8a

Obtained as white powder (44.2 mg, 0.170 mmol, 85%) from **6a** (50 mg, 0.20 mmol) following method D. Purified by trituration with hexane. R_f = 0.31 (Hex/DCM 4/6). m.p. = 229–230 ºC

^{1}H NMR (400 MHz, Acetone-*d6*) δ 9.52 – 9.47 (m, 1H), 8.57 (s, 1H), 8.45 – 8.40 (m, 1H), 8.35 (s, 1H), 8.16 – 8.10 (m, 1H), 7.68 – 7.57 (m, 2H), 7.47 – 7.40 (m, 1H), 7.32 – 7.25 (m, 2H), 2.88 – 2.83 (m, 3H).

^{11}B NMR (128 MHz, Acetone-*d6*) δ 28.1.

^{13}C NMR (101 MHz, Acetone-*d6*) δ 152.4 (C), 141.7 (C), 140.6 (C), 138.3 (C), 133.0 (C), 130.6 (CH), 129.9 (CH), 127.6 (CH), 126.8 (CH), 125.3 (CH), 124.9 (CH), 123.9 (C), 123.3 (CH), 121.7 (CH), 119.7 (CH), 20.5 (CH_3).

HRMS (APCI+) m/z: $[M+H]^+$ Calcd for $[C_{17}H_{14}BO_2]^+$ 261.1081. Found 261.1081.

5-Methoxy-12-methyl-5*H*-benzo[*e*]naphtho[1,2-*c*][1,2]oxaborinine (8b)

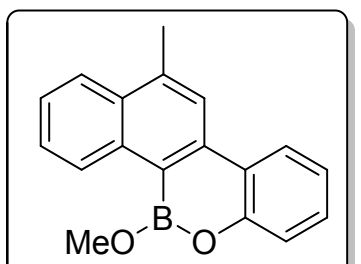

8b

Obtained as moisture sensitive white solid (62.0 mg, 0.228 mmol, 57%) from **6a** (100 mg, 0.40 mmol) following method E.

^{1}H NMR (400 MHz, $CDCl_3$) δ 9.35 (d, *J* = 8.2 Hz, 1H), 8.22 (d, *J* = 7.5 Hz, 1H), 8.11 – 8.00 (m, 2H), 7.64 (ddd, *J* = 8.5, 6.7, 1.5 Hz, 1H), 7.58 (ddd, *J* = 8.2, 6.8, 1.5 Hz, 1H), 7.44 (ddd, *J* = 8.3, 6.8, 1.5 Hz, 1H), 7.39 (dd, *J* = 8.1, 1.6 Hz, 1H), 7.31 – 7.24 (m, 1H), 4.12 (s, 3H), 2.81 (s, 3H).

^{11}B NMR (128 MHz, $CDCl_3$) δ 28.2.

^{13}C NMR (101 MHz, $CDCl_3$) δ 151.3 (C), 140.4 (C), 139.3 (C), 137.0 (C), 132.0 (C), 129.8 (CH), 128.8 (CH), 126.8 (CH), 125.9 (CH), 124.2 (CH), 124.1 (CH), 123.1 (C), 122.5 (CH), 120.8 (CH), 119.2 (CH), 51.4 (CH_3), 20.7 (CH_3).

HRMS (APCI+): substitution of the OMe group for OH was observed.

5-Mesityl-12-methyl-5*H*-benzo[*e*]naphtho[1,2-*c*][1,2]oxaborinine (8c)

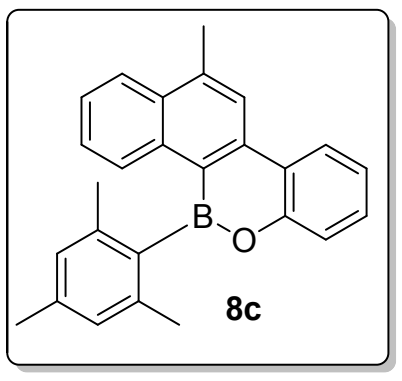

Obtained as white powder (67.2 mg, 0.185 mmol, 93%) from **6a** (50 mg, 0.20 mmol) following method E. R_f = 0.26 (Hexane). m.p. = 189–190 ºC

^{1}H NMR (400 MHz, CDCl$_3$) δ 8.47 (dd, *J* = 8.2, 1.5 Hz, 1H), 8.36 (s, 1H), 8.34 (d, *J* = 8.6 Hz, 1H), 8.11 (dd, *J* = 8.4, 1.3 Hz, 1H), 7.62 (dd, *J* = 8.1, 1.4 Hz, 1H), 7.57 – 7.50 (m, 2H), 7.42 (ddd, *J* = 8.3, 7.1, 1.4 Hz, 1H), 7.33 (ddd, *J* = 8.4, 6.8, 1.4 Hz, 1H), 6.99 (s, 2H), 2.92 (s, 3H), 2.42 (s, 3H), 2.17 (s, 6H).

^{11}B NMR (128 MHz, CDCl$_3$) δ 43.9.

^{13}C NMR (101 MHz, CDCl$_3$) δ 152.0 (C), 141.2 (C), 139.6 (C), 139.0 (2xC), 138.2 (C), 137.9 (C), 132.1 (C), 129.2 (CH), 128.0 (2xCH), 127.9 (CH), 127.3 (CH), 126.1 (CH), 124.5 (CH), 124.2 (CH), 123.4 (CH), 123.2 (C), 121.1 (CH), 120.4 (CH), 22.2 (2xCH_3), 21.6 (CH_3), 21.1 (CH_3).

HRMS (APCI+) m/z: $[M]^+$ Calcd for $[C_{26}H_{23}BO]^+$ 362.1836. Found 362.1839.

12-Phenyl-5*H*-benzo[*e*]naphtho[1,2-*c*][1,2]oxaborinin-5-ol (8d)

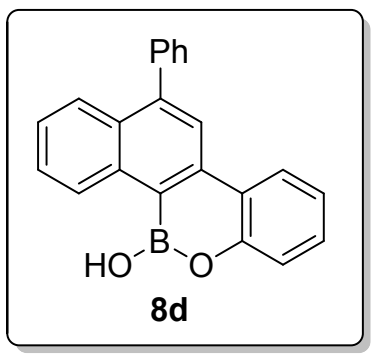

Obtained as white powder (168.2 mg, 0.522 mmol, 87%) from **6b** (186 mg, 0.60 mmol) following method D. Purified by trituration with hexane. R_f = 0.37 (CH_2Cl_2). m.p. >275 ºC

^{1}H NMR (400 MHz, CDCl$_3$) δ 9.37 (d, *J* = 8.5 Hz, 1H), 8.26 (dd, *J* = 8.1, 1.5 Hz, 1H), 8.22 (s, 1H), 7.93 (d, *J* = 8.4 Hz, 1H), 7.66 (ddd, *J* = 8.5, 6.8, 1.4 Hz, 1H), 7.60 – 7.48 (m, 6H), 7.47 – 7.41 (m, 1H), 7.35 (dd, *J* = 8.1, 1.4 Hz, 1H), 7.31 – 7.22 (m, 1H), 5.07 (s, 1H).

^{11}B NMR (128 MHz, CDCl$_3$) δ 28.2.

^{13}C NMR (101 MHz, CDCl$_3$) δ 151.3 (C), 145.5 (C), 141.0 (C), 140.7 (C), 137.7 (C), 131.2 (C), 130.1 (2xCH), 129.4 (CH), 129.3 (CH), 128.5 (2xCH), 127.9 (CH), 127.4 (CH), 126.6 (CH), 126.3 (CH), 124.4 (CH), 123.3 (C), 122.8 (CH), 121.3 (CH), 119.2 (CH).

HRMS (APCI+) m/z: $[M+H]^+$ Calcd for $[C_{22}H_{16}BO_2]^+$ 323.1237. Found 323.1232.

5-Mesityl-12-phenyl-5*H*-benzo[*e*]naphtho[1,2-*c*][1,2]oxaborinine (8e)

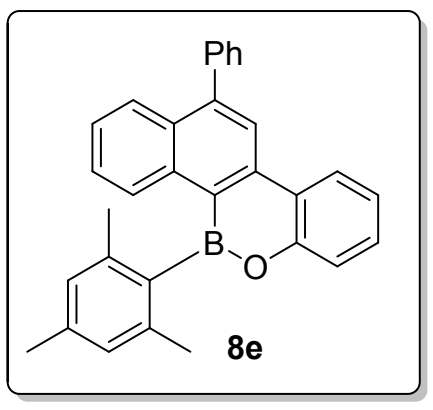

Obtained as off-white solid (234.3 mg, 0,552 mmol, 92%) from **6b** (186 mg, 0.60 mmol) following method E. R_f = 0.25 (Hexane). m.p. = 245–246 ºC

^{1}H NMR (400 MHz, CDCl$_3$) δ 8.44 (d, *J* = 8.3, 1.5 Hz, 1H), 8.42 (s, 1H), 8.40 (d, *J* = 8.5 Hz, 1H), 7.94 (dd, *J* = 8.5, 1.5 Hz, 1H), 7.66 – 7.49 (m, 7H), 7.46 – 7.36 (m, 2H), 7.33 (ddd, *J* = 8.4, 6.8, 1.5 Hz, 1H), 7.01 (s, 2H), 2.43 (s, 3H), 2.21 (s, 6H).

^{11}B NMR (128 MHz, CDCl$_3$) δ 42.2.

^{13}C NMR (101 MHz, CDCl$_3$) δ 152.0 (C), 146.5 (C), 140.9 (C), 139.2 (C), 139.0 (2xC), 138.4 (C), 138.3 (C), 131.2 (C), 130.1 (2xCH), 129.4 (CH), 128.6 (2xCH), 128.0 (3xCH), 127.6 (CH), 127.5 (CH), 126.8 (CH), 126.2 (CH), 124.3 (CH), 123.5 (CH), 123.2 (C), 121.5 (CH), 120.4 (CH), 22.3 (2xCH_3), 21.6 (CH_3).

HRMS (APCI+) m/z: $[M+H+MeCN-Mes]^+$ Calcd for $[C_{24}H_{17}BNO]^+$ 346.1397. Found 346.1399. The mass corresponding to a compound coming from the substitution of the mesityl groups for a MeCN molecule, used in the sample preparation, is observed.

12-Methyl-5-phenyl-5,6-dihydrobenzo[*e*]naphtho[1,2-*c*][1,2]azaborinine (9a)

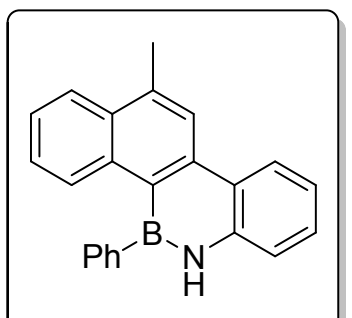

9a

Obtained as white solid (42.0 mg, 0.132 mmol, 22%) from **7a** (140 mg, 0.60 mmol) following method E with slight modifications (2,4,6-tri*tert*butylpyridine (1 equiv.) and BCl_3 (4 equiv.) are employed to limit decomposition). R_f = 0.42 (Hexane). m.p. = 176–177 ºC.

^{1}H NMR (400 MHz, $CDCl_3$) δ 8.58 (d, *J* = 8.2 Hz, 1H), 8.48 (s, 1H), 8.44 (d, *J* = 8.5 Hz, 1H), 8.10 (dd, *J* = 8.3, 1.4 Hz, 1H), 7.79 (bs, 1H), 7.72 – 7.66 (m, 2H), 7.55 – 7.45 (m, 5H), 7.35 (ddd, *J* = 8.3, 7.1, 1.4 Hz, 1H), 7.31 (dd, *J* = 8.0, 1.3 Hz, 1H), 7.29 – 7.22 (m, 1H), 2.91 (d, *J* = 1.0 Hz, 3H).

^{11}B NMR (128 MHz, $CDCl_3$) δ 36.5.

^{13}C NMR (101 MHz, $CDCl_3$) δ 139.5 (C), 138.8 (C), 138.4 (C), 137.6 (C), 132.0 (C), 131.9 (2xCH), 130.5 (CH), 128.4 (2xCH), 128.2 (CH), 128.1 (CH), 125.7 (CH), 125.5 (CH), 124.8 (CH), 124.2 (CH), 123.6 (C), 121.9 (CH), 121.8 (CH), 118.8 (CH), 20.9 (CH_3).

HRMS (APCI+) m/z: $[M+H]^+$ Calcd for $[C_{23}H_{19}BN]^+$ 320.1605. Found 320.1606.

5,14-Dibora-6,15-dioxa-5,14-dimesityl-9,18-dimethylbenzo[*c*]naphtho[2,1-*k*]tetraphene (11)

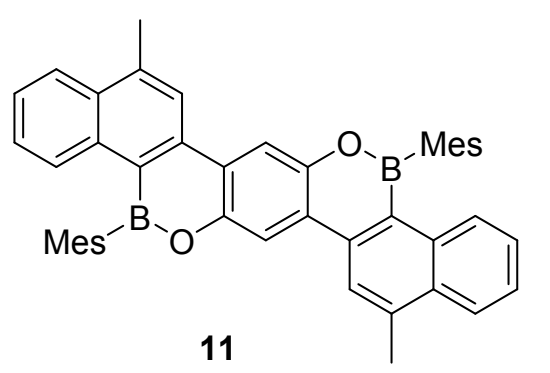

11

Obtained as white powder (22.5 mg, 0.035 mmol, 77%) from **10** (19 mg, 0.045 mmol) following method E with slight modifications (6 equiv BCl_3, $CHCl_3$ 0.12 M for the cyclization; 6 equiv MesMgBr, toluene 0.05 M for the quenching step). R_f = 0.56 (Hexane/AcOEt 94/6). m.p. >275 ºC.

^{1}H NMR (400 MHz, $CDCl_3$) 8.66 (s, 2H), 8.41 – 8.34 (m, 4H), 8.13 (d, *J* = 8.3, 1.3 Hz, 2H), 7.56 (ddd, *J* = 8.1, 6.8, 1.2 Hz, 2H), 7.36 (ddd, *J* = 8.3, 6.7, 1.3 Hz, 2H), 7.02 (s, 4H), 2.91 (s, 6H), 2.45 (s, 6H), 2.22 (s, 12H).

^{11}B NMR (128 MHz, $CDCl_3$) δ 46.1.

^{13}C NMR (101 MHz, $CDCl_3$) δ 148.0 (2xC), 141.5 (2xC), 138.95 (4xC), 138.93 (2xC), 138.2 (2xC), 138.0 (2xC), 132.5 (2xC), 128.02 (4xCH), 127.96 (2xCH), 127.5 (2xCH), 126.4 (2xCH), 124.6 (2xCH), 124.3 (2xC), 121.5 (2xCH), 114.5 (2xCH), 22.3 (4xCH_3), 21.6 (2xCH_3), 21.1 (2xCH_3).

HRMS (APCI+) m/z: $[M+H+MeCN-Mes]^+$ Calcd for $[C_{39}H_{32}B_2NO_2]^+$ 568.2613. Found 568.2607. The mass corresponding to a compound coming from the substitution of the mesityl groups for a MeCN molecule, used in the sample preparation, is observed.

8,17-Dibora-9,18-dioxa-8,17-dimesityl-6,15-dimethylbenzo[*c*]naphtho[2,1-*k*]tetraphene (13)

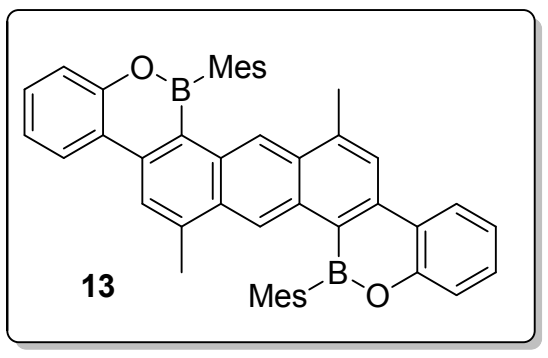

Obtained as pale yellow powder (25.0 mg, 0.039 mmol, 86%) from **12** (19 mg, 0.045 mmol) following method E with slight modifications (6 equiv. BCl_3, $CHCl_3$ 0.12M for the cyclization; 6 equiv. MesMgBr, toluene 0.05 M for the quenching step). R_f = 0.35 (Hexane/AcOEt 97/3). m.p. >275 ºC.

^{1}H NMR (400 MHz, $CDCl_3$) δ 9.18 (s, 2H), 8.43 (dd, *J* = 8.3, 1.5 Hz, 2H), 8.19 (s, 2H), 7.63 (dd, *J* = 8.1, 1.4 Hz, 2H), 7.54 (ddd, *J* = 8.3, 7.0, 1.5 Hz, 2H), 7.42 (ddd, *J* = 8.4, 7.1, 1.4 Hz, 2H), 7.06 (s, 4H), 2.44 (s, 6H), 2.21 (bs, 6H), 2.20 (s, 12H).

^{11}B NMR (128 MHz, $CDCl_3$) δ 44.8.

^{13}C NMR (101 MHz, $CDCl_3$) 152.2 (2xC), 142.2 (2xC), 140.2 (2xC), 138.9 (4xC), 138.2 (2xC), 134.6 (2xC), 131.0 (2xC), 129.1 (2xCH), 128.0 (4xCH), 124.2 (2xCH), 123.8 (2xCH), 123.3 (2xCH), 123.2 (2xC), 120.6 (2xCH), 120.3 (2xCH), 22.1 (4xCH_3), 21.3 (2xCH_3), 19.8 (2xCH_3)

HRMS (APCI+) m/z: $[M+H]^+$ Calcd for $[C_{46}H_{41}B_2O_2]^+$ 647.3287. Found 647.3281.

7-Mesityl-12-(prop-1-en-2-yl)-7,12-dihydroindeno[2,1-*e*]naphtho[1,8-*bc*]borinine (15)

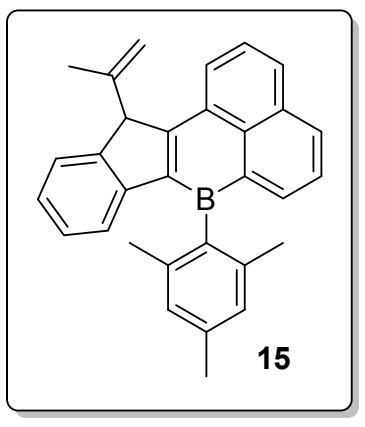

Obtained as orange powder (140.3 mg, 0.342 mmol, 57%) from **14** (169 mg, 0.60 mmol) following method E with slight modifications (BBr_3 instead of BCl_3 is used as the boron electrophile). R_f = 0.15 (Hexane). m.p. 188–189 ºC.

^{1}H NMR (400 MHz, $CDCl_3$) δ 8.69 (dd, *J* = 7.3, 1.2 Hz, 1H), 8.31 (dd, *J* = 6.8, 1.5 Hz, 1H), 8.27 (dd, *J* = 8.1, 1.5 Hz, 1H), 8.10 (dd, *J* = 8.1, 1.2 Hz, 1H), 7.75 – 7.69 (m, 2H), 7.52 (d, *J* = 7.5 Hz, 1H), 7.25 (td, *J* = 7.3, 1.3 Hz, 1H), 7.21 – 7.16 (m, 1H), 7.06 – 7.00 (m, 3H), 5.61 – 5.57 (m, 1H), 5.25 (p, *J* = 1.5 Hz, 1H), 5.16 (s, 1H), 2.51 (s, 3H), 2.14 (s, 3H), 2.10 (s, 3H), 1.28 – 1.24 (m, 3H).

^{11}B NMR (128 MHz, $CDCl_3$) δ 58.2.

^{13}C NMR (101 MHz, $CDCl_3$) δ 162.8 (C), 147.6 (C), 147.2 (C), 146.1 (C), 143.4 (CH), 138.45 (C), 138.43 (C), 136.9 (C), 136.4 (CH), 132.19 (CH), 132.17 (C), 131.4 (C), 130.8 (C), 129.9 (CH), 127.7 (CH), 127.22 (CH), 127.16 (CH), 126.6 (CH), 126.1 (CH), 125.7 (CH), 123.3 (CH), 122.4 (CH), 114.3 (CH_2), 60.0 (CH), 23.3 (CH_3), 23.0 (CH_3), 21.6 (CH_3), 17.5 (CH_3).

HRMS (APCI+) m/z: $[M+H]^+$ Calcd for $[C_{31}H_{28}B]^+$ 411.2276. Found 411.2283.

7-Mesityl-12-methyl-7*H*-dinaphtho[1,8-*bc*:2',1'-*e*]borinine (17)

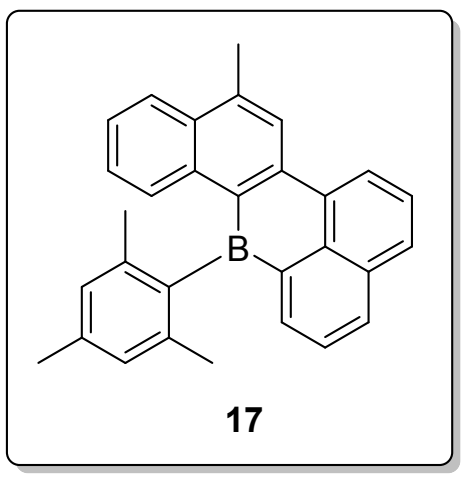

Obtained as yellow powder (127.7 mg, 0.352 mmol, 88%) from **16** (107 mg, 0.40 mmol) following method E with slight modifications (BBr_3 instead of BCl_3 is used as the boron electrophile). R_f = 0.57 (Hexane/AcOEt 97/3). m.p. 206–207 ºC.

^{1}H NMR (400 MHz, $CDCl_3$) δ 8.88 (dt, *J* = 7.3 Hz, 1H), 8.60 (s, 1H), 8.50 (ddd, *J* = 8.7, 1.3, 0.6 Hz, 1H), 8.24 (dd, *J* = 8.2, 1.4 Hz, 1H), 8.11 – 8.06 (m, 2H), 8.04 (dd, *J* = 6.9, 1.5 Hz, 1H), 7.81 (t, *J* = 7.8 Hz, 1H), 7.65

(dd, *J* = 8.0, 6.9 Hz, 1H), 7.49 (ddd, *J* = 8.1, 6.8, 1.2 Hz, 1H), 7.27 (ddd, *J* = 8.5, 6.8, 1.5 Hz, 1H), 7.00 – 6.97 (m, 2H), 2.93 (d, *J* = 1.0 Hz, 3H), 2.47 (s, 3H), 1.97 (s, 6H).

^{11}B NMR (128 MHz, $CDCl_3$) δ 62.2.

^{13}C NMR (101 MHz, $CDCl_3$) δ 143.3 (C), 141.9 (CH), 141.0 (C), 139.5 (C), 137.6 (2xC), 136.5 (C), 135.7 (CH), 133.2 (C), 132.4 (C), 132.2 (C), 130.9 (CH + C), 128.8 (CH), 127.5 (3xCH), 127.0 (CH), 126.7 (CH), 126.2 (CH), 125.8 (CH), 124.2 (CH), 124.0 (CH), 23.2 (2xCH_3), 21.5 (CH_3), 21.2 (CH_3).

HRMS (APCI+) m/z: $[M+H]^+$ Calcd for $[C_{30}H_{26}B]^+$ 397.2122. Found 397.2126.

Comprehensive Structural and Photokinetic Analysis

To establish a fundamental understanding of the relationship between molecular architecture and optical/photophysical behaviors, this section provides a detailed, step-by-step mechanical analysis of the synthesized library. The comprehensive experimental datasets—encompassing steady-state absorption, excitation, and emission profiles, alongside time-resolved fluorescence lifetimes (τ) and deduced kinetic decay constants (k_r and k_{nr})—are systemized herein to map how systematic structural perturbations dictate the electronic properties of these B,X-doped systems.

The analytical breakdown is organized into several structural trends, evaluating the cooperative and individual impacts of core heteroatoms, fusion topologies, peripheral substituents, conformationally flexible rotors, and spatial interactions in core-duplicated macrocycles. All steady-state spectra discussed below are compiled in Figures S6–S9, while the exhaustive photophysical parameters are summarized in Tables S1 and S2.

<u>1. Influence of the Core Heteroatom (N vs. O):</u> comparing the azaborine **5b** and its oxaborine counterpart **4b** reveals that the core heteroatom strongly modulates their photophysical efficiency (Table S1, Figures S6a,b and S7a,b). Both derivatives share nearly identical absorption maxima (~333-337 nm), but the lower electronegativity of nitrogen enhances resonance π-donation, yielding a higher molar extinction coefficient for **5b** (23520 $M^{-1}cm^{-1}$) than for **4b** (19444 $M^{-1}cm^{-1}$). In the excited state, however, the fluorescence quantum yield increases from 0.56 (N) to 0.91 (O). This enhancement is driven by a sixfold drop in the non-radiative rate (k_{nr}), which decreases from 18.3×10^7 s^{-1} to 3.0×10^7 s^{-1}. Structurally, replacing the N–H group with a bivalent oxygen eliminates the high-frequency exocyclic N–H stretching vibration, thereby removing an efficient non-radiative deactivation channel, suppressing thermal dissipation, and maximizing radiative emission. Furthermore, X-ray analysis of the related analogue **4a** confirms a strictly coplanar core in the solid state, which is consistent with a rigid framework lacking large structural distortions and further supports the suppression of non-radiative pathways upon removing the N–H vibration in solution.

5b

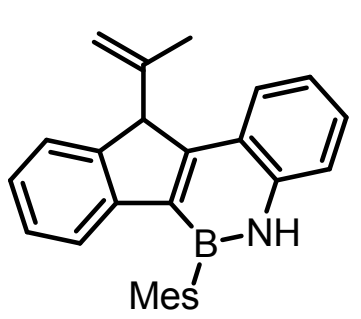

4b

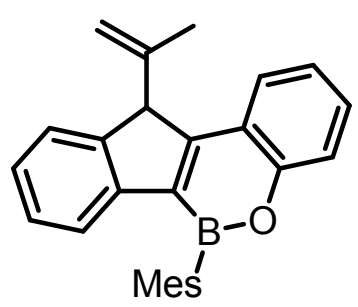

<u>2. Position-dependent π-extension:</u> The impact of the core extension was evaluated by comparing the parent **4b** with the isomers **4c** and **4d**, where the formal addition of a phenyl ring extends the core into a naphthalene-fused system (Table S1, Figures S6b,c and S7b,c). In the ground state, this structural expansion provides significant electronic stabilization through extended π-delocalization across the newly formed polycyclic framework, inducing a clear bathochromic shift in the absorption maxima, moving from 333 nm in **4b** to 338 nm in **4c**, and up to 358 nm in the regioisomeric counterpart **4d**. Extending the framework on the left carbocyclic moiety (**4c**) maintains the absorption at higher energies. In contrast, π-extension on the heteroaromatic right side topology (**4d**) modifies the

4b

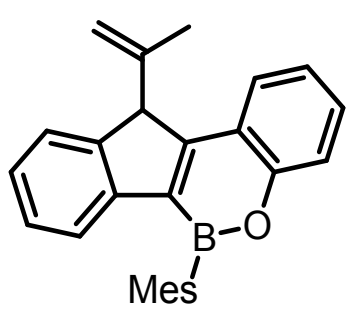

4c

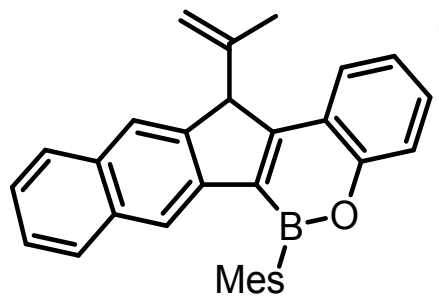

4d

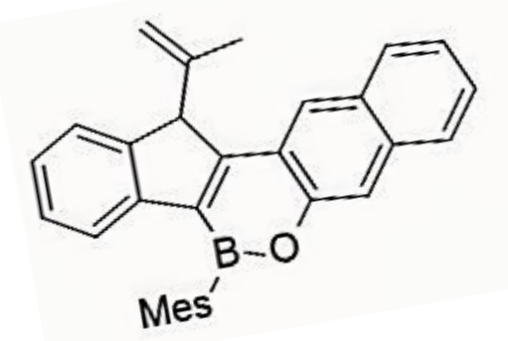

resonance pathways of the extended π-system more effectively; it breaks local orbital symmetry and stabilizes the LUMO, resulting in a more pronounced bathochromic shift of the absorption.

In the excited state (S_1), however, these regiochemical differences lead to a remarkable convergence: despite their different absorption profiles, the emission maxima for both derivatives (**4c** and **4d**) are practically superimposed at the same wavelength (~408 and 412 nm respectively). This behavior demonstrates that **4c** undergoes a much larger Stokes shift due to significant conformational relaxation upon photoexcitation, resulting in a short singlet lifetime (τ=3.6 ns) and a fluorescence quantum yield (ϕ_f) of 0.72. Conversely, the specific substitution pattern in **4d** structurally rigidifies the framework, restricting this excited-state relaxation while nearly doubling the fluorescence lifetime (τ=6.5 ns) and maintaining a high quantum yield (ϕ_f=0.75).

These findings reveal that while site-specific π-extension efficiently shifts ground-state absorption towards lower energies without severely penalizing the emission efficiency, their relaxed emitting states reach a similar energetic minimum. Notably, the parent single-core analogue **4b** exhibits the highest quantum yield of the series (0.91); this high efficiency stems from its smaller, highly symmetric framework, which features a fully allowed emission that maximizes the radiative rate (k_r) while restricting the conformational flexibility and low-frequency skeletal distortions responsible for non-radiative dissipation (k_{nr}) in the larger naphthalene-fused systems.

3. Influence of the Boron Substituent: Within the azaborine sub-family (Table S1, Figures S6a and S7a), the boron substituent significantly modulates both ground-state absorption properties and excited-state efficiencies by balancing steric hindrance and π-conjugation. The unhindered, planar phenyl ring **5c** adopts a coplanar equilibrium geometry in the ground state, which efficiently extends π-conjugation into the core framework, inducing a slight bathochromic shift (~341nm). Conversely, the steric hindrance of the methyl groups in the mesityl derivative **5b** forces the aryl ring into a nonplanar disposition (a steric twist analogous to the distortion observed by analysis of X-ray for related congested azaborine systems like **5d**), decreasing this conjugation and shifting the spectra to slightly higher energies (~337 nm). The aliphatic methyl analogue **5a** exhibits the most blue-shifted profiles (~334 nm).

5a **5b** **5c**

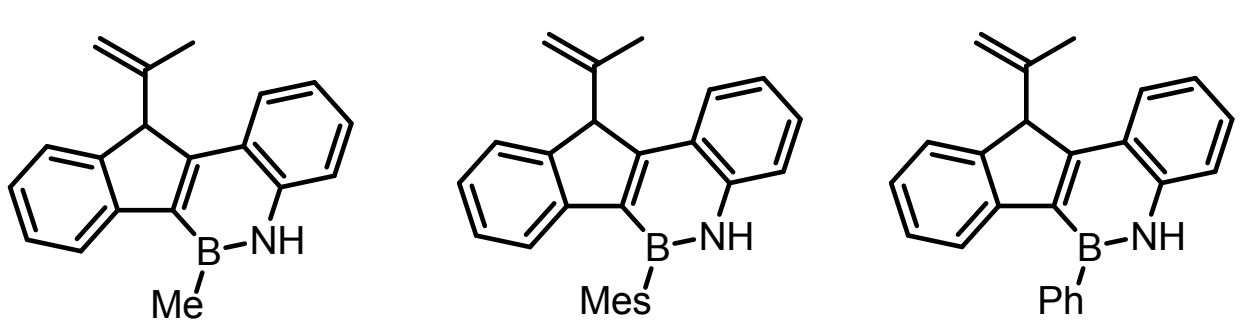

Crucially, this structural trend is reflected in the steady-state emission spectra, where the maxima undergo a progressive bathochromic shift following the **5a** → **5b** → **5c**. Interestingly, despite their structural differences, **5b** and **5c** exhibit nearly identical fluorescence quantum yields (0.56 and 0.59 respectively) and decay lifetimes (both 2.4 ns) which translates into perfectly matched radiative (k_r) and non-radiative (k_{nr}) rate constants. This kinetic coincidence arises because both derivatives suffer from distinct, yet equally penalizing, non-radiative deactivation pathways in the S_1 state. For **5c**, the absence of bulky *ortho*-methyl results in a very low rotational barrier; this allows the phenyl ring to undergo dynamic, out-of-plane torsional motions upon photoexcitation, dissipating energy through non-radiative internal conversion. In contrast, the bulky

mesityl group in **5b** remains sterically constrained but its peripheral methyl groups act as active vibrational sinks. Remarkably, replacing these aromatic units with a compact methyl group (**5a**) eliminates both the torsional motions and the additional vibrational sinks, drastically reducing the non-radiative rate (k_{nr}) by half and yielding the highest ϕ_f of the series (0.78).

<u>4. Influence of the Exocyclic Diphenylmethylene Molecular Rotor:</u> Replacing the isopropenyl group with a diphenylmethylene unit, yielding a benzofulvene-type framework, dramatically alters both ground-state optics and excited-state relaxation. In the azaborine series (**5b** vs. **5d**) (Table S1 and Figures S6a,d and S7a,d) the extended π-conjugation induces a pronounced bathochromic shift in both the absorption (337 nm to 383 nm) and in the emission (375 nm to 462 nm) spectra but is also accompanied by a near-total collapse in fluorescence efficiency (from 0.56 to 0.01). Although X-ray analysis of **5d** confirms that the two exocyclic phenyl rings are twisted out of plane due to steric hindrance, the ground-state framework still gains a net expansion of the π-system that accounts for this red-shifted absorption.

5b

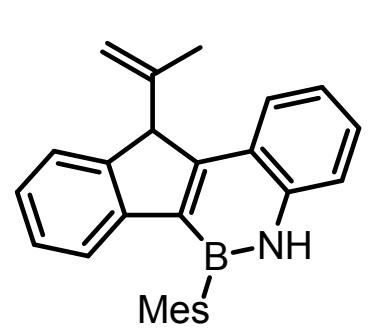

5d

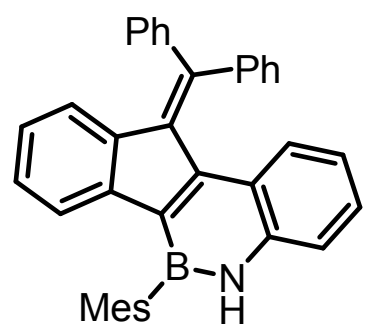

4a

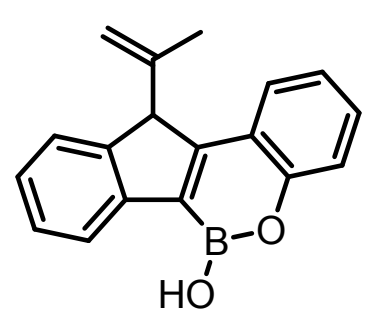

4e

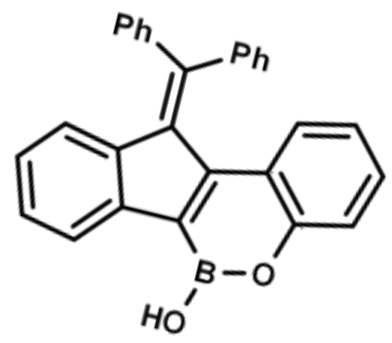

Crucially, photoexcitation to the S_1 state reduces the π-bond character of the exocyclic double bond. In solution this pre-existing distortion combined with a lowered rotational barrier in S_1 causes rapid out-of-plane torsional motions; this molecular rotor action drains the remaining energy through non-radiative pathways, suppressing fluorescence. We observed a similar red-shift and a complete loss of fluorescence in the oxaborine analogues (**4a** vs. **4e**) (Table S1 and Figures S6b,d and S7b). Here, the lack of a mesityl group leaves the boron center exposed. This exposure, combined with the high rotational flexibility of the exocyclic phenyl rings, drives the excited framework towards instantaneous thermal dissipation, resulting in a complete absence of fluorescence.

<u>5. Expansion of the Core Heterocycle (5- vs. 6-Membered Rings):</u> The specific impact of the central heterocycle size on the photophysical properties was evaluated by comparing the 5-membered oxaborine derivatives with their expanded 6-membered counterparts, specifically the **4a** vs. **8a** and **4b** vs. **8c** pairs (Tables S1 and S2 and Figures S6b, S7b, S8a and S9a). In the ground state, moving from 5-membered to the 6-membered analogues induces a prominent hypsochromic shift in the absorption maxima (26-28 nm) along with a decrease in the molar extinction coefficients. As supported by X-ray data (which show that the 6-membered core remains essentially coplanar in the solid state), this pronounced blue-shift is not driven by a ground-state structural distortion. Instead, it stems from a more localized π-electron distribution within the expanded six-membered ring, which widens the HOMO–LUMO energy gap.

4a

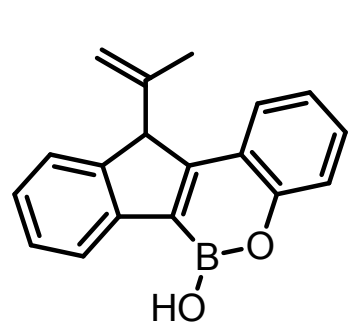

8a

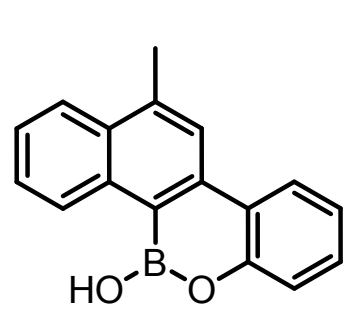

4b

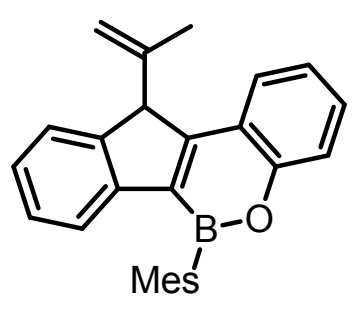

8c

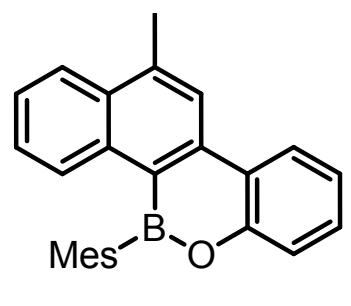

Similarly, the steady-state emission spectra of the six-membered analogues (Figure S8a, S9a) show a significant blue-shift compared to their five-membered precursors, confirming that this widened energy gap persists in the relaxed excited state (S_1). Concurrently, this core expansion leads to a severe drop in the ϕ_f for both series, though driven by completely distinct kinetic mechanisms. For the hydroxyl derivatives (**4a** and **8a**), the drop in ϕ_f from 0.66 to 0.40 is caused by a drastic decrease of the radiative rate (k_r) (from 26.4×10^7 to 3.5×10^7 s^{-1}). Because the electronic transition loses its fully allowed character, the emission slows down, forcing a longer singlet lifetime (τ) (from 2.5 to 11.4 ns). Conversely, the ϕ_f of the mesityl derivatives (**4b** and **8c**) collapses down from 0.91 to 0.20 due to a different pathway. Here, we observe a pronounced, sixfold increase in the non-radiative decay (k_{nr}) from 3.0×10^{7}s^{-1} to 17.0×10^{7}s^{-1}. In this case, building upon the vibrational deactivation pathways discussed for the mesityl series in Section 3, the combination of the bulky mesityl group and increased dynamic flexibility of the six-membered ring in the excited state activates fast non-radiative pathways that rapidly release the excited-state energy.

6. Interplay of Core Size (5- vs. 6-Membered Rings), Boron Substitution, and Peripheral Conjugation in Oxaborines: The combined effect of heterocycle size (5- vs. 6-membered rings) and the boron substituent (-OH vs. -Mes) reveals a distinct interplay between conformational flexibility and steric shielding (Tables S1 and S2, and Figures S6a, S7a, S8b and S9b). In the ground state, the 5-membered derivatives **4a** and **4b** share identical absorption maxima (333 nm) but the mesityl in **4b** more than doubles ε value. Moving to the six-membered analogues (**8d** and **8e**) induces a general hypsochromic shift toward ~300 nm. Rather than reflecting ground-state structural distortion, this blue-shift stems from the electronic nature of the expanded core; expanding the central heterocycle alters the orbital contributions and widens the HOMO–LUMO gap. Within this blue-shifted series, peripheral phenyl conjugation controls transition intensities. In the hydroxyl derivative **8d**, peripheral phenyl alignment maximizes the transition dipole moment, yielding a high ε (21,325 M^{-1}cm^{-1}). Conversely, severe steric hindrance from the mesityl group in **8e** distorts the flexible six-membered core and twists the peripheral phenyl ring out of plane. This structural deformation breaks electronic delocalization, collapsing ε to 5,720 M^{-1}cm^{-1}.

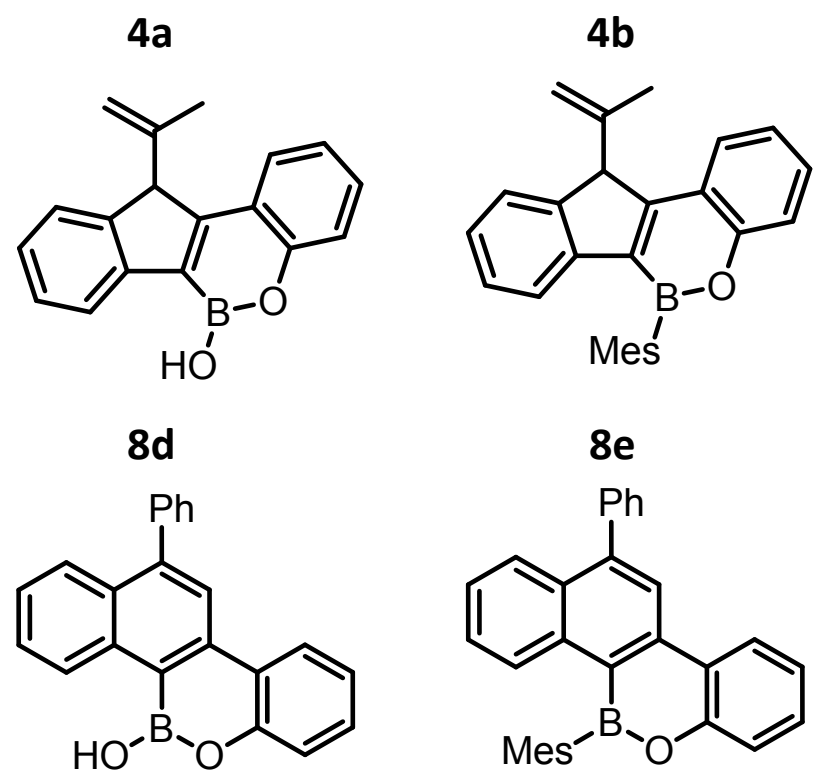

In the excited state (S_1), the rigid 5-membered framework allows the mesityl group to increase ϕ_f from 0.66 (**4a**) to 0.91 (**4b**) by effectively suppressing non-radiative decay. Moving to the more flexible 6-membered analogues minimize this rigidity-driven enhancement. While **8d** and **8e** exhibit similar ϕ_f values (0.34 and 0.37), their kinetic balances contrast sharply. The -OH analogue (**8d**) exhibits a prolonged singlet lifetime (τ=7.9 ns) due to a slow radiative rate (k_r= 4.3×10^7 s^{-1}). In **8e**, the mesityl group doubles k_r (9.3×10^7 s^{-1}), shortening τ to 4.0 ns; however, this radiative advantage is cancelled by increased non-radiative pathways driven by low-frequency skeletal motions of the bulky mesityl group and local steric hindrance within the looser core.

Evaluating the two series vertically confirms that expanding the central heterocycle systematically penalizes emission efficiency. The transition from five- to six-membered rings introduces significant dynamic flexibility in the excited state. This lowers ϕ_f significantly in the hydroxyl series, and even more severely in the mesityl derivatives. In both cases, the larger central ring introduces low-frequency vibrational modes that accelerate thermal dissipation to the solvent, ultimately dominating the excited-state relaxation.

7. From Single B–O to Double B–O-containing Systems: Positional Isomerism in Coupled Oxaborines: The photophysical profiles of the core-duplicated isomers, **11** and **13**, highlight how positional isomerism drives ground-state configuration and excited-state kinetics when compared to the monomeric precursor **8c** (Table S2 and Figures S8a,d and S9a,d). In the ground state, both dimers display resolved vibronic features corresponding to their lowest-energy $S_0 \rightarrow S_1$ ($\pi \rightarrow \pi^*$) transition. For **11**, where the oxygen atoms face inward, the extended and highly coplanar framework yields a fully allowed transition. This structure results in a well-resolved vibronic absorption profile with sharp maxima at 380, 362, and 345 nm with absorption maximum at 380 nm with a high molar extinction coefficient (ε= 59,930 $M^{-1}cm^{-1}$) at this lowest energy peak (Figure S8d). Conversely, the outward orientation of the oxygen atoms in **13** induces severe steric clashes between the bulky mesityl groups. This geometric distortion breaks the ideal orbital overlap and lowers the local symmetry, rendering its fundamental $S_0 \rightarrow S_1$ transition less allowed. This electronic decoupling causes the lowest-energy transition to appear as a series of low-intensity bands at around 397 nm (ε= 11,290 $M^{-1}cm^{-1}$), a value that closely resembles that of the **8c** (ε= 12,075 $M^{-1}cm^{-1}$). Instead, the ground-state spectrum of **13** is dominated by a pronounced, high-energy absorption band centered in the UV region at 325 nm (ε= 66,430 $M^{-1}cm^{-1}$) which represents a highly allowed transition to an upper electronic state ($S_0 \rightarrow S_2$) stabilized by the twisted non-planar conformation of the interacting chromophores.

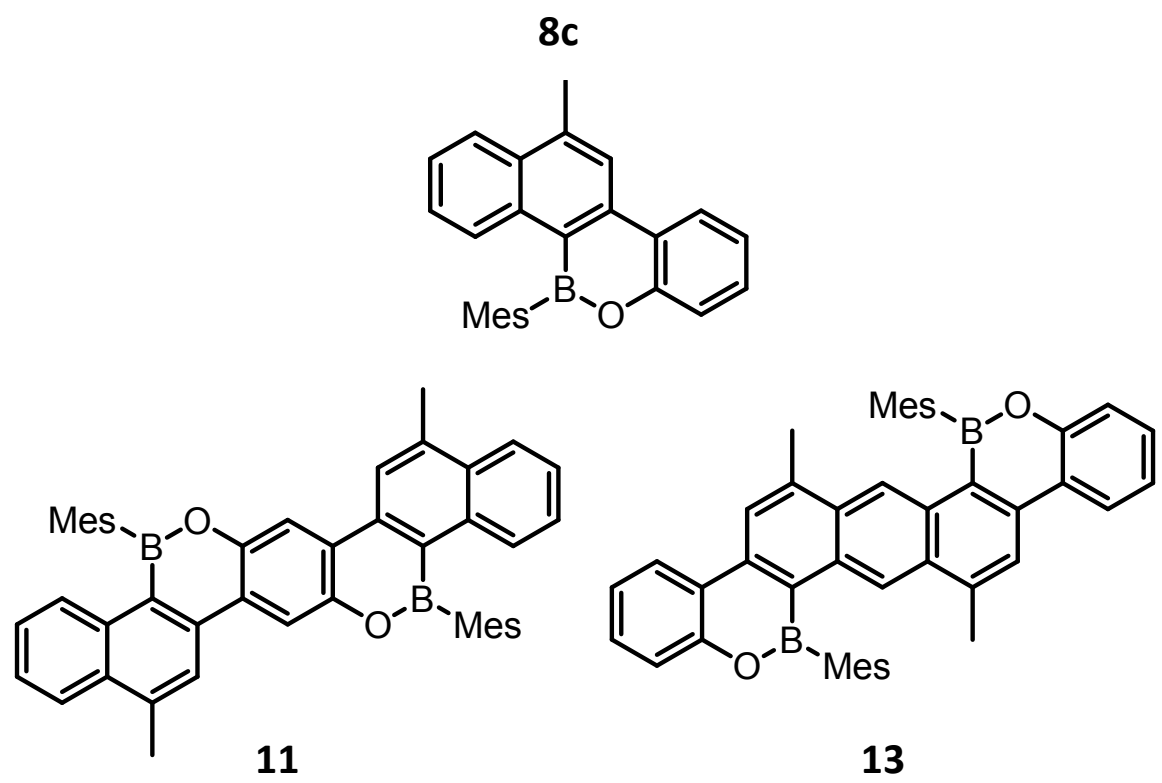

From the first singlet excited state (S_1), the symmetry of this emitting state seems to control the deactivation kinetics. For **11**, the fully allowed nature of the coplanar S_1 state drives an exceptionally fast radiative rate (k_r =70.0×10^7 s^{-1}) and effectively suppresses non-radiative decay down to k_{nr} = 6.9×10^7 s^{-1}, yielding a high fluorescence quantum yield of 0.91 with τ = 1.3 ns. On the other hand, because fluorescence in **13** originates from the symmetry-forbidden S_1 state (emitting a structured band with the maximum located at 425 nm), its radiative deactivation is hindered, dropping k_r to 12.1×10^7 s^{-1}. Concurrently, the structural flexibility of this outward configuration allows low-frequency vibrations to easily compete with the slow emission, causing the non-radiative decay to remain high (k_{nr}=17.4×10^7 s^{-1}) and nearly identical to the monomeric precursor **8c** (k_{nr} = 17.0×10^7 s^{-1}). This kinetic balance limits the final emission efficiency of **13** isomer to a ϕ_f for the emission of 0.41 with a prolonged τ of 3.4 ns.

<u>8. Photophysical Behavior of Oxygen-Free Expanded Analogues (15 and 17)</u>. Beyond the oxygen-containing oxaborine cores, removing the heteroatom oxygen yields a specialized family of oxygen-free B-doped polycyclic aromatic hydrocarbons whose optical and photophysical properties are heavily shifted toward the red region. A detailed analysis of their ground-state absorption profiles reveals a pronounced bathochromic shift, showing that compound **15** absorbs at 349 nm, whereas **17** exhibits a low-energy absorption maximum at 399 nm.

15 **17**

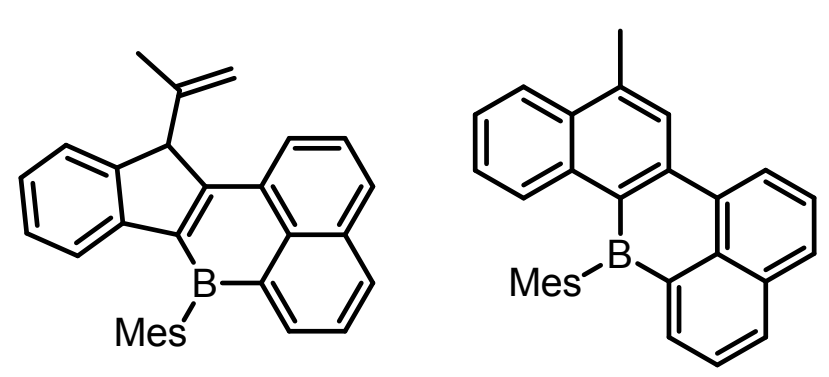

This red-shifted behavior extends to their excited-state properties, as exemplified by emission maximums in the visible spectrum at 542 nm for **15** and 480 nm for **17**, while maintaining comparable fluorescence quantum yields (0.19 and 0.17, respectively). Furthermore, both compounds display remarkably large Stokes shifts (particularly significant in **15**), reflecting substantial structural relaxation, geometric reorganization, and conformational shifts upon photoexcitation.

In the case of compound **17**, X-ray analysis provides vital structural insights into its ground-state geometry in the solid state, revealing a significant deviation from core planarity driven by severe steric congestion between the mesityl substituent and the fjord-region hydrogen atoms. Although crystal data for **15** are not available—making it difficult to ascertain whether an analogous solid-state distortion is present in both derivatives—this structural feature in **17** illustrates a conformational flexibility that can undergo further adaptation in solution and upon photoexcitation. While their kinetic deactivation parameters remain nearly identical, this structural distortion directly affects the HOMO–LUMO energy gap and electronic structure, explaining the differences in their absorption and emission maxima.

Table S1. Photophysical properties and decay kinetics of B-X-doped benzo[*a*]fluorene derivatives.

Compd.	$\lambda_{abs,max}$(nm)[a]	ε($M^{-1}cm^{-1}$)[b]	λ_{em} (nm)[c]	ϕ_f[d]	τ (ns)[e]	k_r (10^7 s^{-1})[f]	k_{nr} (10^7 s^{-1})[f]	τ^0 (ns)[g]
**4a**	333 (310)	8640±85	356/**371**/387(s)	0.66	2.5	26.4	13.6	3.8
**4b**	333 (320)	19444±110	368(s)/**385**/402(s)	0.91	3.0	30.3	3.0	3.3
**4c**	338 (335)	25265±625	387(s)/**408**/434(s)	0.72	3.6	20.0	7.8	5.0
**4d**	358 (355)	14450±60	393/**412**/435(s)	0.75	6.5	11.5	3.9	8.7
**4e**	384(380)	4780±80	---	0.00	---	----	---	---
**5a**	334 (330)	8220±220	356/**373**/388(s)	0.78	2.3	33.9	9.6	2.9
**5b**	337 (320)	23520±990	355/**375**/393(s)	0.56	2.4	23.3	18.3	4.3
**5c**	341 (320)	14350±40	365/**381**/399(s)	0.59	2.4	24.6	17.1	4.1
**5d**	383(390)	9650±30	445(s)/**462**	0.01 [h]	6.9 [i]	0.1	14.4	766.7
**15**	349(350)	9480±140	**542**	0.19 [h]	13.7 [i]	1.4	5.9	72.1

(a) Lowest-energy experimental absorption maximum $\lambda_{abs,max;}$ values in parentheses correspond to λ_{exc} used for monitoring the emission spectra. (b) Molar extinction coefficients (ε) determined at $\lambda_{abs,max}$. (c) Emission maxima λ_{em}; (s) denotes a shoulder in the vibronic profile, values in bold indicate the $\lambda_{em,max}$ selected for monitoring the excitation spectra. (d) Fluorescence quantum yield ϕ_f, determined using 9,10-diphenylanthracene (perylene) **[h]** in cyclohexane as a standard.[11] (e) Singlet excited-state lifetimes (τ) recorded by time-correlated single-photon counting (TCSPC); All decay profiles were fitted to a monoexponential model following excitation with a NanoLED (Horiba) emitting at 295 nm (370 nm) **[i]**. (f) Radiative k_r (=ϕ_f/τ) and non-radiative k_{nr} (=(1-ϕ_f)/τ) decay rate constants calculated assuming a unitary deactivation from the S_1 state. (g) Natural or radiative lifetime τ^0 (=1/k_r). Measurements were performed in dilute solutions of dichloromethane (DCM) at 25ºC.

Table S2. Photophysical properties and decay kinetics of B-X-doped chrysene derivatives.

Compd.	$\lambda_{abs,max}$(nm)[a]	ε($M^{-1}cm^{-1}$)	λ_{em} (nm)[b]	ϕ_f[c]	τ (ns)[d]	k_r (10^7 s^{-1})[f]	k_{nr} (10^7 s^{-1})[f]	τ^0 (ns)[g]
**8a**	307(320)	4670±170	**347**/365/383	0.40	11.4	3.5	5.3	28.5
**8c**	305(320)	12075±190	354/**370**/387(s)	0.20	4.7	4.3	17.0	23.5
**8d**	299(320)	21325±960	356/**372**/387(s)	0.34	7.9	4.3	8.4	23.2
**8e**	305(320)	5720±15	365(s)/**383**/400(s)	0.37	4.0	9.3	15.8	10.8
**9a**	314(320)	7160±260	363/**378**/395(s)	0.40	4.3	9.3	13.9	10.8
**17**	399(400)	18780±350	**480**	0.17 [h]	11.4	1.5	7.3	67.1
**11**	380 (355)	59930±155	**385**/405/427/453(s)	0.91	1.3	70.0	6.9	1.4
**13**	397	11290±120	**425**/452/478/516(s)	0.41 [h]	3.4 [i]	12.1	17.4	8.3

(a) Lowest-energy experimental absorption maximum $\lambda_{abs,max;}$ values in parentheses correspond to λ_{exc} used for monitoring the emission spectra. (b) Molar extinction coefficients (ε) determined at $\lambda_{abs,max}$. (c) Emission maxima λ_{em}; (s) denotes a shoulder in the vibronic profile, values in bold indicate the $\lambda_{em,max}$ selected for monitoring the excitation spectra. (d) Fluorescence quantum yield ϕ_f, determined using 9,10-diphenylanthracene (perylene) **[h]** in cyclohexane as a standard.[11] (e) Singlet excited-state lifetimes (τ) recorded by time-correlated single-photon counting (TCSPC); All decay profiles were fitted to a monoexponential model following excitation with a NanoLED (Horiba) emitting at 295 nm (370 nm) **[i]**. (f) Radiative k_r (=ϕ_f/τ) and non-radiative k_{nr} (=(1-ϕ_f)/τ) decay rate constants calculated assuming a unitary deactivation from the S_1 state. (g) Natural or radiative lifetime τ^0 (=1/k_r). Measurements were performed in dilute solutions of dichloromethane (DCM) at 25ºC.

[11] A. M. Brouwer, *Pure Appl. Chem.* **2011**, *83*, 2213–2228.

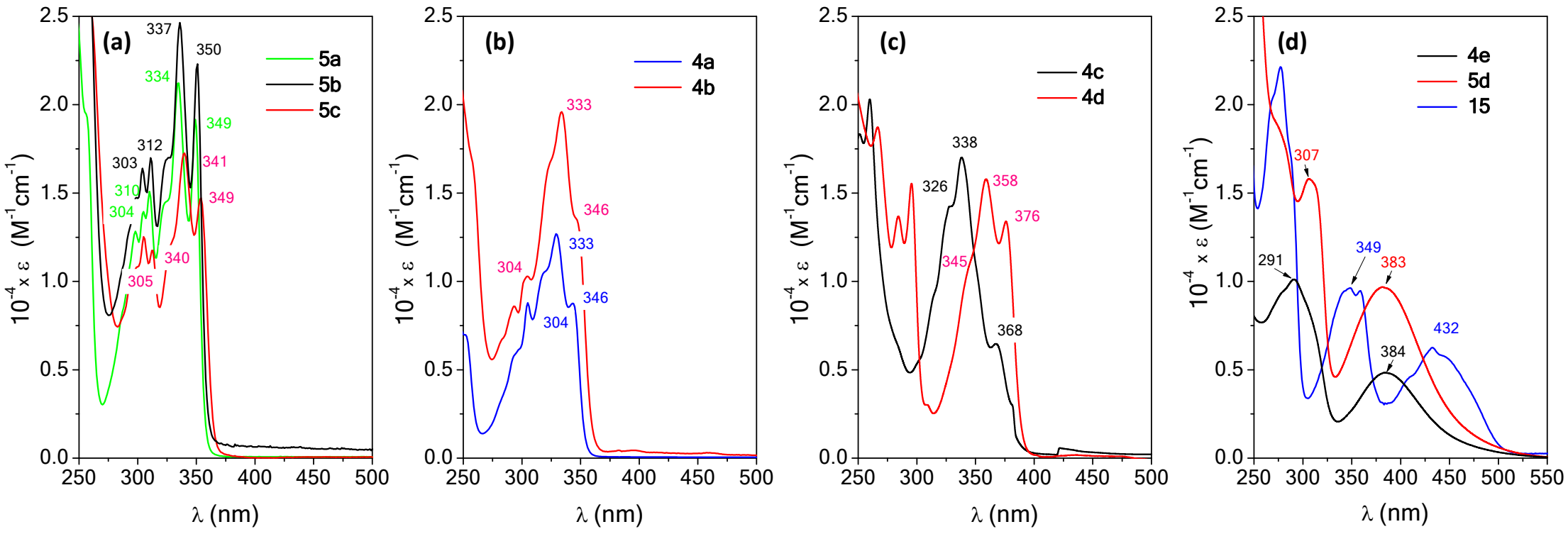

Figure S5. Absorption spectra (as molar absorptivity, ε, $M^{-1}cm^{-1}$ vs wavelength) of representative 5-membered B–X-doped benzo[a]fluorene-based heterocycles in dilute solutions of DCM at 25 ºC temperature: panel (**a**) displays the effect of the exocyclic boron-substituents (–Me, –Mes, and –Ph) within the azaborine subfamily (**5a**, **5b**, and **5c**); panel (**b**) illustrates the effect of the exocyclic boron-substituents (–OH and –Mes) within the oxaborine (**4a** and **4b**) frameworks; panel (**c**) illustrates the impact of ring-fusion topology and angular extension on the lowest-energy electronic transitions for benzo[a]fluorenes **4c** and **4d**; and panel (**d**) showcases the exocyclic diphenyl rotor-containing frameworks (**4e** and **5d**) alongside the naphthalene-fused derivative **15**.

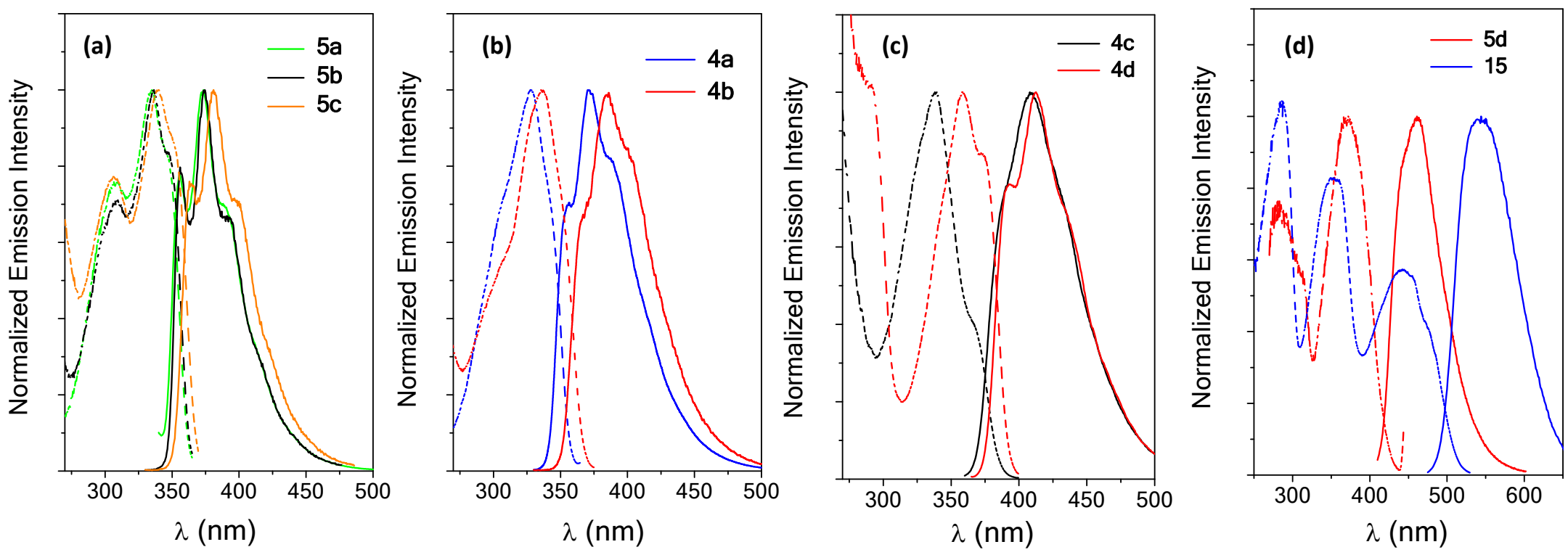

Figure S6. Normalized emission spectra upon excitation at λ_{exc} and excitation spectra (upon $\lambda_{em,max}$ emission wavelength) of representative 5-membered B–X-doped benzo[*a*]fluorene-based heterocycles in dilute solutions of DCM at 25 ºC temperature: panel (**a**) displays the effect of the exocyclic boron-substituents (–Me, –Mes, and –Ph) within the azaborine subfamily (**5a**, **5b**, and **5c**); panel (**b**) illustrates the effect of the exocyclic boron-substituents (–OH and –Mes) within the oxaborine (**4a** and **4b**) frameworks; panel (**c**) illustrates the impact of ring-fusion topology and angular extension on the lowest-energy electronic transitions for benzo[*a*]fluorenes **4c** and **4d**; and panel (**d**) showcases the exocyclic diphenyl rotor-containing frameworks (**4e** and **5d**) alongside the naphthalene-fused derivative **15**.

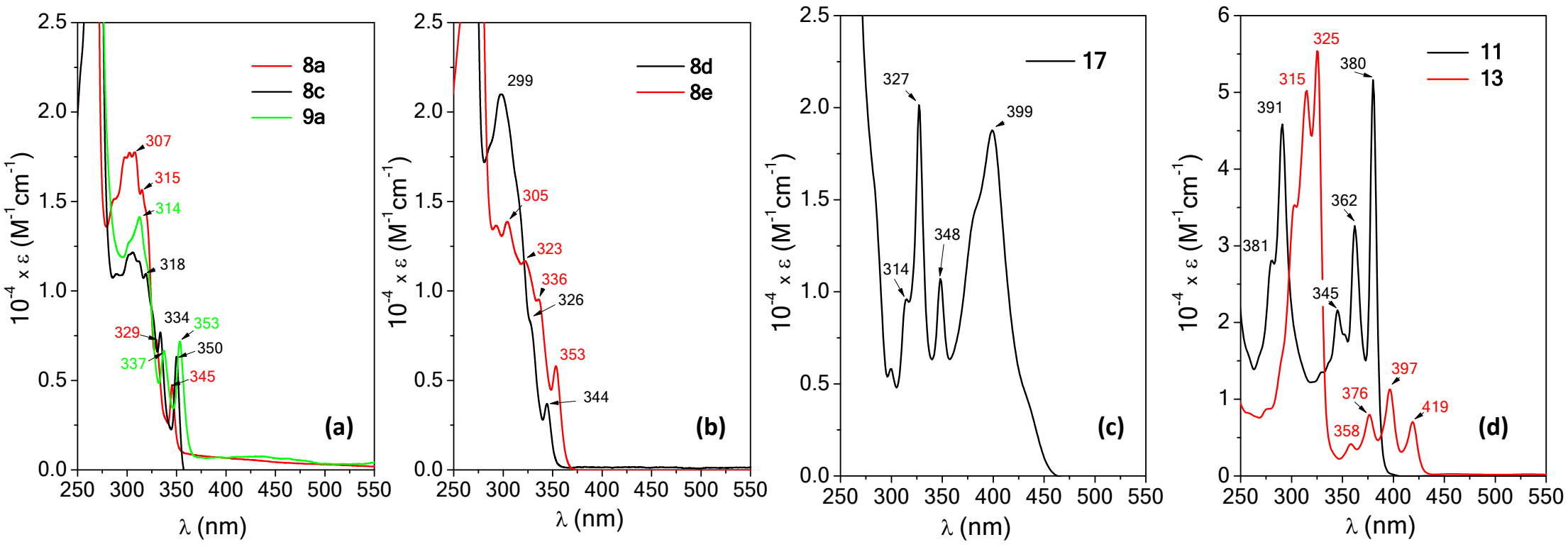

Figure S7. Absorption spectra (as molar absorptivity, ε, $M^{-1}cm^{-1}$ vs wavelength) of representative 6-membered B–X-doped chrysene-based heterocycles, naphthalene-fused extensions, and dimeric macrocycles recorded in dilute solutions of DCM at 25 ºC: panel **(a)** displays the absorption profiling within the chrysene-based subfamily, comparing oxaborines (**8a** and **8c**) and azaborine (**9a**) frameworks to illustrate the impact of core heteroatoms and exocyclic boron-substituents; panel **(b)** illustrates the ground-state absorption behavior of α-phenyl-substituted oxaborines (**8d** and **8e**); panel **(c)** showcases the naphthalene-fused derivative **17**, highlighting the retention of high-energy transitions alongside the appearance of an intense, distinct low-energy band approaching the visible edge due to core linear polycyclic extension; and panel **(d)** demonstrates the dramatic contrast in electronic transition probabilities and spatial orientation between the macrocyclic positional regioisomers **11** and **13**, highlighting the profound effect of intramolecular exciton coupling on the resulting molar extinction coefficients.

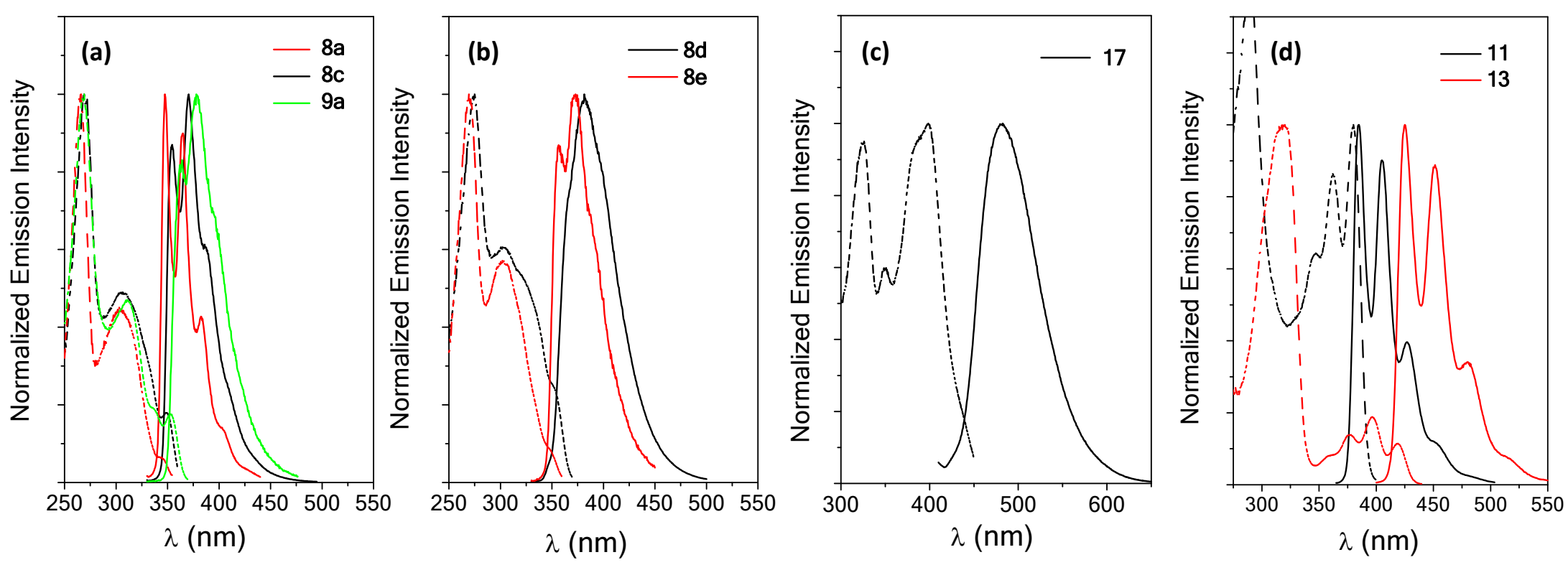

Figure S8. Normalized fluorescence emission (solid lines) and excitation (dashed lines) spectra of representative 6-membered B–X-doped chrysene-based heterocycles, naphthalene-fused extensions, and dimeric macrocycles recorded in dilute solutions of DCM at 25 ºC. Panel **(a)** displays the excitation and emission profiles within the chrysene-based series, comparing oxaborines (**8a** and **8c**) and azaborine (**9a**) frameworks to demonstrate the impact of core heteroatoms and exocyclic boron-substituents; panel **(b)** illustrates the photophysical behavior of α-phenyl-substituted oxaborines (**8d** and **8e**); panel **(c)** showcases the naphthalene-fused derivative **17**, highlighting the loss of structured profiling and the pronounced bathochromic shift associated with linear extension and active excited-state relaxation; and panel **(d)** highlights the contrast between the macrocyclic positional regioisomers **11** and **13** demonstrating the influence of internal geometry on the resulting intramolecular exciton coupling.

X-ray diffraction data for 4a, 5d, 8a and 17

Single crystals from compound **4a** obtained by slow evaporation of a saturated acetone solution were coated in mineral oil and X-ray intensity were collected at 200 K on a Bruker-Nonius KappaCCD diffractometer equipped with graphite-monochromated Mo Kα radiation (λ = 0.71073 Å) and an Oxford Cryostream 700 unit. The structure was solved with ShelXT (intrinsic phasing) and refined by ShelXL (least square minimization) in Olex2.

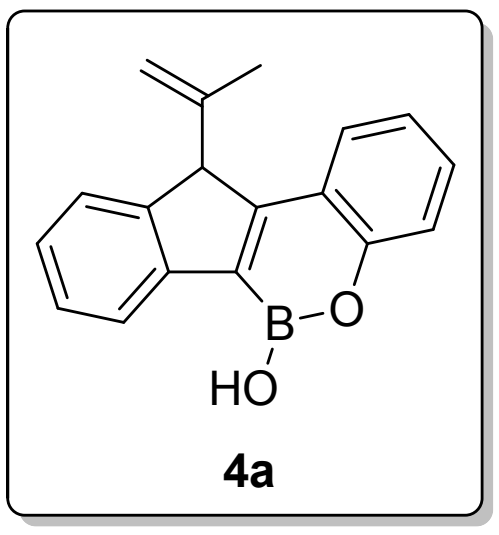

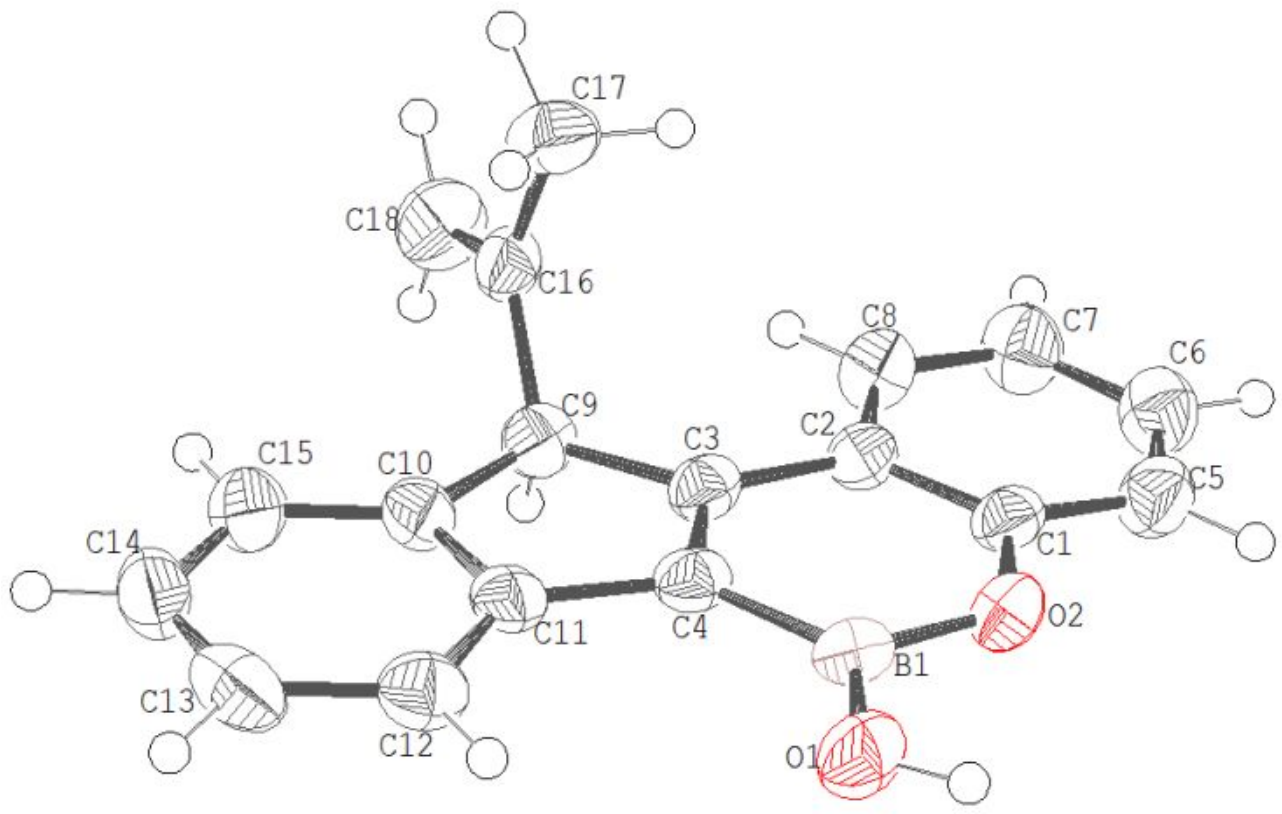

Figure S9. ORTEP plot of **4a** with 50% ellipsoids.

Table S3. Crystal data and structure refinement for compound **4a**

Deposition number	2575355	
Identification code	**4a**	
Empirical formula	$C_{18} H_{15} B O_2$	
Formula weight	274.11	
Temperature [K]	200.00	
Wavelength [Å]	0.71073	
Crystal system	monoclinic	
Crystal habit	colourless block	
Space Group	P 2_1/c	
Unit cell dimensions	a = 11.7455(7) Å	α = 90 º
	b = 6.1968(3) Å	β = 90.979(2)º
	c = 19.3649(9) Å	γ = 90º
Volume [Å^3]	1409.26(13)	
Z	4	

Density (calculated) [g cm^{-3}]	1.292
Absorption Coefficient [mm^{-1}]	0.082
F(000)	576
Cristal size [mm^{3}]	0.264 x 0.19 x 0.13
θ range for data collection	3.452 to 25.019 º
Index ranges	-13 <= h <= 11, -7 <= k <= 7, -23 <= l <= 23
Reflection collected	19888
Independent reflections	2490 [R(int) = 0.0688]
Reflections [I>2σ(I)]	1885
Absorption correction	multi-scan
Max. and min. transmission	0.7461 and 0.6929
Refinement method	full y Fsqd
Data / Restraints / Parameters	2490 / 0 / 192
Goodness-of-fit on F^{2}	1.065
Final R indices [I>2σ(I)]	R1 = 0.0551, wR2 = _0.1342
R indices (all data)	R1 = 0.0774, wR2 = 0.1474
Largest diff. peak and hole [e. Å^{-3}]	0.384 and -0.176

Single crystals from compound **5d** obtained by slow evaporation of a saturated hexane solution were coated in mineral oil and X-ray intensity were collected at 200 K on a Bruker-Nonius KappaCCD diffractometer equipped with graphite-monochromated Mo Kα radiation (λ = 0.71073 Å) and an Oxford Cryostream 700 unit. The structure was solved with ShelXT (intrinsic phasing) and refined by ShelXL (least square minimization) in Olex2.

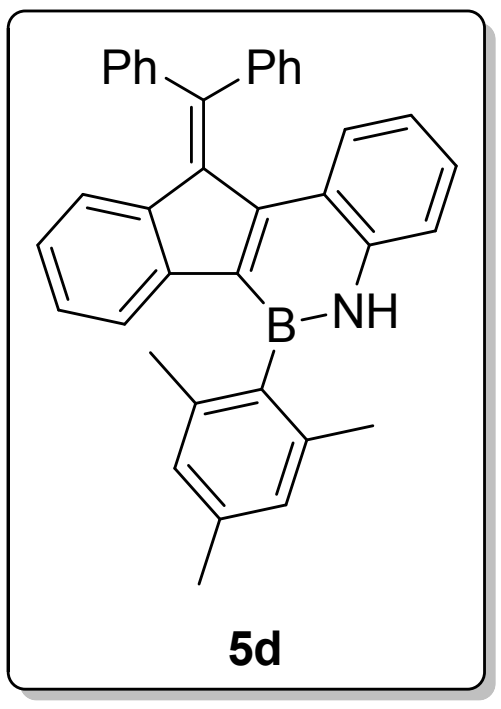

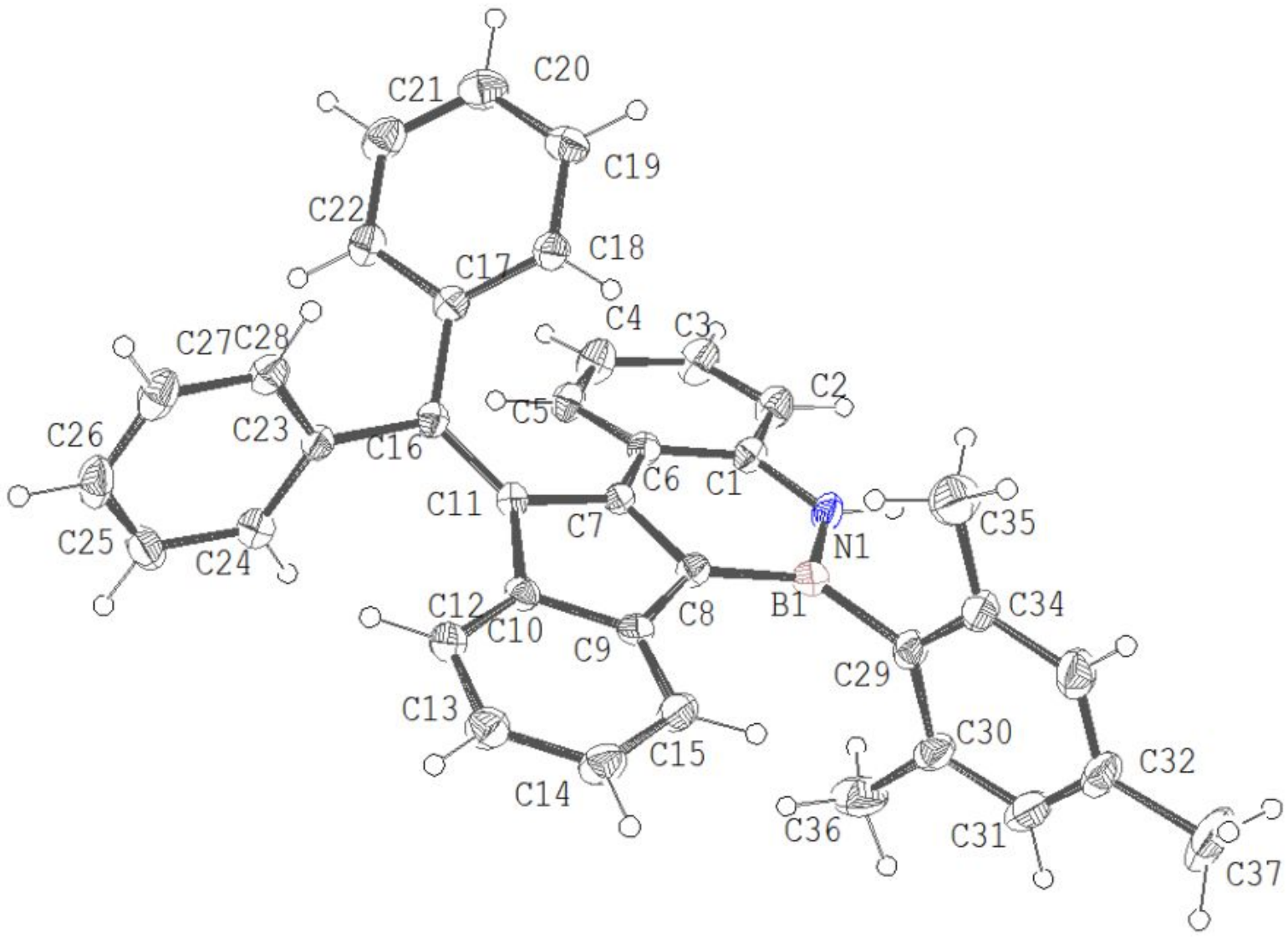

Figure S10. ORTEP plot of **5d** with 30% ellipsoids.

Table S4. Crystal data and structure refinement for compound **(5d)**

Deposition number	2575422	
Identification code	**5d**	
Empirical formula	C_{37} H_{30} BN	
Formula weight	499.43	
Temperature [K]	200	
Wavelength [Å]	0.71073	
Crystal system	monoclinic	
Crystal habit	red plate	
Space Group	P $2_12_12_1$	
Unit cell dimensions	a = 10.0664(3) Å	α = 90 º
	b = 12.0643(4) Å	β = 90 º
	c = 23.2448 (10)Å	γ = 90 º
Volume [Å3]	1249.66(13)	

Z	4
Density (calculated) [g cm^{-3}]	1.175
Absorption Coefficient [mm^{-1}]	0.067
F(000)	1056
Cristal size [mm^3]	0.278 x 0.226 x 0.101
Diffractometer	κ-geometry diffractometer
Radiation source	MoKα
θ range for data collection	3.506 to 25.017 º
Index ranges	-10 <= h <= 11, -14 <= k <= 14, -27 <= l <= 27
Reflection collected	34592
Independent reflections	4975 [R(int) = 0.0712]
Reflections [I>2σ(I)]	4116
Completeness to θ = 25.017 º	0.996
Absorption correction	multi-scan
Max. and min. transmission	0.7456 and 0.6977
Refinement method	full y Fsqd
Data / Restraints / Parameters	4975 / 0 / 355
Goodness-of-fit on F^2	1.090
Final R indices [I>2σ(I)]	R1 = 0.0445, wR2 = 0.0865
R indices (all data)	R1 = 0.0595, wR2 = 0.0923
Flack parameter configuration)	-0.7 (10) (insufficient for determining absolute
Largest diff. peak and hole [e. Å^{-3}]	0.135 and -0.157

Single crystals from compound **8a** obtained by slow evaporation of a saturated acetone solution were coated in mineral oil and X-ray intensity were collected at 200 K on a Bruker-Nonius KappaCCD diffractometer equipped with graphite-monochromated Mo Kα radiation (λ = 0.71073 Å) and an Oxford Cryostream 700 unit. The structure was solved with ShelXT (intrinsic phasing) and refined by ShelXL (least square minimization) in Olex2.

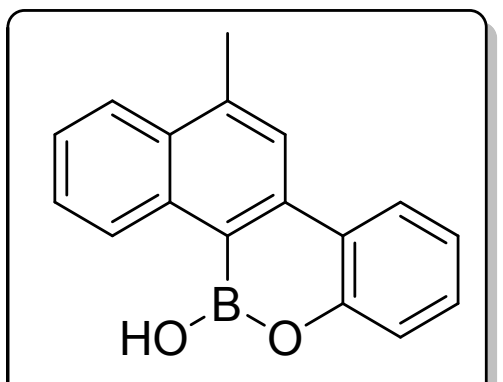

8a

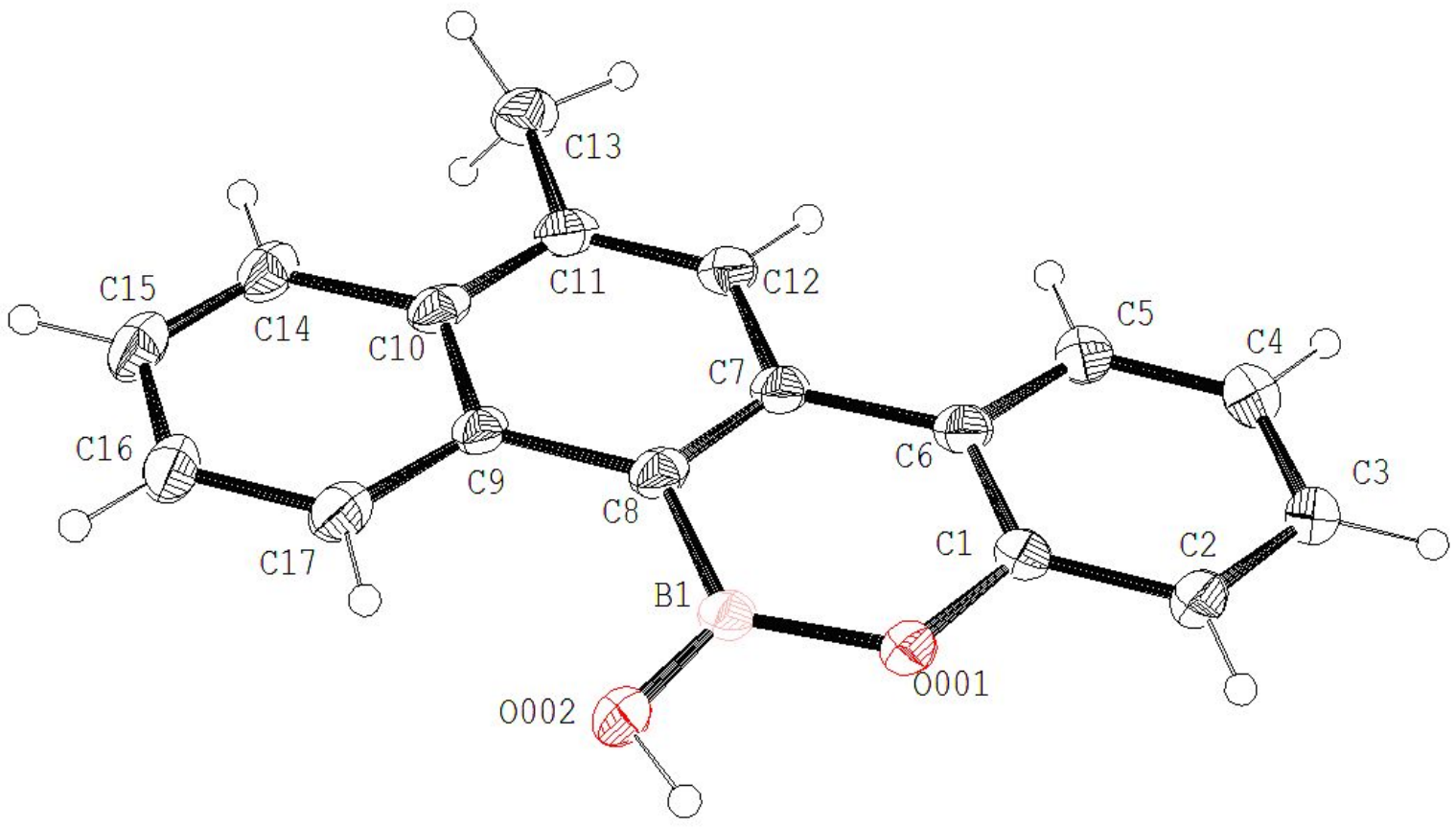

Figure S11. ORTEP plot of **8a** with 30% ellipsoids.

Table S5. Crystal data and structure refinement for compound **8a**.

Deposition number	2575420	
Identification code	**8a**	
Empirical formula	C_{17} H_{13} BO_2	
Formula weight	260.08	
Temperature [K]	200	
Wavelength [Å]	0.71073	
Crystal system	monoclinic	
Crystal habit	colourless needle	
Space Group	P 2_1/n	
Unit cell dimensions	a = 13.7964(9) Å	α = 90 º
	b = 5.3701(3) Å	β = 95.340(3)
	c = 16.9408(10)Å	γ = 90 º
Volume [Å^3]	1249.66(13)	
Z	4	
Density (calculated) [g cm^{-3}]	1.382	
Absorption Coefficient [mm^{-1}]	0.088	

F(000)	544
Cristal size [mm^3]	0.406 x 0.099 x 0.06
Diffractometer	κ-geometry diffractometer
Radiation source	MoKα
θ range for data collection	3.647 to 25.021 º
Index ranges	-16 <= h <= 16, -6 <= k <= 6, -19 <= l <= 20
Reflection collected	22341
Independent reflections	2207 [R(int) = 0.0994]
Reflections [I>2σ(I)]	1594
Completeness to θ = 25.021 º	0.998
Absorption correction	multi-scan
Max. and min. transmission	0.7456 and 0.6157
Refinement method	full y Fsqd
Data / Restraints / Parameters	2207 / 0 / 183
Goodness-of-fit on F^2	1.104
Final R indices [I>2σ(I)]	R1 = 0.0818, wR2 = 0.1901
R indices (all data)	R1 = 0.1122, wR2 = 0.2065
Largest diff. peak and hole [e. $Å^{-3}$]	0.410 and -0.240

Single crystals from compound **17** obtained by slow evaporation of a saturated hexane solution were coated in mineral oil and X-ray intensity were collected at 200 K on a Bruker-Nonius KappaCCD diffractometer equipped with graphite-monochromated Mo Kα radiation (λ = 0.71073 Å) and an Oxford Cryostream 700 unit. The structure was solved with ShelXT (intrinsic phasing) and refined by ShelXL (least square minimization) in Olex2.

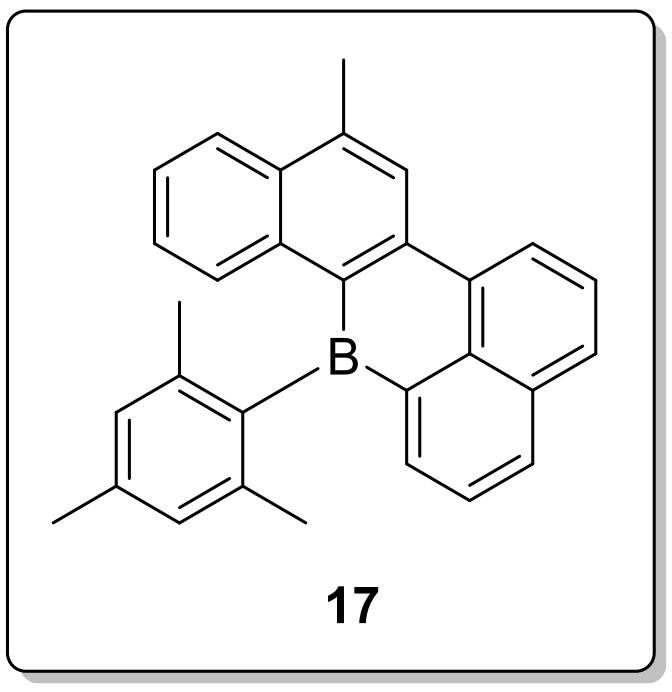

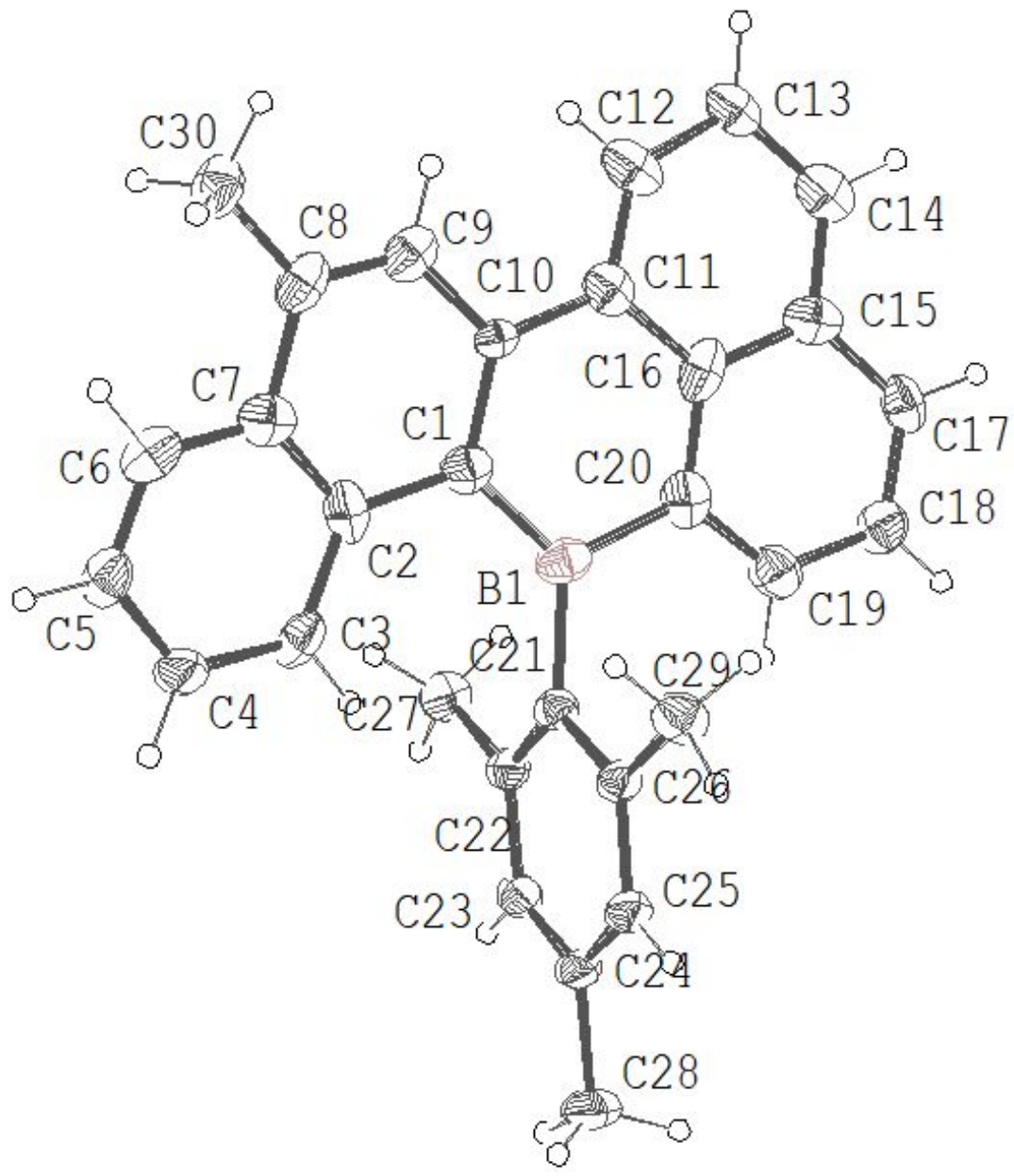

Figure S12. ORTEP plot of **17** with 30% ellipsoids.

Table S6. Crystal data and structure refinement for compound **17**.

Deposition number	2575425	
Identification code	**17**	
Empirical formula	$C_{30}H_{25}B$	
Formula weight	396.31	
Temperature [K]	200.00	
Wavelength [Å]	0.71073	
Crystal system	monoclinic	
Crystal habit	yellow block	
Space Group	$P2_1/n$	
Unit cell dimensions	a = 7.6066 (3)) Å	α = 90 º
	b = 16.7325 (8) Å	β = 110.637(2) º
	c = 17.1715 (7) Å	γ = 90 º

Volume [Å^3]	2147.99 (16)
Z	4
Density (calculated) [g cm^{-3}]	1.225
Absorption Coefficient [mm^{-1}]	0.068
F(000)	840
Cristal size [mm^3]	0.211 x 0.166 x 0.148
Diffractometer	κ-geometry diffractometer
Radiation source	MoKα
θ range for data collection	3.403 to 25.022 º
Index ranges	-9 <= h <= 9, -19 <= k <= 19, -20 <= l <= 20
Reflection collected	62176
Independent reflections	3773 [R(int) = 0.089]
Reflections [I>2σ(I)]	3045
Absorption correction	multi-scan
Max. and min. transmission	0.7456 and 0.6968
Refinement method	full y Fsqd
Data / Restraints / Parameters	4773 / 273 / 284
Goodness-of-fit on F^2	1.085
Final R indices [I>2σ(I)]	R1 = 0.1127, wR2 = 0.2975
R indices (all data)	R1 = 0.1300, wR2 = 0.3116
Largest diff. peak and hole [e. Å^{-3}]	1.036 and -0.307

Copies of ^{1}H, ^{13}C, ^{19}F and ^{11}B NMR spectra for novel compounds

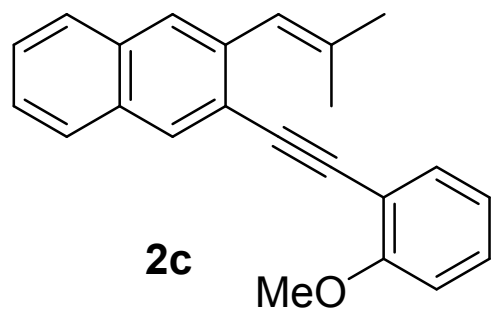

2c

^{1}H-NMR (400 MHz, $CDCl_3$)

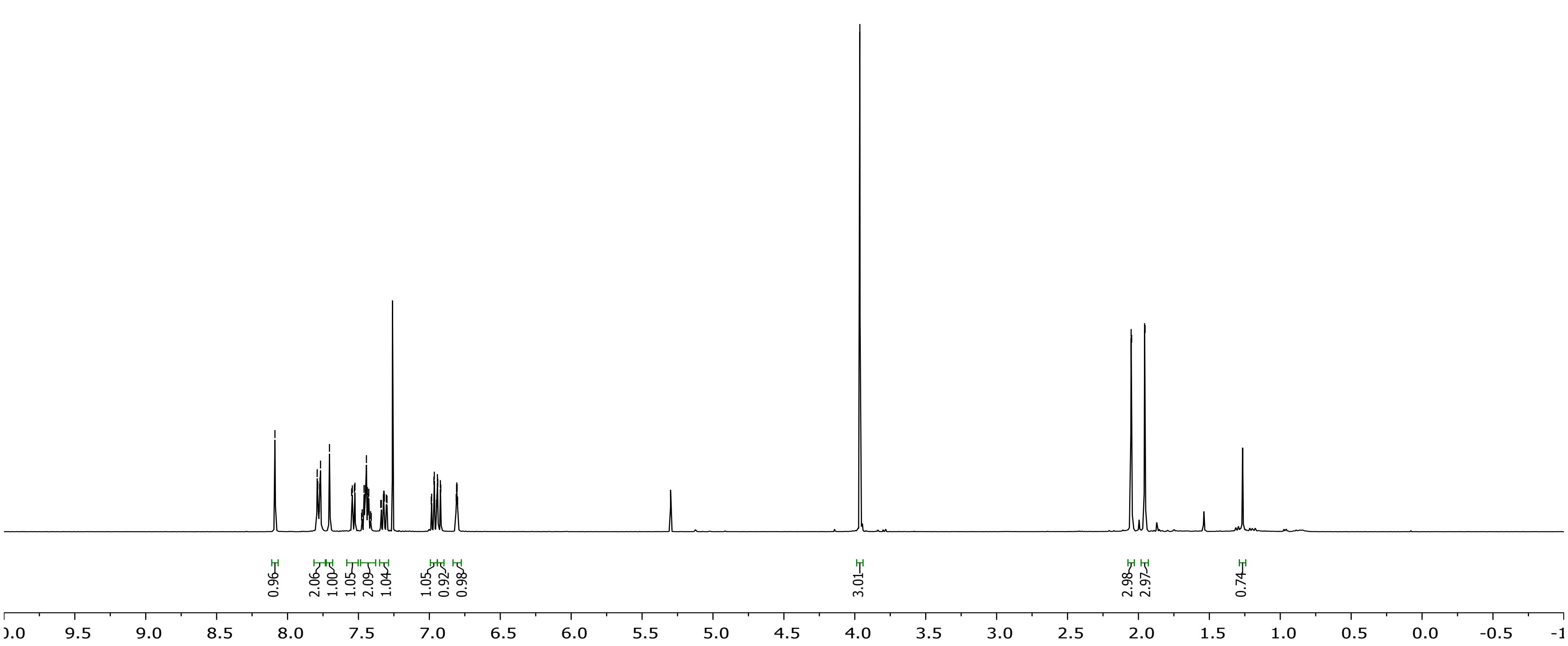

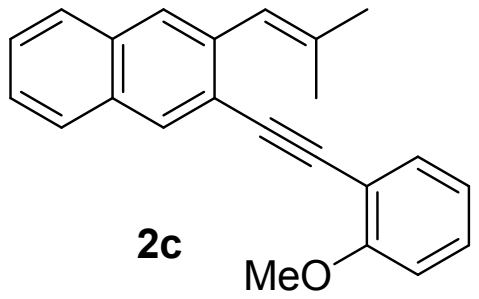

2c

^{13}C-NMR (101 MHz, $CDCl_3$)

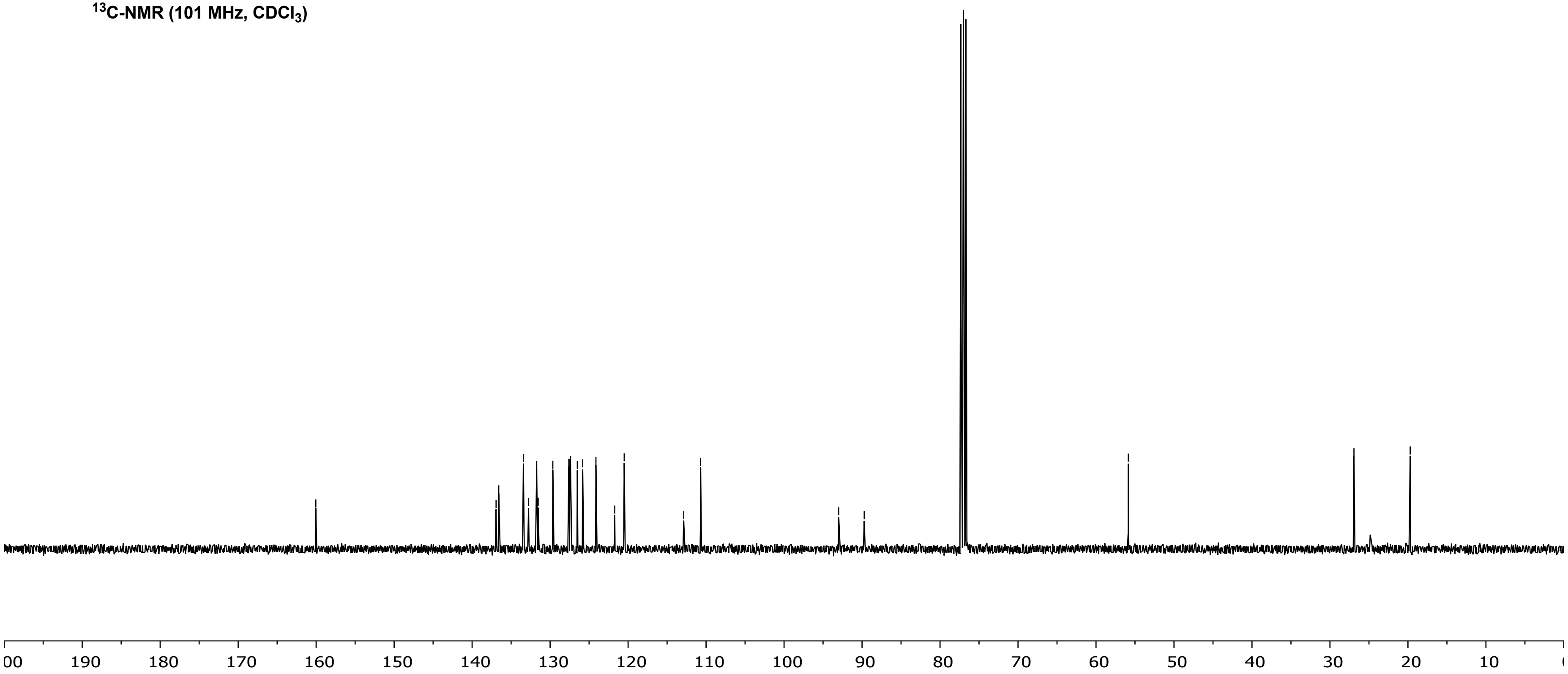

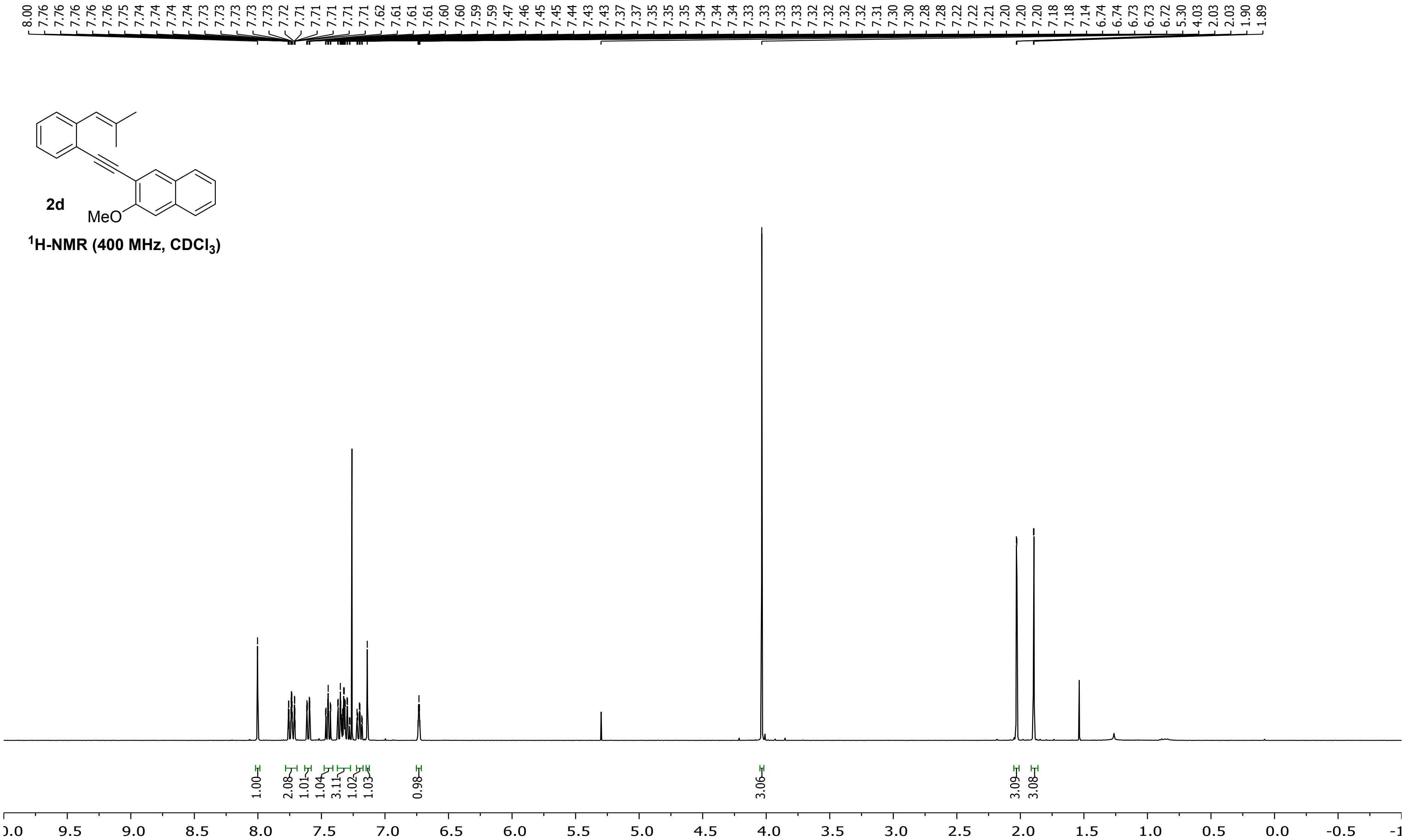
2d
MeO
^{1}H-NMR (400 MHz, $CDCl_3$)
1.00
2.08
1.01
1.04
3.11
1.02
1.03
0.98
3.06
3.09
3.08

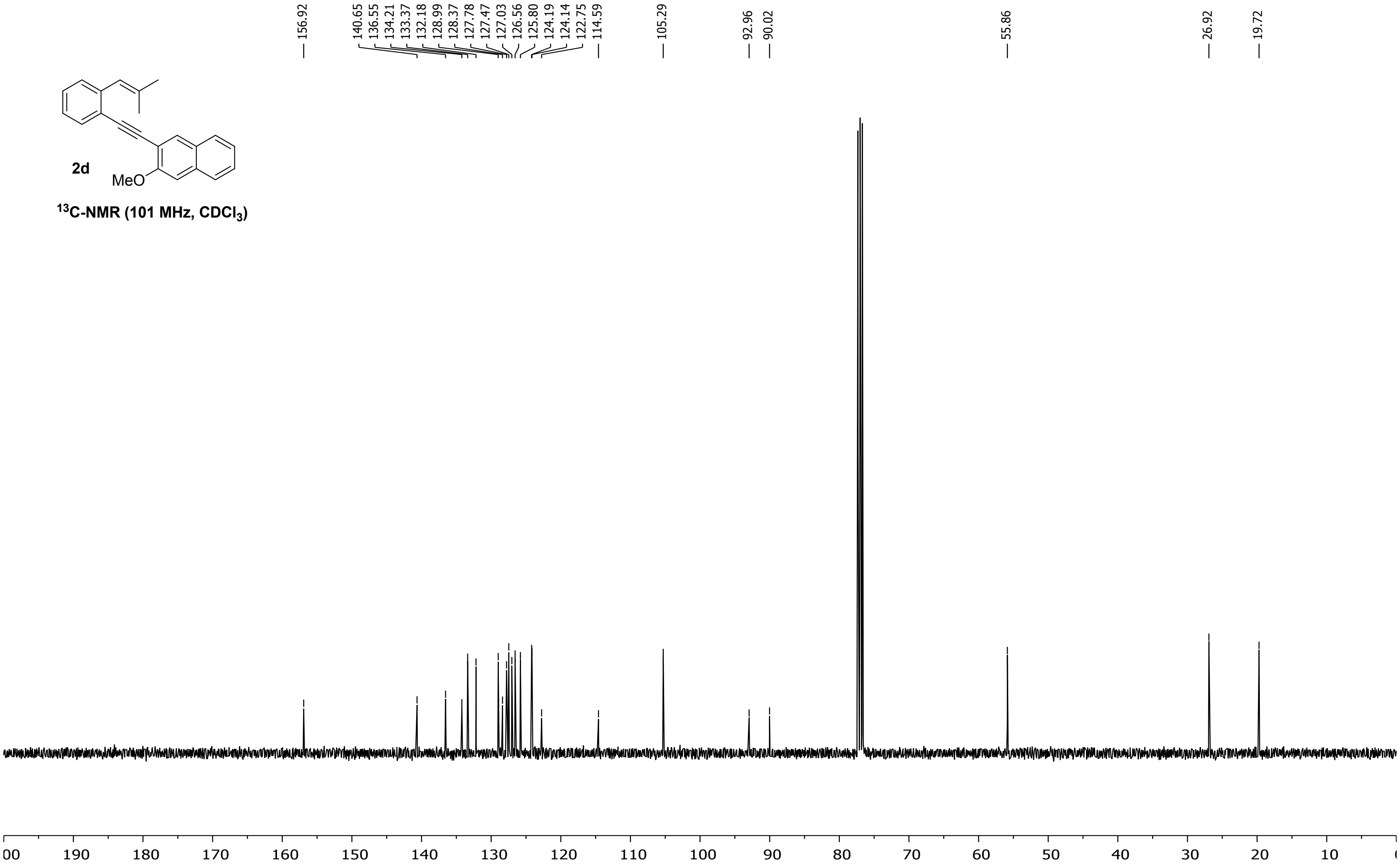

^{13}C-NMR (101 MHz, $CDCl_3$)

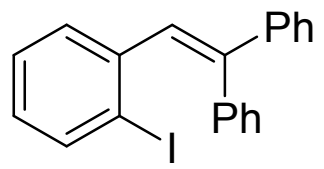

^{1}H-NMR (400 MHz, $CDCl_3$)

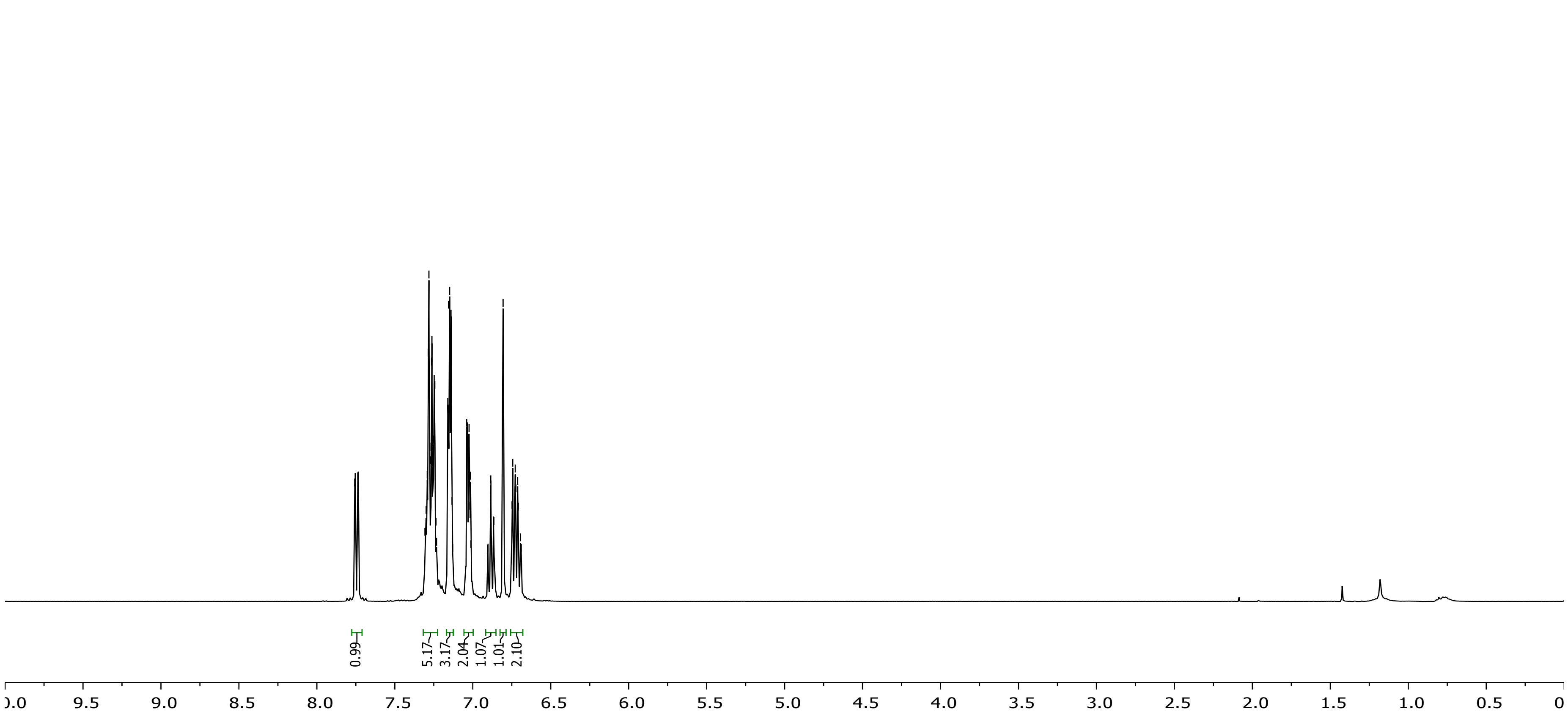

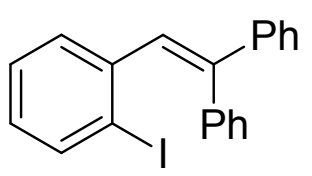

^{13}C-NMR (101 MHz, $CDCl_3$)

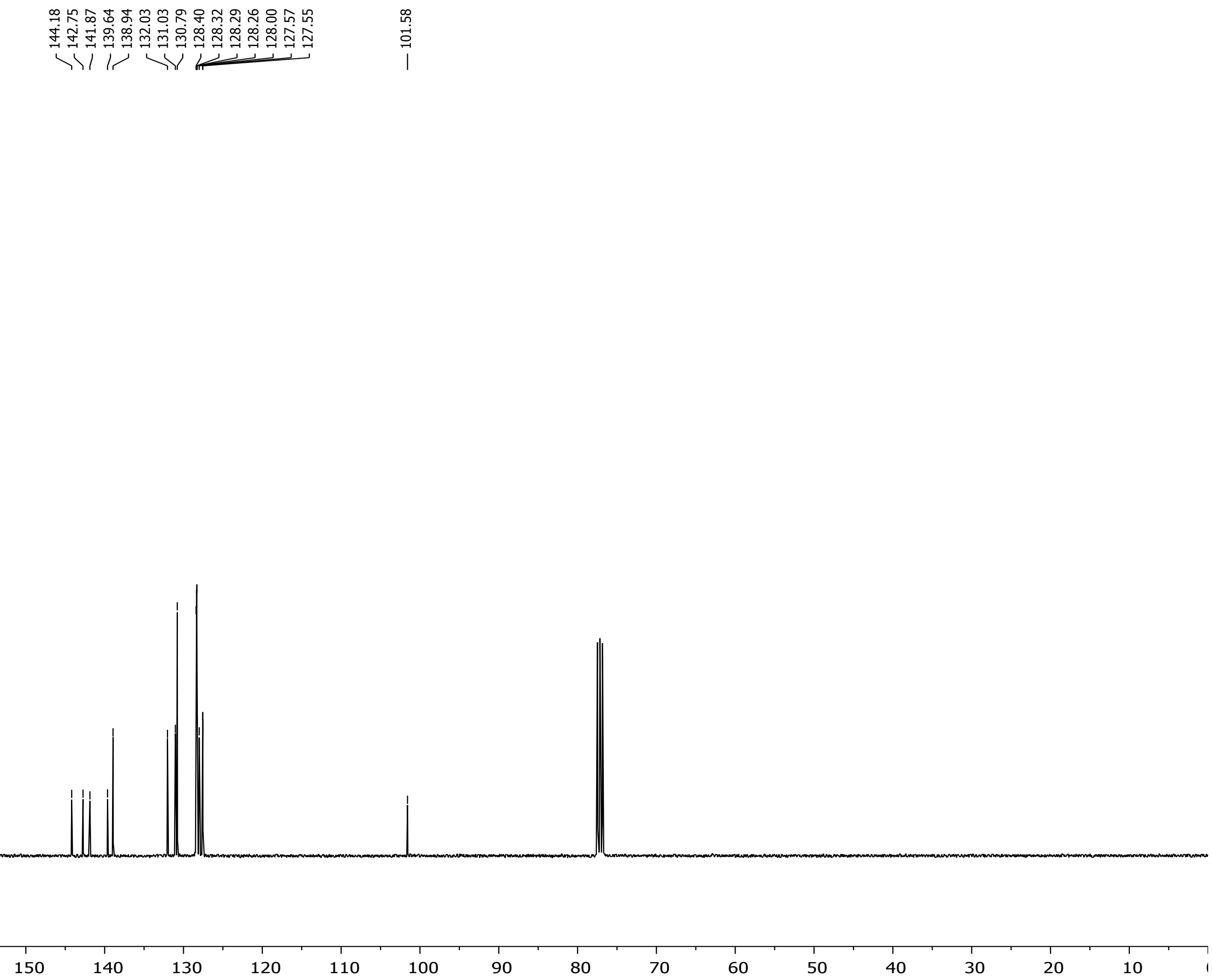

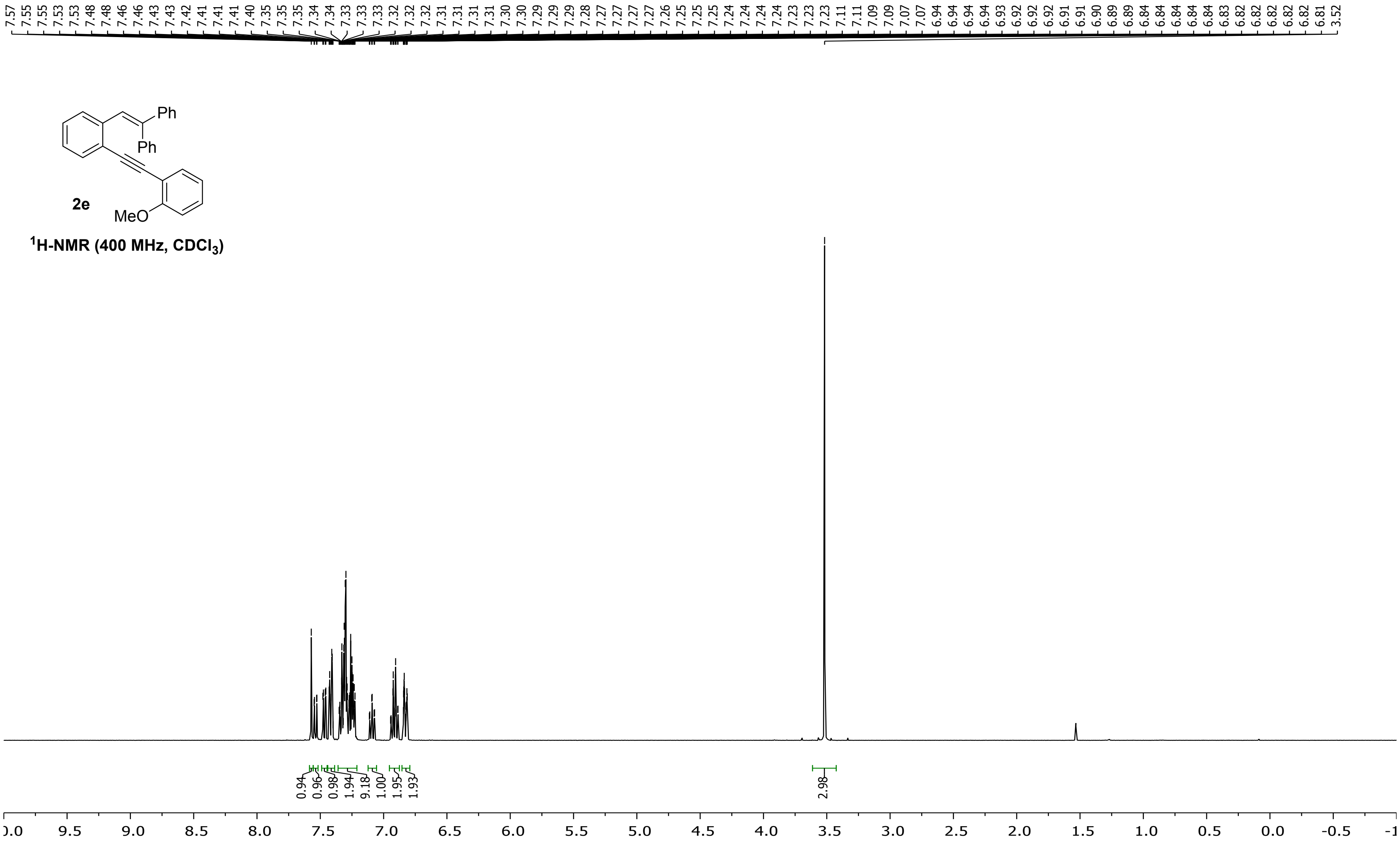
7.57 7.55 7.55 7.53 7.53 7.48 7.48 7.46 7.46 7.43 7.43 7.42 7.41 7.41 7.41 7.40 7.35 7.35 7.35 7.34 7.34 7.33 7.33 7.33 7.32 7.32 7.32 7.31 7.31 7.31 7.30 7.30 7.29 7.29 7.29 7.28 7.27 7.27 7.27 7.27 7.26 7.25 7.25 7.25 7.24 7.24 7.24 7.24 7.23 7.23 7.23 7.11 7.11 7.09 7.09 7.07 7.07 6.94 6.94 6.94 6.93 6.92 6.92 6.92 6.91 6.91 6.90 6.89 6.89 6.84 6.84 6.84 6.84 6.83 6.82 6.82 6.82 6.82 6.82 6.81 3.52
Ph
Ph
2e
MeO
^{1}H-NMR (400 MHz, CDCl$_3$)
0.94 0.96 0.98 1.94 9.18 1.00 1.95 1.93
2.98
0.0 9.5 9.0 8.5 8.0 7.5 7.0 6.5 6.0 5.5 5.0 4.5 4.0 3.5 3.0 2.5 2.0 1.5 1.0 0.5 0.0 -0.5 -1

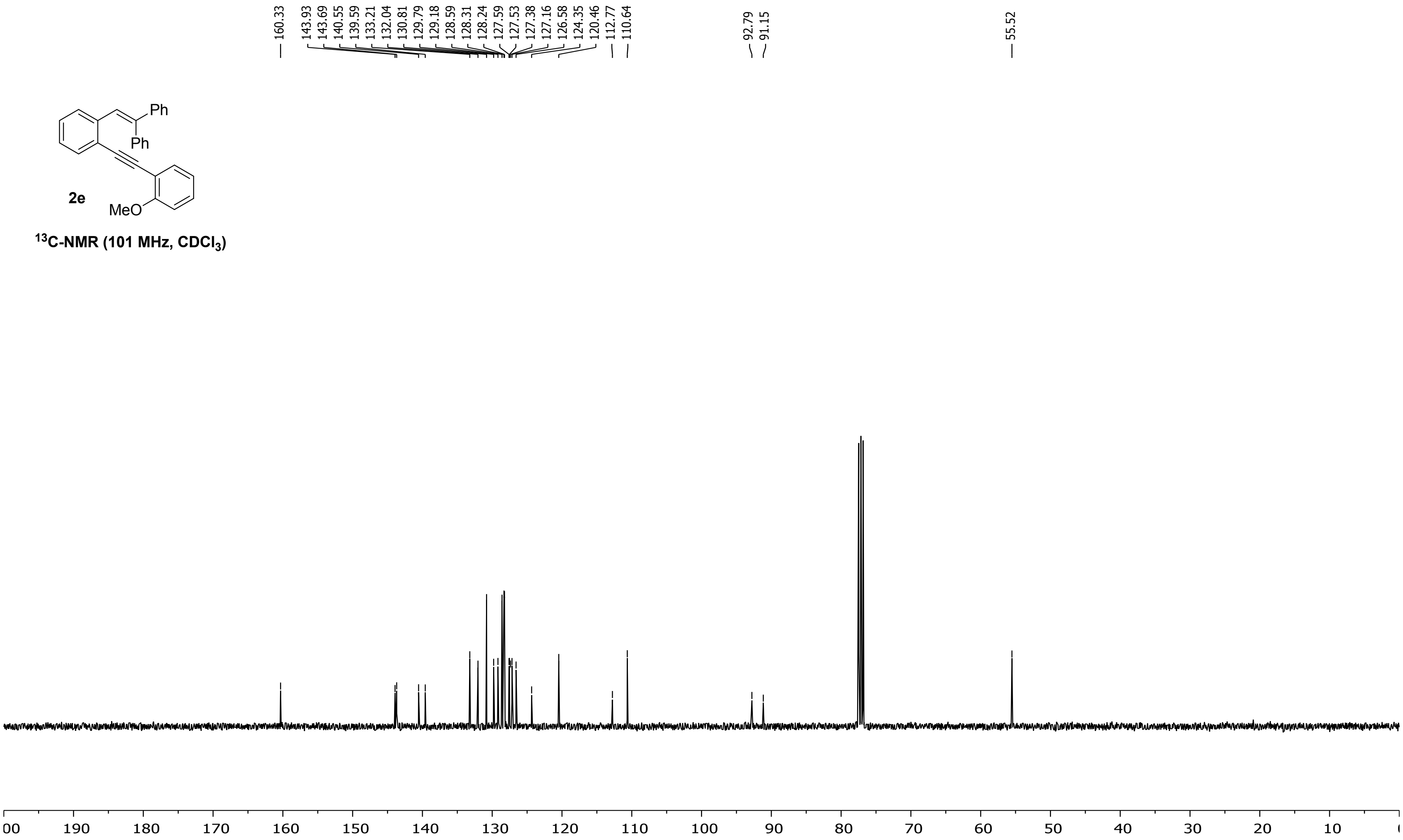
160.33
143.93
143.69
140.55
139.59
133.21
132.04
130.81
129.79
129.18
128.59
128.31
128.24
127.59
127.53
127.38
127.16
126.58
124.35
120.46
112.77
110.64
92.79
91.15
55.52
Ph
Ph
2e
MeO
^{13}C-NMR (101 MHz, $CDCl_3$)
00
190
180
170
160
150
140
130
120
110
100
90
80
70
60
50
40
30
20
10

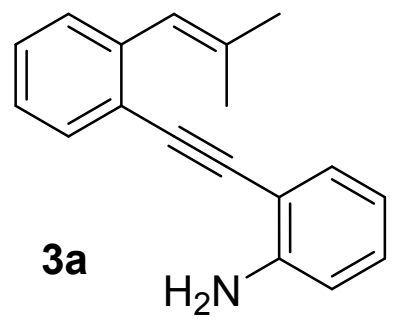

3a

^{1}H-NMR (400 MHz, $CDCl_3$)

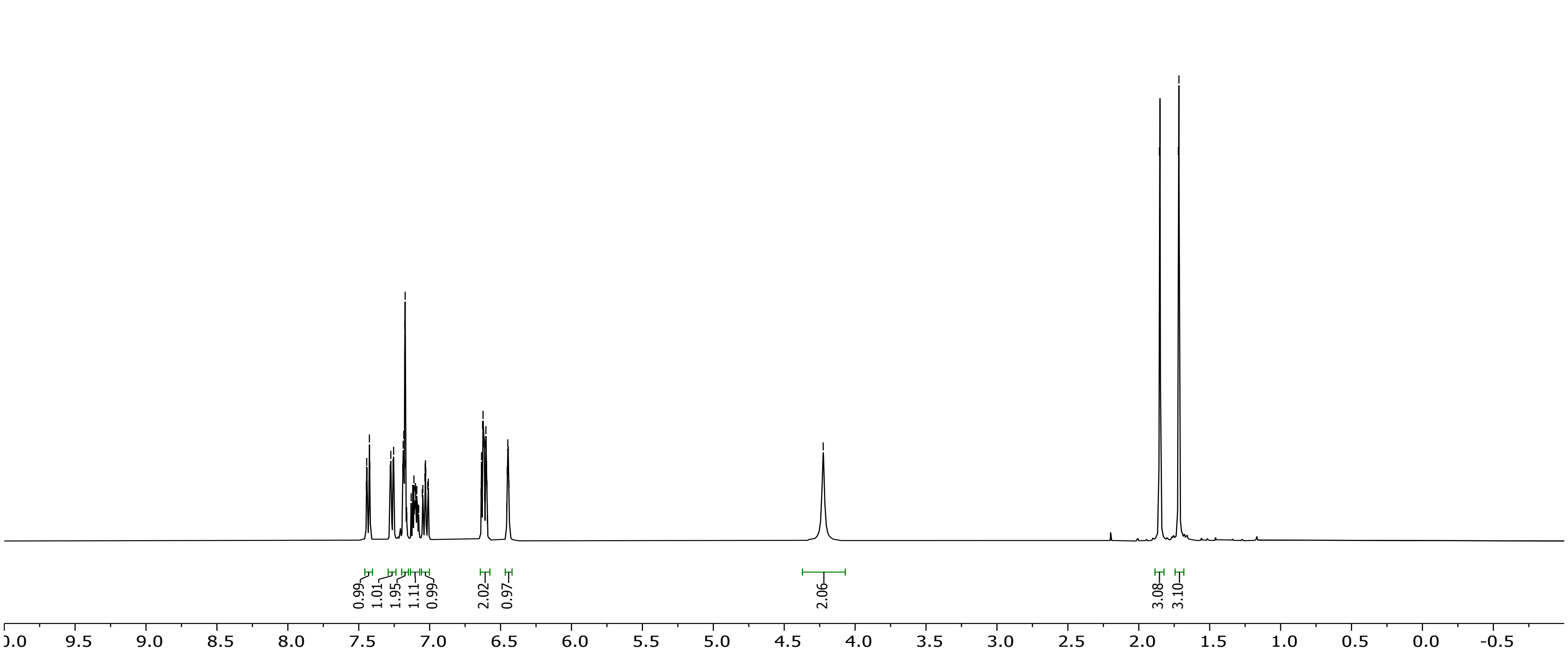

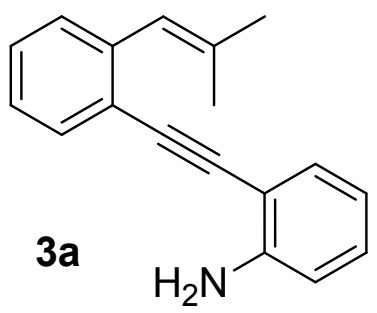

3a

^{13}C-NMR (101 MHz, $CDCl_3$)

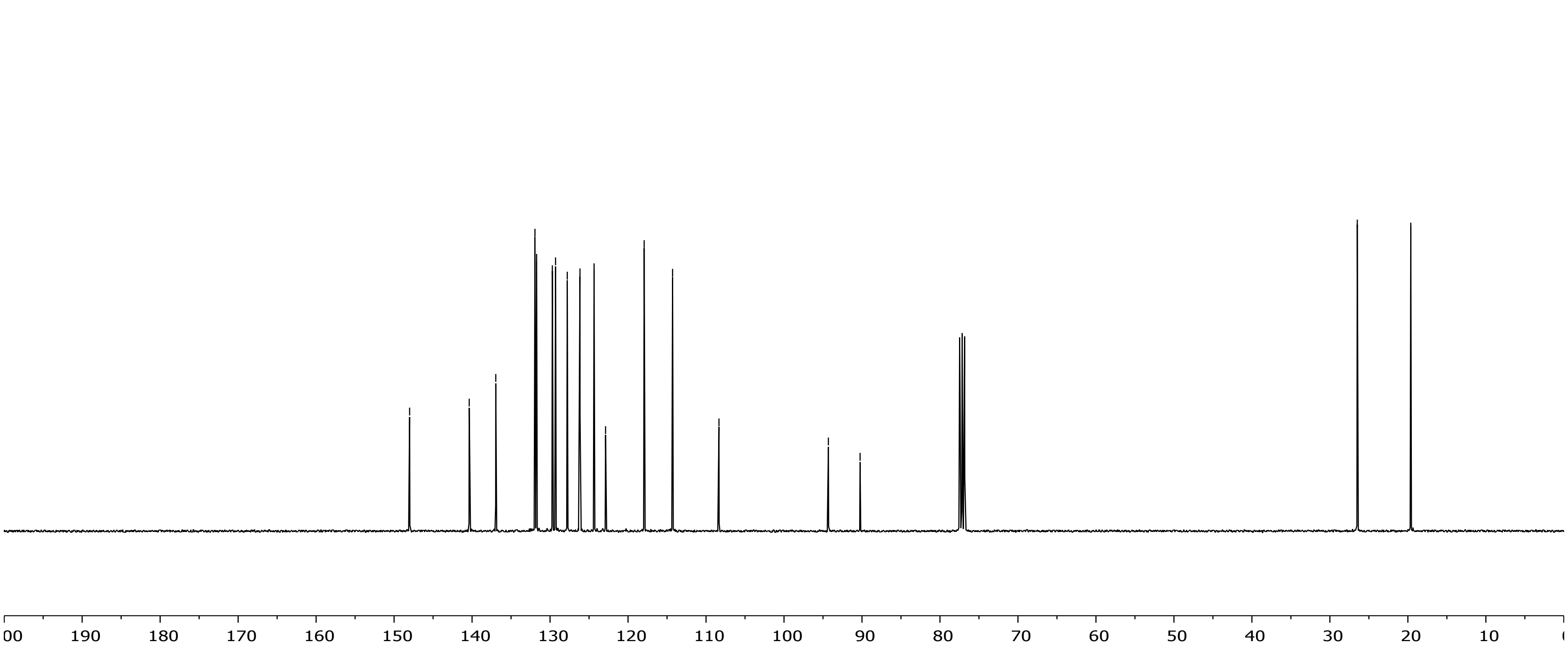

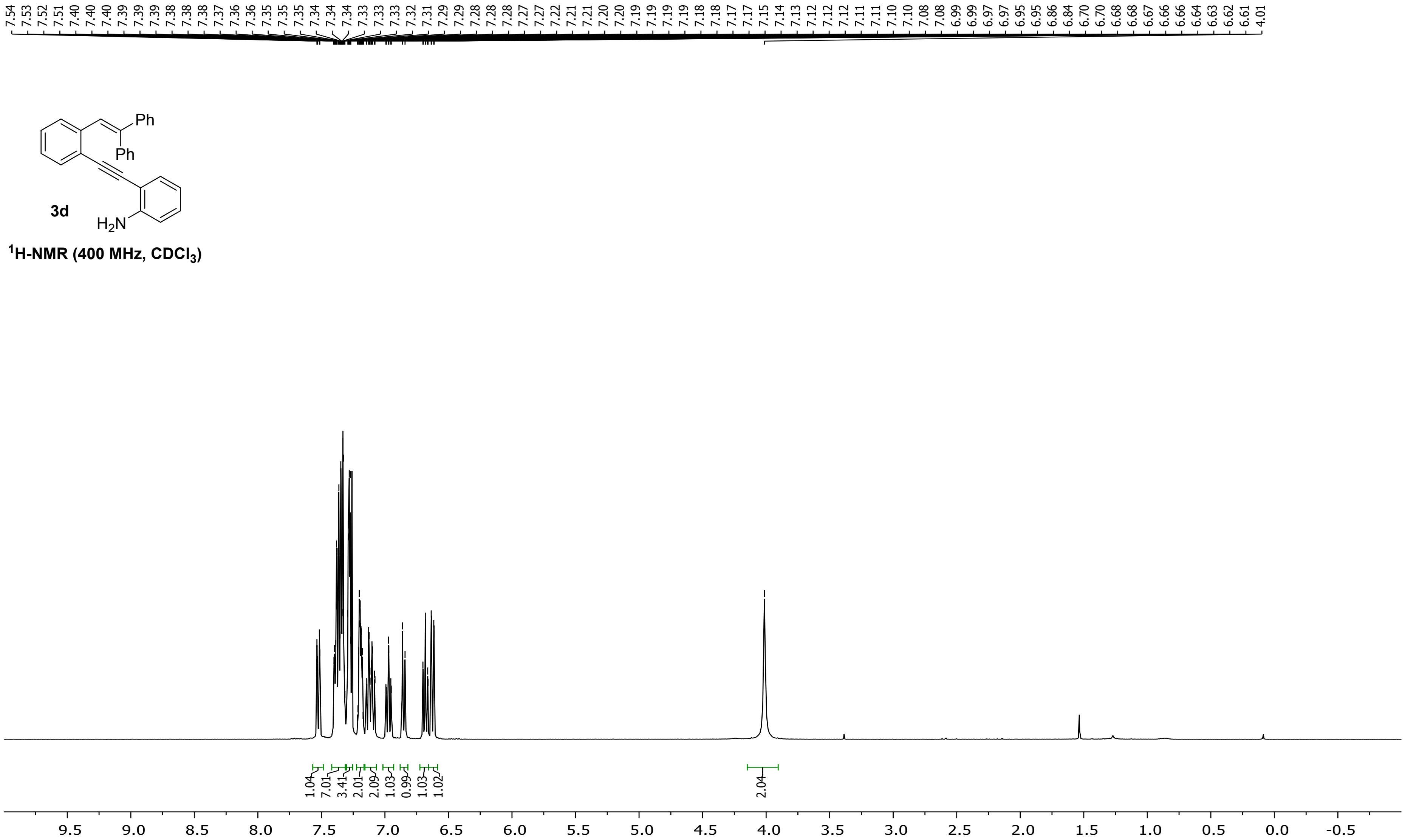
7.54 7.53 7.52 7.51 7.40 7.40 7.39 7.39 7.39 7.38 7.38 7.37 7.36 7.35 7.35 7.35 7.34 7.34 7.34 7.33 7.33 7.33 7.32 7.31 7.29 7.29 7.28 7.28 7.28 7.27 7.27 7.22 7.21 7.21 7.20 7.20 7.19 7.19 7.19 7.19 7.18 7.18 7.17 7.17 7.15 7.14 7.13 7.12 7.12 7.12 7.11 7.11 7.10 7.10 7.08 7.08 6.99 6.99 6.97 6.97 6.95 6.95 6.86 6.84 6.70 6.70 6.68 6.68 6.67 6.66 6.66 6.64 6.63 6.62 6.61 4.01
Ph
Ph
3d
H$_2$N
^{1}H-NMR (400 MHz, CDCl$_3$)
1.04 7.01 3.41 2.01 2.09 1.03 0.99 1.03 1.02
2.04
9.5 9.0 8.5 8.0 7.5 7.0 6.5 6.0 5.5 5.0 4.5 4.0 3.5 3.0 2.5 2.0 1.5 1.0 0.5 0.0 -0.5

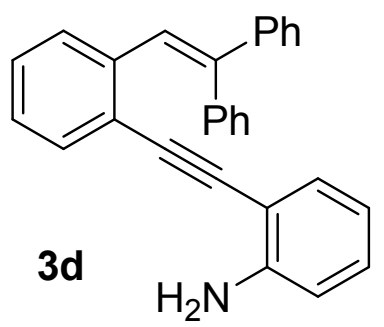

3d

^{13}C-NMR (101 MHz, CDCl$_3$)

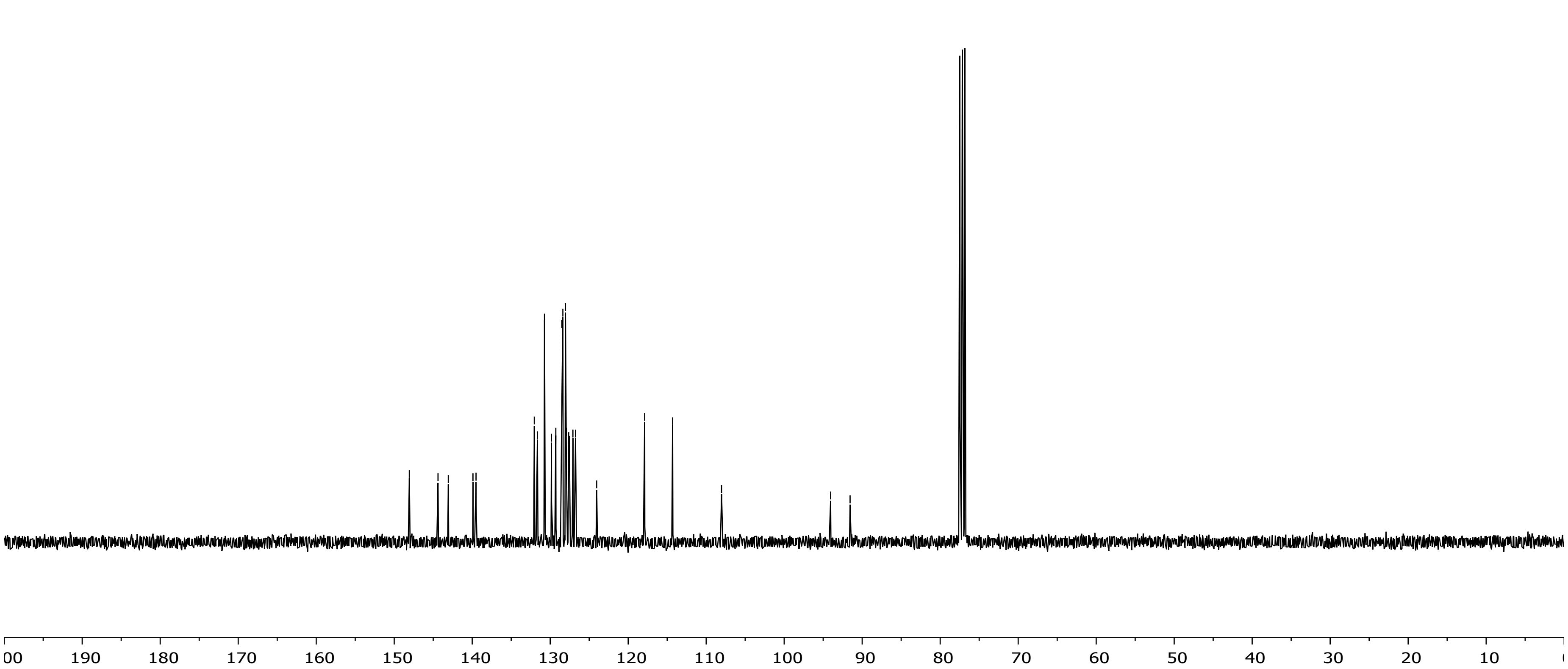

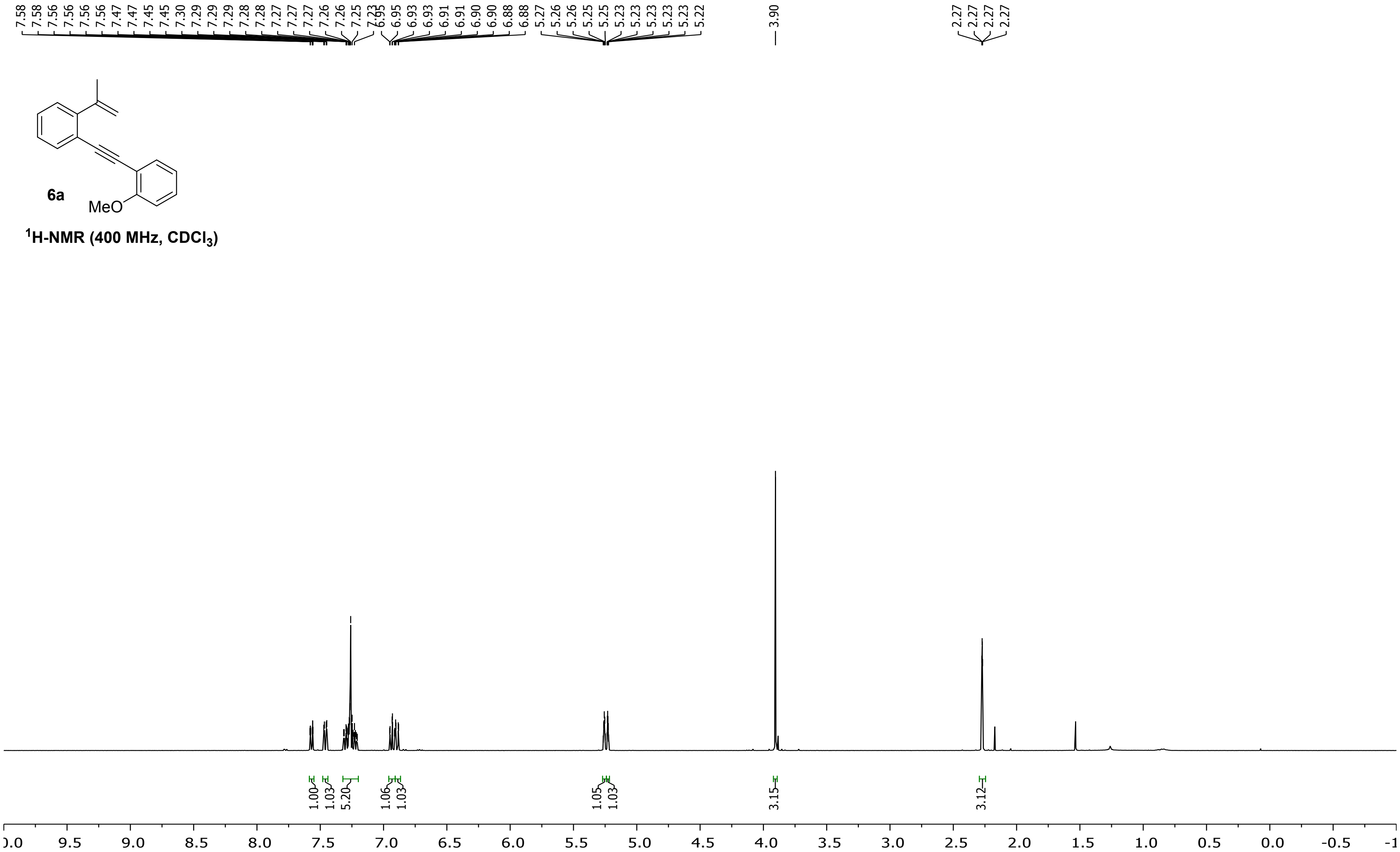
7.58 7.58 7.56 7.56 7.56 7.47 7.47 7.45 7.45 7.30 7.29 7.29 7.29 7.28 7.28 7.27 7.27 7.27 7.26 7.26 7.25 7.23 6.95 6.95 6.93 6.93 6.91 6.91 6.90 6.88 6.88 5.27 5.26 5.26 5.25 5.25 5.23 5.23 5.23 5.23 5.23 5.22
3.90
2.27 2.27 2.27 2.27
6a
MeO
^{1}H-NMR (400 MHz, $CDCl_3$)
1.00 1.03 5.20 1.06 1.03 1.05 1.03 3.15 3.12
10.0 9.5 9.0 8.5 8.0 7.5 7.0 6.5 6.0 5.5 5.0 4.5 4.0 3.5 3.0 2.5 2.0 1.5 1.0 0.5 0.0 -0.5 -1

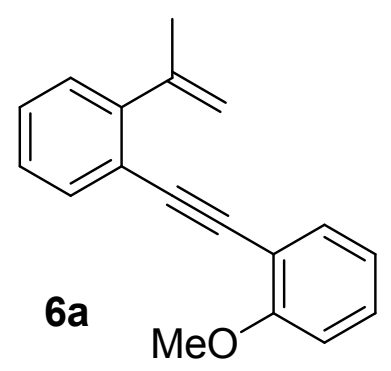

6a

^{13}C-NMR (101 MHz, $CDCl_3$)

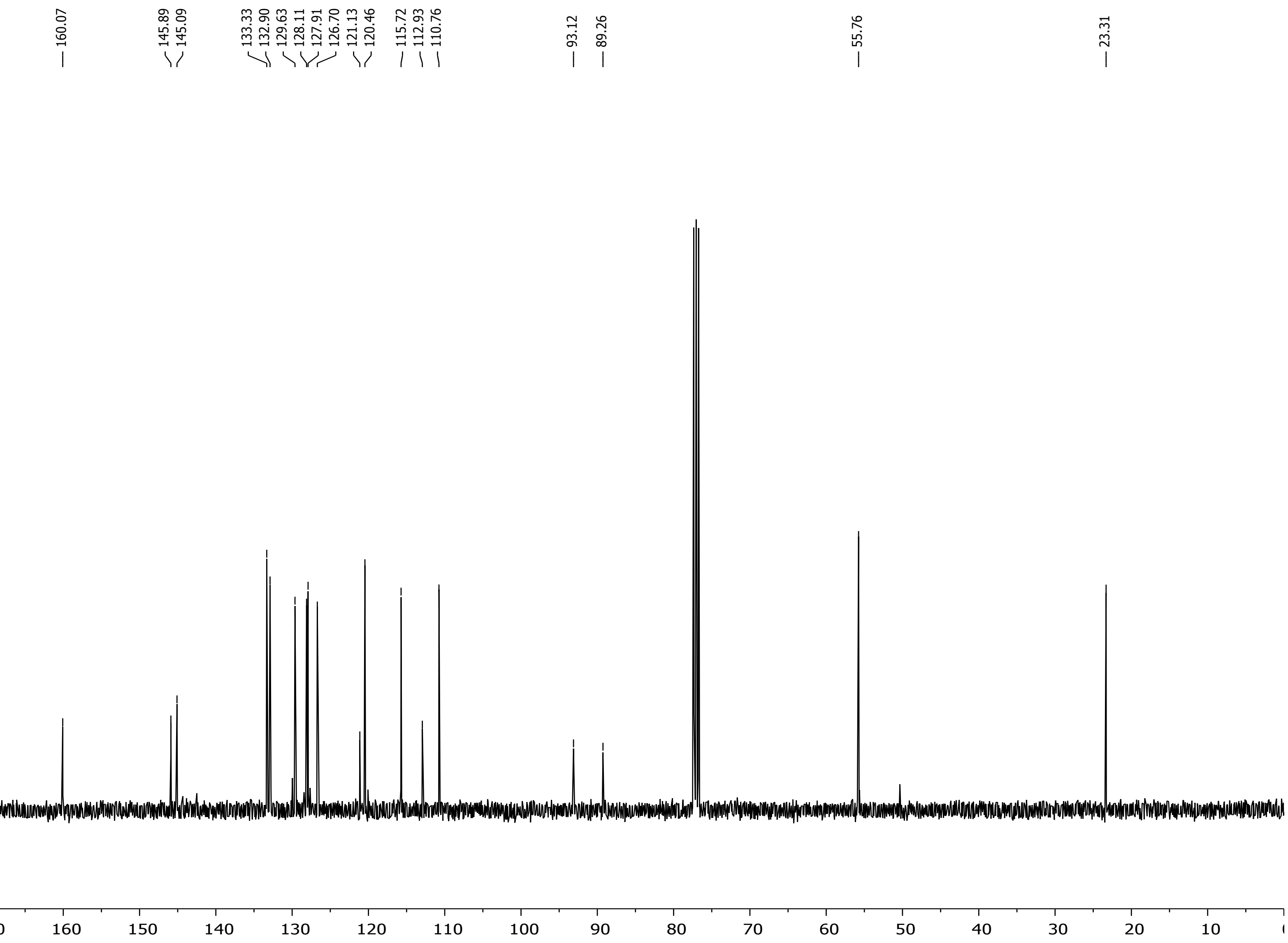

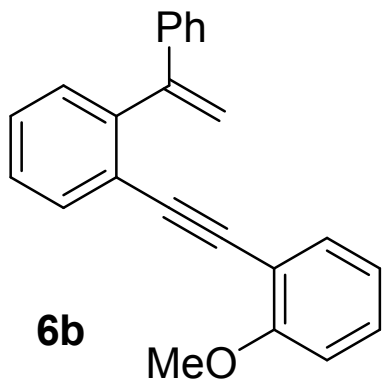

6b

^{1}H-NMR (400 MHz, $CDCl_3$)

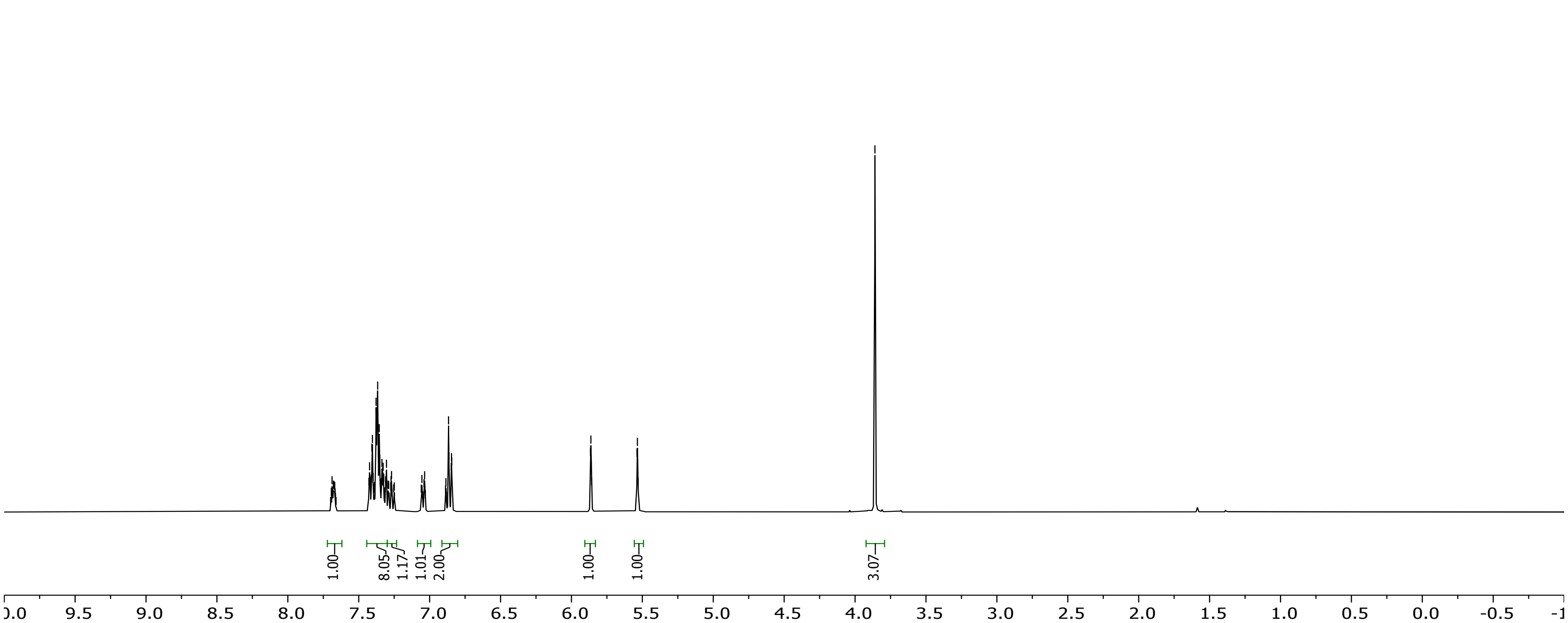

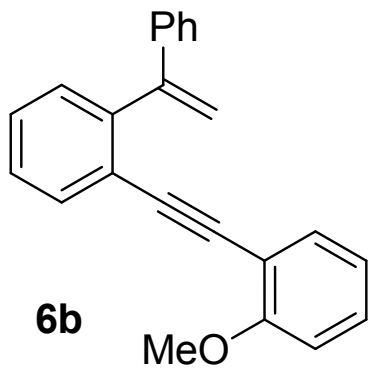

6b

^{13}C-NMR (101 MHz, CDCl$_3$)

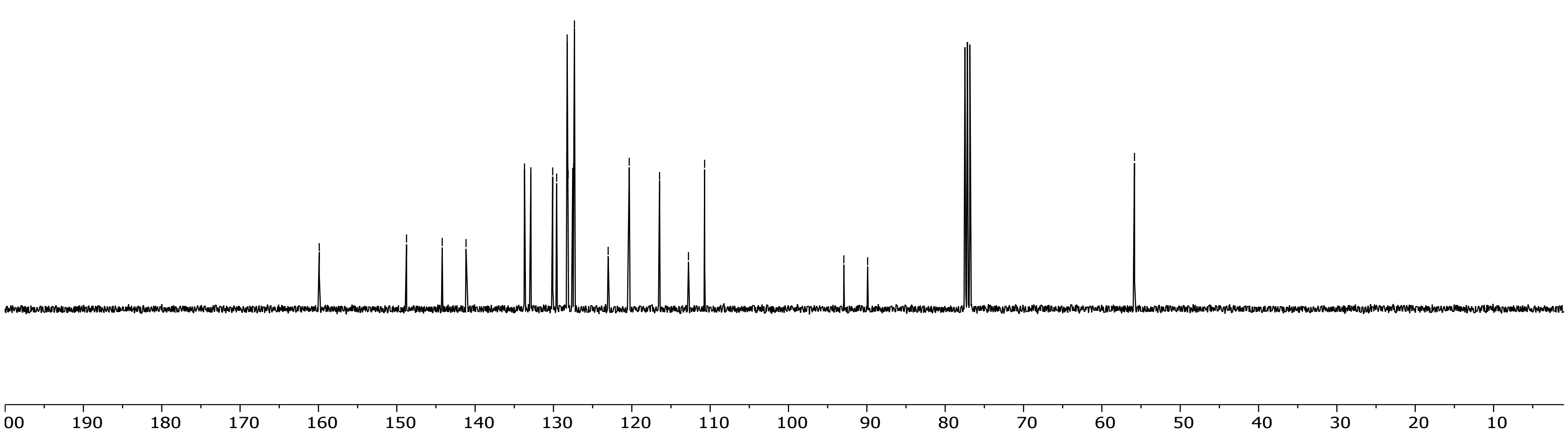

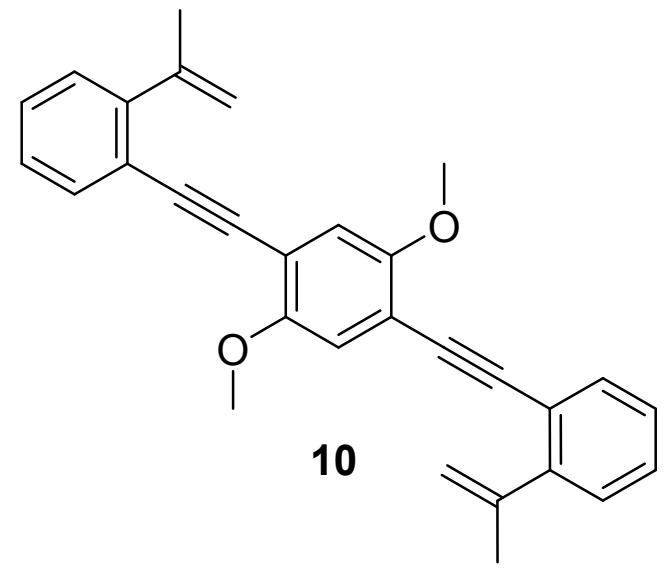

10

^{1}H-NMR (400 MHz, CDCl$_3$)

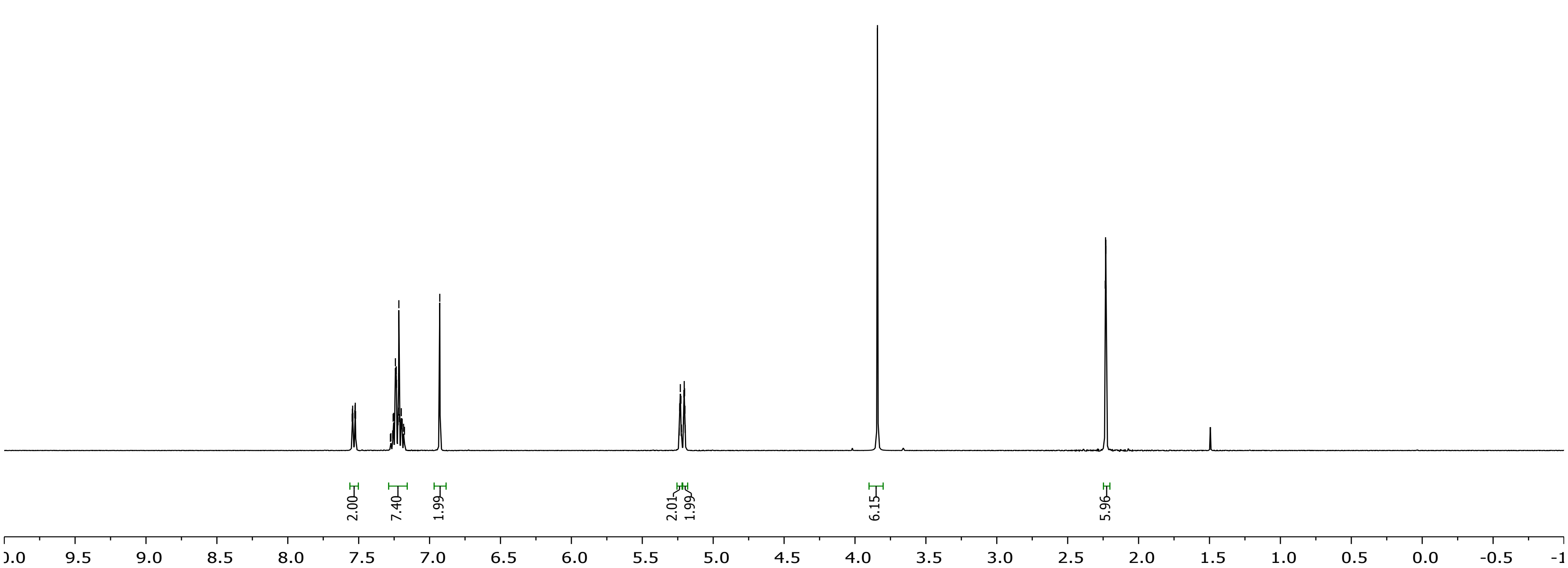

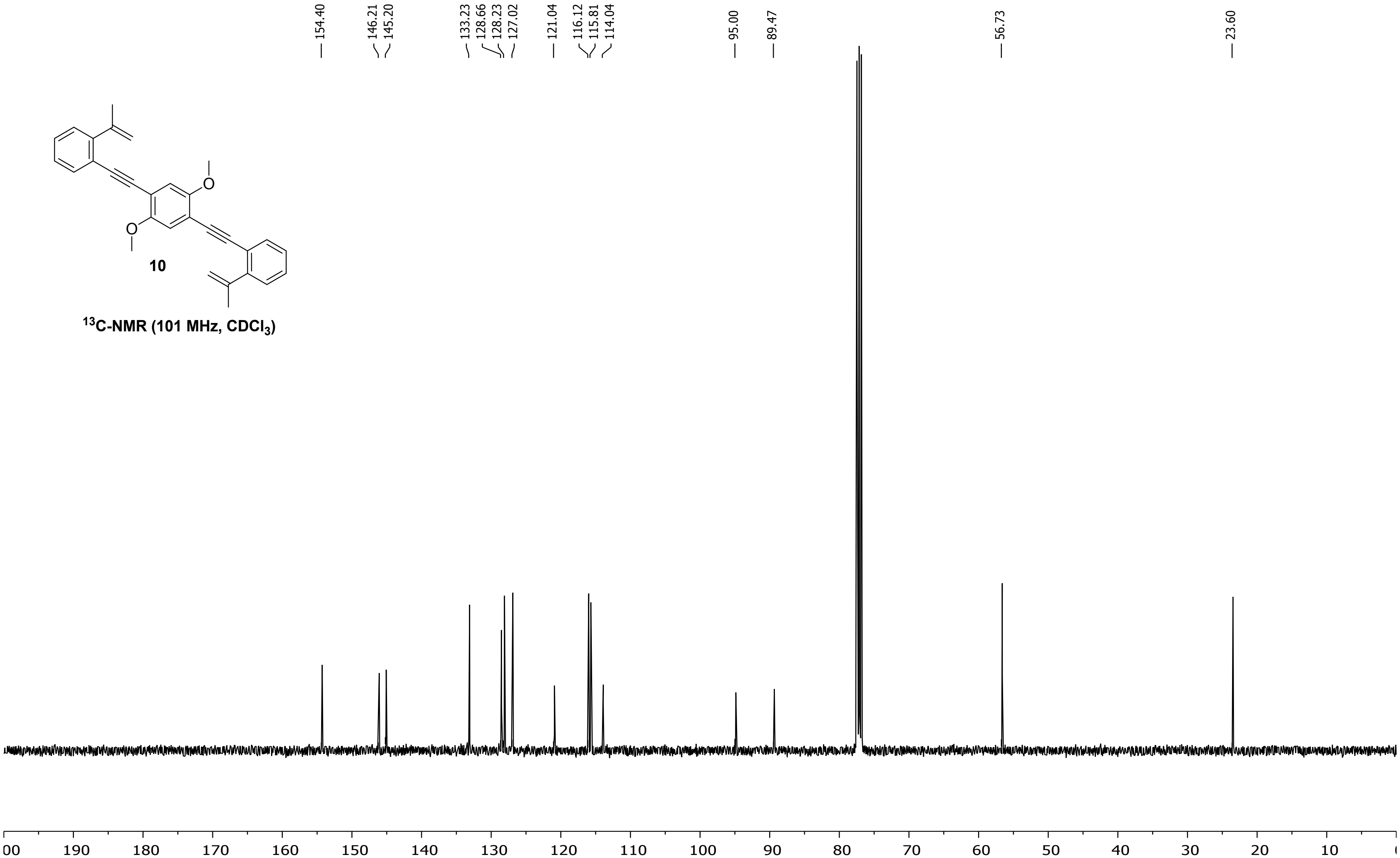

10

^{13}C-NMR (101 MHz, CDCl$_3$)

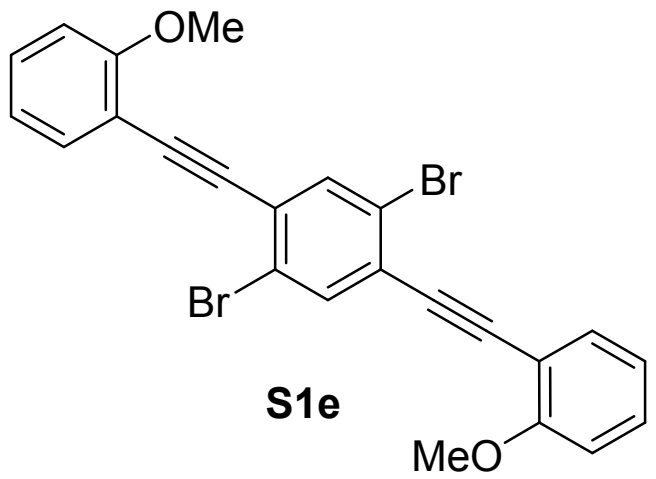

S1e

^{1}H-NMR (400 MHz, CDCl$_3$)

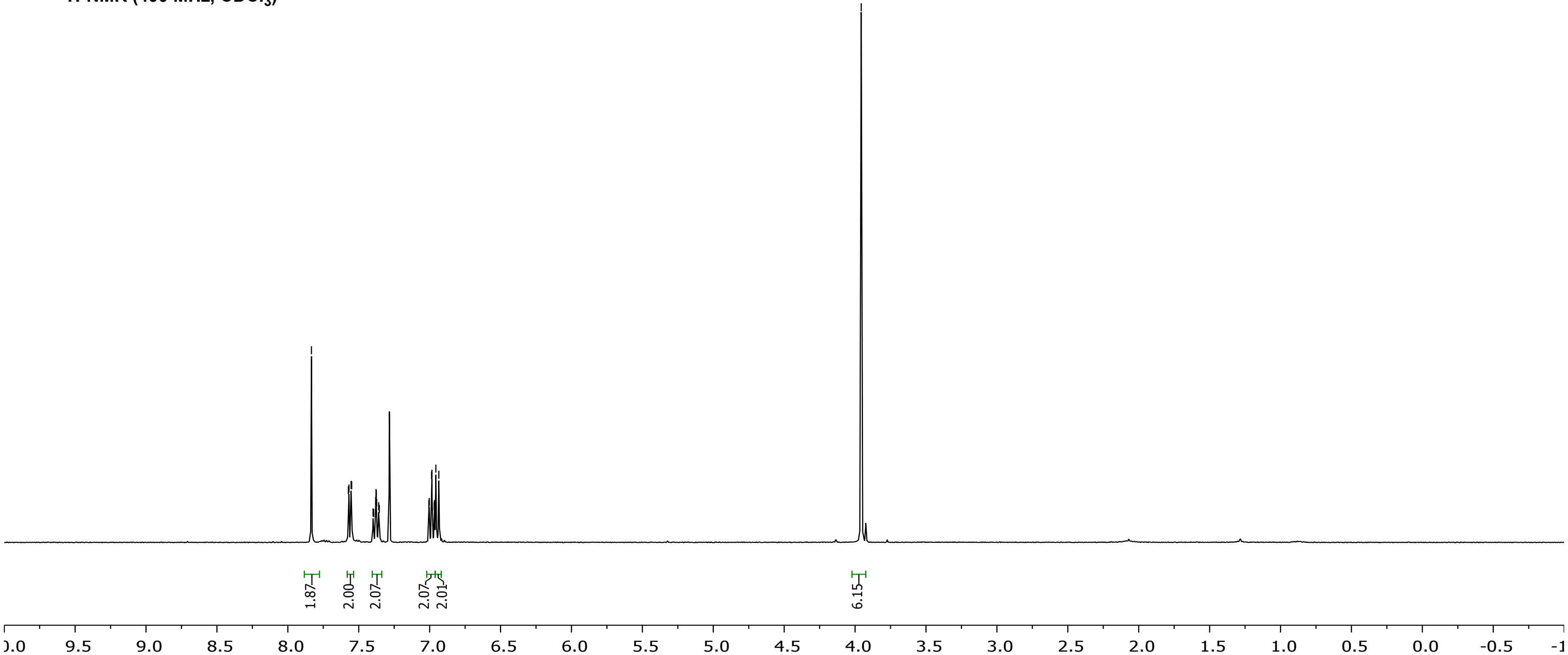

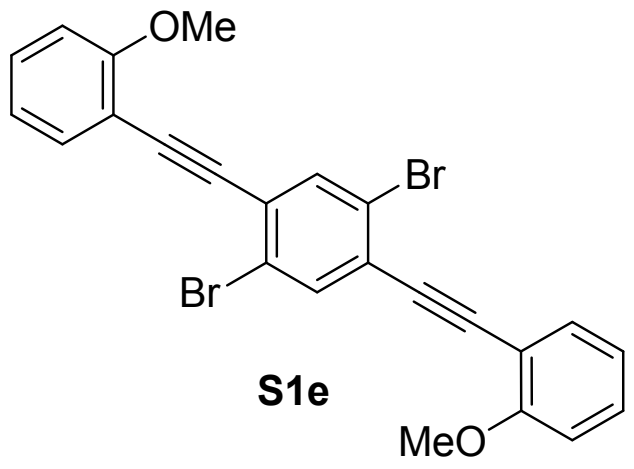

S1e

^{13}C-NMR (101 MHz, $CDCl_3$)

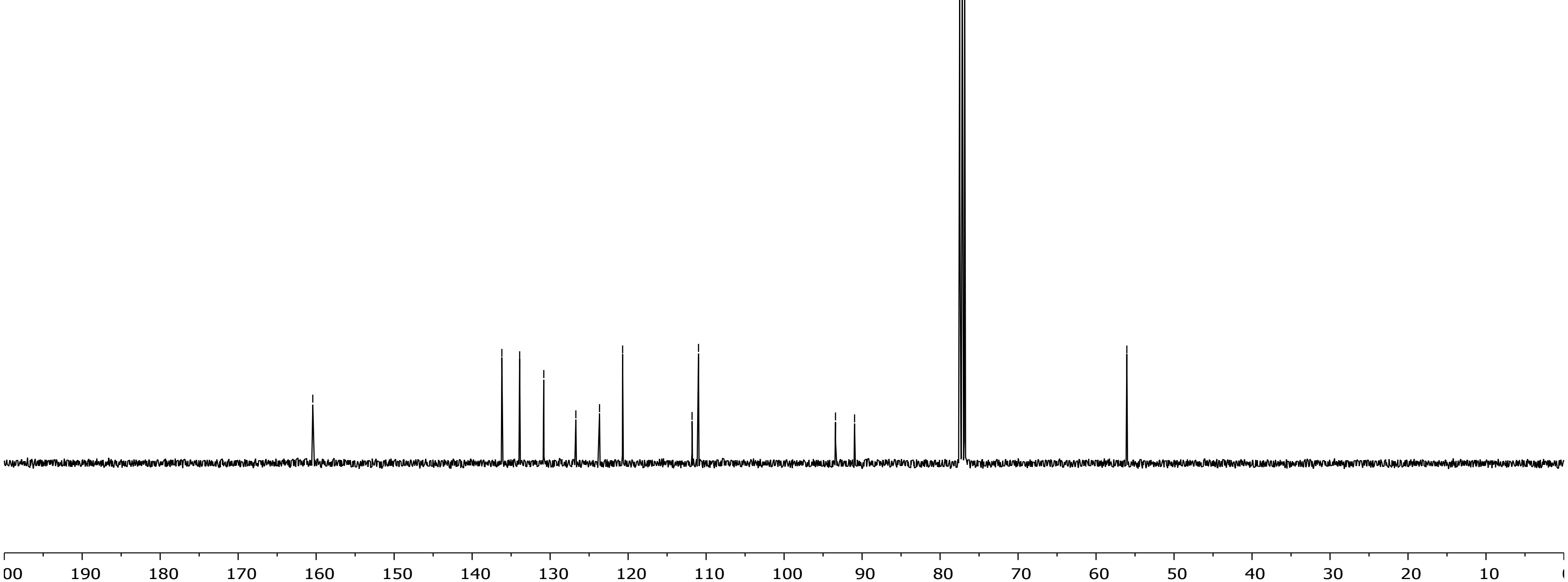

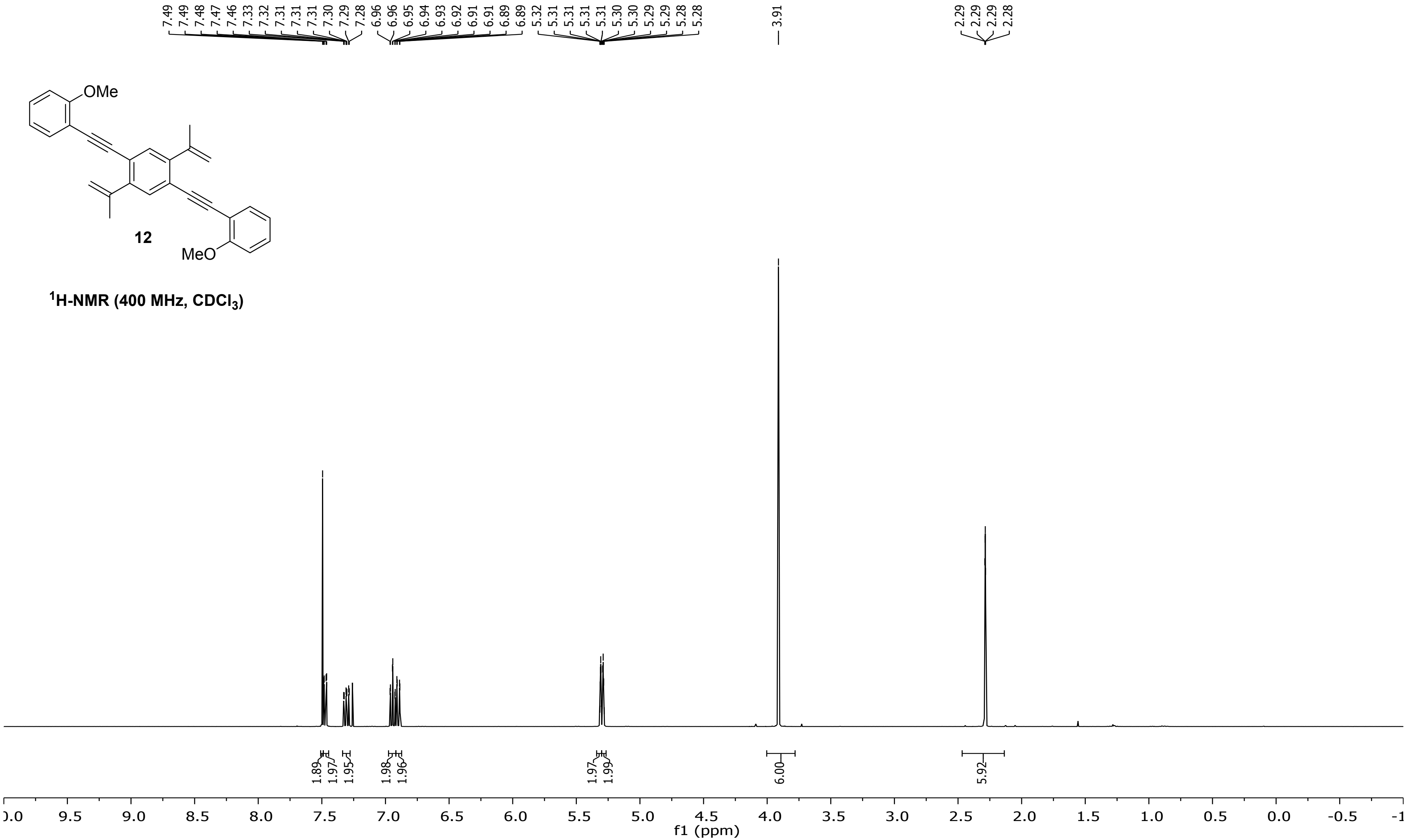

12

^{1}H-NMR (400 MHz, $CDCl_3$)

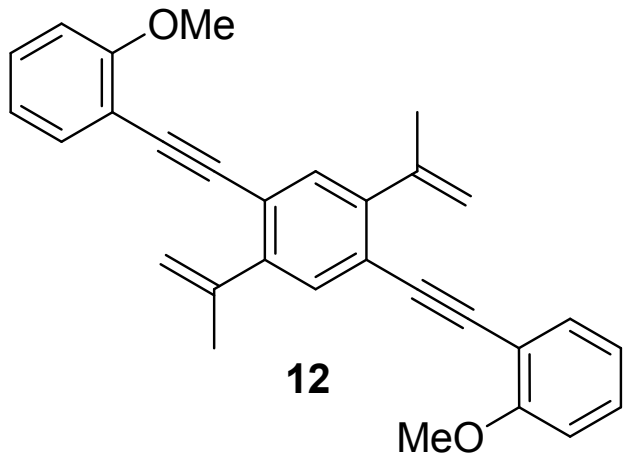

12

^{13}C-NMR (101 MHz, $CDCl_3$)

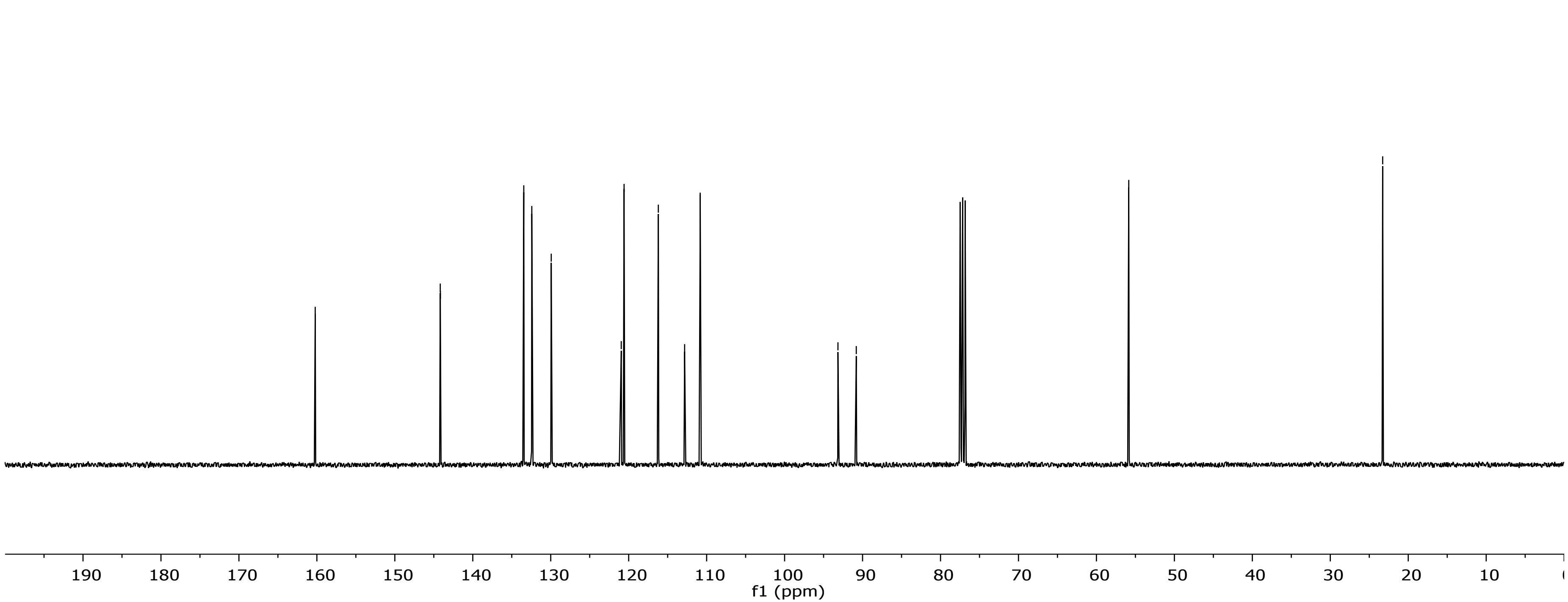

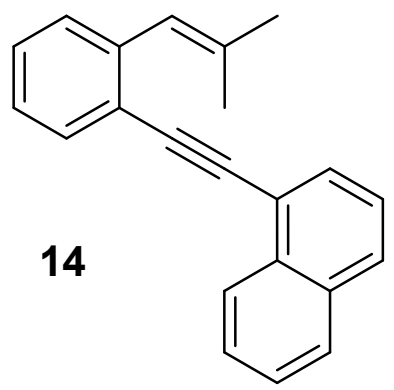

14

^{1}H-NMR (400 MHz, $CDCl_3$)

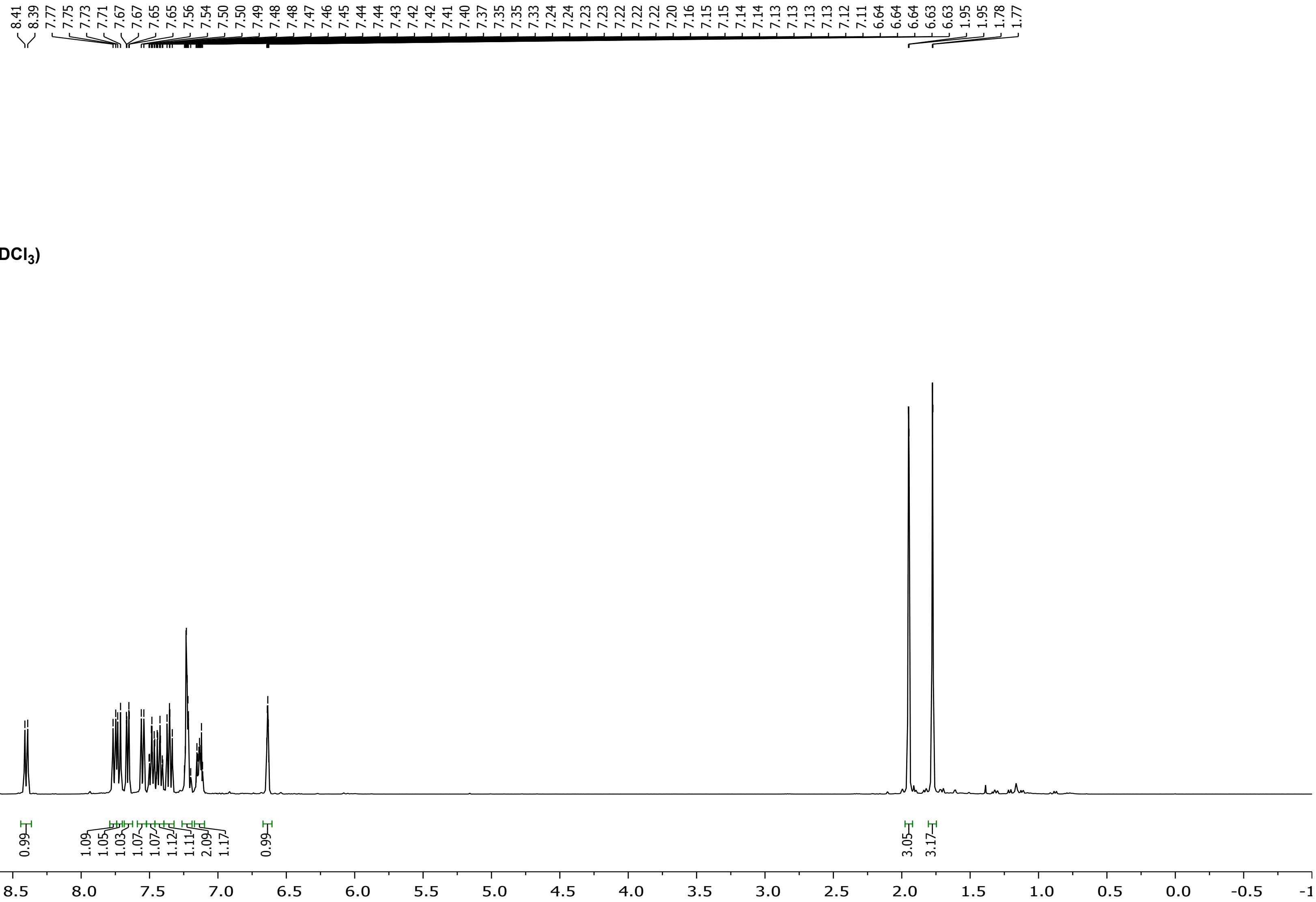

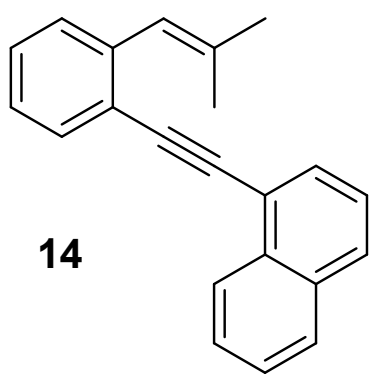

14

^{13}C-NMR (101MHz, $CDCl_3$)

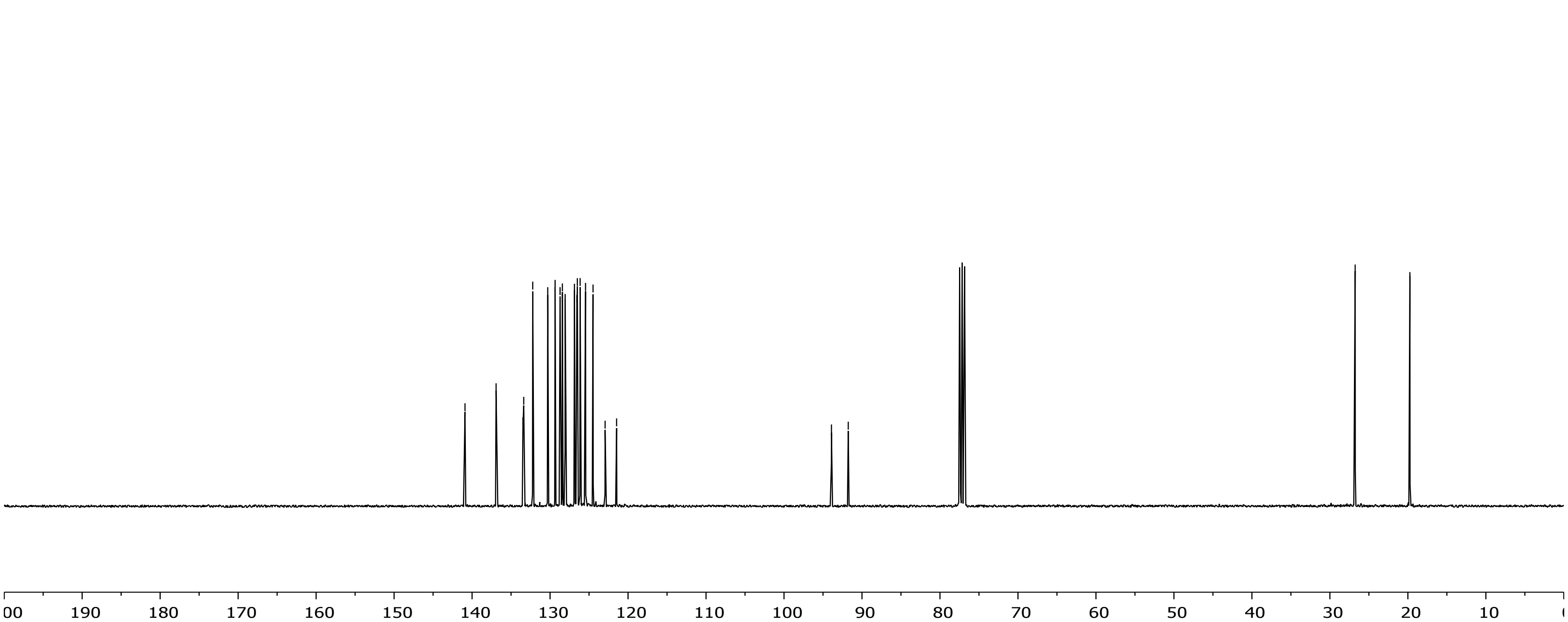

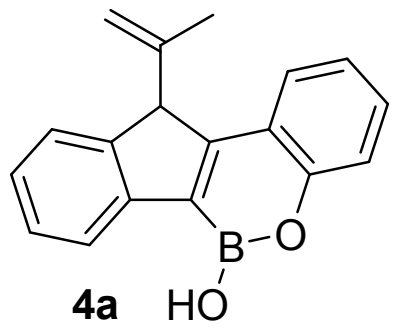

4a

^{1}H-NMR (400 MHz, Acetone-*d6*)

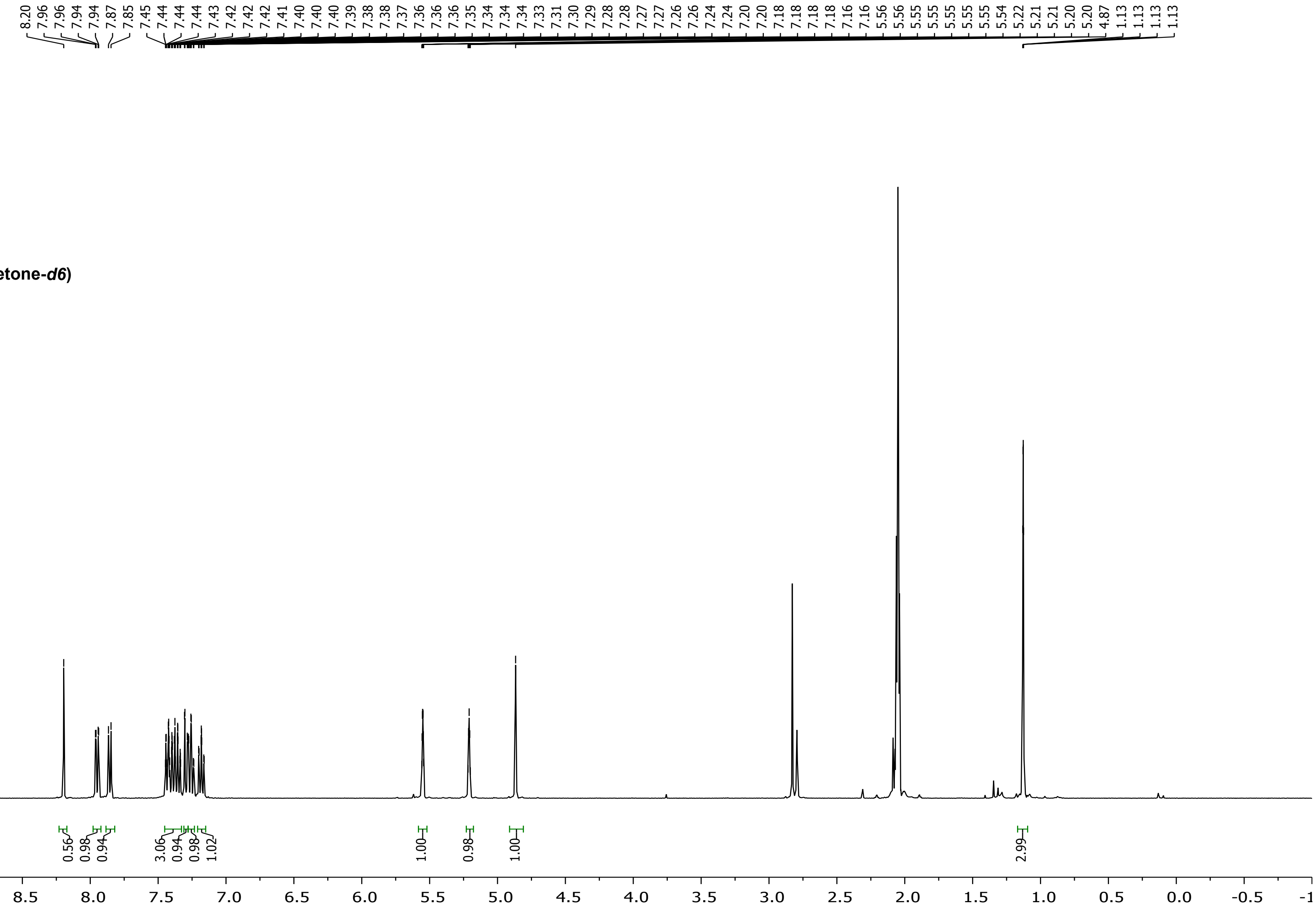

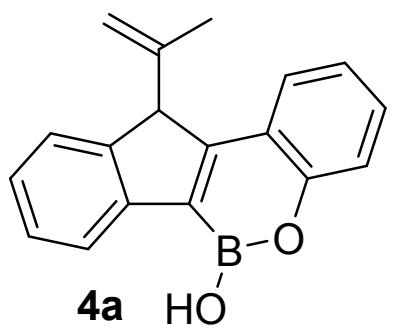

4a

^{11}B-NMR (128 MHz, Acetone-*d6*)

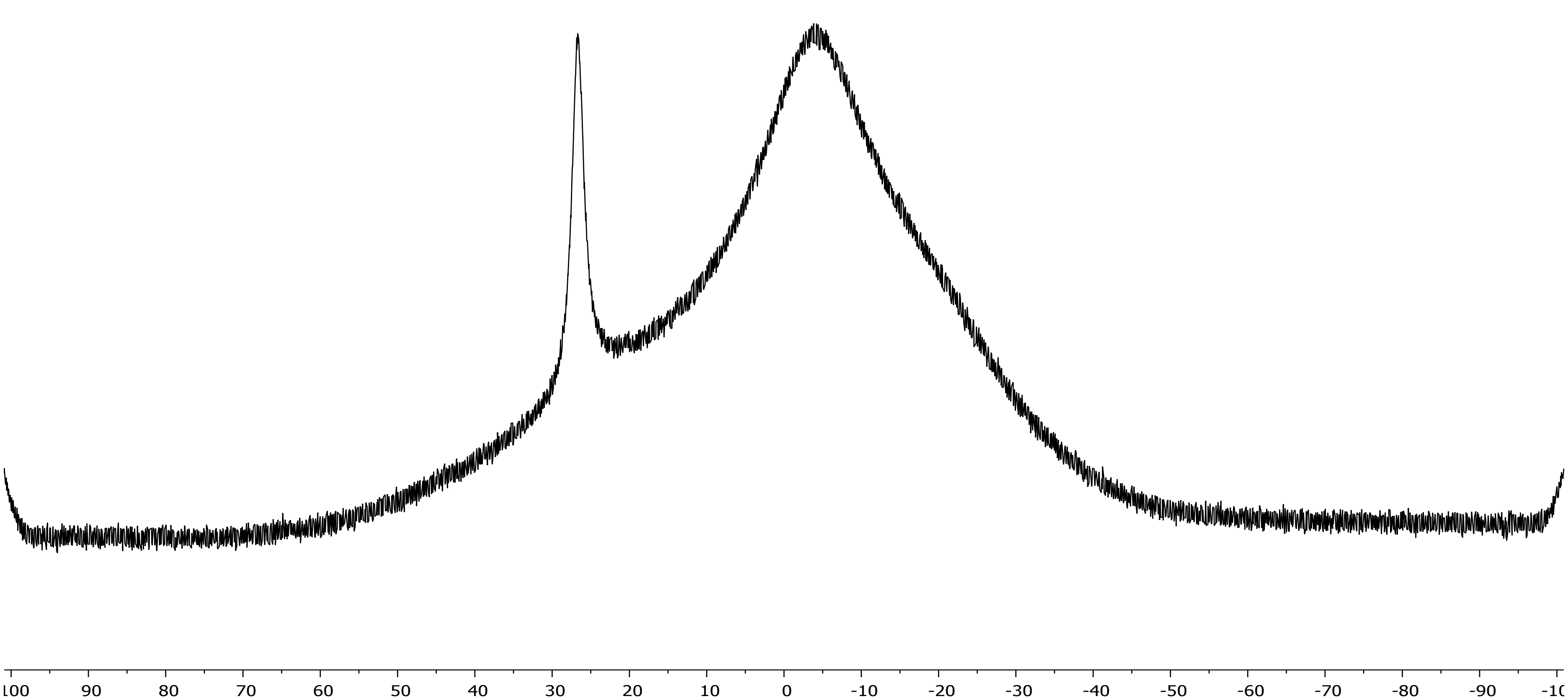

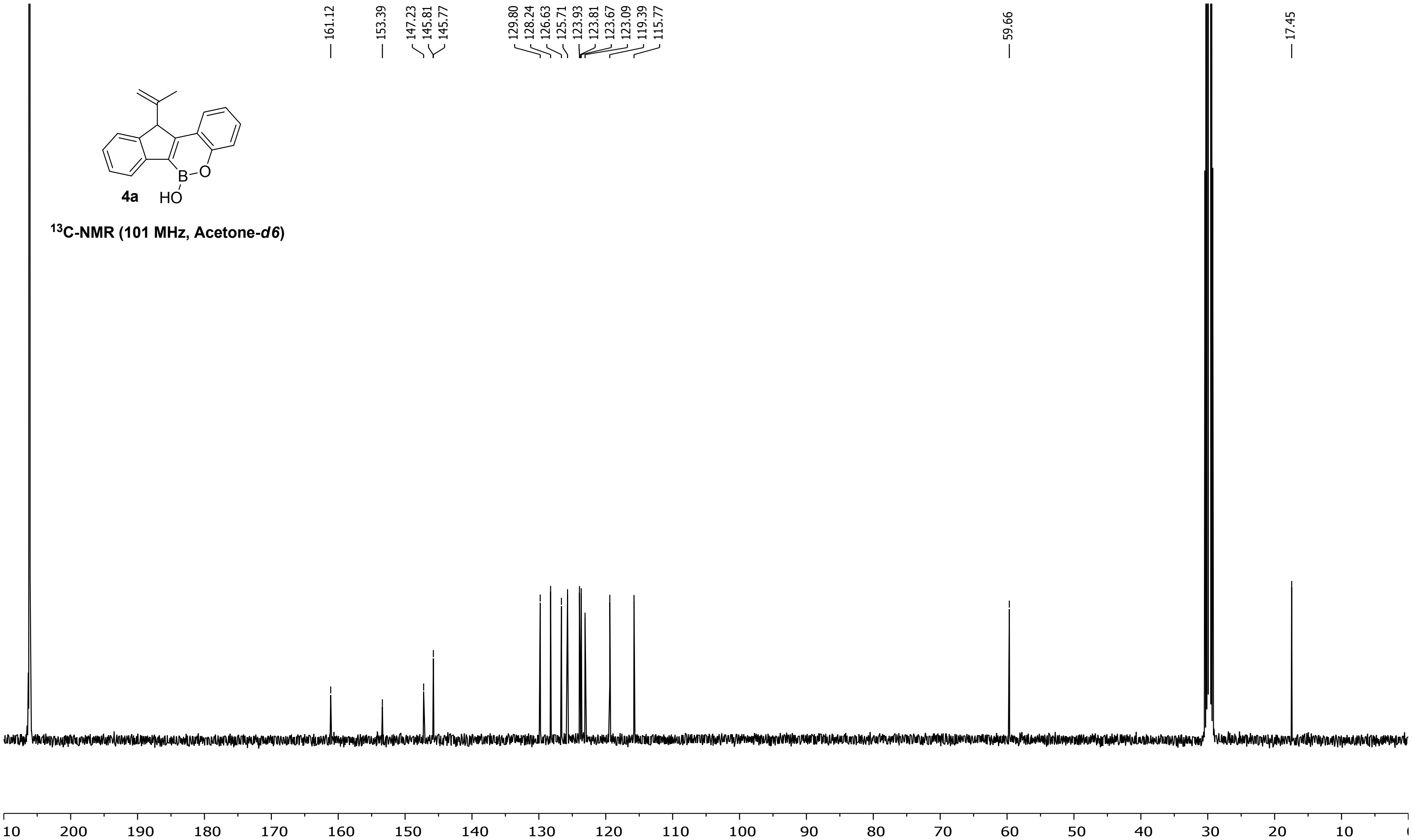

161.12
153.39
147.23
145.81
145.77
129.80
128.24
126.63
125.71
123.93
123.81
123.67
123.09
119.39
115.77
59.66
17.45
4a
B
O
HO
13C-NMR (101 MHz, Acetone-d6)
10 200 190 180 170 160 150 140 130 120 110 100 90 80 70 60 50 40 30 20 10

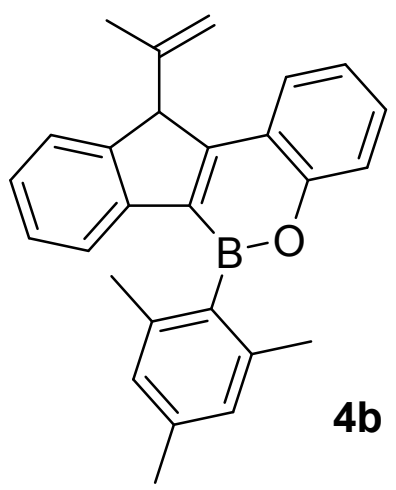

4b

^{1}H-NMR (400 MHz, $CDCl_3$)

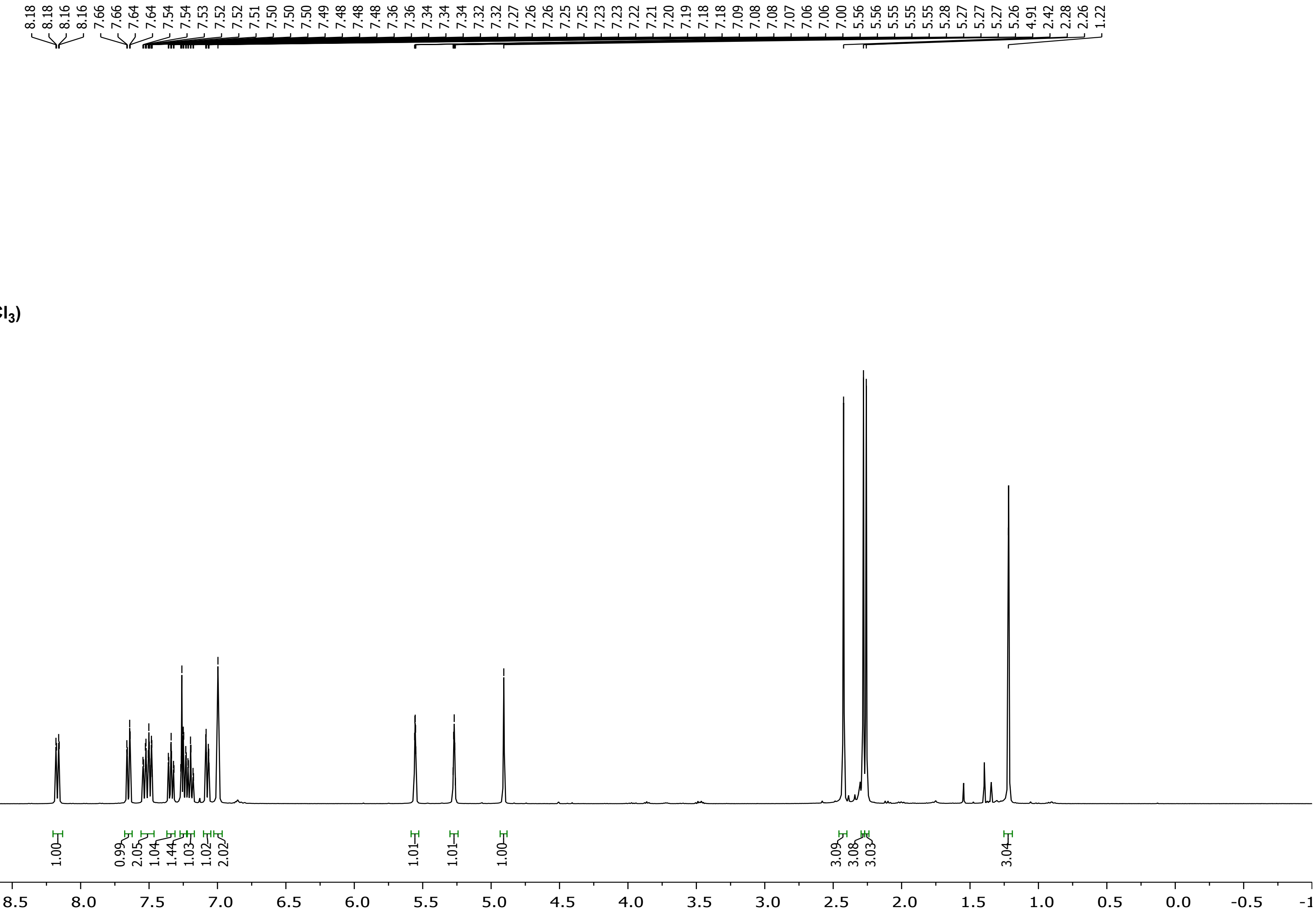

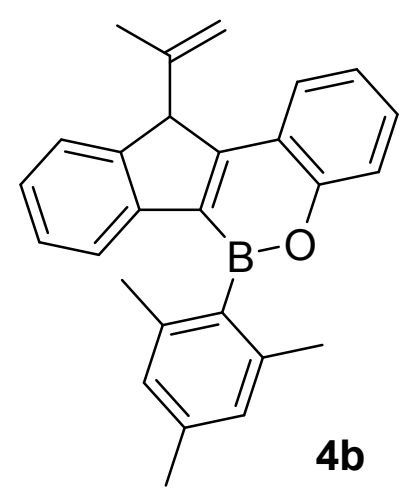

4b

^{11}B-NMR (128 MHz, $CDCl_3$)

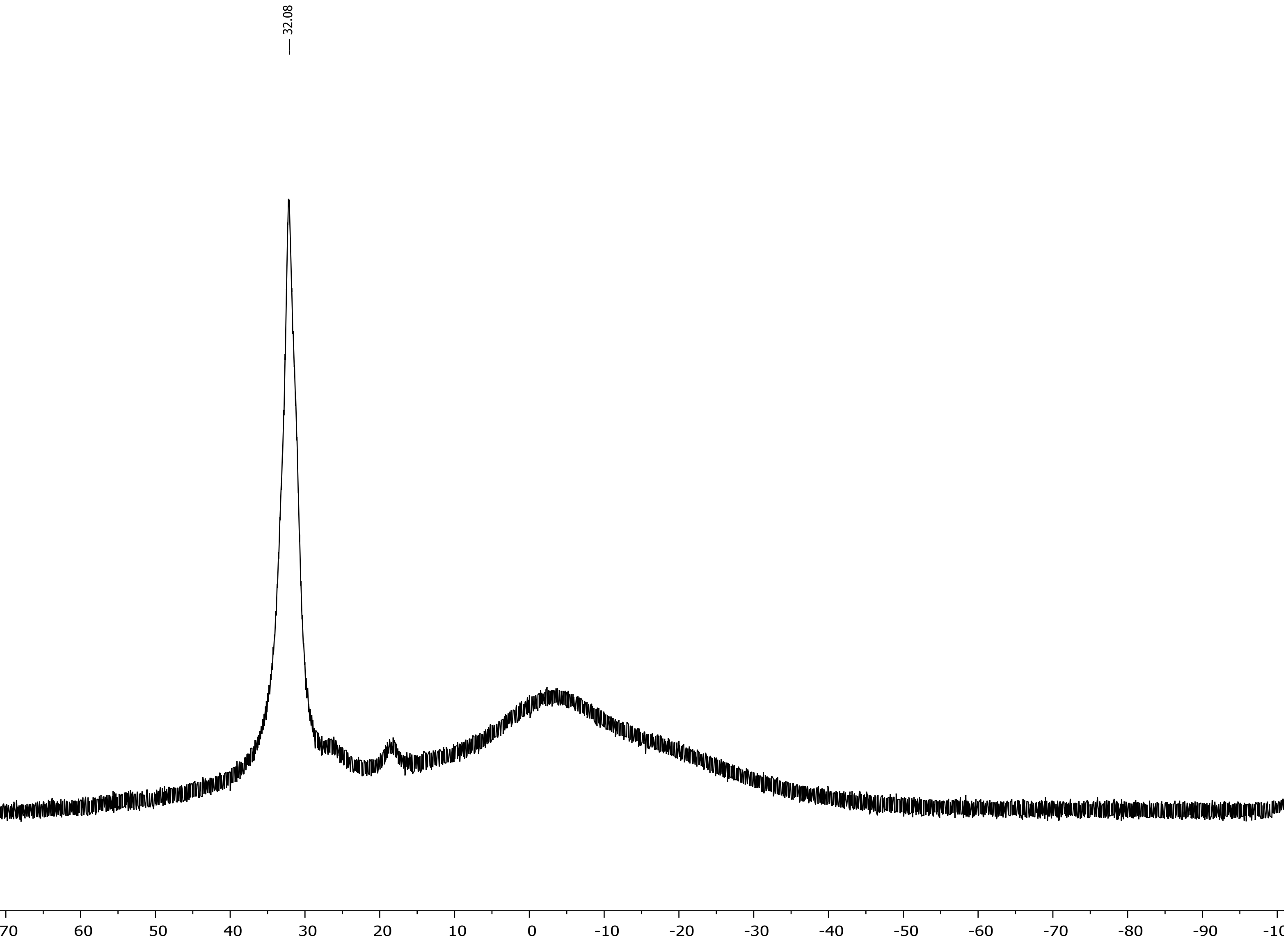

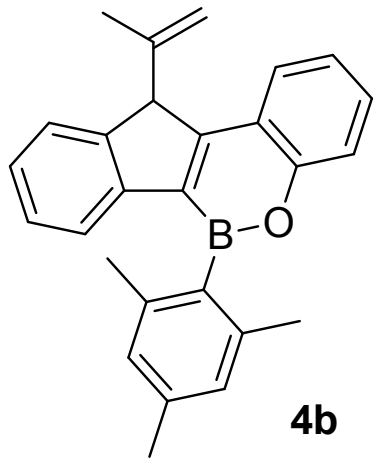

4b

^{13}C-NMR (101 MHz, CDCl$_3$)

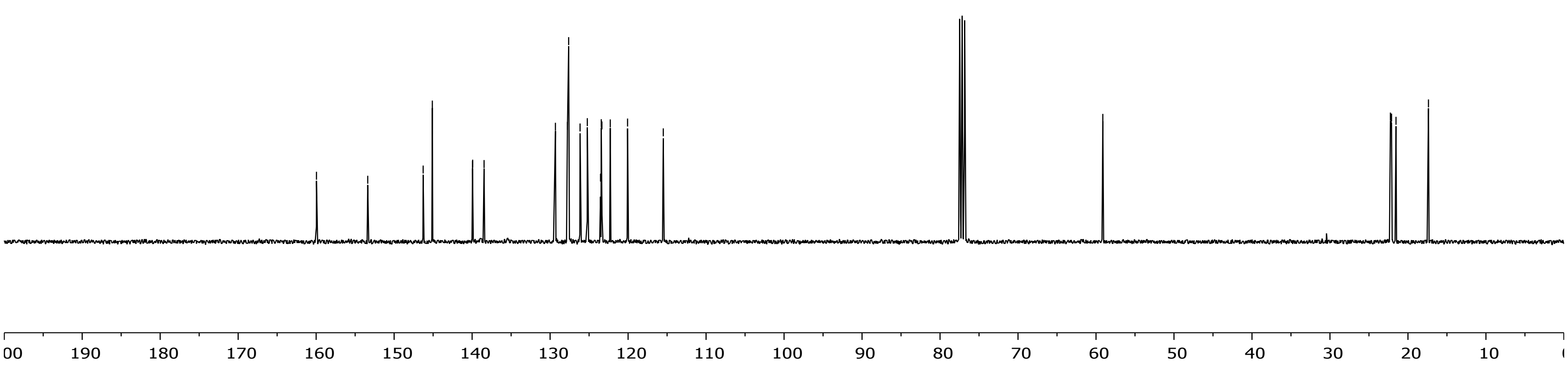

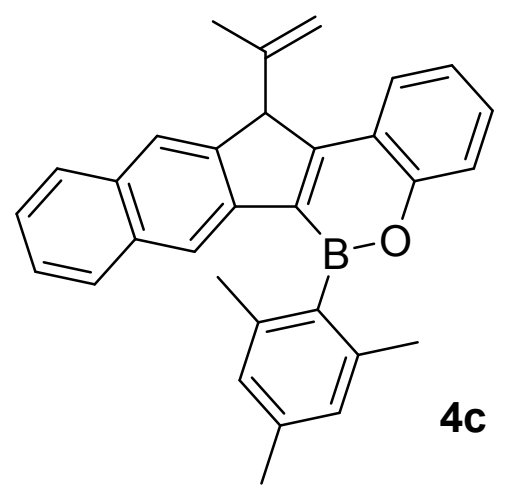

4c

^{1}H-NMR (400 MHz, $CDCl_3$)

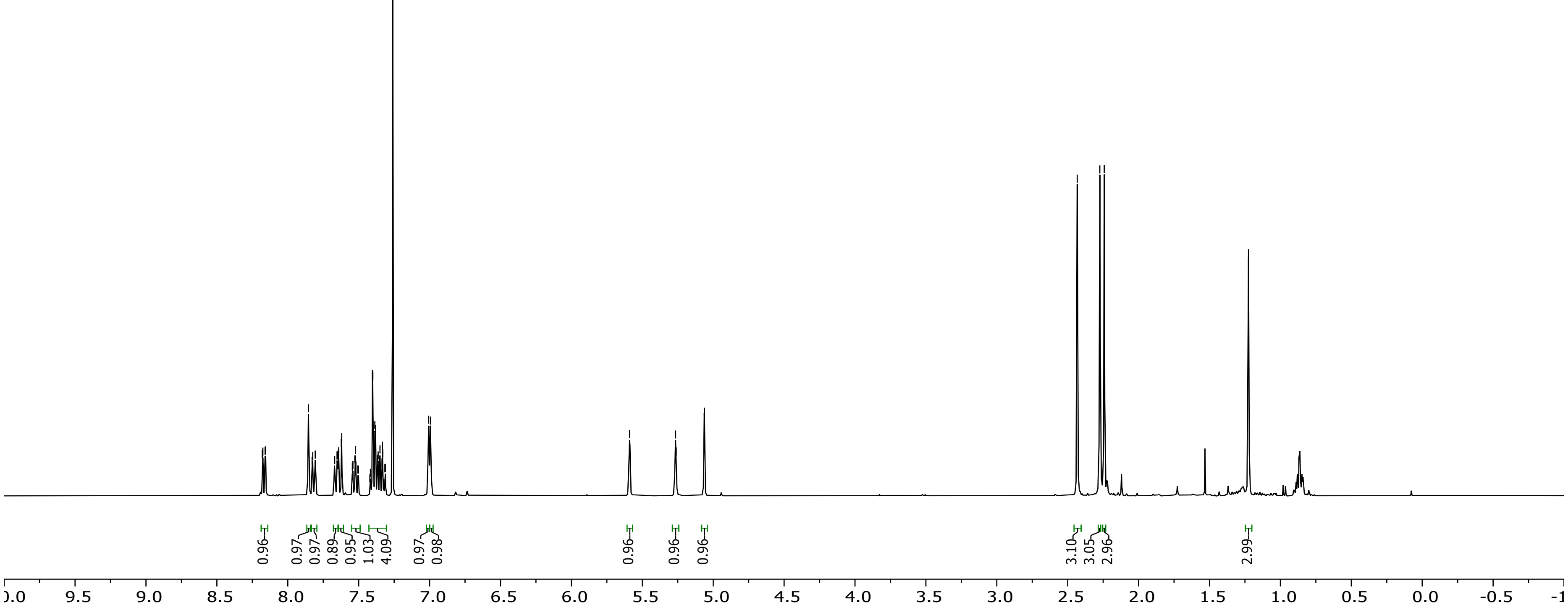

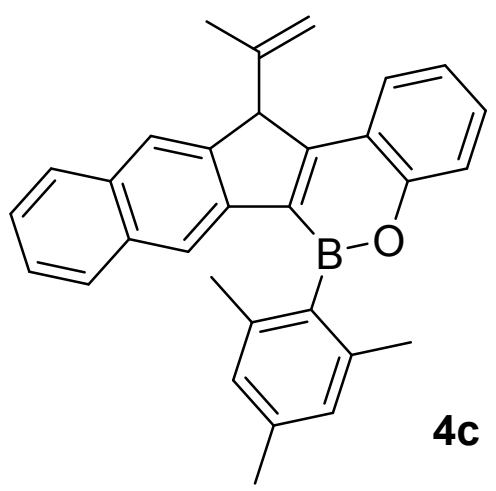

4c

^{11}B-NMR (128 MHz, $CDCl_3$)

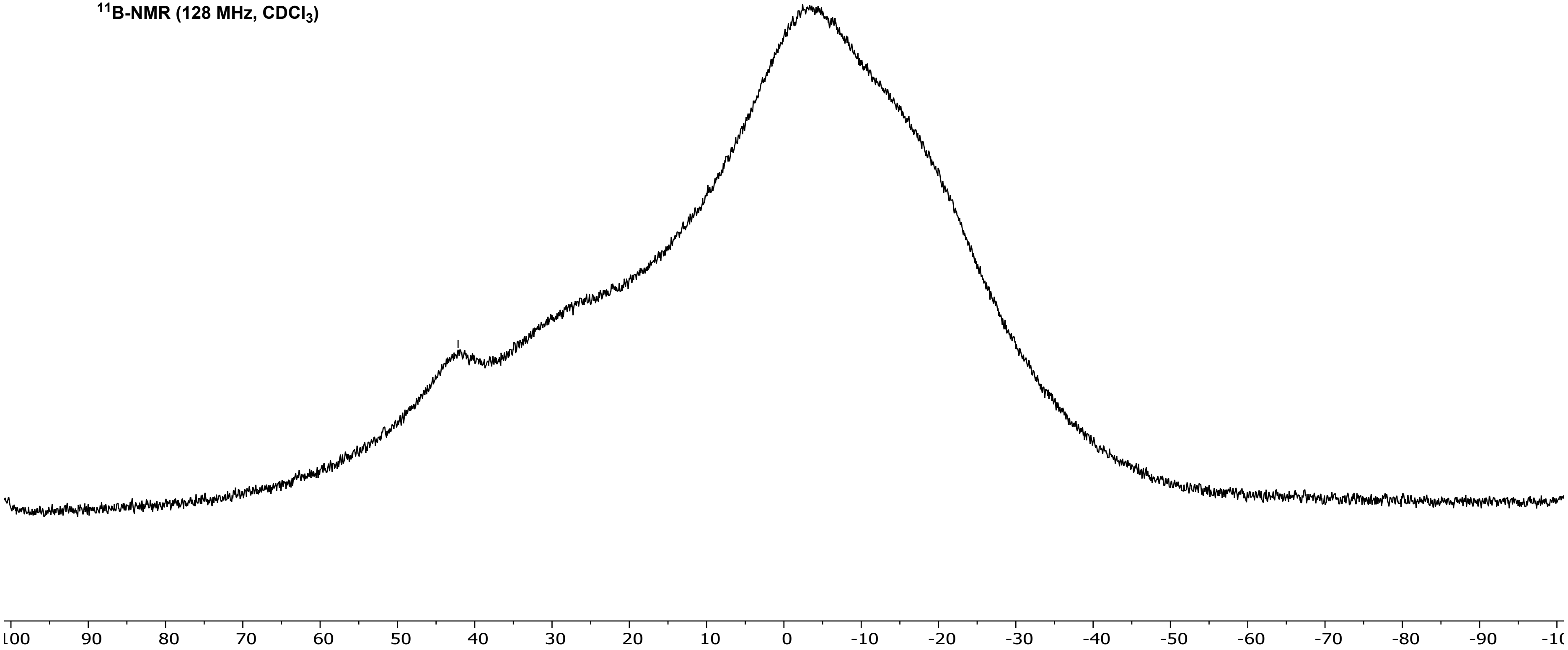

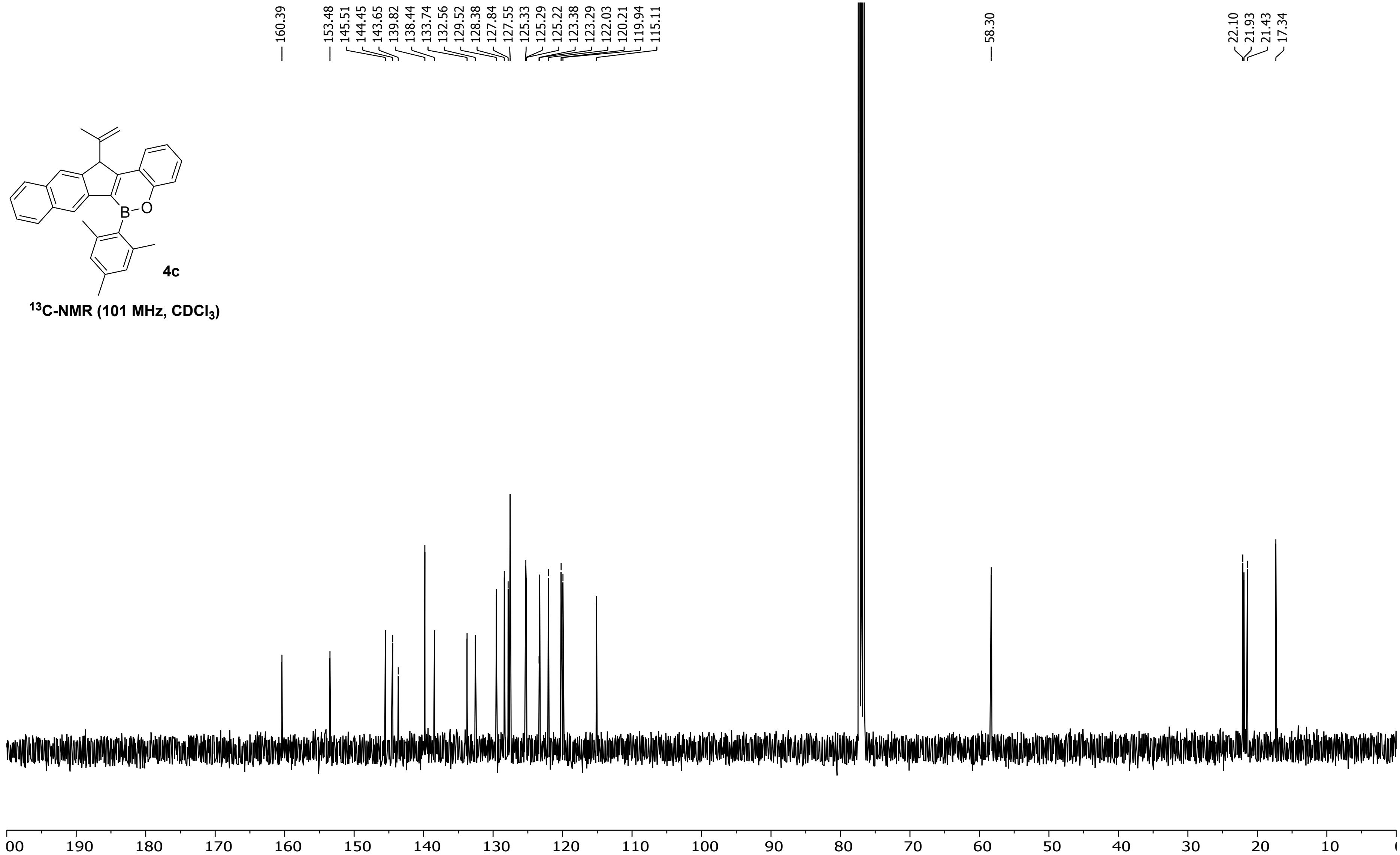
160.39
153.48
145.51
144.45
143.65
139.82
138.44
133.74
132.56
129.52
128.38
127.84
127.55
125.33
125.29
125.22
123.38
123.29
122.03
120.21
119.94
115.11
58.30
22.10
21.93
21.43
17.34
B
O
4c
^{13}C-NMR (101 MHz, $CDCl_3$)
190
180
170
160
150
140
130
120
110
100
90
80
70
60
50
40
30
20
10

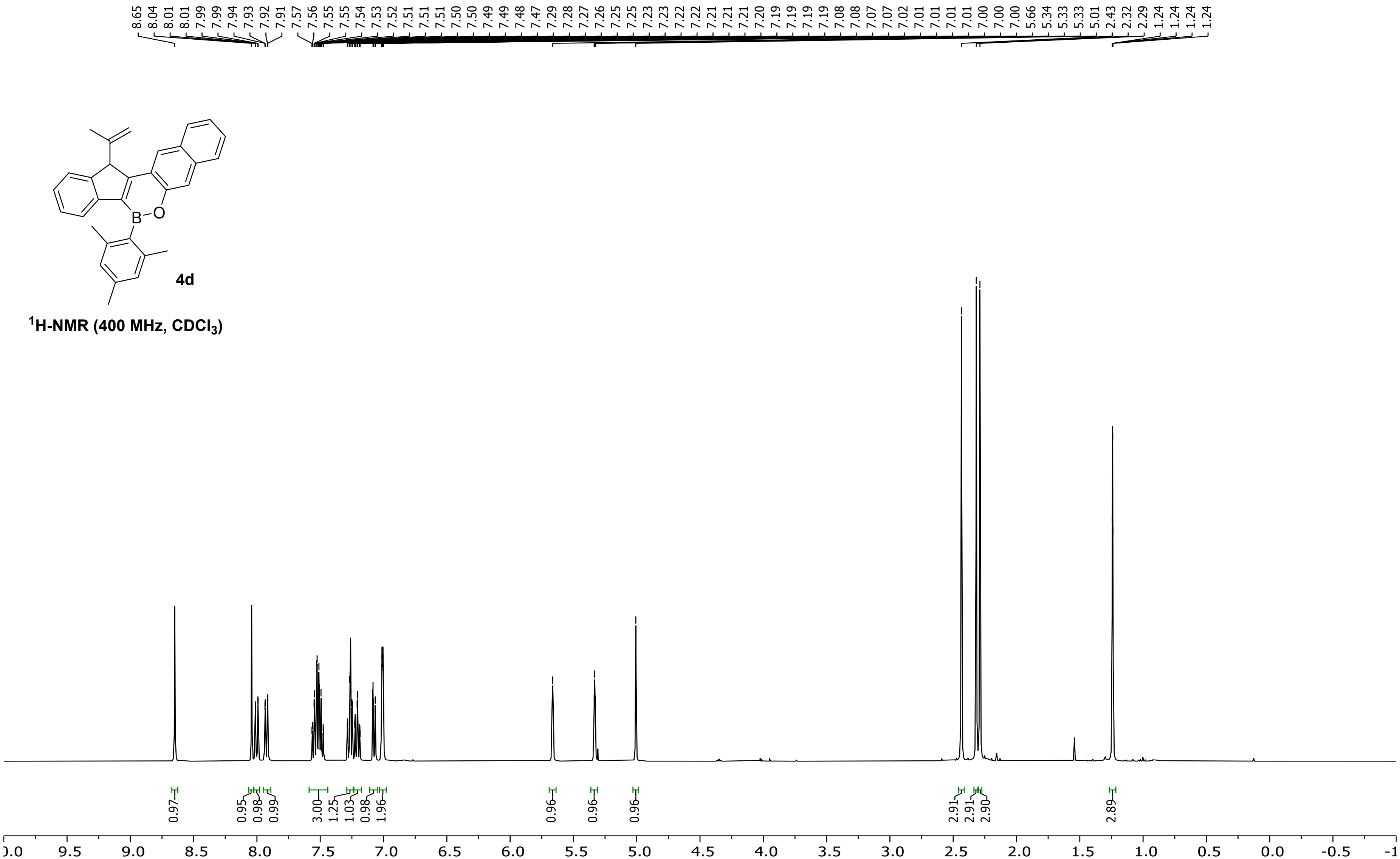

4d

^{1}H-NMR (400 MHz, $CDCl_3$)

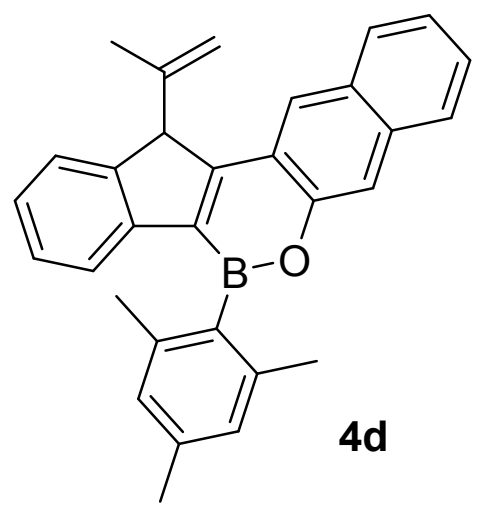

4d

^{11}B-NMR (128 MHz, $CDCl_3$)

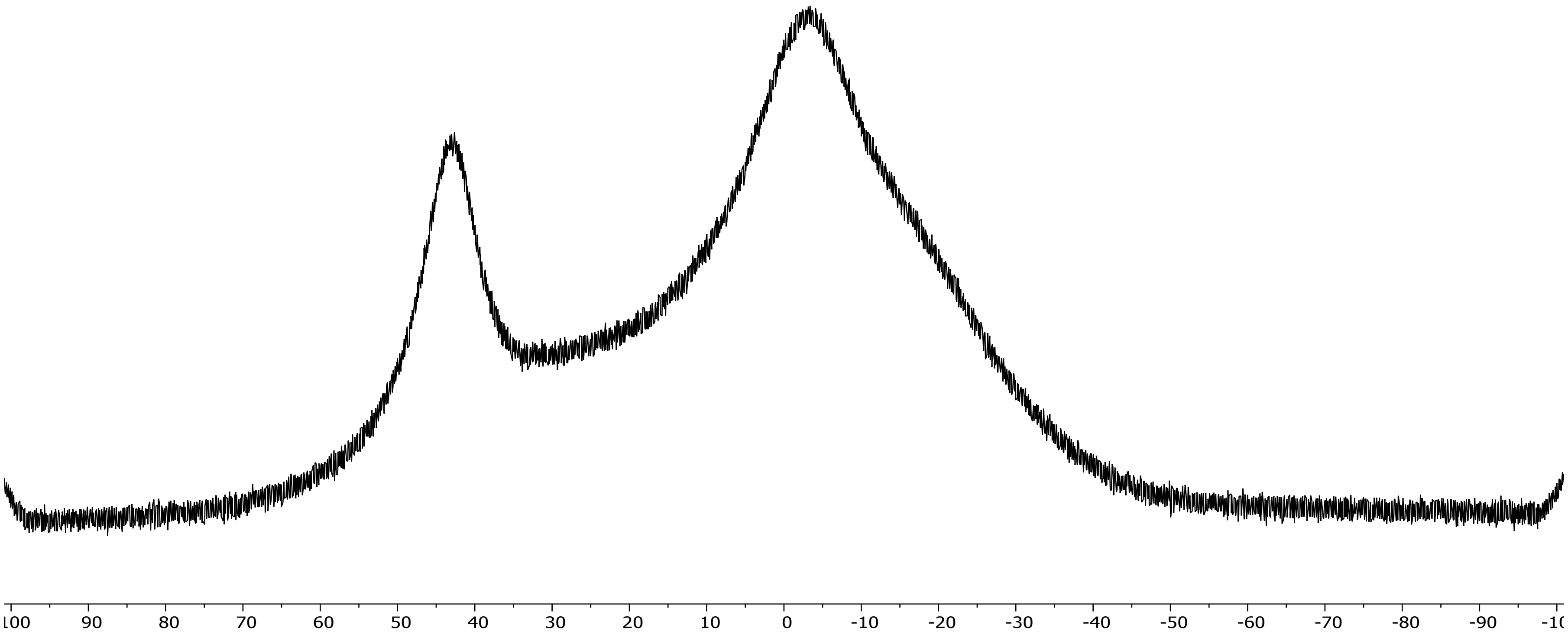

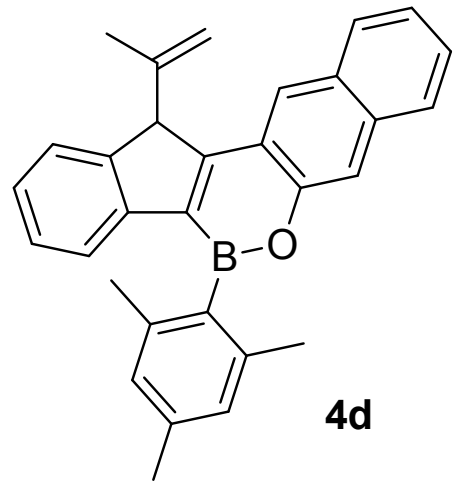

4d

^{13}C-NMR (101 MHz, CDCl$_3$)

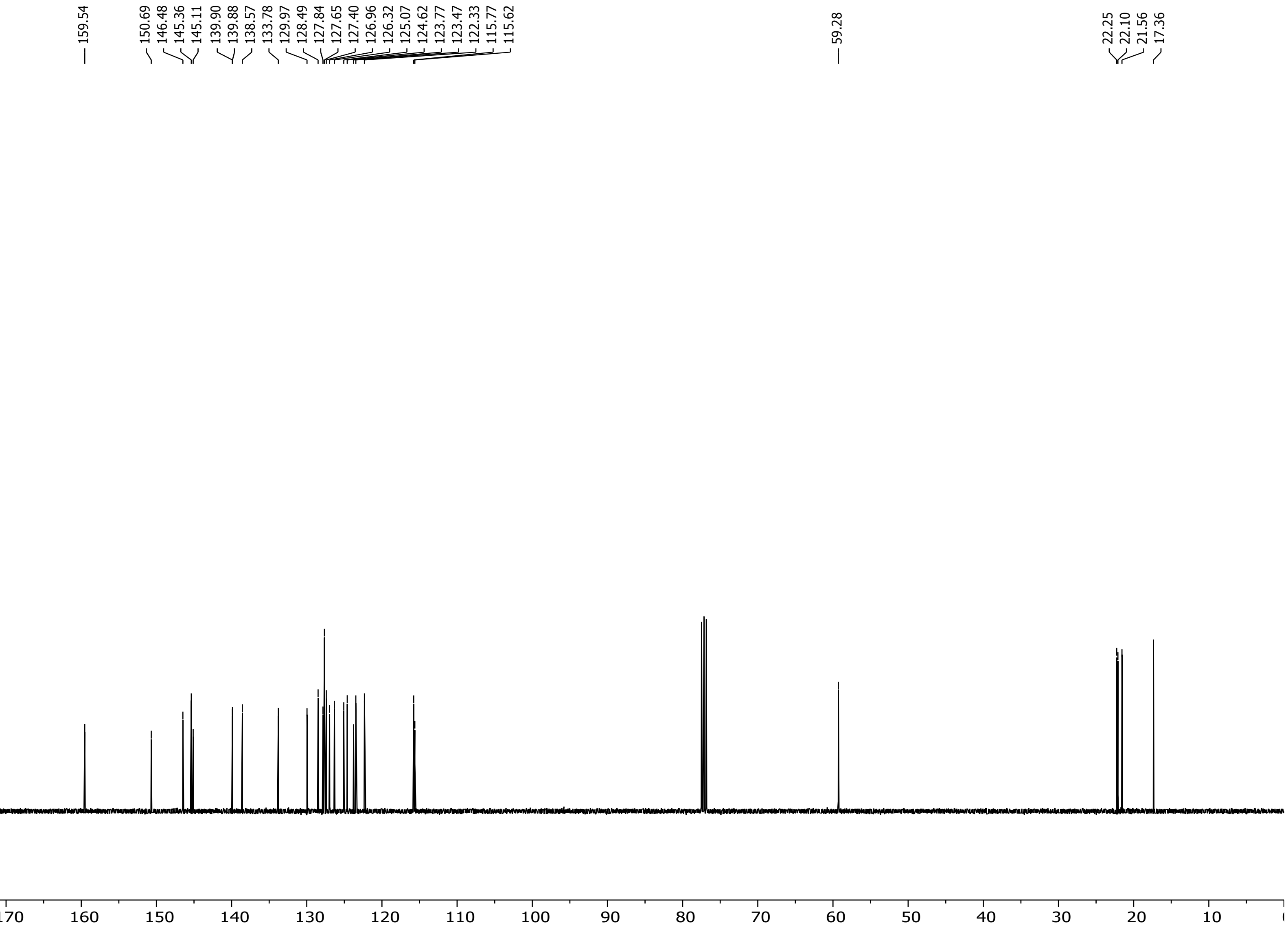

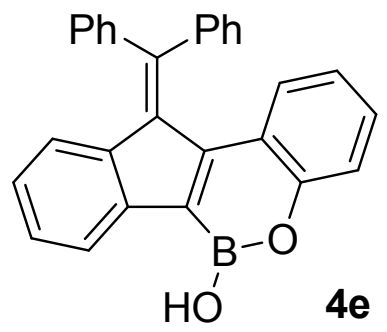

4e

^{1}H-NMR (400 MHz, Acetone-*d6*)

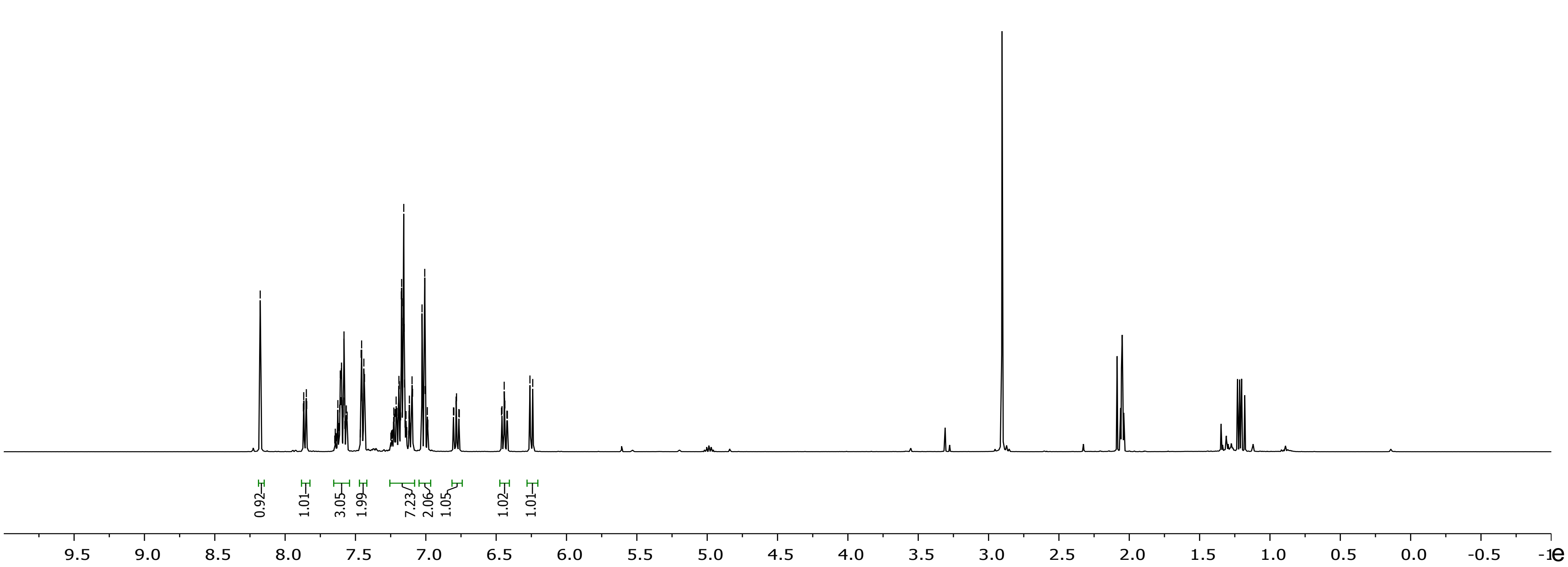

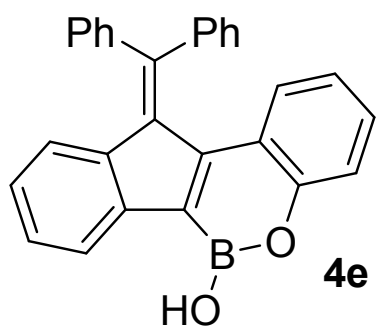

4e

^{11}B-NMR (128 MHz, Acetone-*d6*)

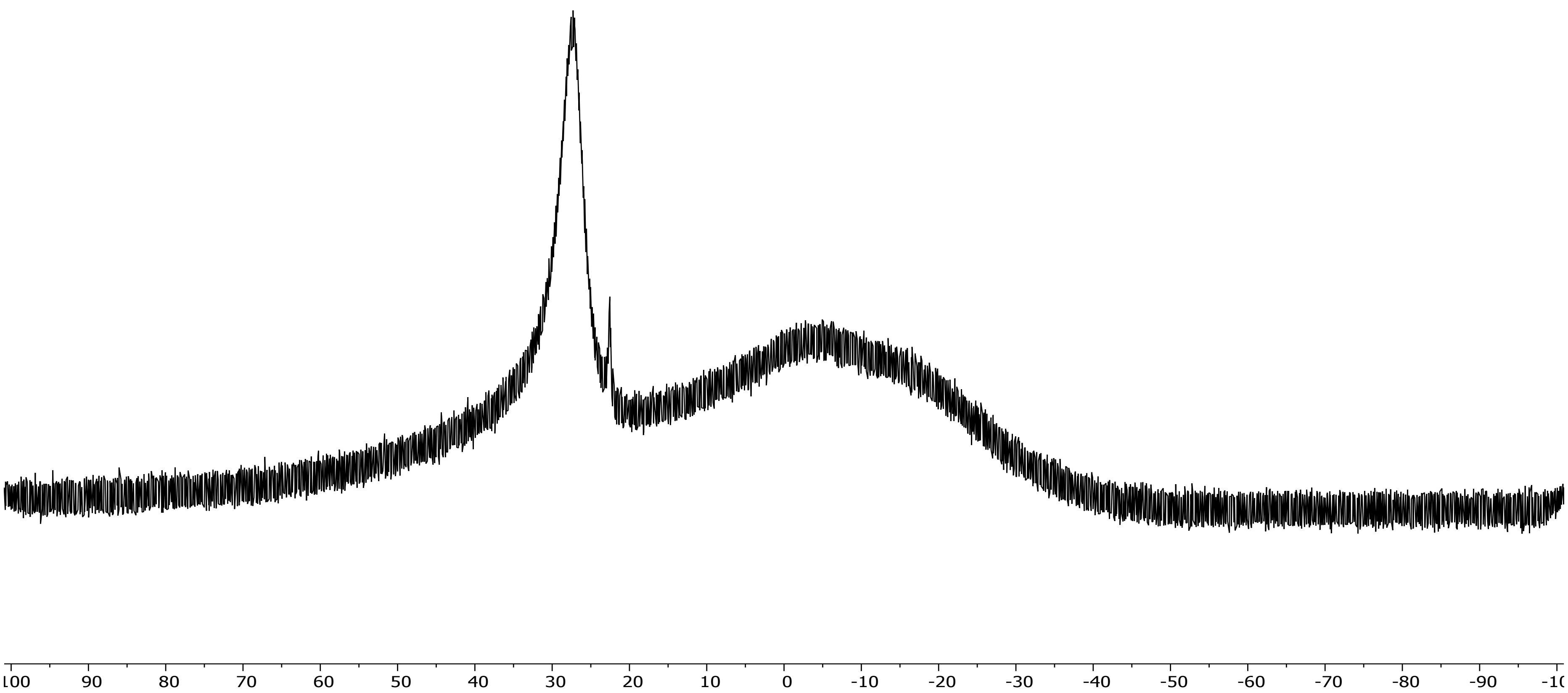

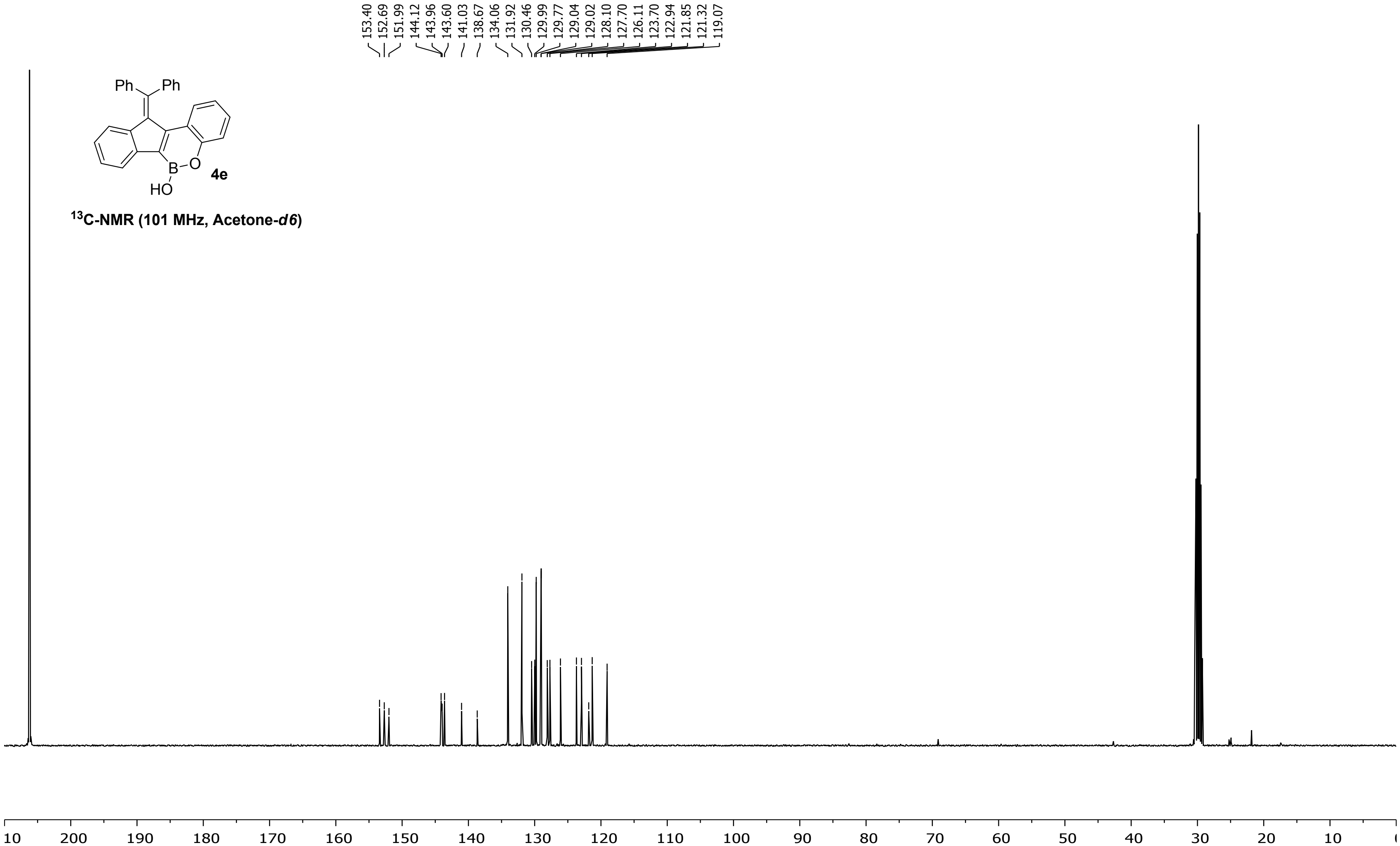

^{13}C-NMR (101 MHz, Acetone-*d6*)

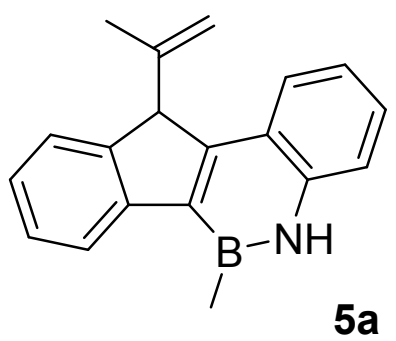

5a

^{1}H-NMR (400 MHz, $CDCl_3$)

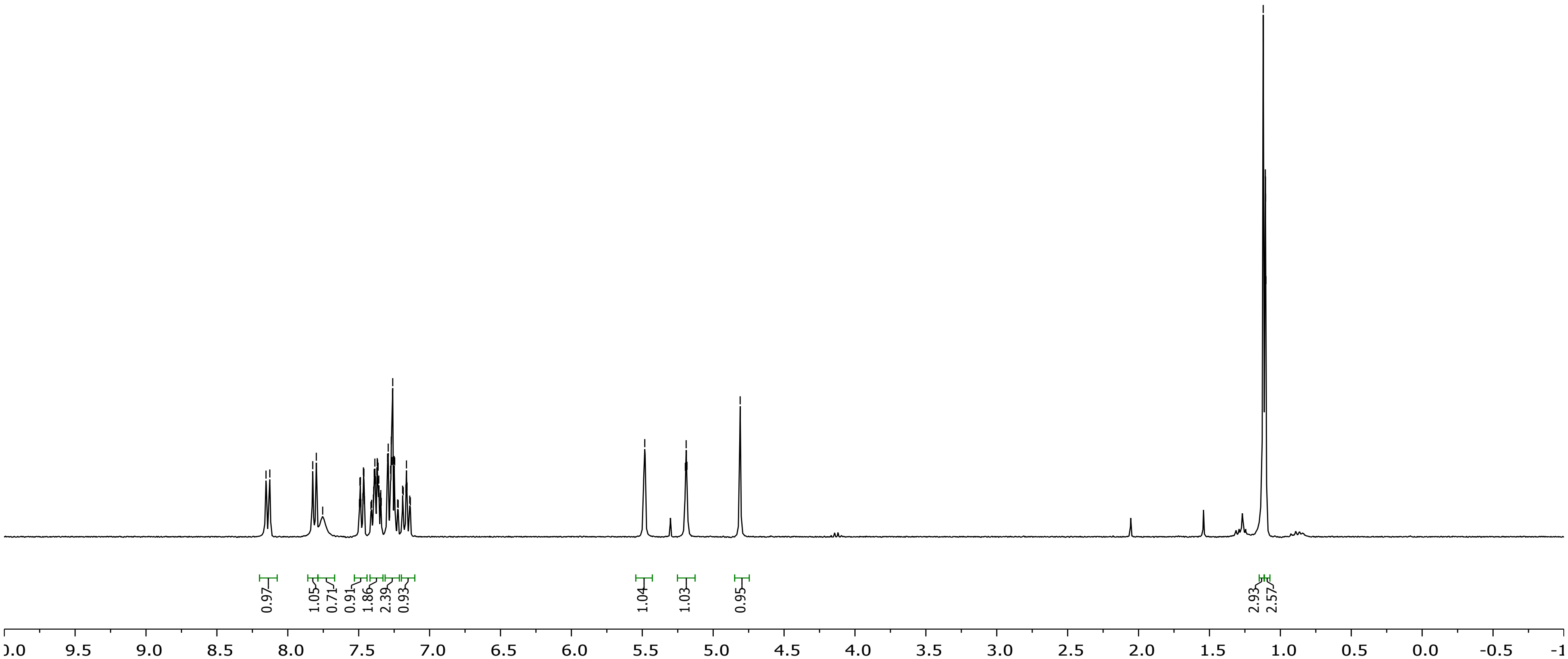

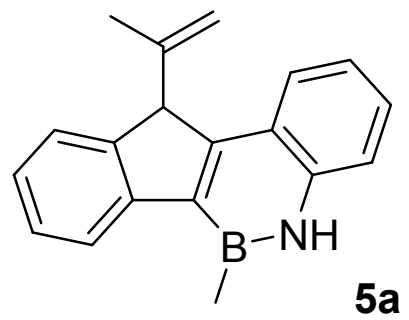

5a

^{11}B-NMR (128 MHz, $CDCl_3$)

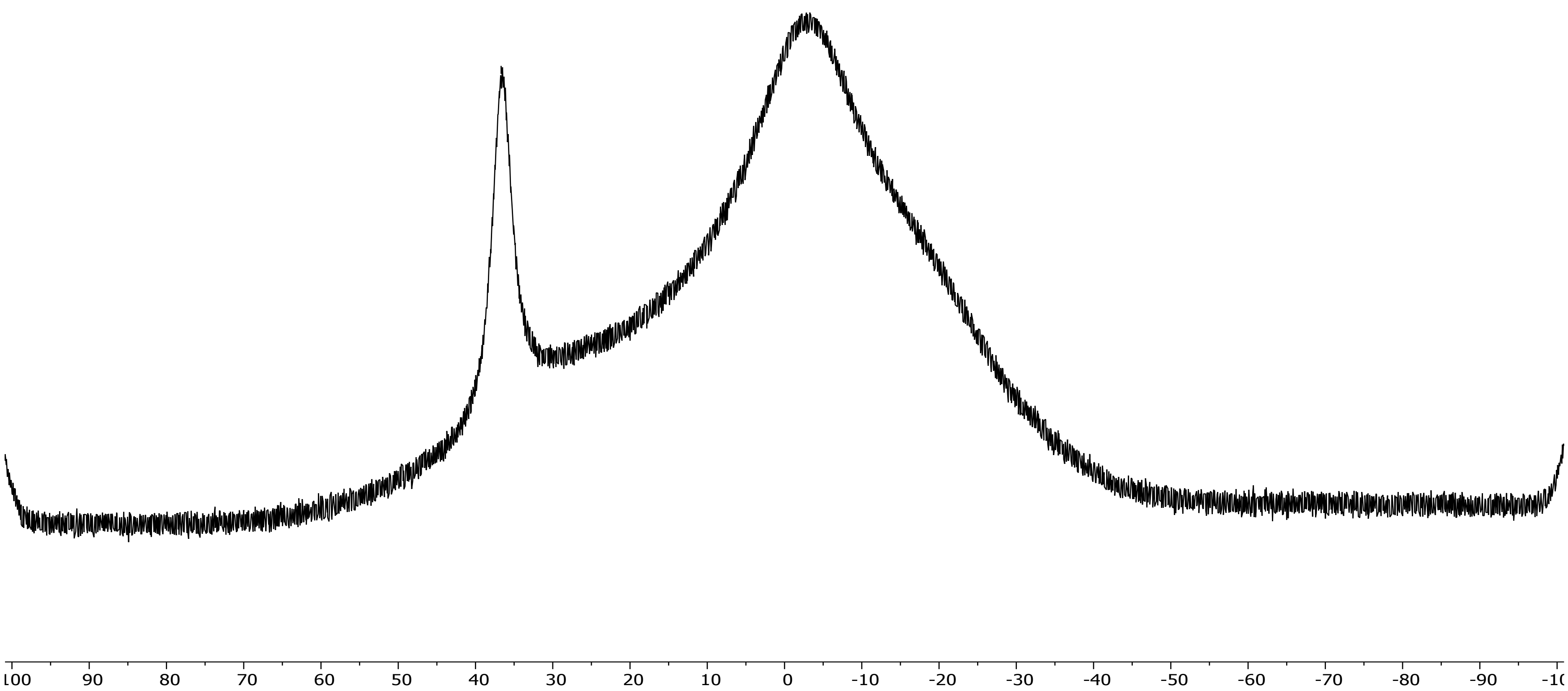

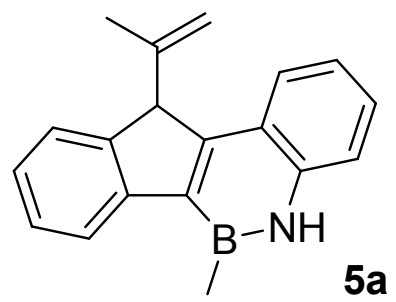

5a

^{13}C-NMR (101 MHz, $CDCl_3$)

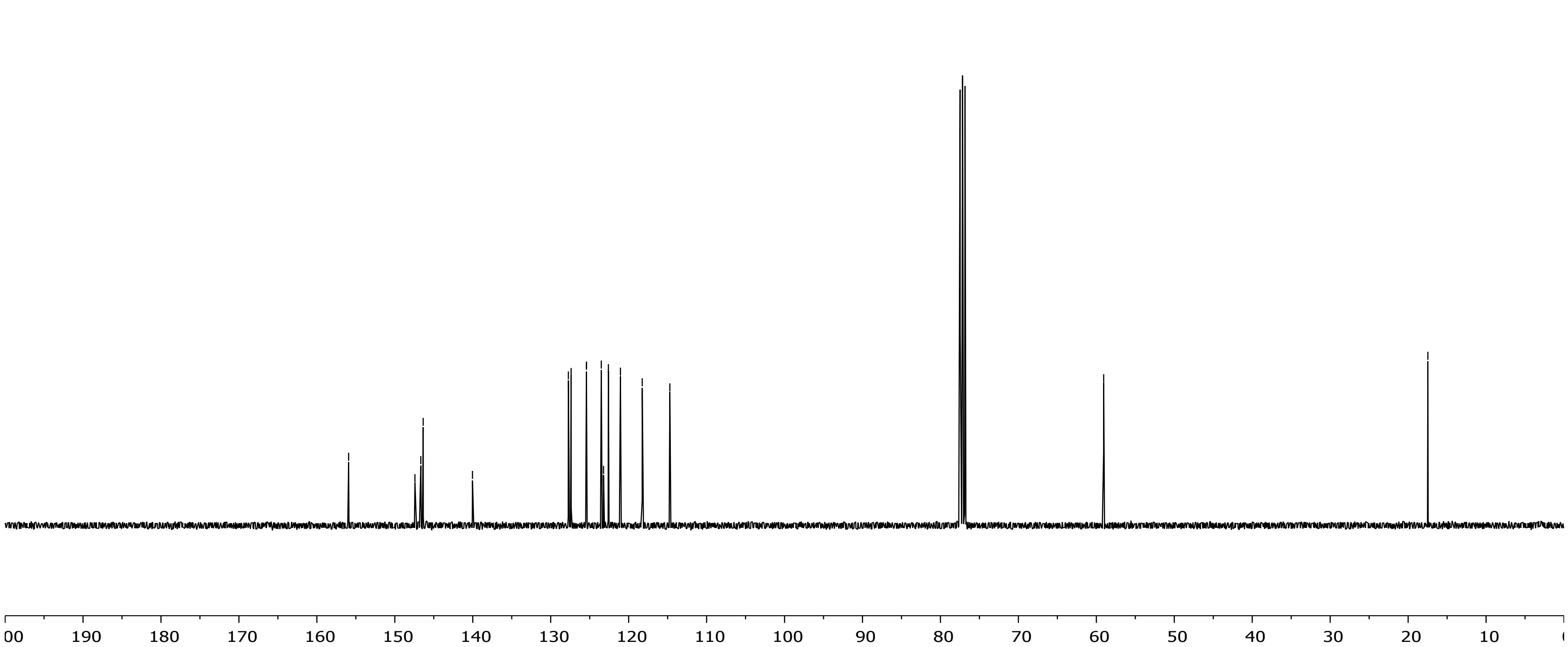

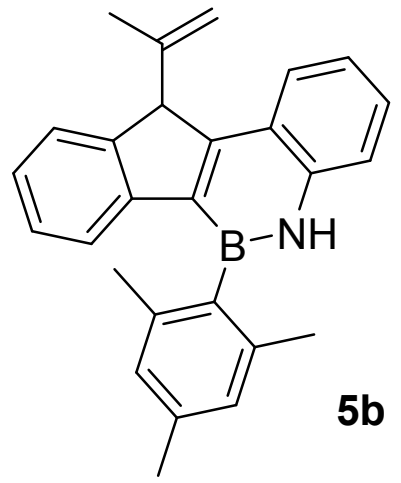

5b

^{1}H-NMR (400 MHz, CDCl$_3$)

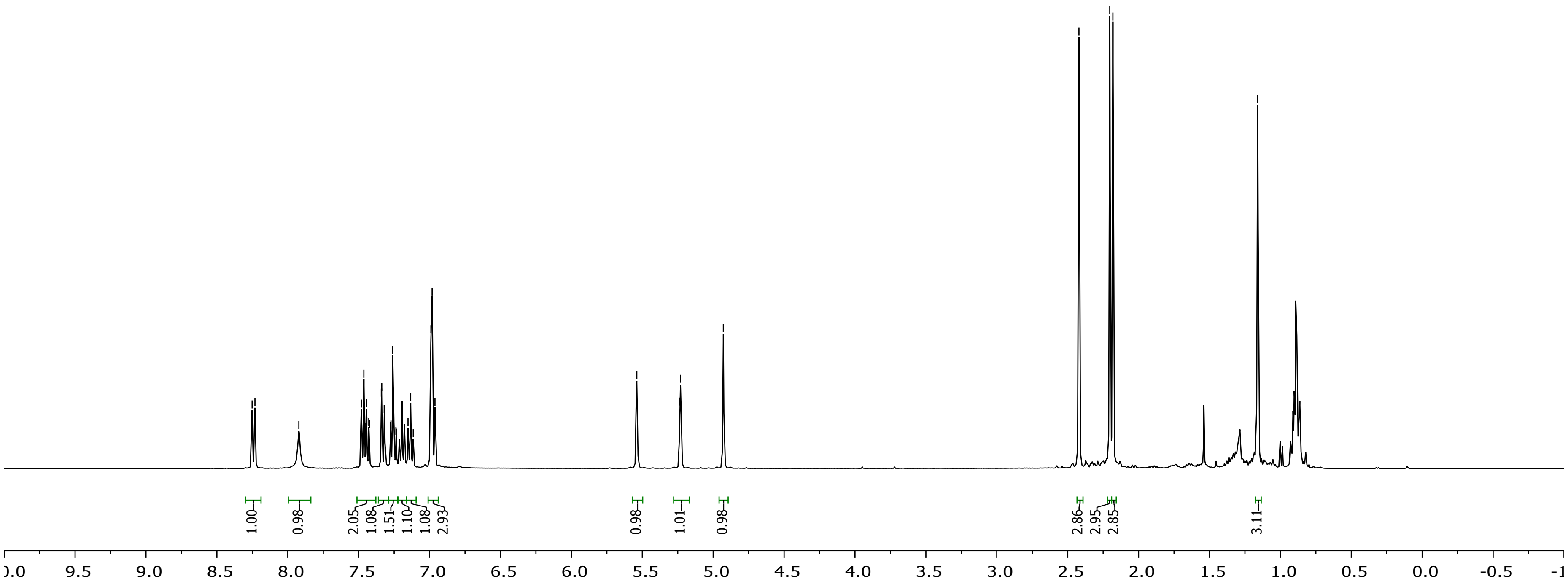

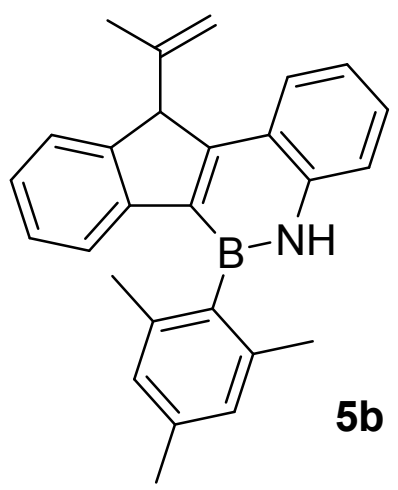

5b

^{11}B-NMR (128 MHz, $CDCl_3$)

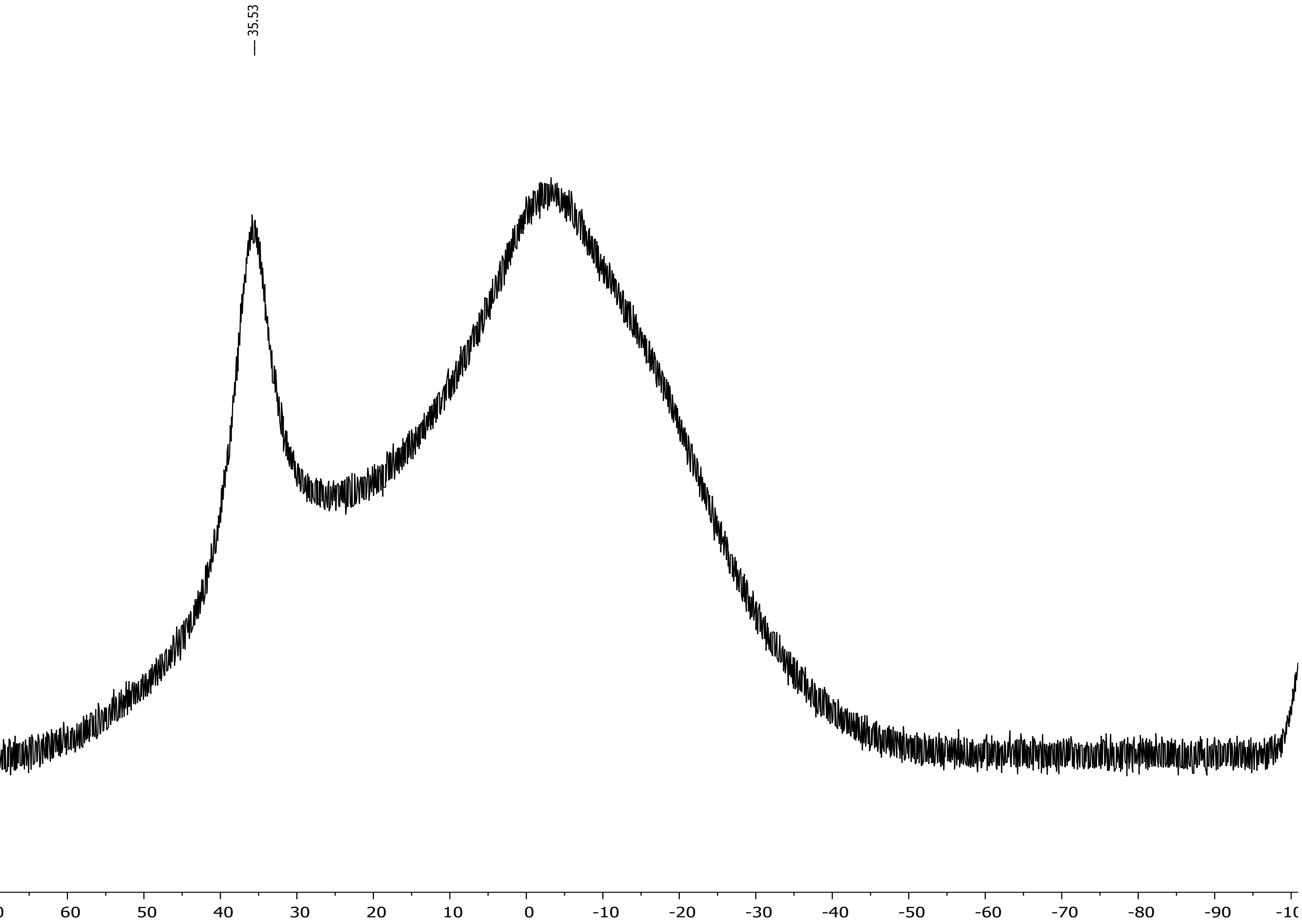

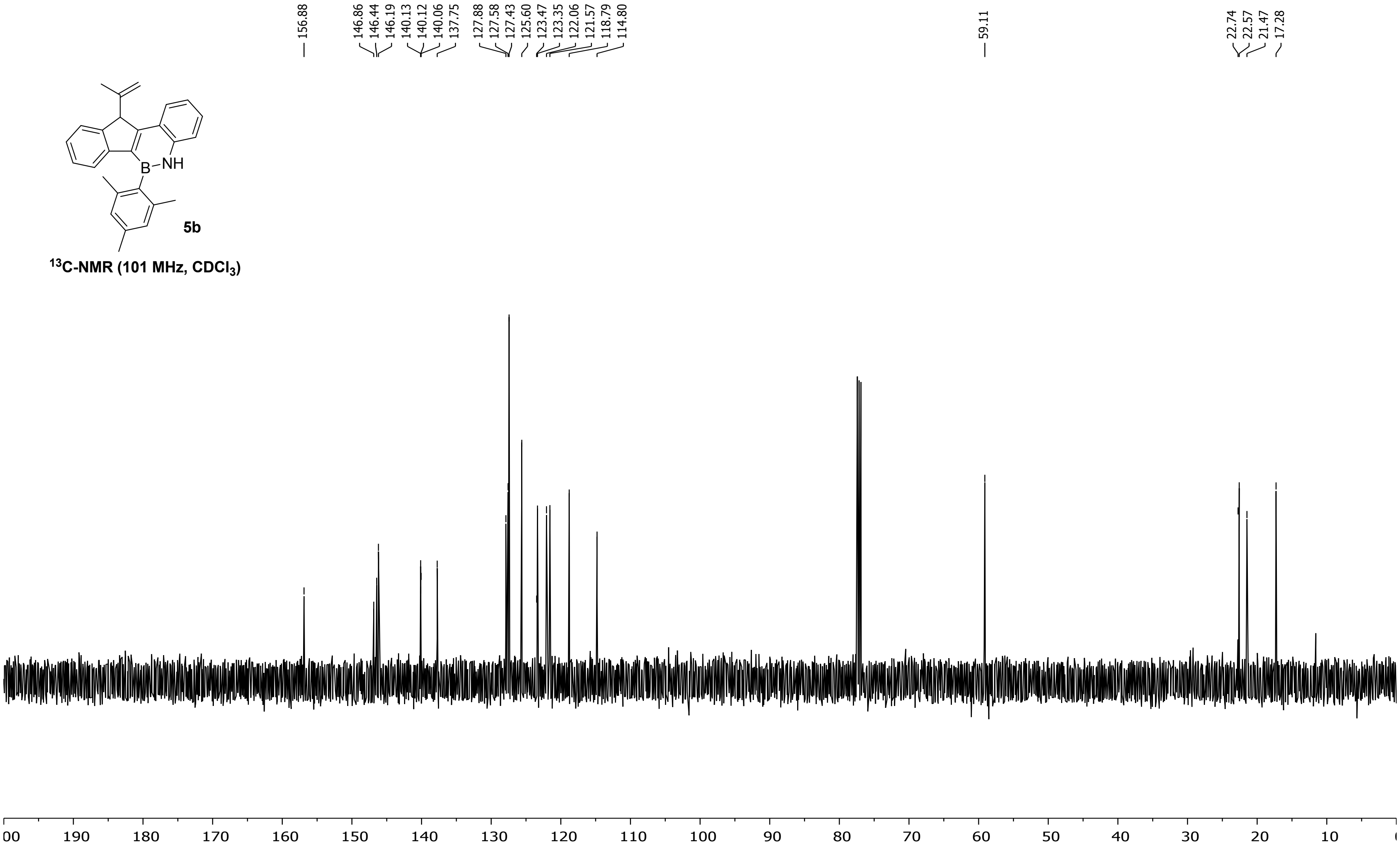

^{13}C-NMR (101 MHz, $CDCl_3$)

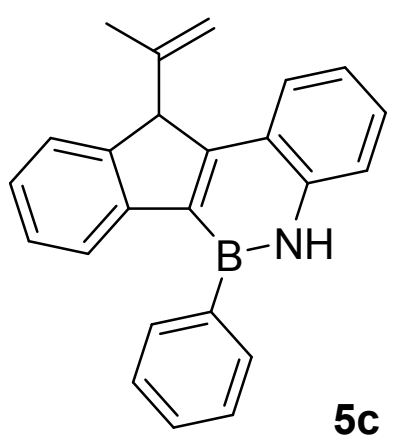

5c

^{1}H-NMR (400 MHz, $CDCl_3$)

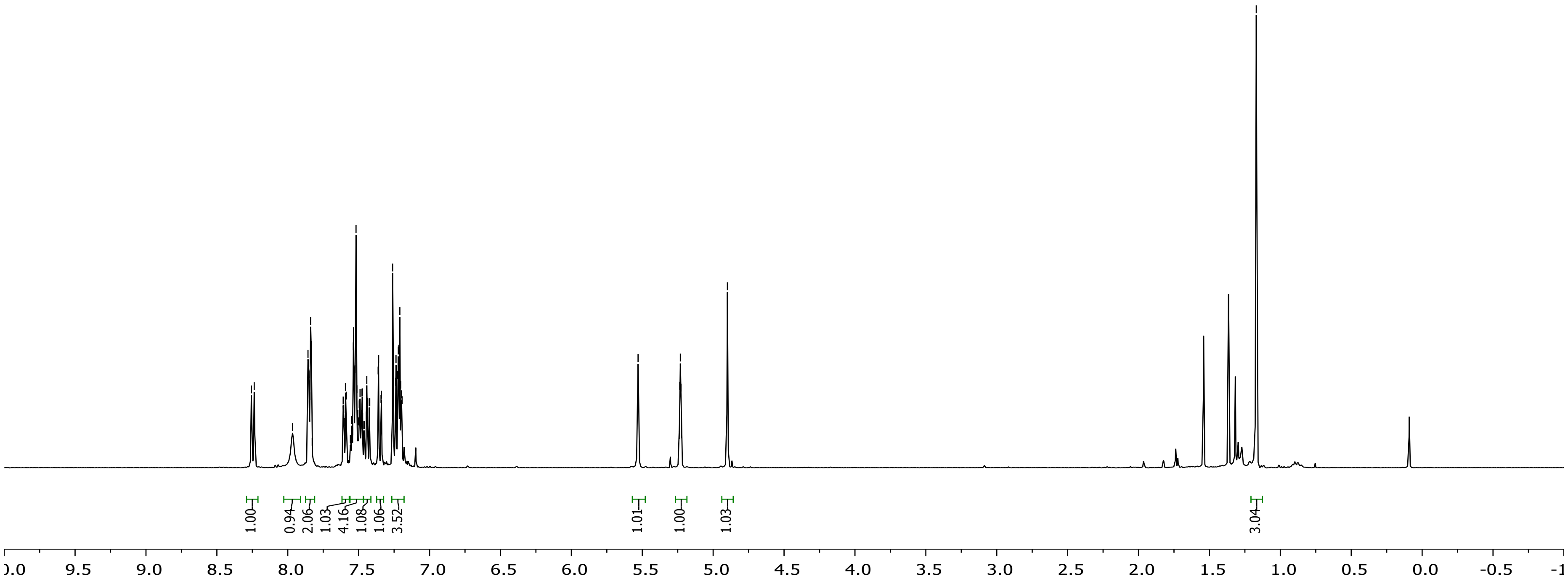

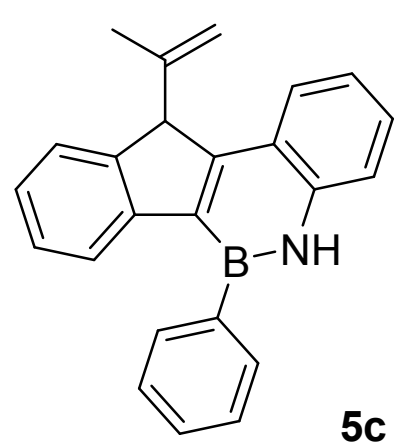

5c

^{11}B-NMR (128 MHz, $CDCl_3$)

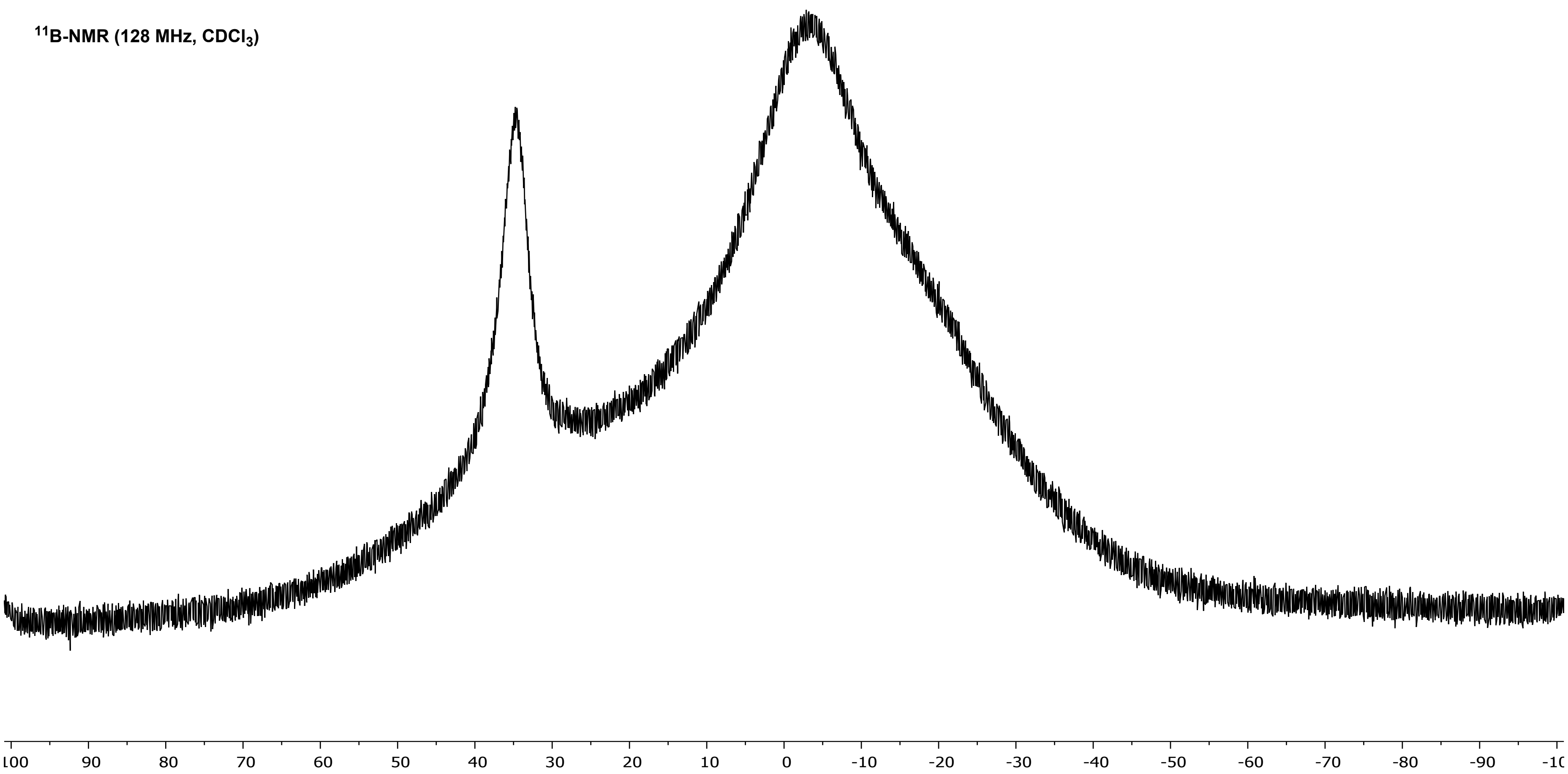

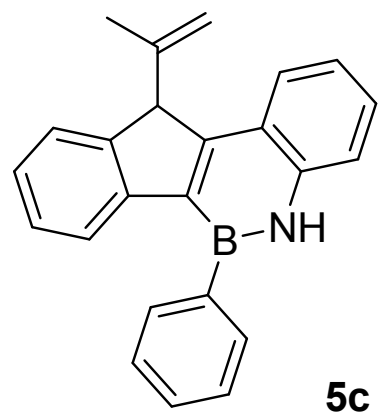

5c

^{13}C-NMR (101 MHz, $CDCl_3$)

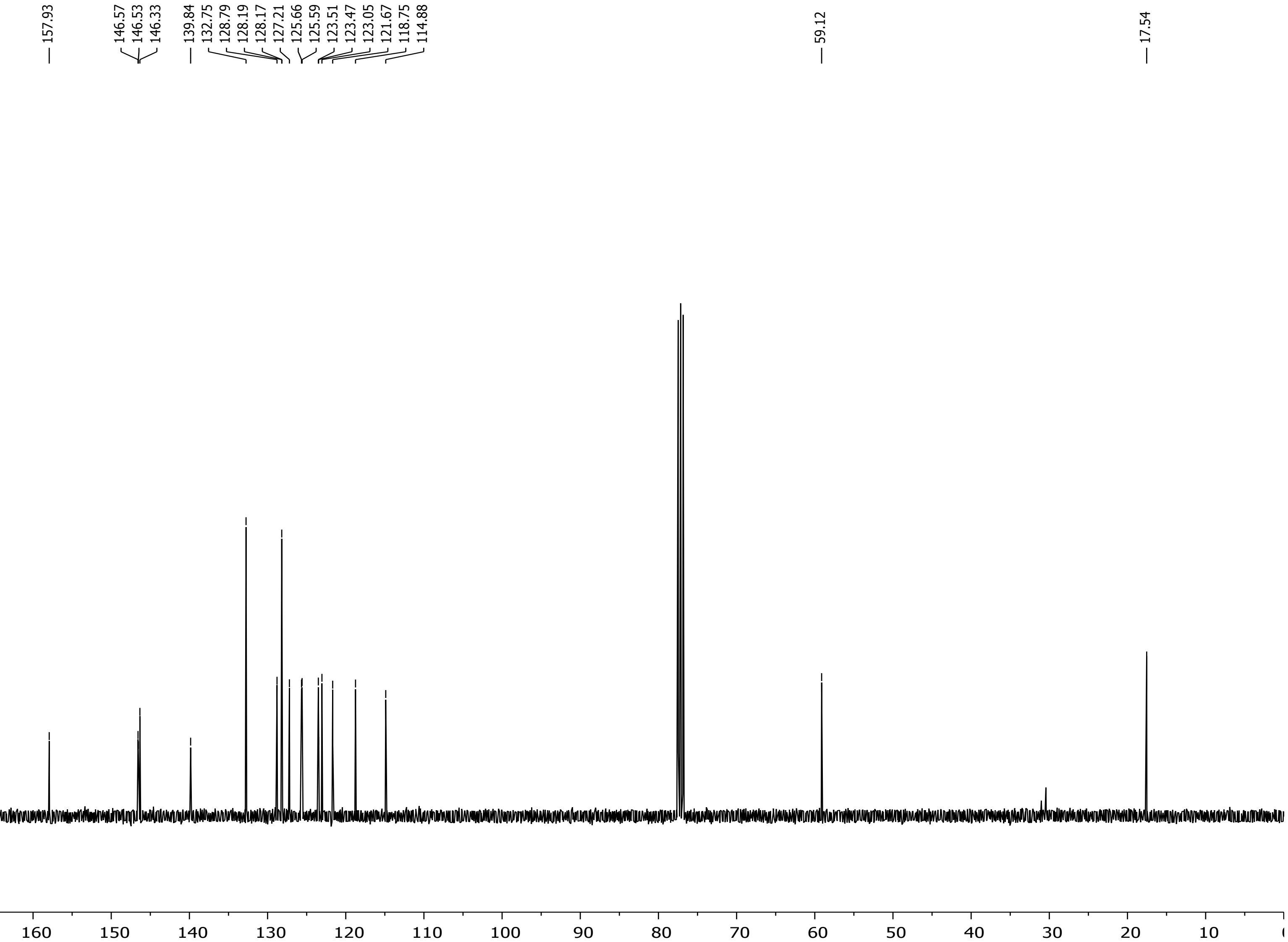

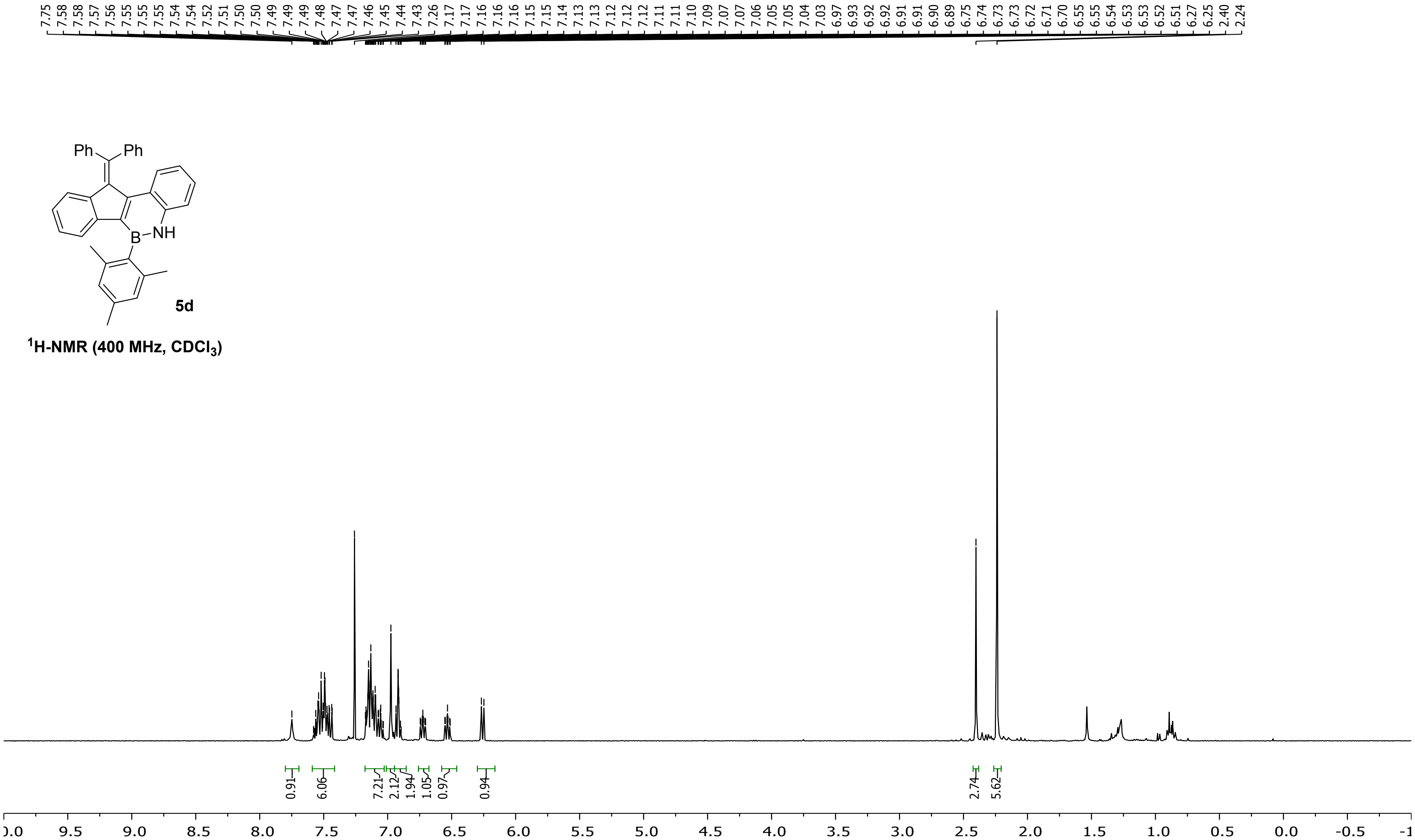

5d

^{1}H-NMR (400 MHz, $CDCl_3$)

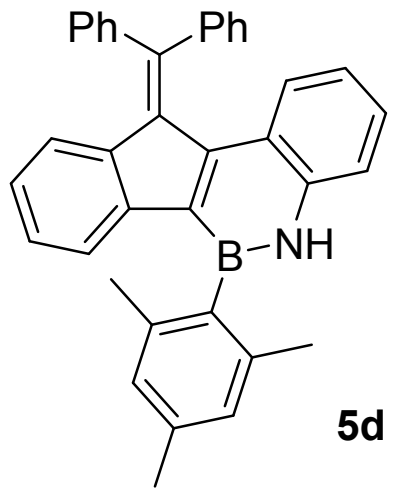

5d

^{11}B-NMR (128 MHz, $CDCl_3$)

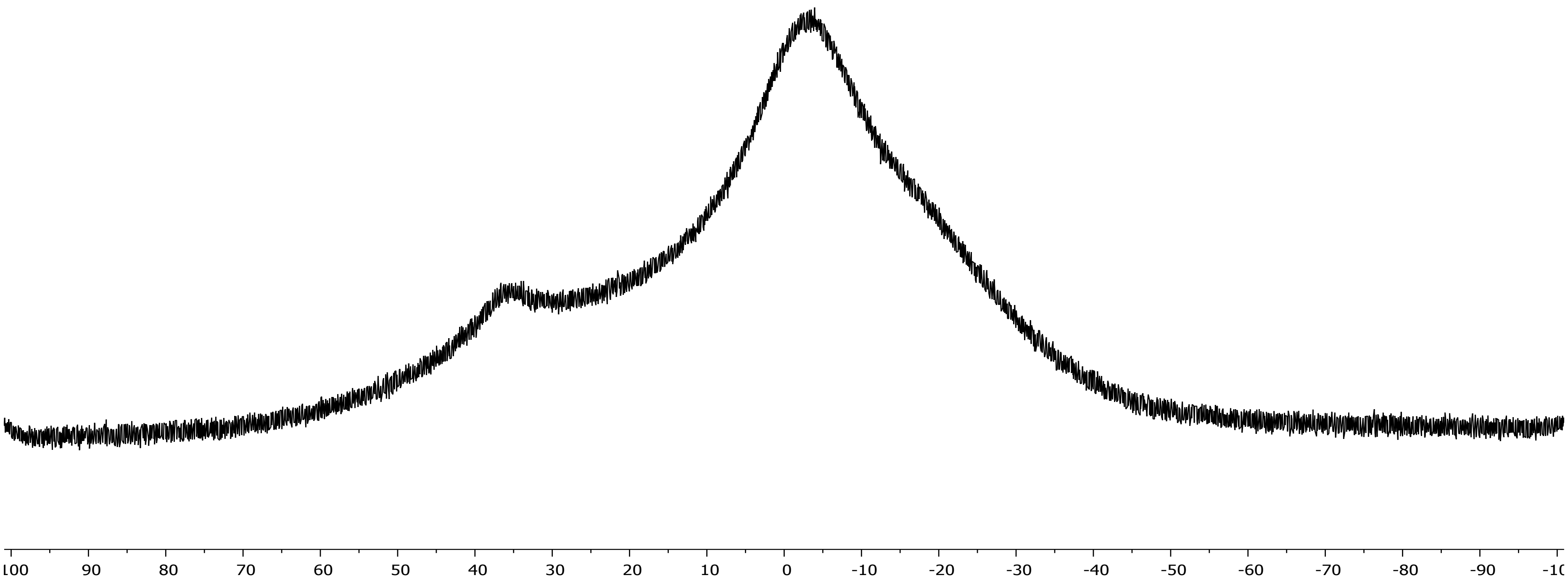

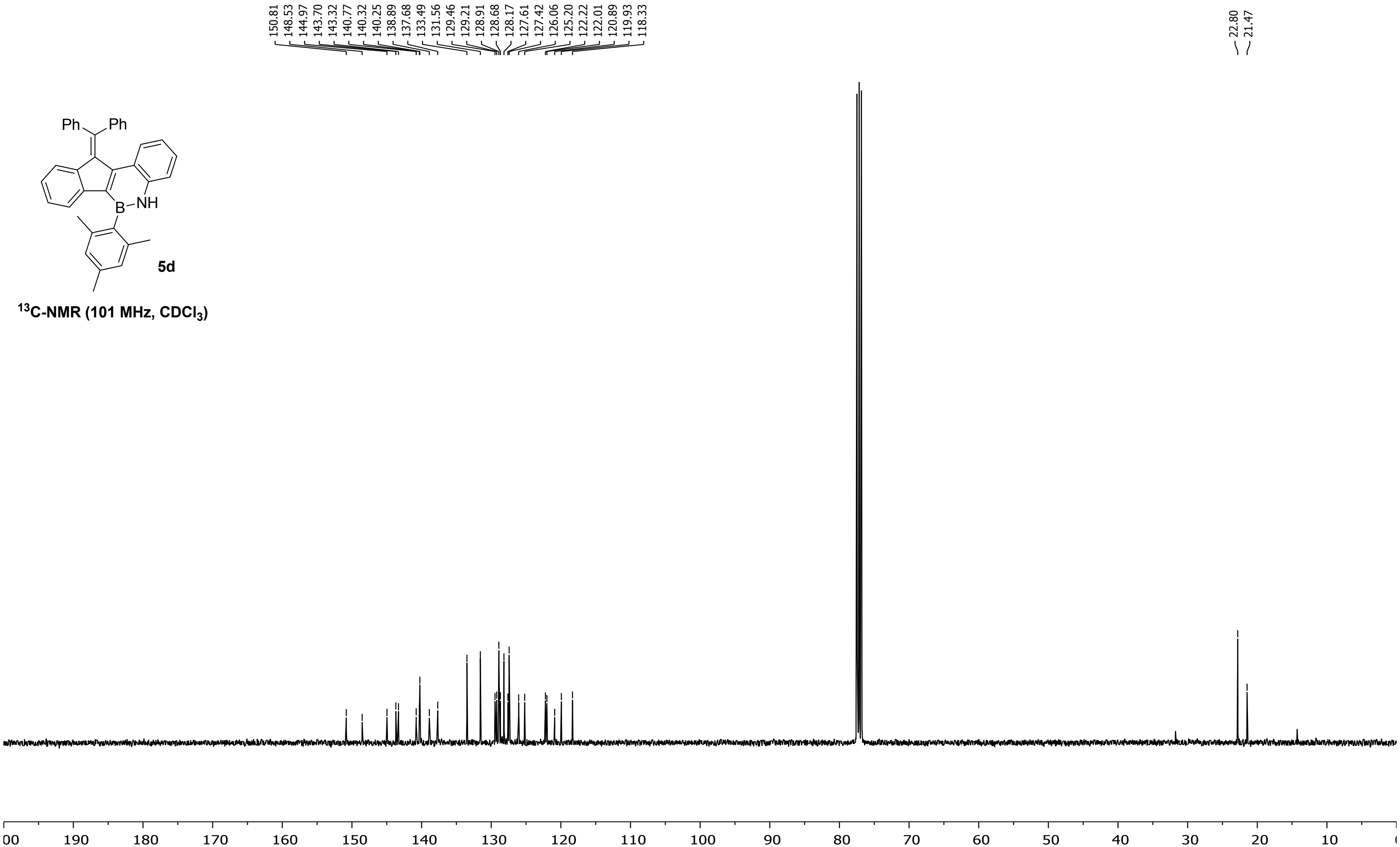

5d

^{13}C-NMR (101 MHz, $CDCl_3$)

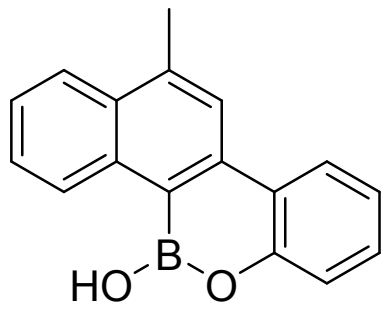

8a

^{1}H-NMR (400 MHz, Acetone-*d6*)

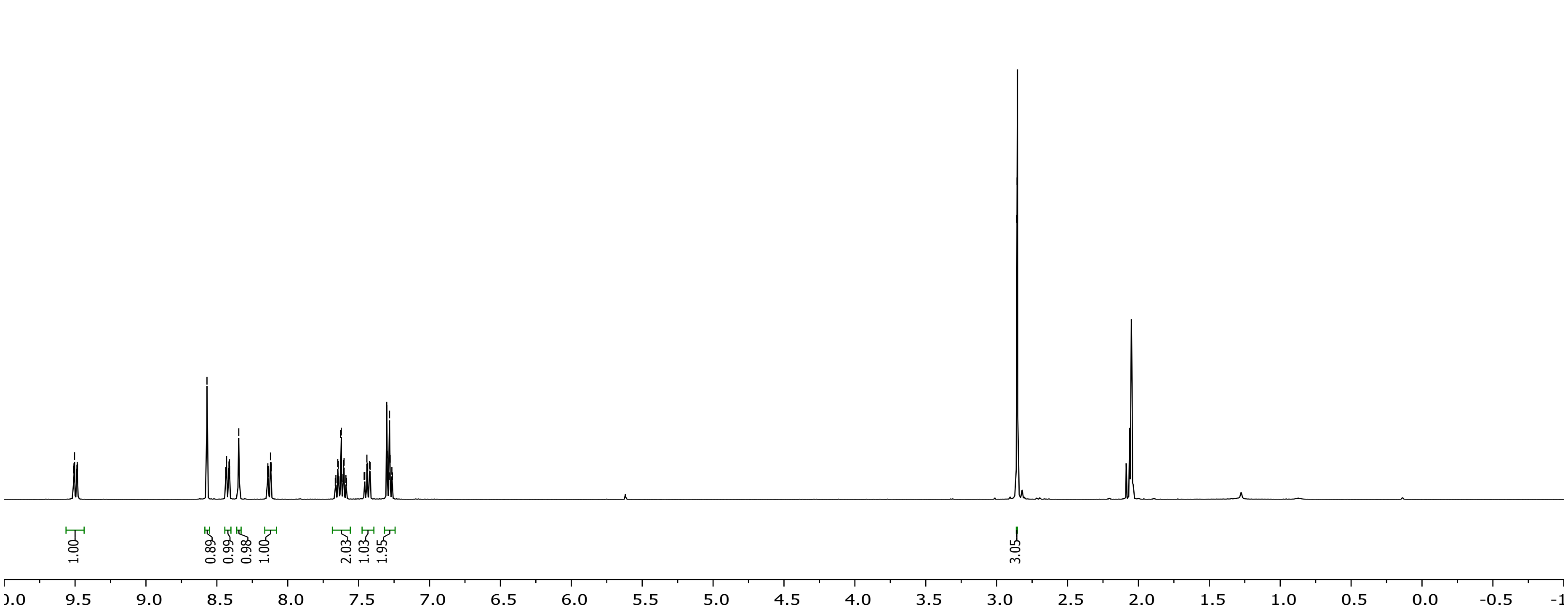

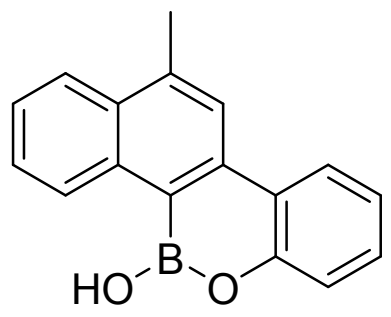

8a

^{11}B-NMR (128 MHz, Acetone-*d6*)

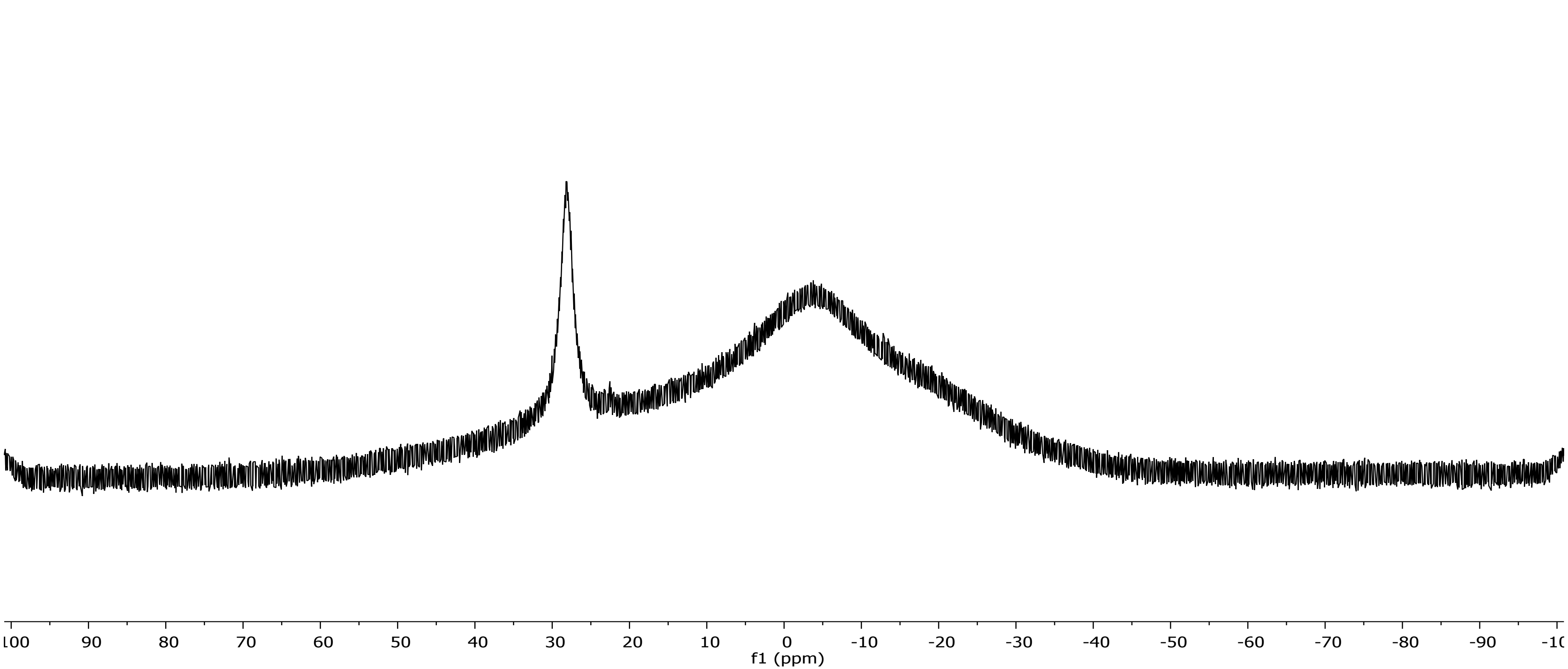

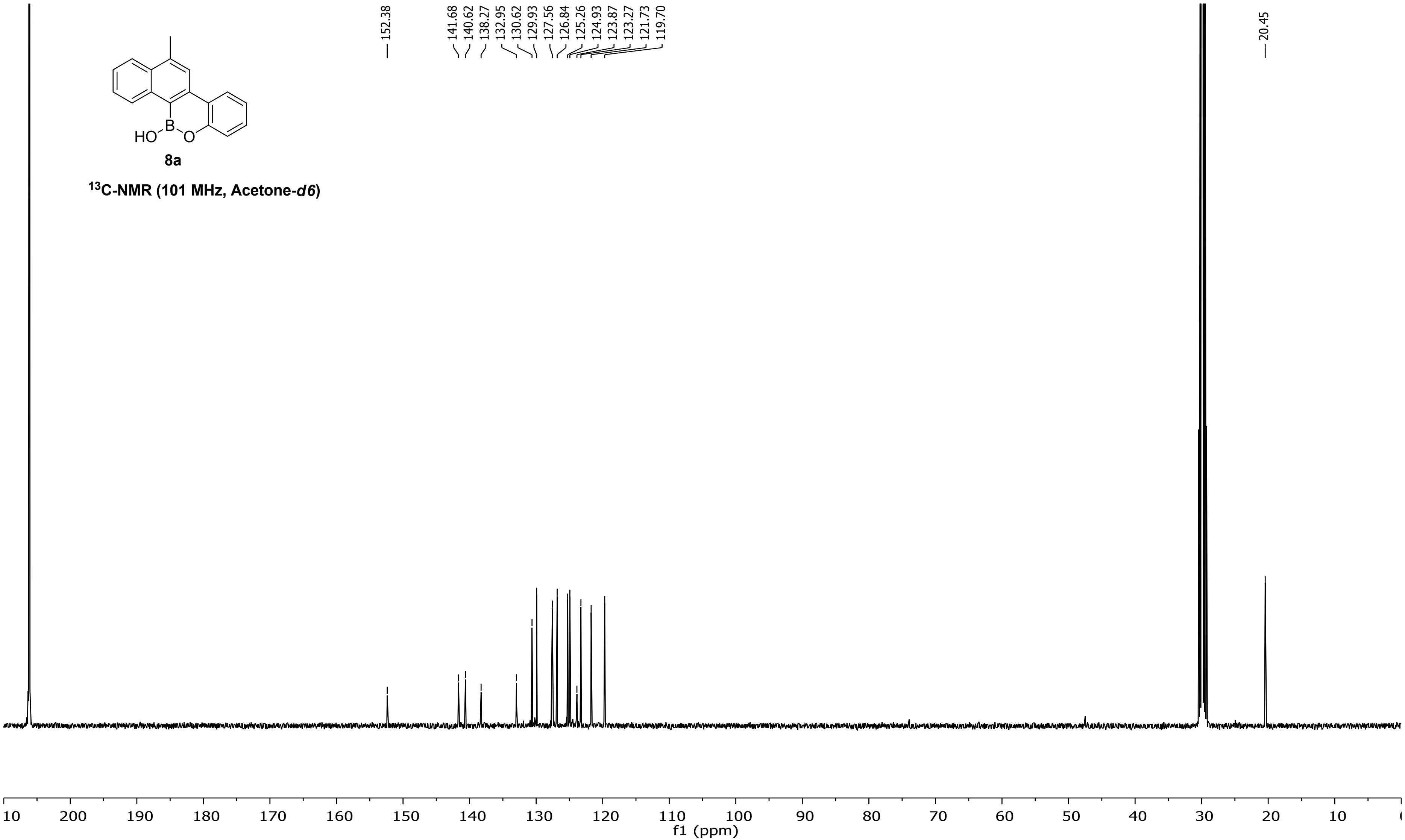

8a

^{13}C-NMR (101 MHz, Acetone-*d6*)

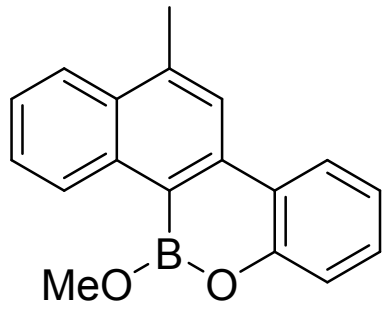

8b

^{1}H-NMR (400 MHz, CDCl$_3$)

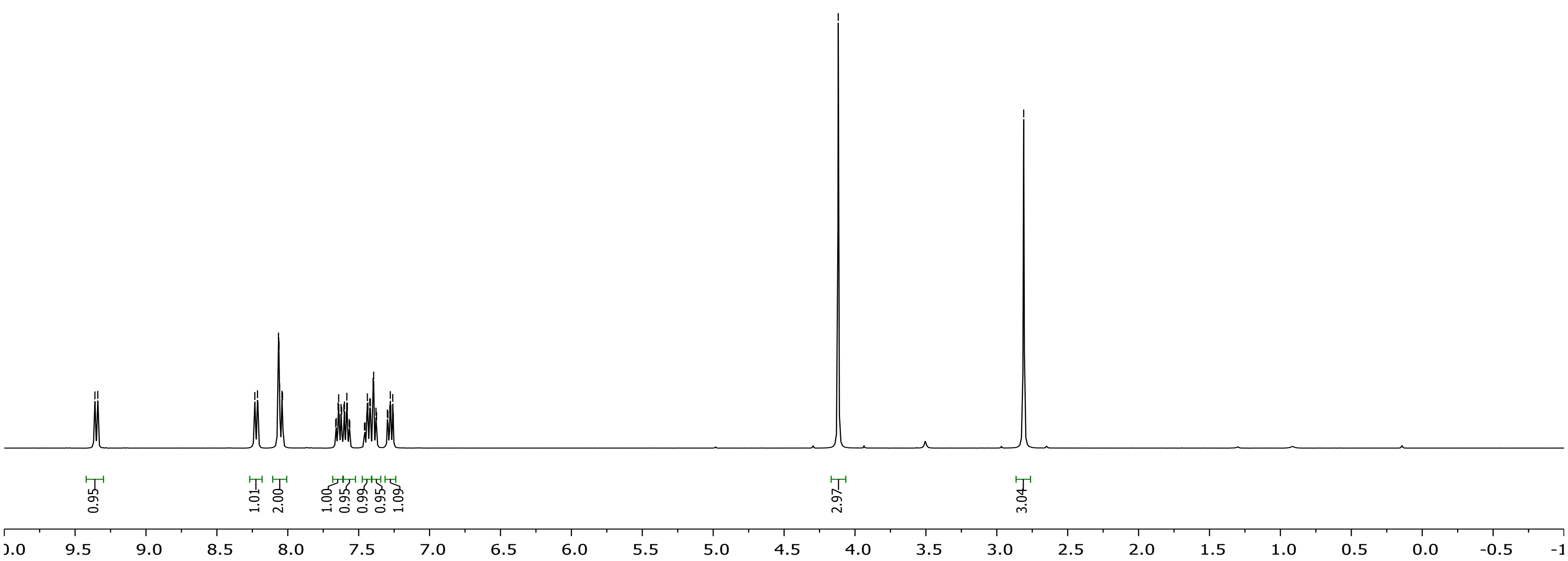

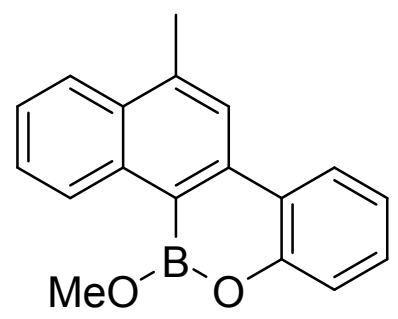

8b

^{11}B-NMR (128 MHz, $CDCl_3$)

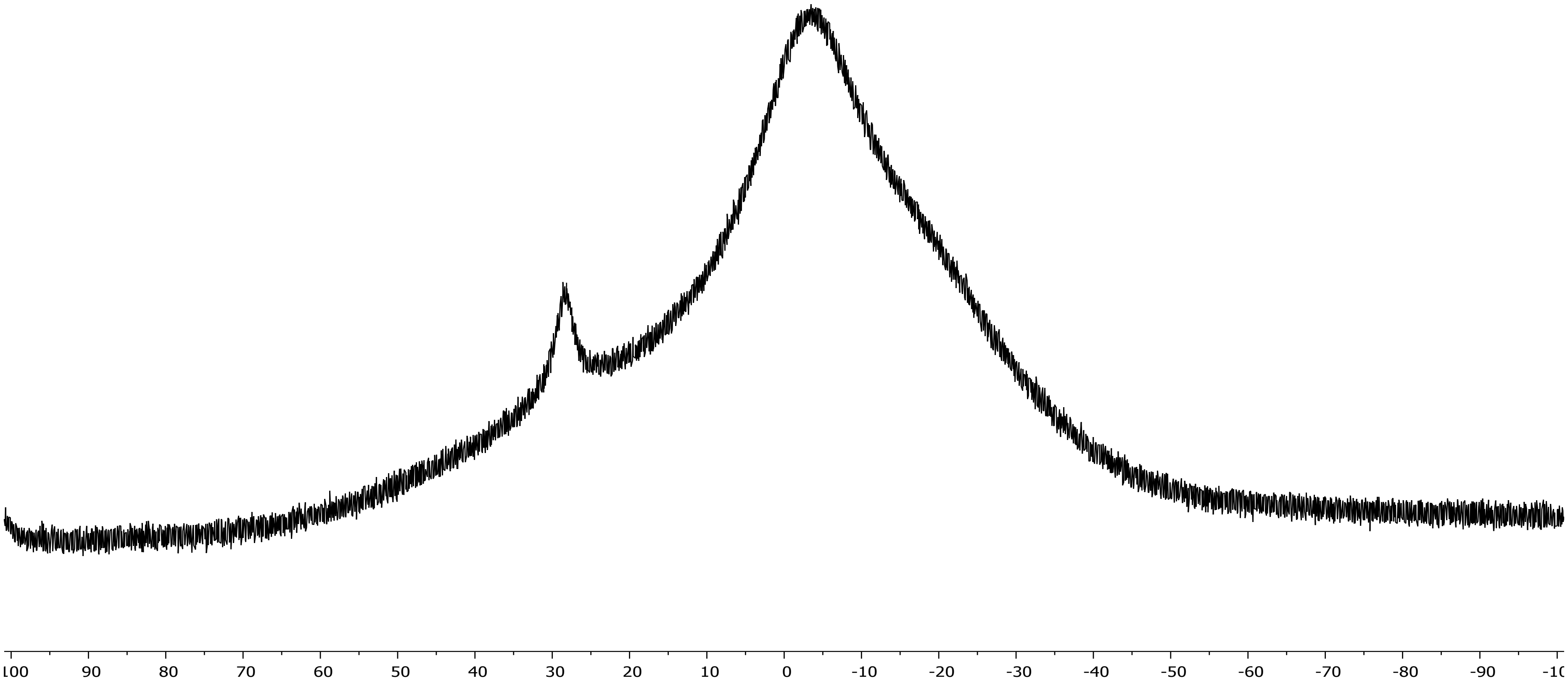

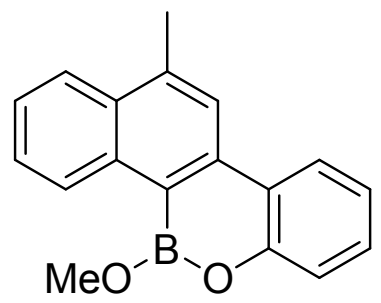

8b

^{13}C-NMR (101 MHz, $CDCl_3$)

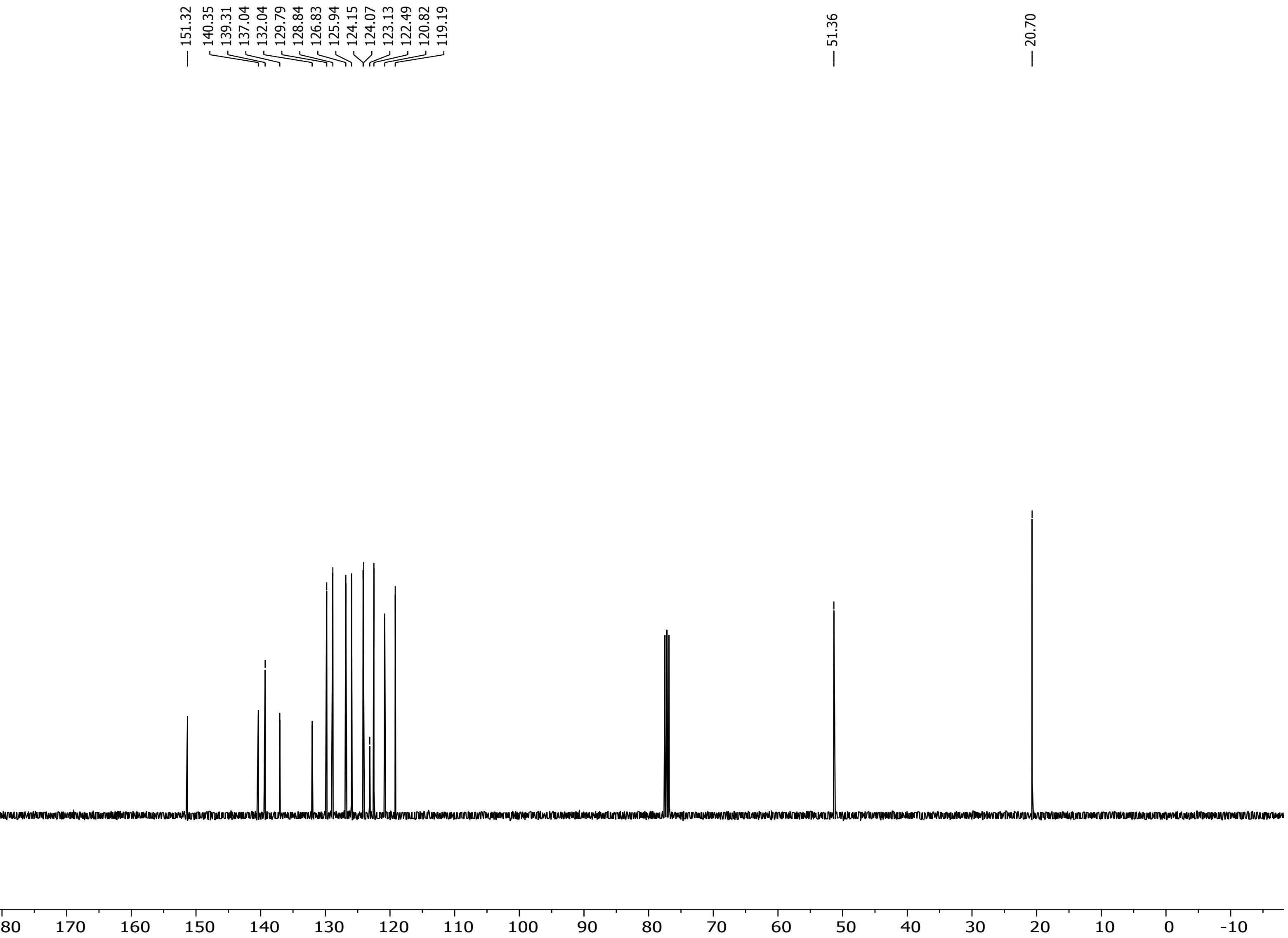

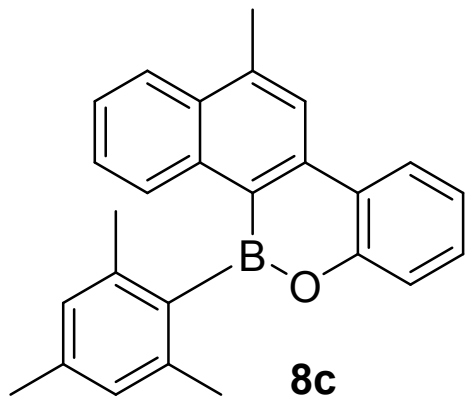

8c

^{1}H-NMR (400 MHz, $CDCl_3$)

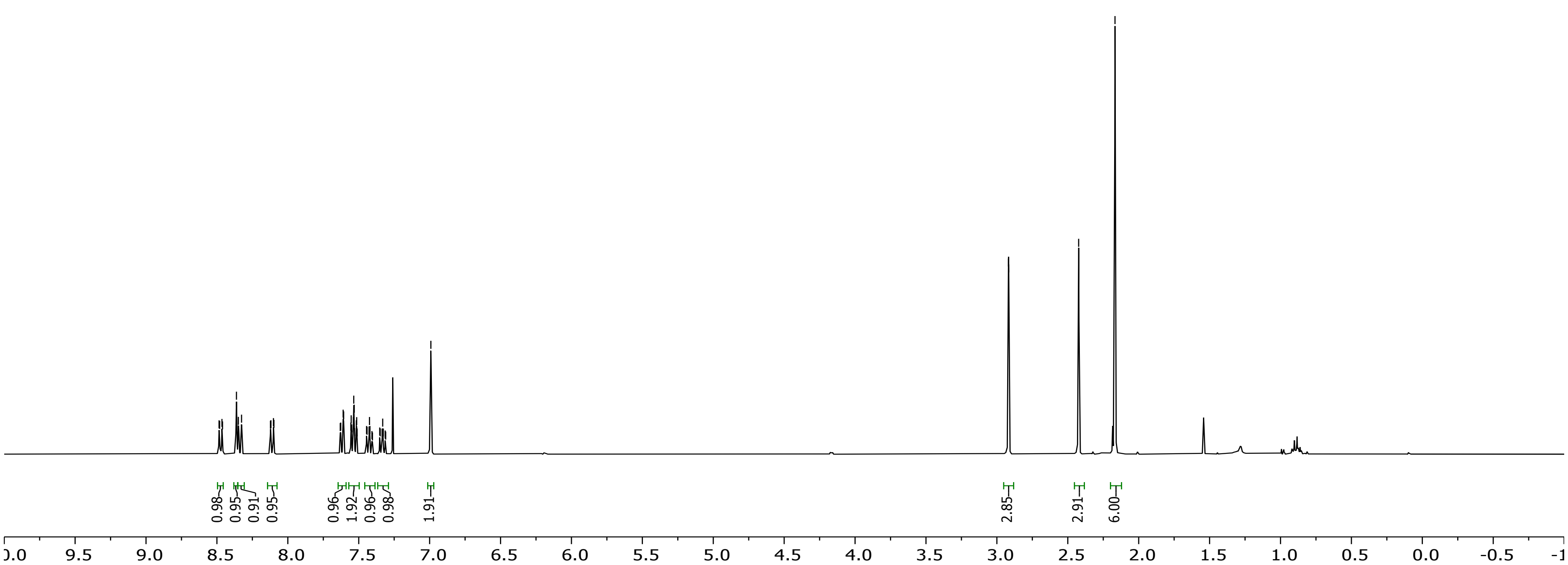

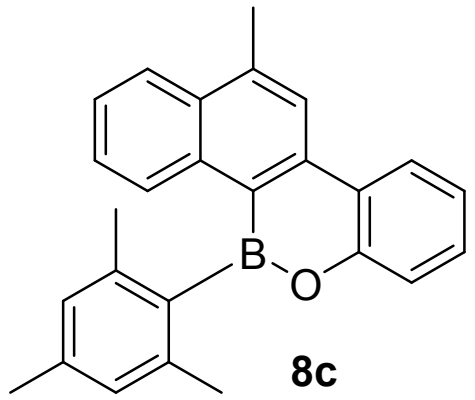

8c

^{11}B-NMR (128 MHz, $CDCl_3$)

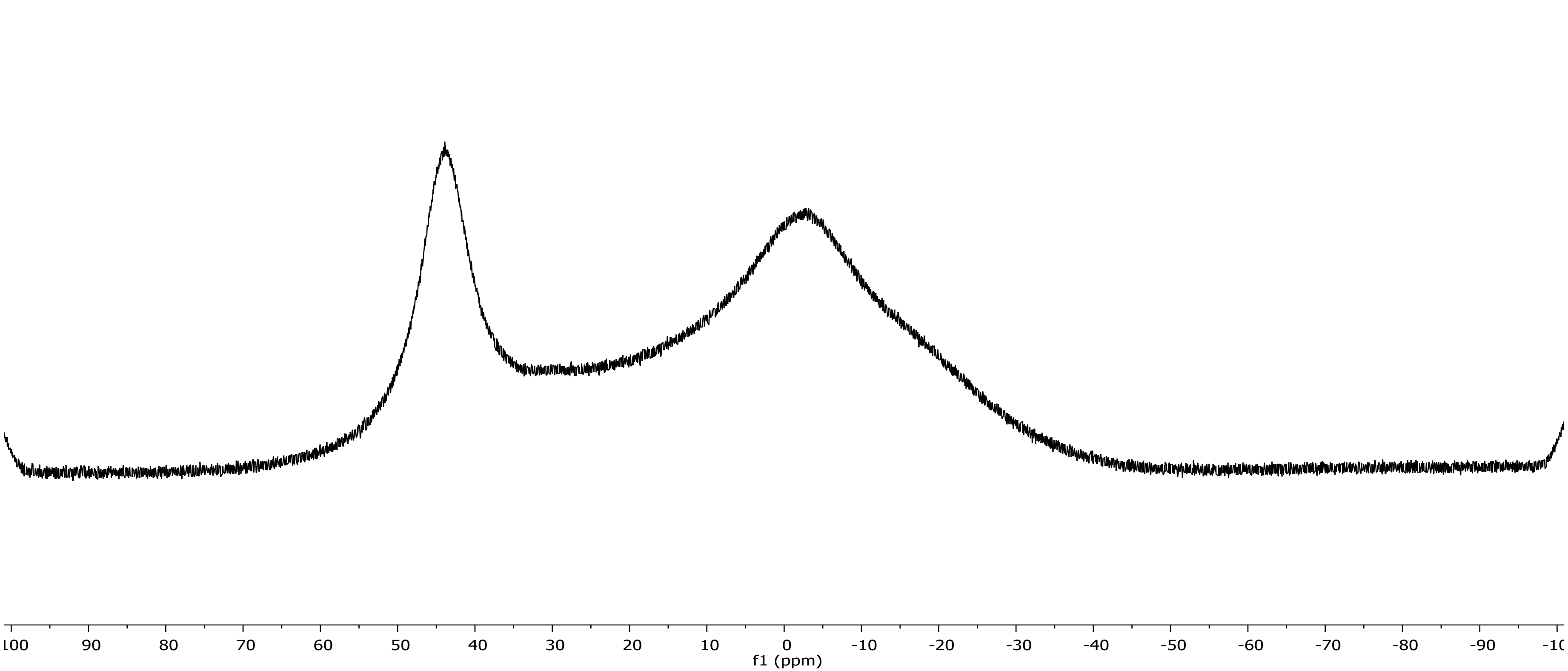

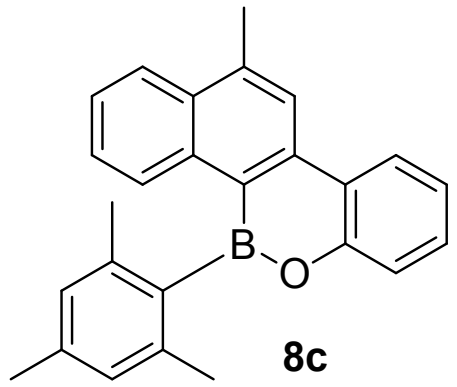

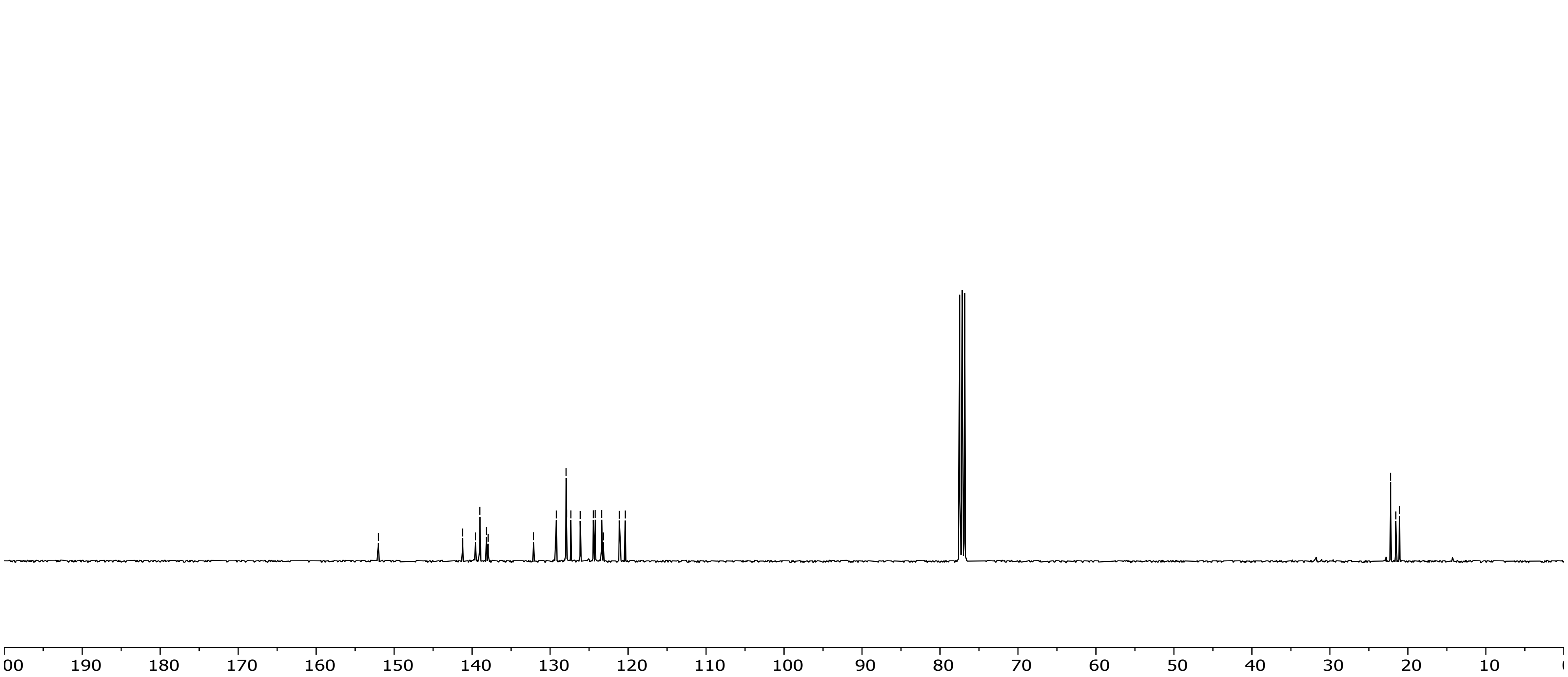

8c

^{11}B-NMR (101 MHz, CDCl$_3$)

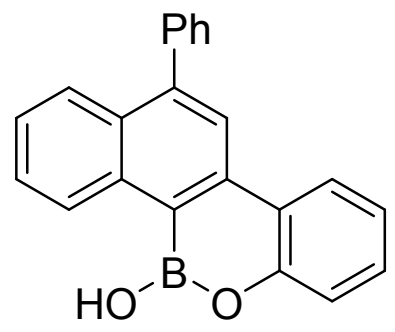

8d

^{1}H-NMR (400 MHz, $CDCl_3$)

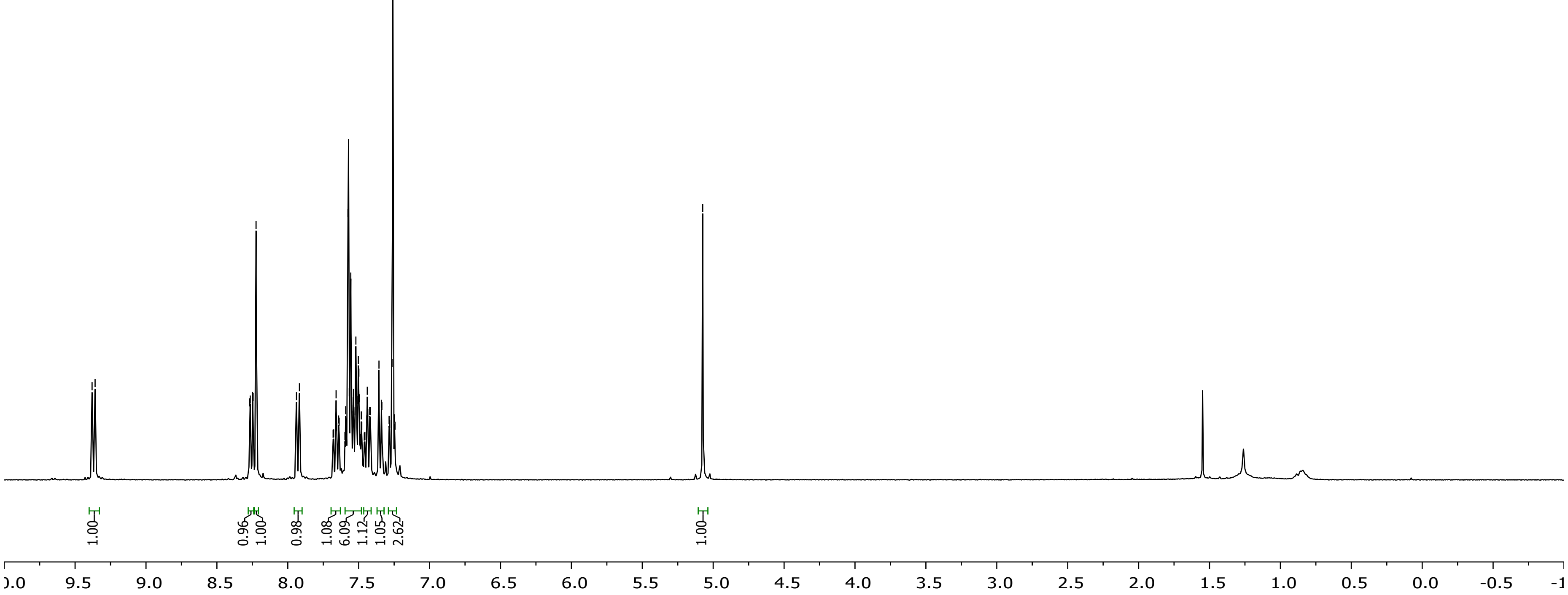

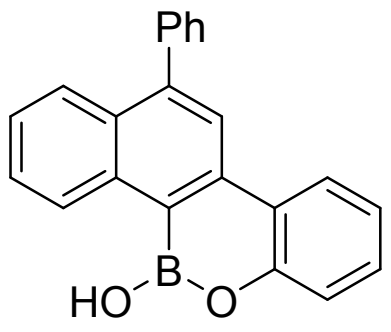

8d

^{11}B-NMR (128 MHz, $CDCl_3$)

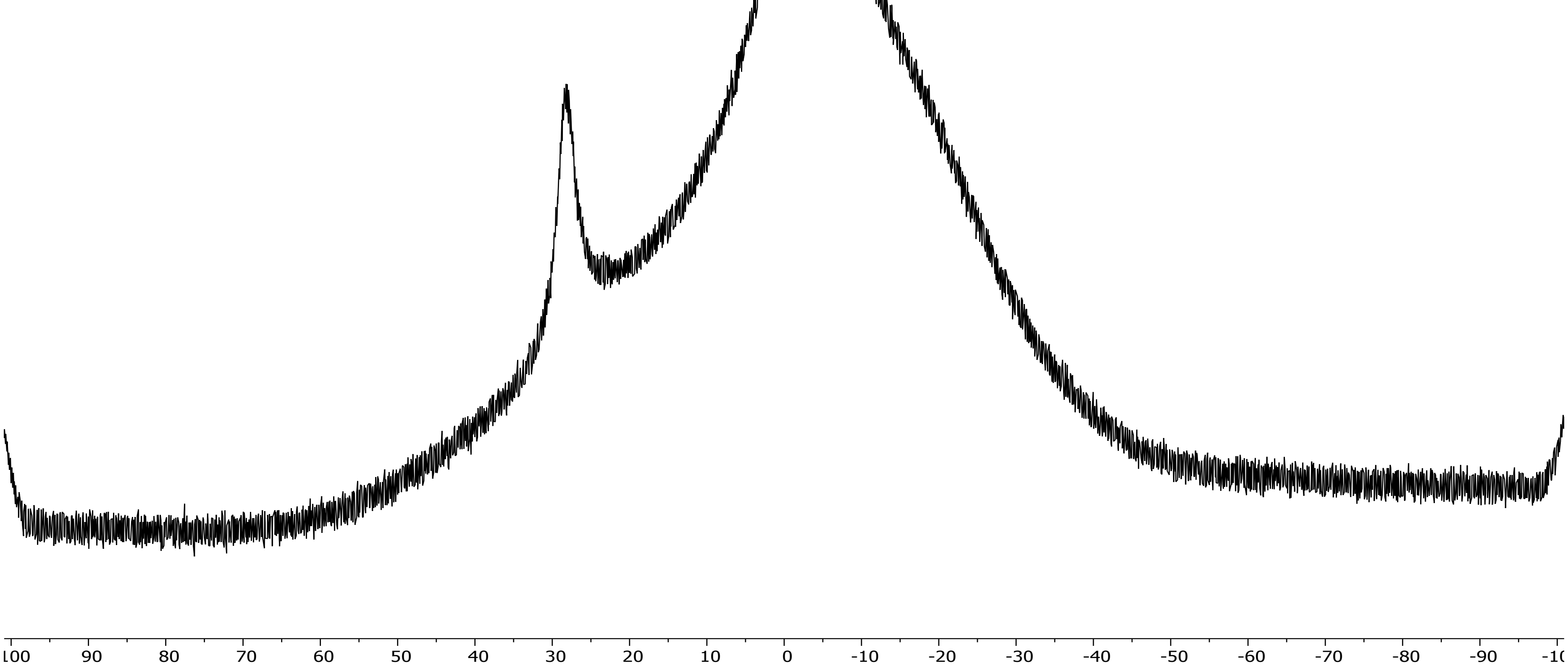

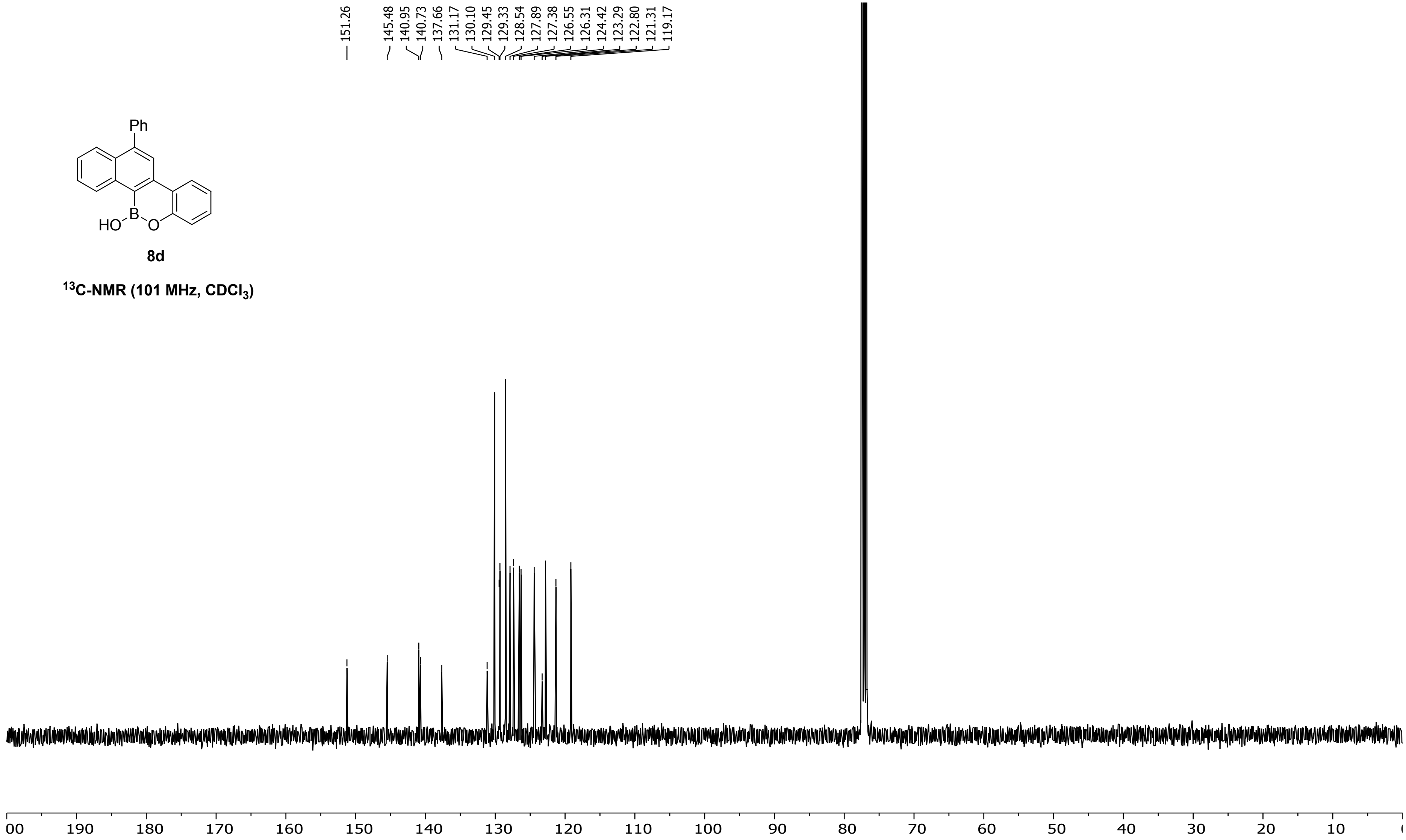

8d

^{13}C-NMR (101 MHz, $CDCl_3$)

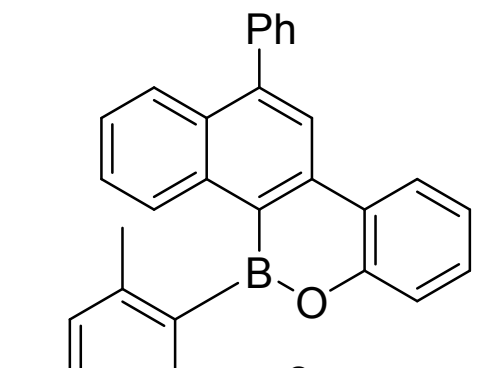

8e

^{1}H-NMR (400 MHz, CDCl$_3$)

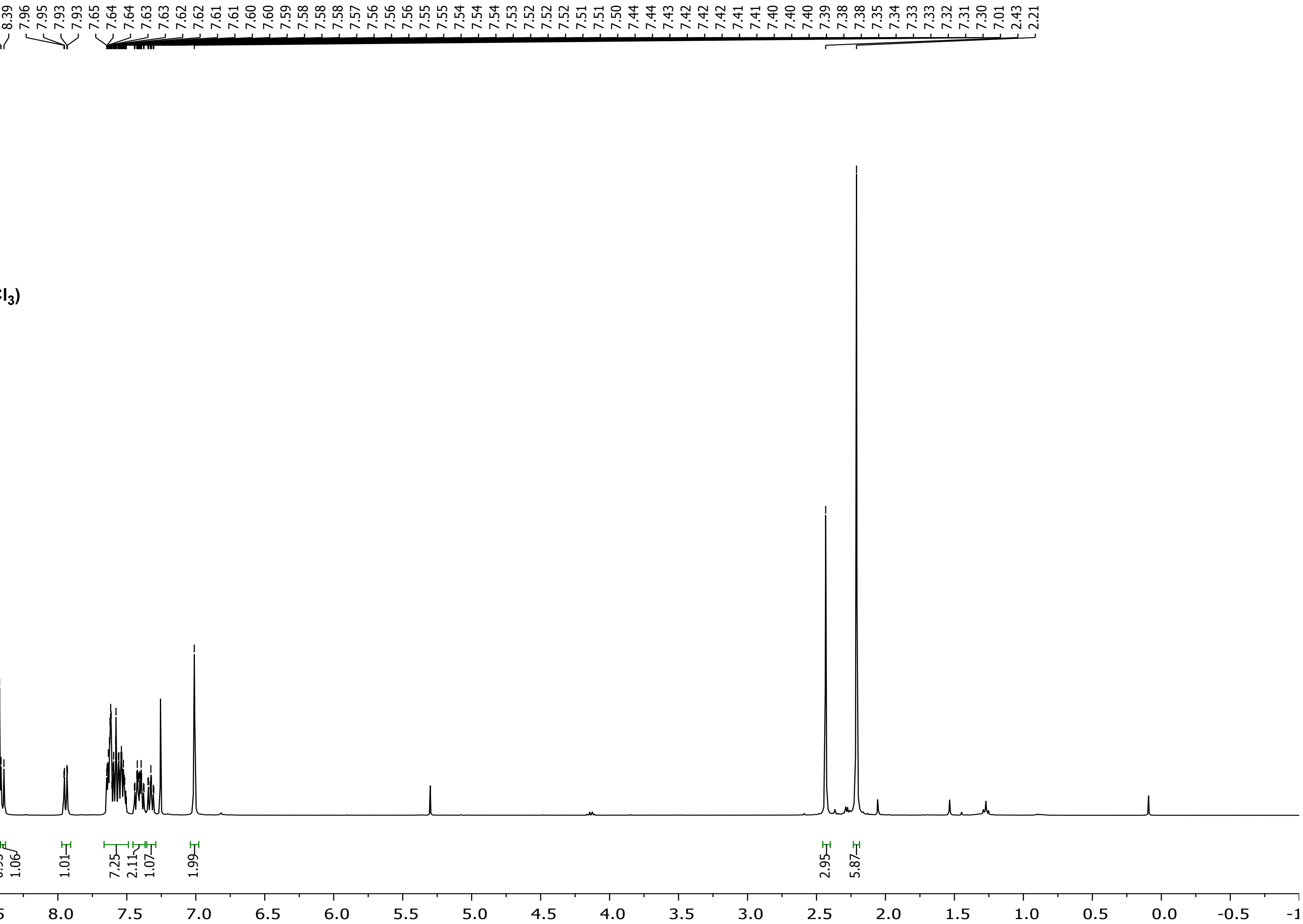

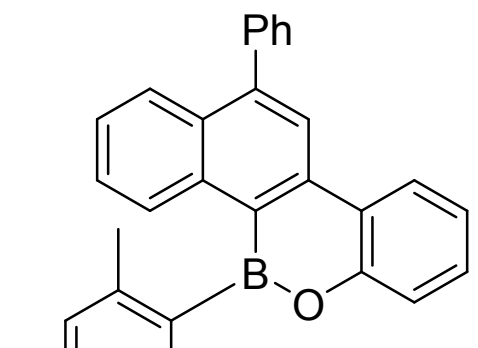

8e

^{11}B-NMR (128 MHz, CDCl$_3$)

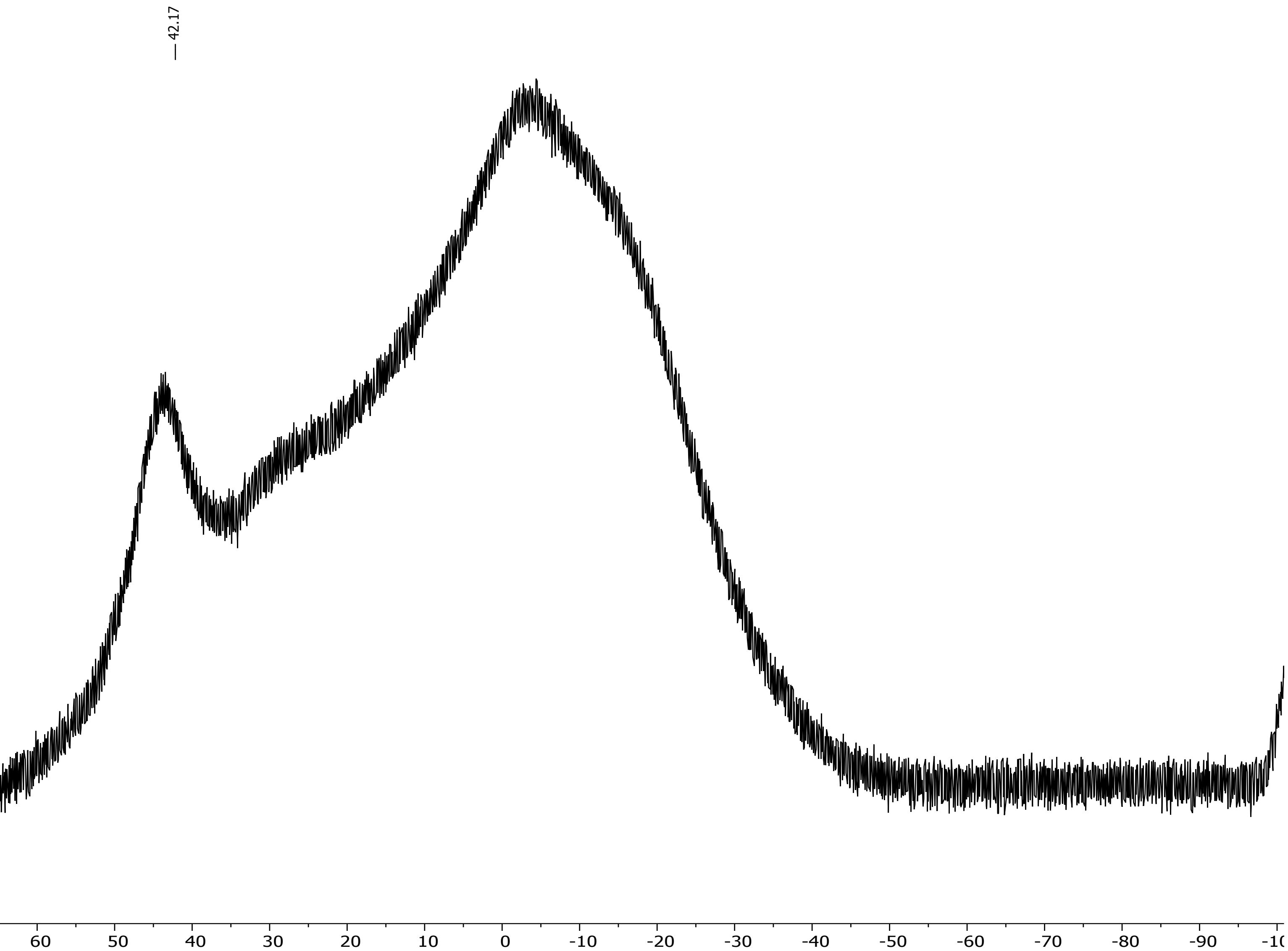

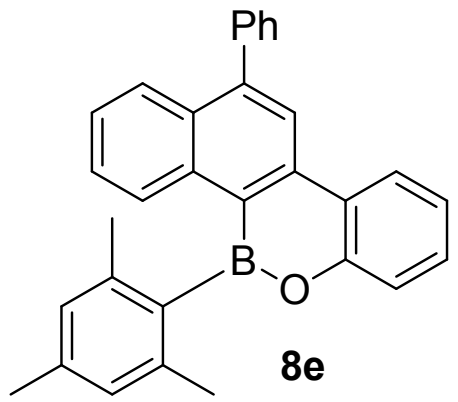

8e

^{13}C-NMR (101MHz, $CDCl_3$)

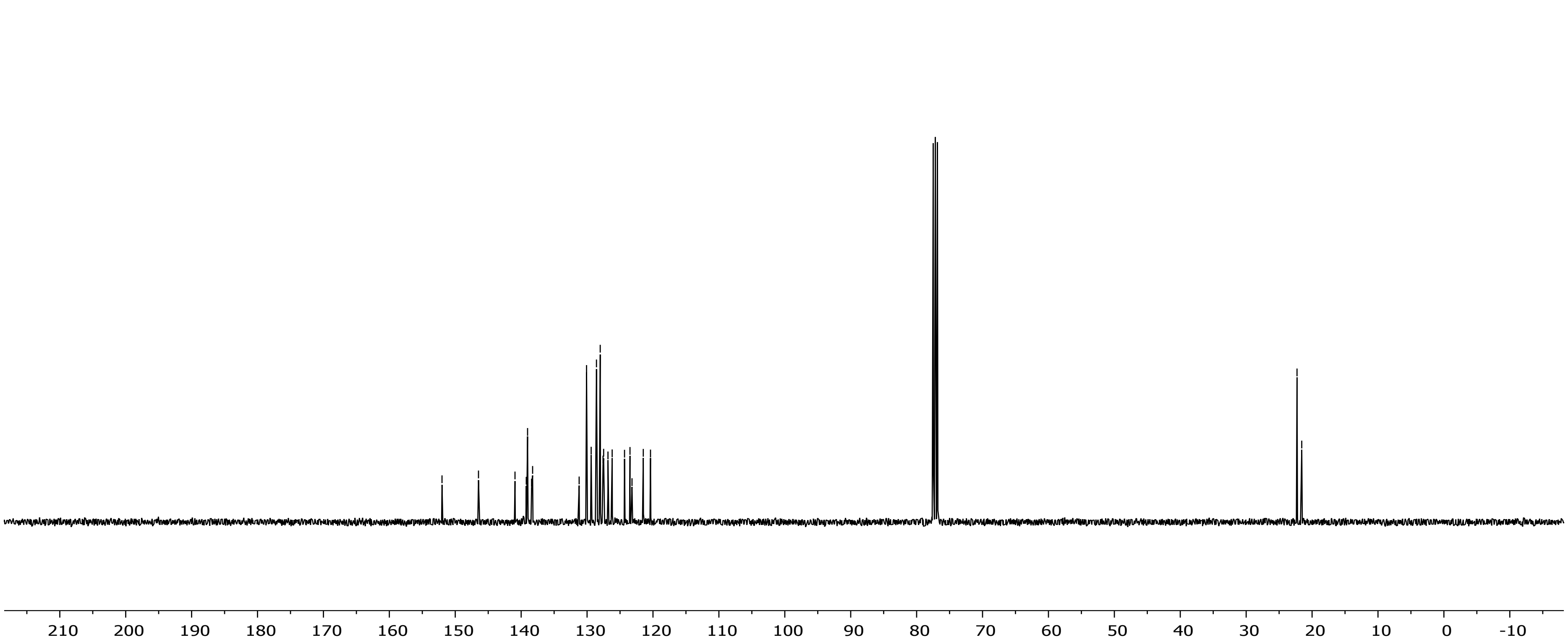

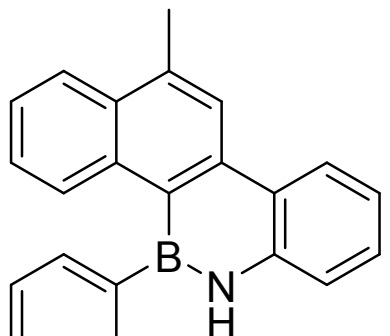

9a

^{1}H-NMR (400 MHz, $CDCl_3$)

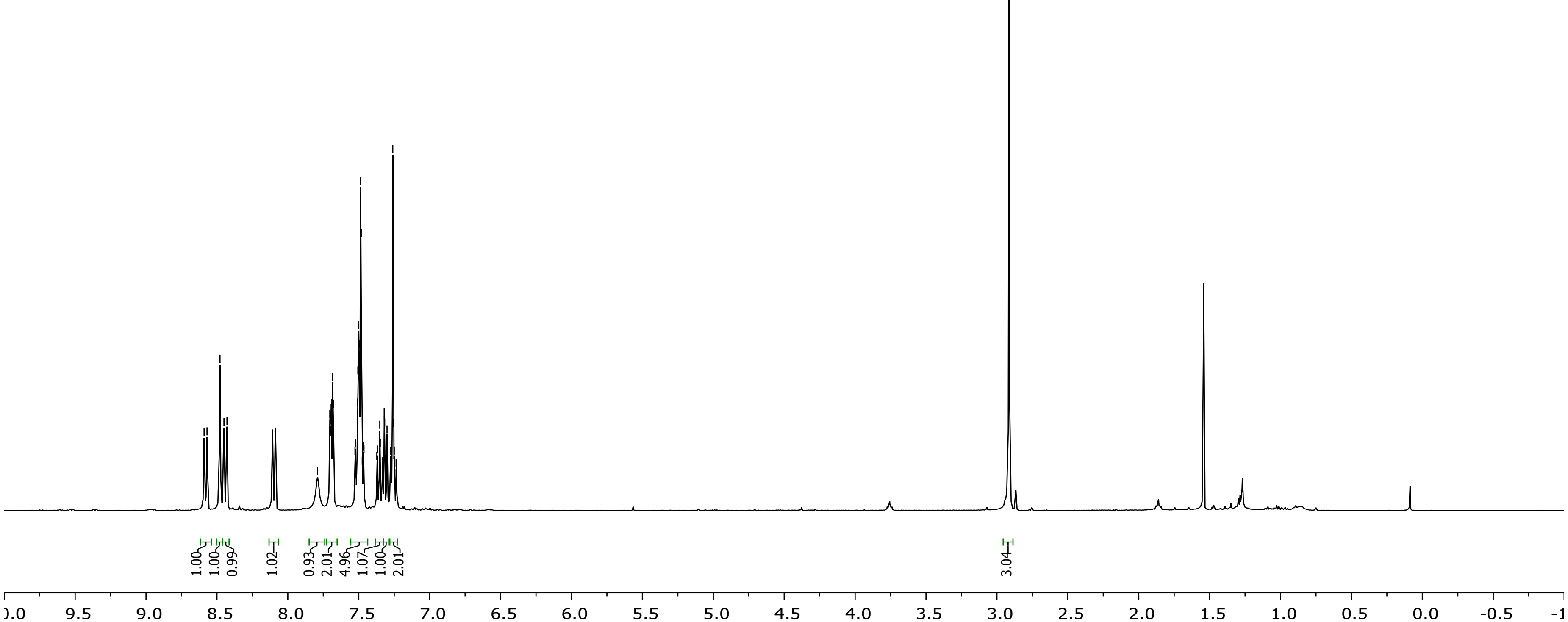

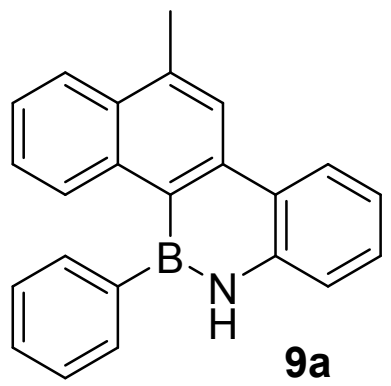

9a

^{11}B-NMR (128 MHz, $CDCl_3$)

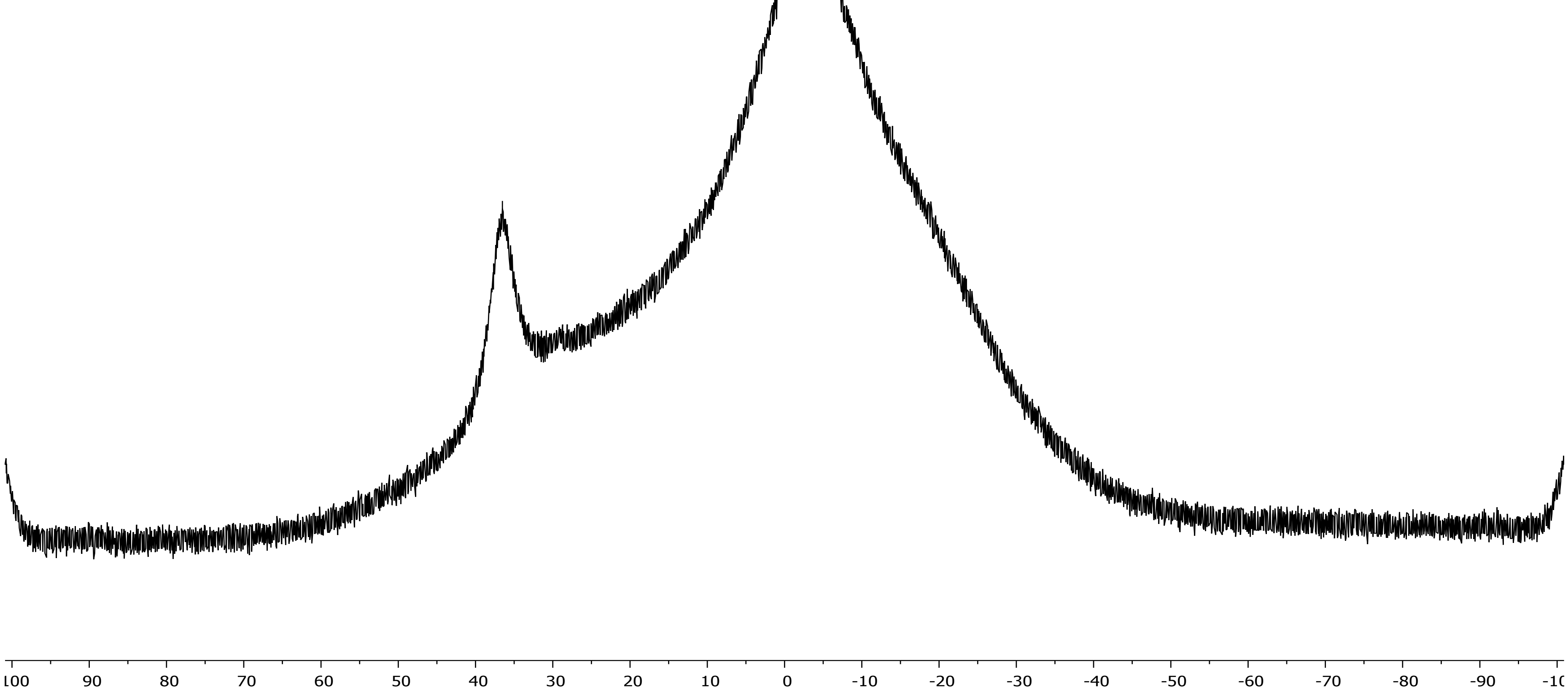

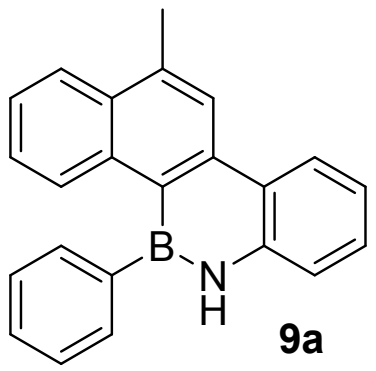

9a

^{13}C-NMR (101 MHz, $CDCl_3$)

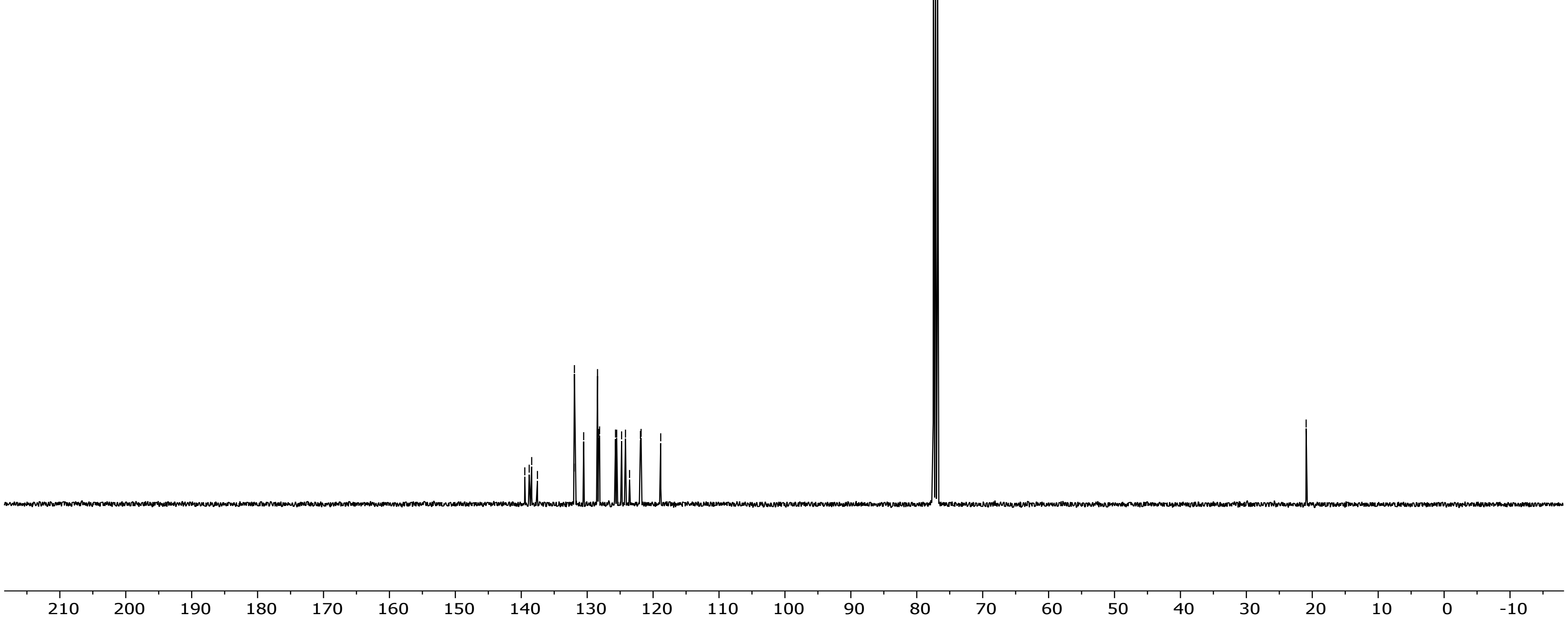

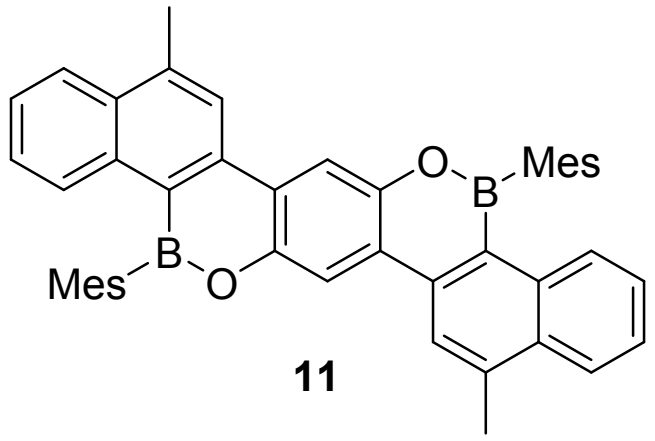

11

^{1}H-NMR (400 MHz, CDCl$_3$)

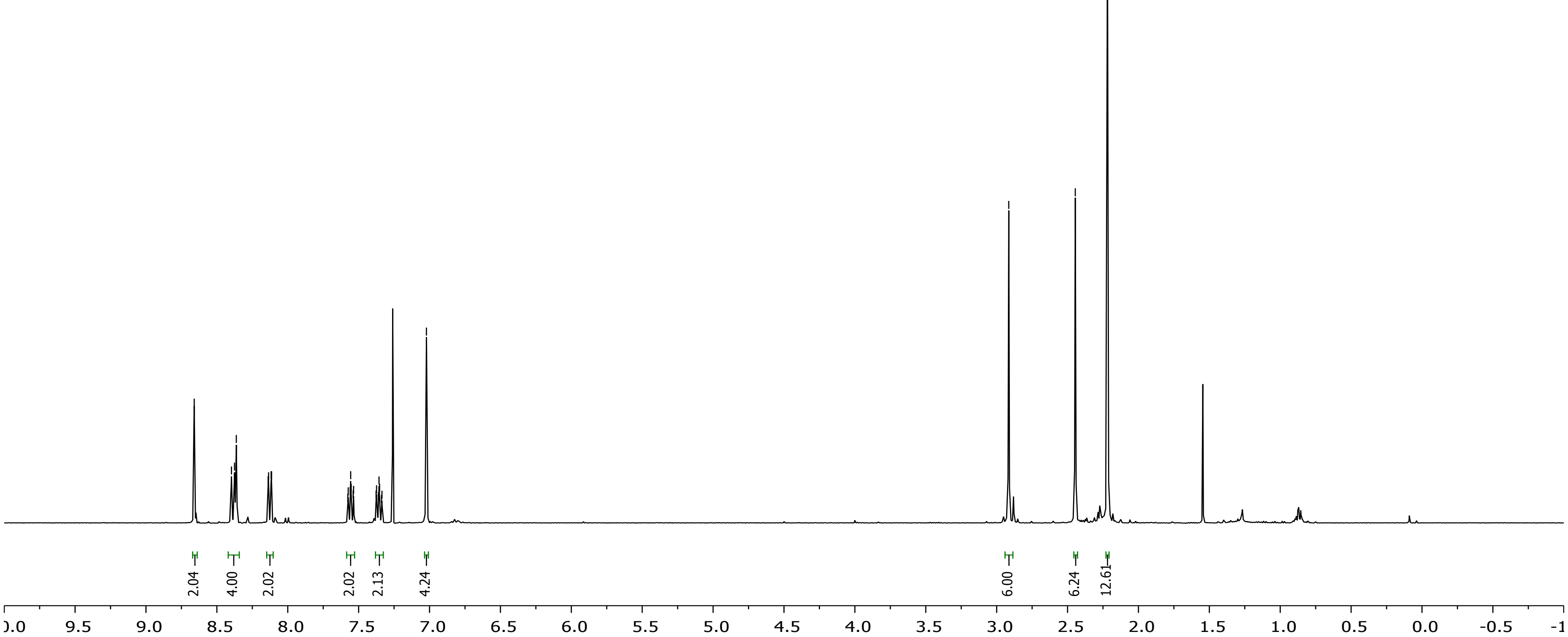

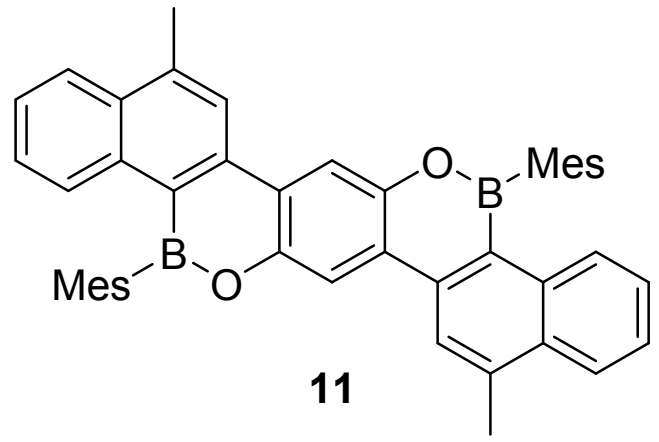

11

^{11}B-NMR (128 MHz, $CDCl_3$)

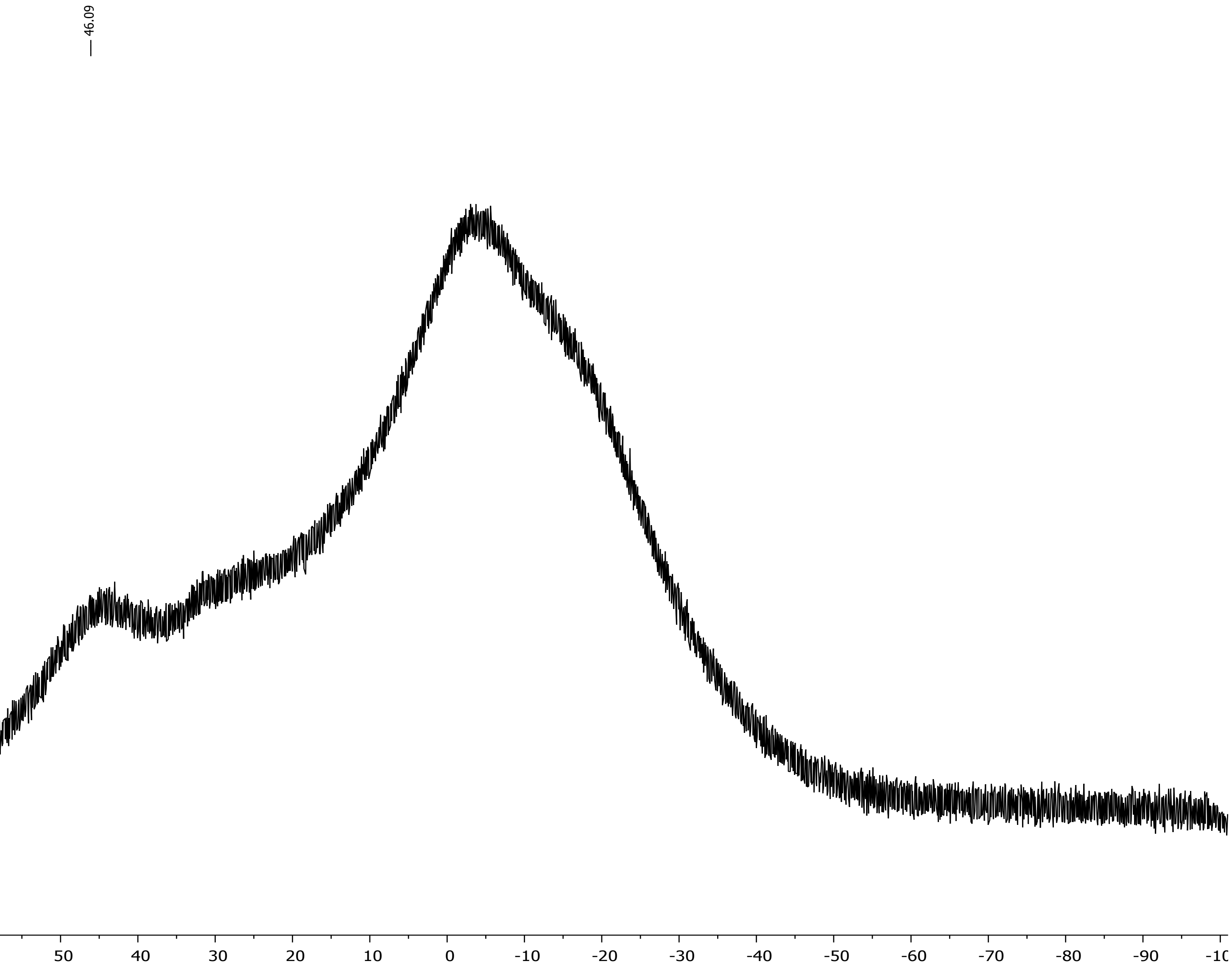

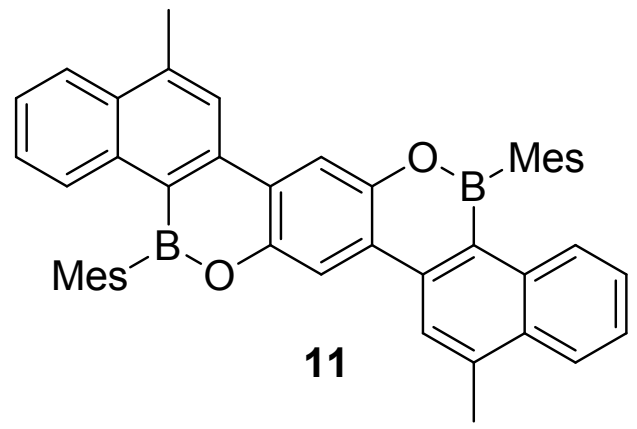

11

^{13}C-NMR (101 MHz, $CDCl_3$)

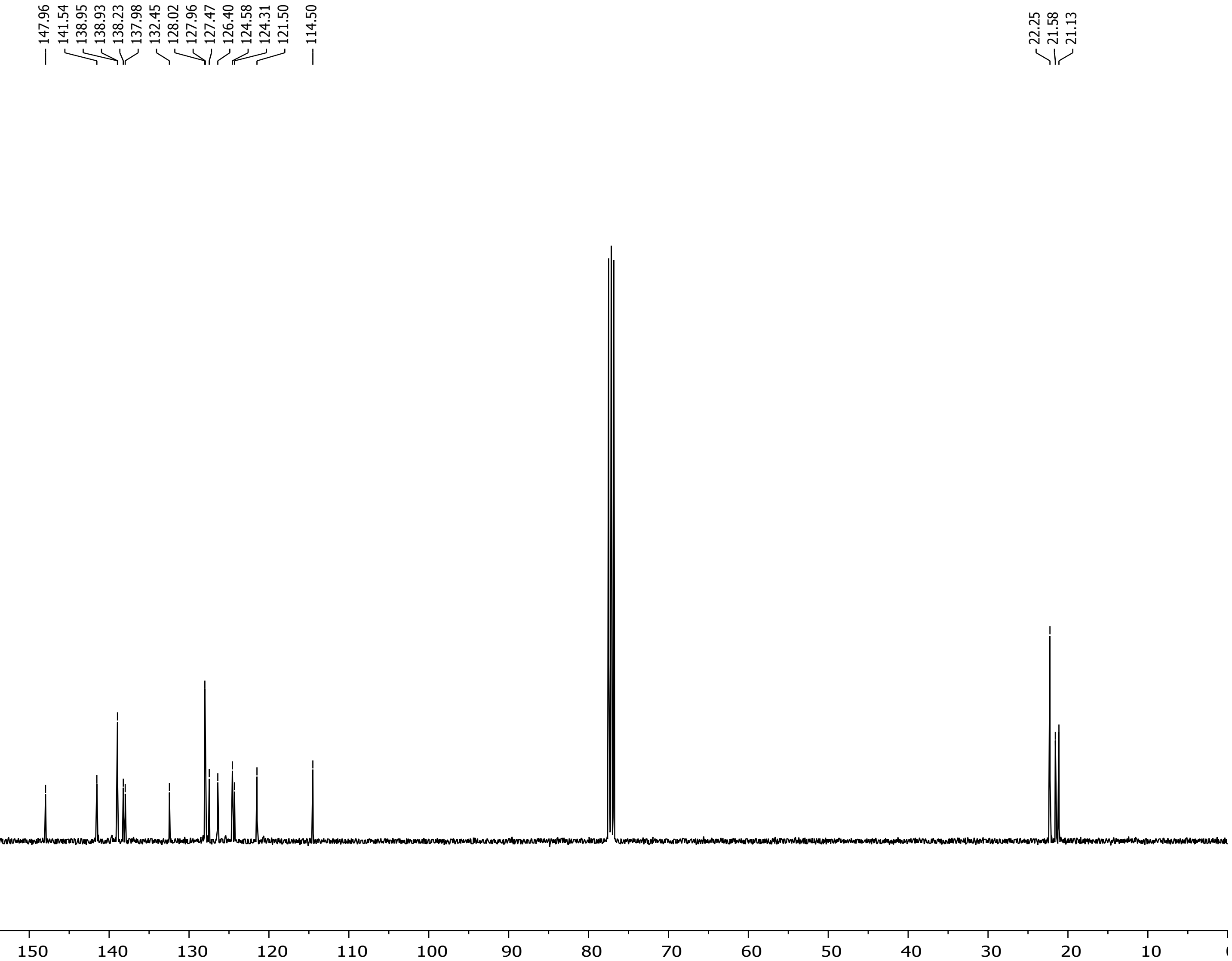

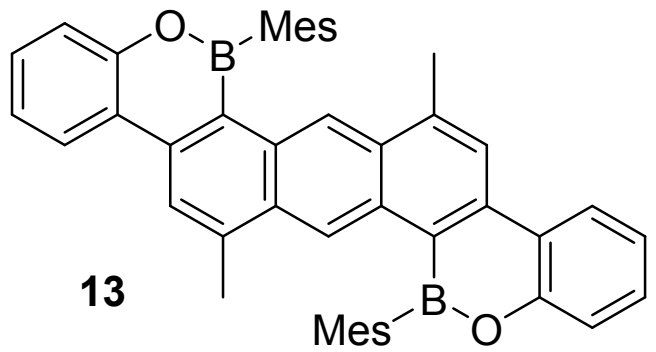

13

^{1}H-NMR (400 MHz, $CDCl_3$)

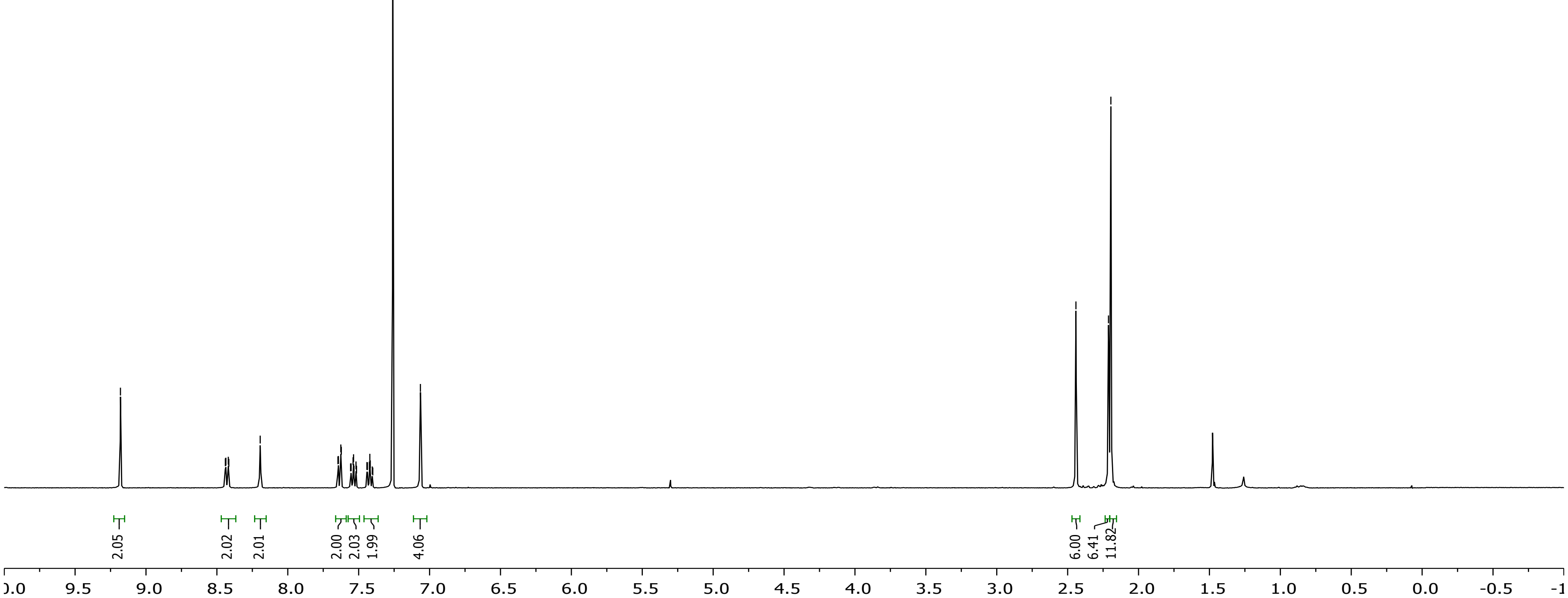

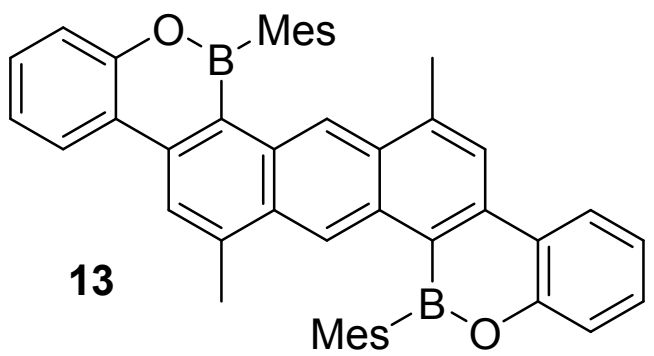

13

^{11}**B-NMR (128 MHz, $CDCl_3$)**

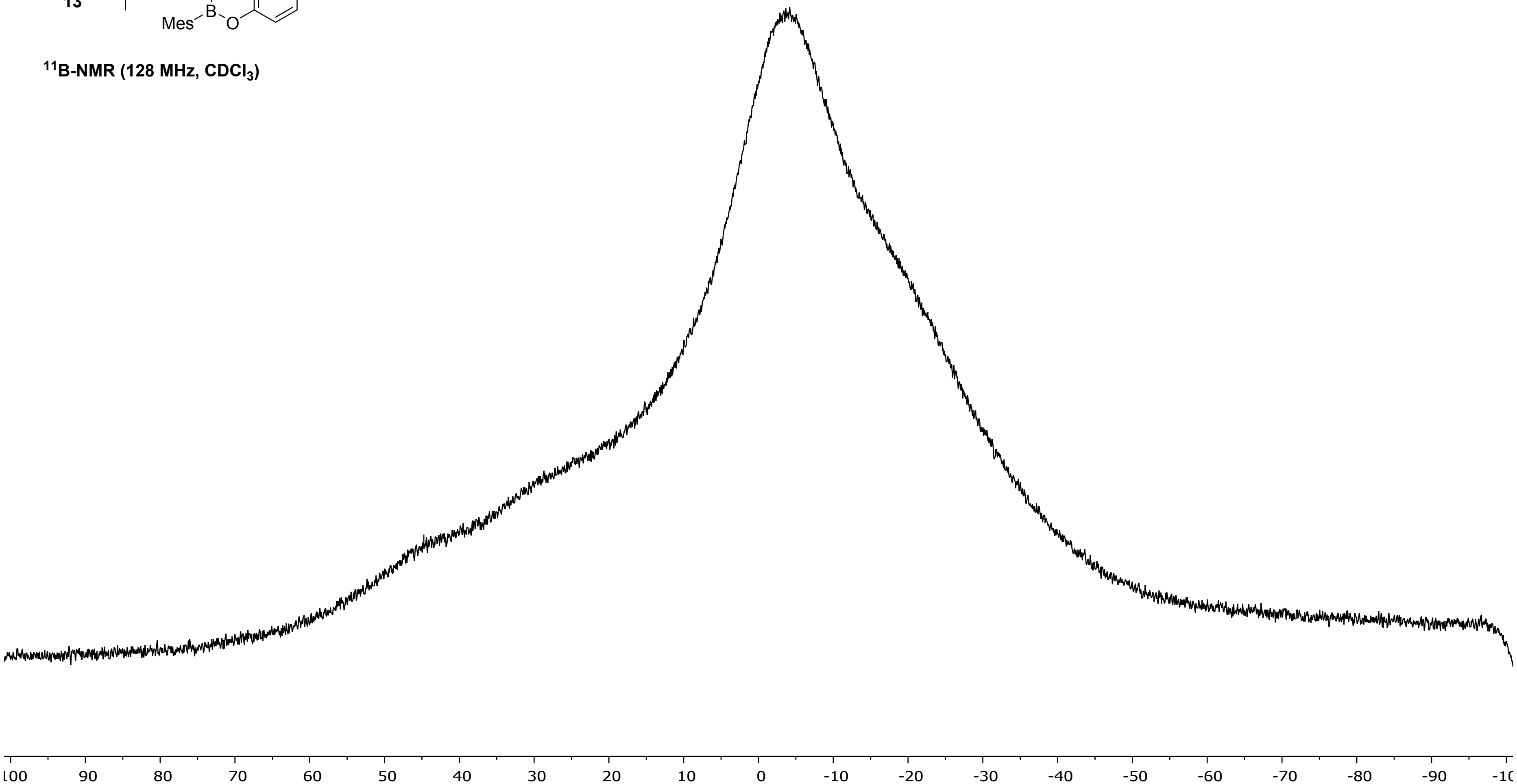

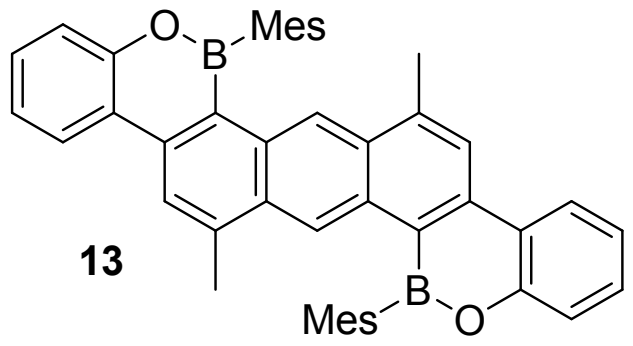

13

^{13}C-NMR (101 MHz, $CDCl_3$)

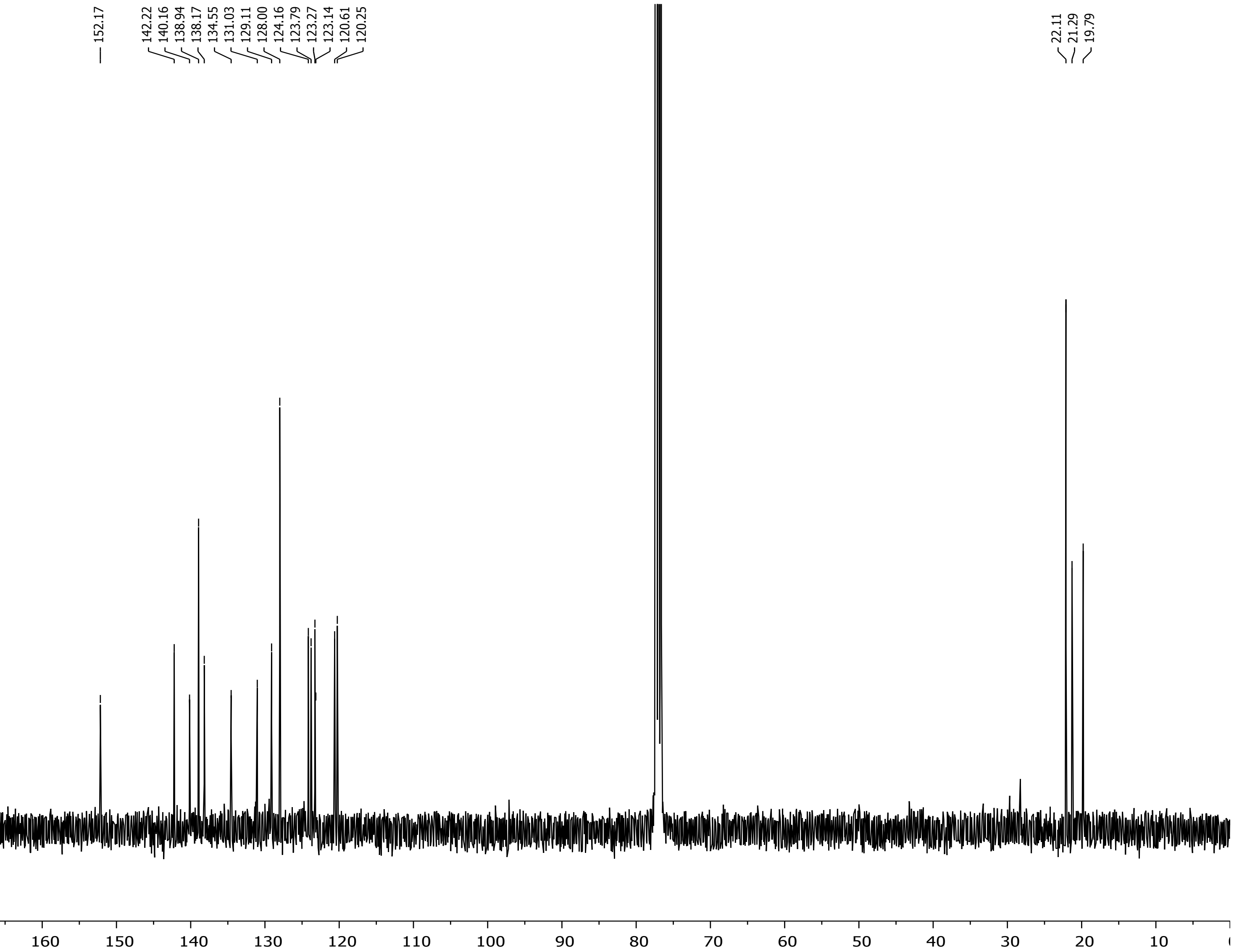

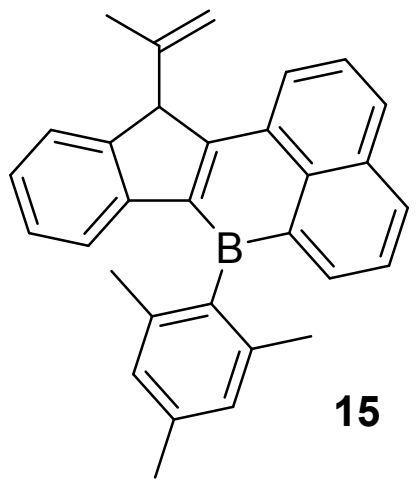

15

^{1}H-NMR (400 MHz, $CDCl_3$)

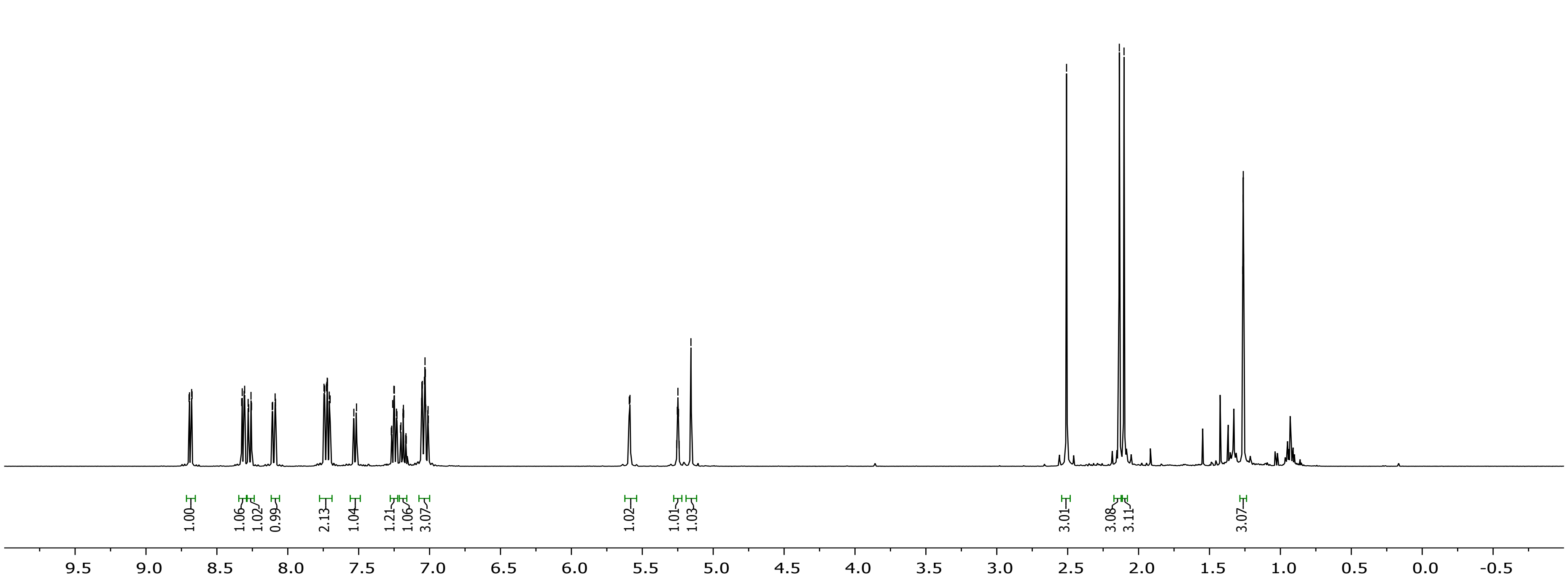

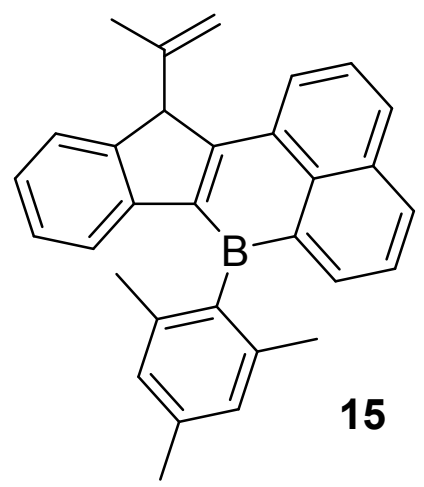

15

^{11}B-NMR (128 MHz, CDCl$_3$)

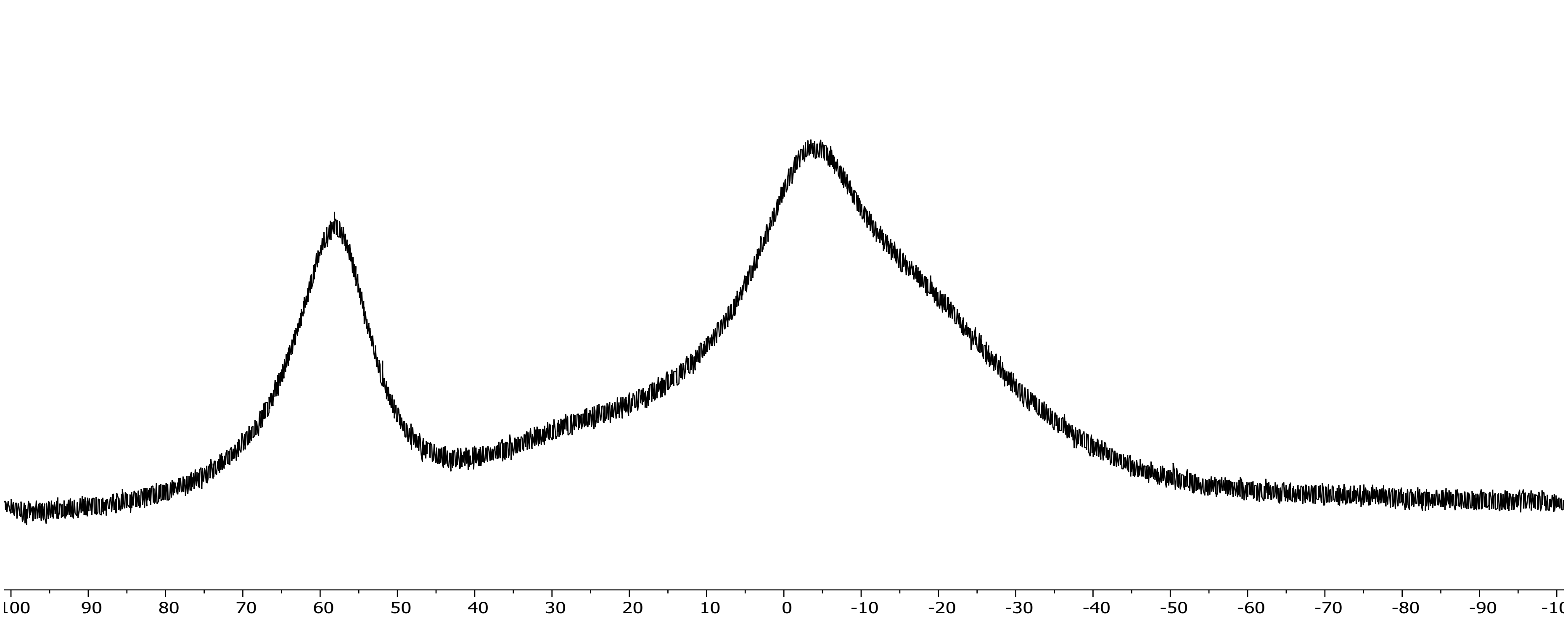

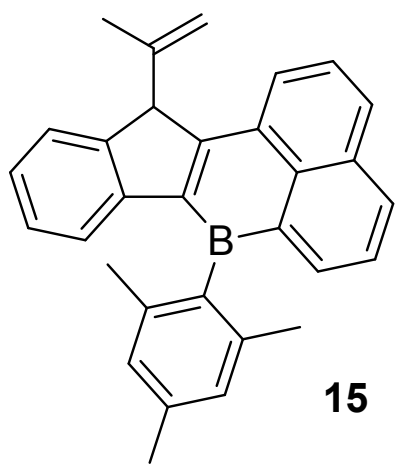

15

^{13}**C-NMR (101 MHz, CDCl$_3$)**

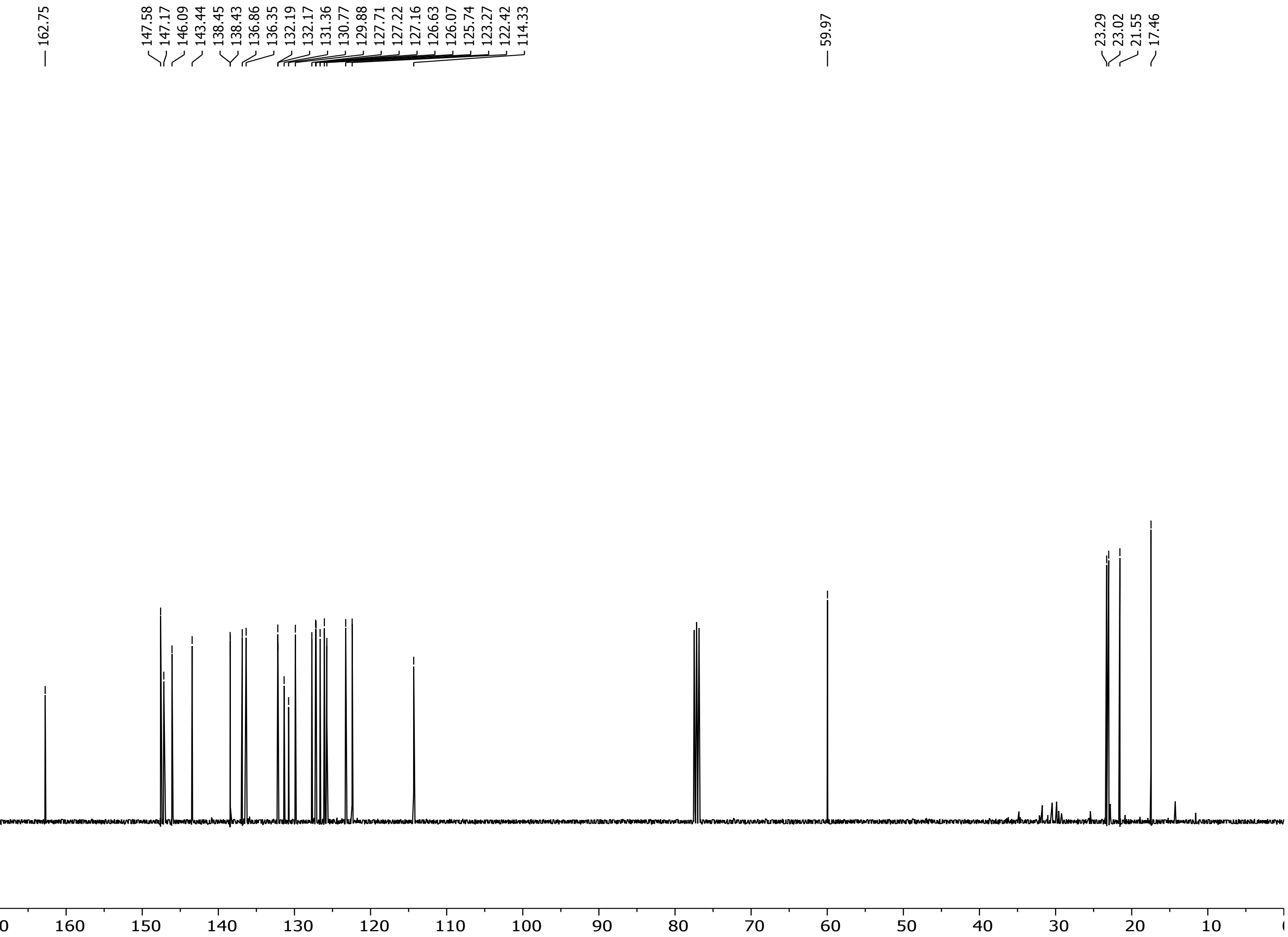

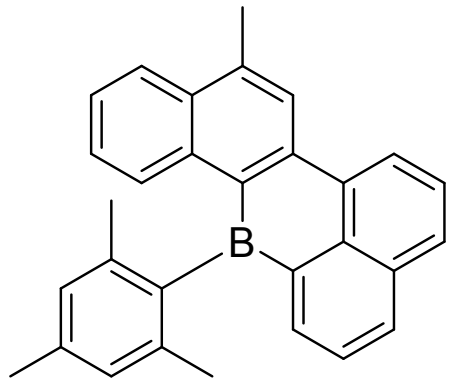

16

^{1}H-NMR (400 MHz, $CDCl_3$)

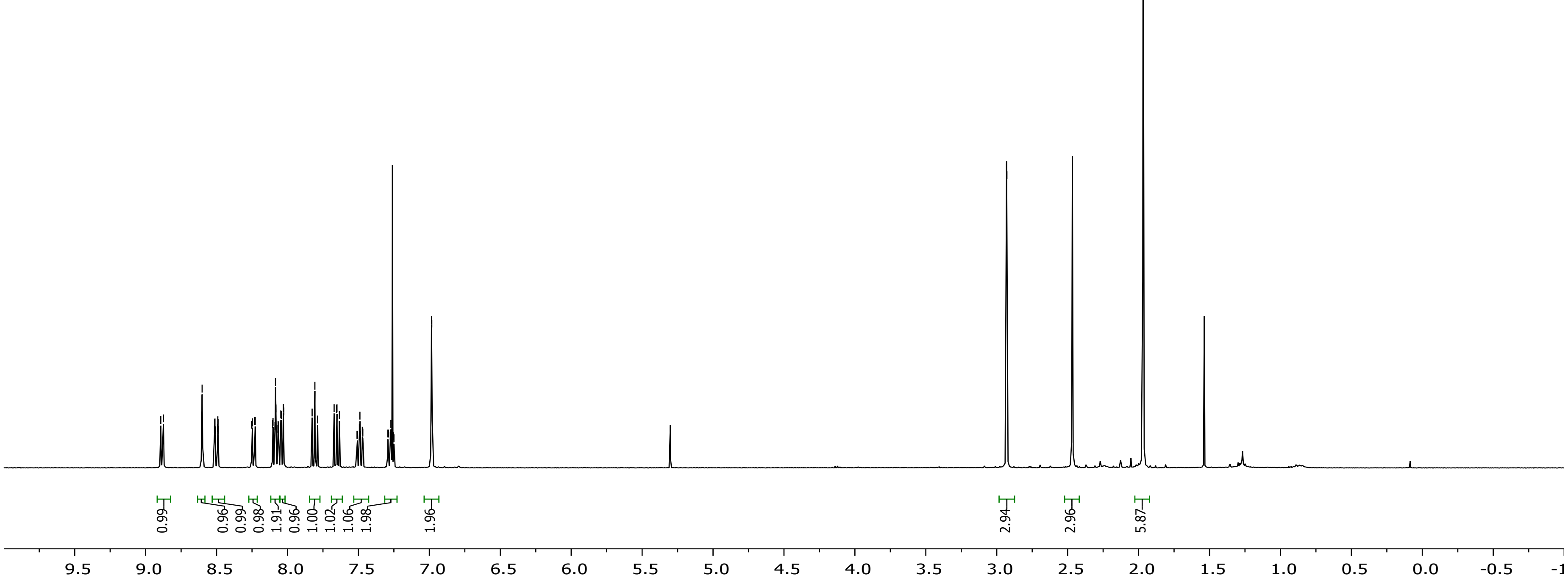

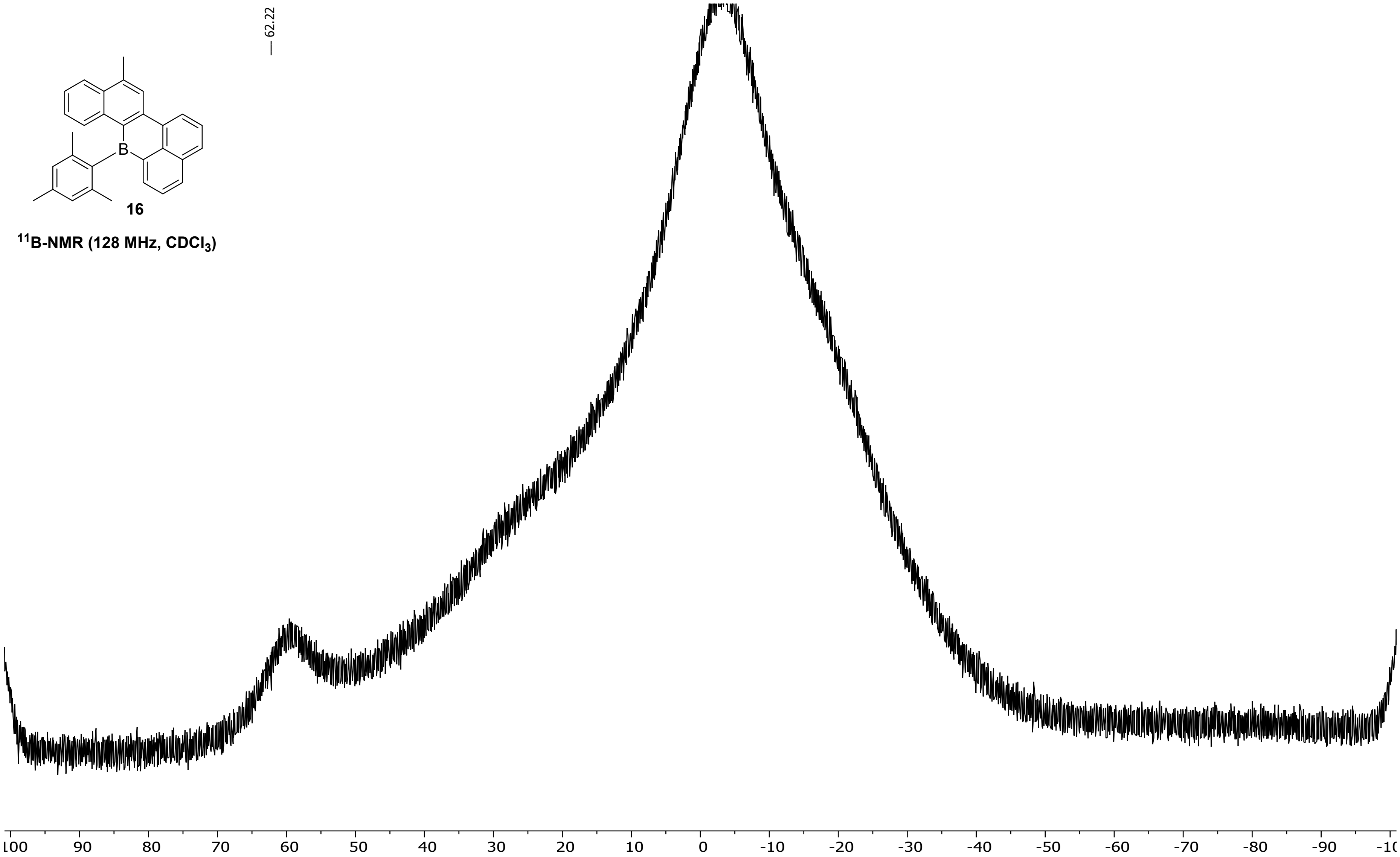

^{11}B-NMR (128 MHz, $CDCl_3$)

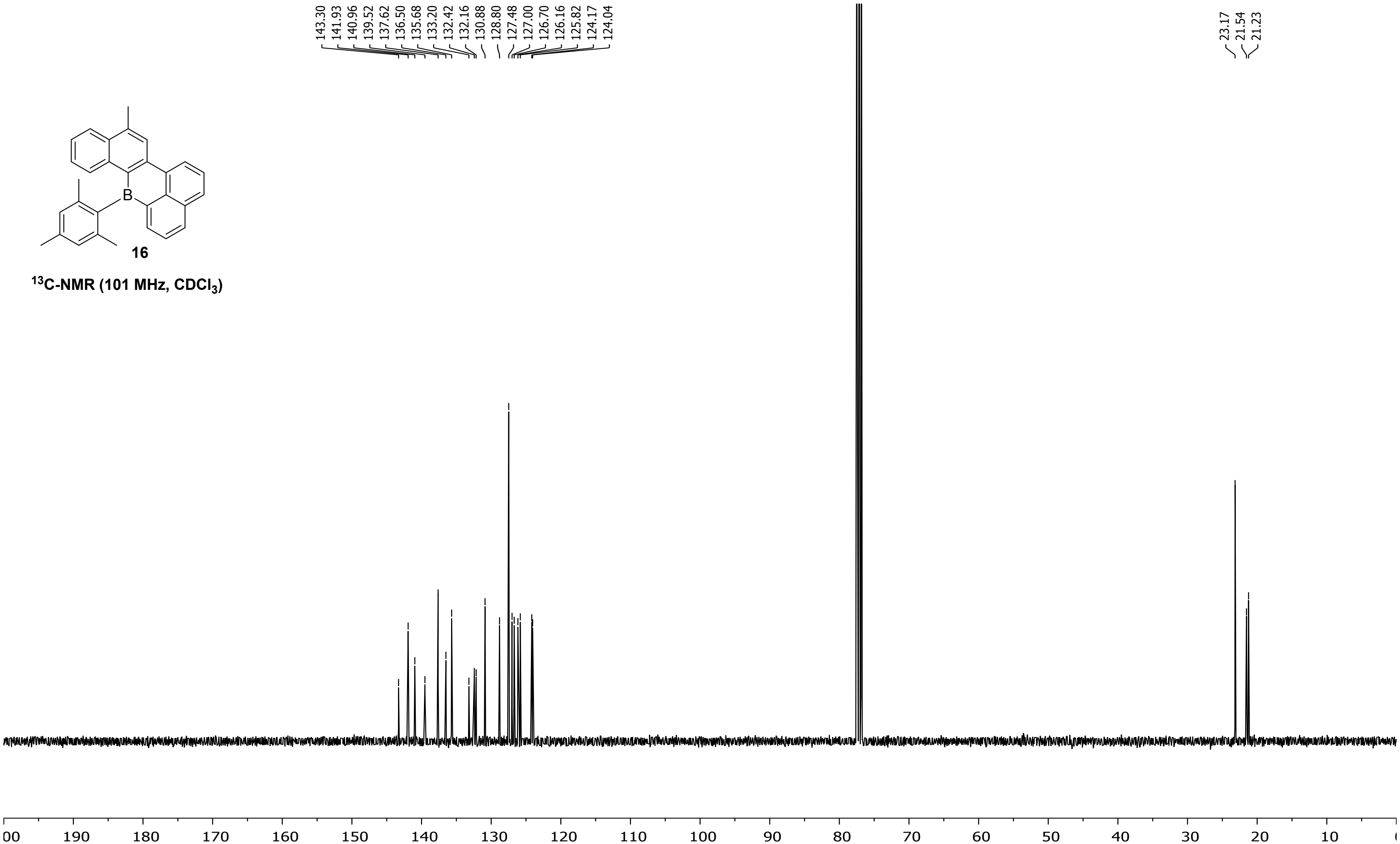

16

^{13}C-NMR (101 MHz, $CDCl_3$)
